# Profiling of BDQ-induced transcriptome suggests amino acid metabolism and stress responses as alternate mechanisms contributing to BDQ tolerance in *Mycobacterium tuberculosis*

**DOI:** 10.1128/spectrum.01455-25

**Published:** 2025-12-16

**Authors:** Dania Khalid Saeed, Sadia Shakoor, Javaria Ashraf, Zahra Hasan, Rumina Hasan

**Affiliations:** 1 Department of Pathology and Laboratory Medicine, The Aga Khan University 9615, Karachi, Pakistan; 2 Faculty of Infectious and Tropical Diseases, London School of Hygiene and Tropical Medicine 4906, London, United Kingdom; Universita degli Studi dell'Insubria, Varese, Italy

**Keywords:** *Mycobacterium tuberculosis*, metabolic fluxes, stress pathways, BDQ-induced transcriptional changes, bedaquiline resistance

## Abstract

Under bedaquiline (BDQ) pressure, a temporary persistence period (24–96 h) has been observed, during which H37Rv undergoes metabolic rerouting. However, little is known of transcriptomic changes in BDQ-resistant *Mycobacterium tuberculosis* (Mtb) isolates during this period. We explored transcriptomic adaptations occurring under inhibitory concentrations of BDQ to delineate pathways supporting drug tolerance and contributing to BDQ resistance. We report overexpression of genes involved in the biosynthesis of L-arginine and L-cysteine in our study isolates. Among stress response genes, genes from the *suf* operon, involved in Fe-S biogenesis, were upregulated in the study isolates. Differentially expressed amino acid gene clusters likely indicate an under-recognized metabolic pathway contributing to BDQ persistence in the study clinical isolates. Furthermore, Fe-S stress response activated under BDQ pressure may be of particular interest as a mechanism broadly used by Mtb in mitigating different environmental stresses. We propose that these pathways should be explored further as potential drug targets.

**IMPORTANCE:**

Keeping in mind the complex interplay between mutations, gene expression, and drug resistance, knowledge of pathways induced under bedaquiline (BDQ) stress in BDQ-resistant clinical *Mycobacterium tuberculosis* (Mtb) isolates is limited. Furthermore, focusing on mechanisms supporting tolerance can help identify potential targets for drugs that act against dormant bacilli or select synergistic drug combinations. Such information may be useful in identifying other alternate mechanisms of resistance and tolerance. Our study explores changes occurring in the transcriptome of BDQ-resistant isolates exposed to inhibitory concentrations of BDQ under a specific tolerance time point. Our study identifies differentially expressed pathways and genes that are: (i) similarly expressed in both H37Rv strain and clinical isolates, (ii) expressed only in clinical isolates, and (iii) reported to be similarly induced by literature in Mtb exposed to other anti-tuberculosis drugs. These genes and pathways present themselves as potential markers that may have diagnostic, prognostic, and therapeutic value that can be explored further.

## INTRODUCTION

Drug-resistant tuberculosis (DR-TB) is a serious public health concern (1–3). A high proportion of the global rifampin/multidrug-resistant (RR/MDR) tuberculosis (TB) burden is reported from densely populated and low-middle-income countries having limited access to diagnostics and treatment (2, 3). Until recently, treatment of MDR TB (defined as TB resistant to rifampicin [RIF] and isoniazid [INH] [2, 3]) required 18–20 months of therapy involving more than five drugs (4). The endorsement of the all-oral 6/9 months bedaquiline (BDQ), pretomanid, linezolid/with moxifloxacin (BPaL/BPaLM) regimens for treating RR/MDR TB, by the WHO in 2022 (5), is thus a significant milestone in steps toward addressing the current DR-TB epidemic.

Among the BPaL/BPaLM drugs, both BDQ and pretomanid have contributed to significantly shortening therapy time (6). BDQ-based regimens have been linked to an increase in the global success rate for treating MDR TB from 50% to 59% (7), underscoring the effectiveness of BDQ. However, concerns over the future efficacy of the BPaL/BPaLM regimen are raised by reports of baseline (8–10) and acquired resistance to BDQ in TB patients (11, 12). While variants in several genes, including *atpE*, *pepQ*, *rv0678*, and *rv1979c* (13), are linked to BDQ resistance, reports of isolates phenotypically resistant to BDQ without any identified genetic basis (14–16) indicate the need to consider additional mechanisms of resistance.

The ability of *Mycobacterium tuberculosis* (Mtb) to develop resistance to multiple drugs is attributed to its metabolic flexibility (17). As an adaptive strategy of survival under stress conditions, including drug pressure, Mtb enters a non-replicating state of metabolic quiescence, that is tolerance (18). Additional strategies used by Mtb to mitigate drug stress include rerouting of its central carbon metabolism (CCM) and alterations in cell wall synthesis (19–21). Transcriptional changes reflecting modifications in redox state, cell wall permeability, and a decrease in protein synthesis have been described by earlier *in vitro* studies to support tolerance to front line TB drugs, including RIF (22, 23) and INH (24, 25). Conversely, metabolic fluxes particularly in the CCM are reported to support bactericidal activity of drugs (26–28). Under BDQ pressure, a transient drug-tolerant lag phase (24–96 h) is observed for both BDQ-sensitive (29, 30) and BDQ-resistant (31) strains. During this time, metabolic re-routing is reported (29) to establish temporary BDQ tolerance. Simultaneously, glycolytic vulnerabilities have also been described to contribute to BDQ susceptibility (32).

Differences in transcriptional responses between drug-resistant and susceptible isolates (33) and between H37Rv and clinical isolates (23, 34) have also been reported. However, there is a paucity of transcriptomic and metabolomic studies exploring pathways induced under drug pressure among clinical isolates including drug-resistant isolates (23, 30, 33, 35). Our previous work on the growth-inhibition pattern of BDQ-resistant clinical isolates reported an extended killing phase (24–96 h), similar to that of control drug-sensitive lab strain H37Rv (31). These findings suggest that the temporary lag period could be used as a model for exploring BDQ-induced transcriptional responses in clinical isolates. Such models may help study under-recognized pathways contributing to drug resistance. To identify genes and pathways that may support BDQ tolerance and by extension resistance to it, we profiled the transcriptome of BDQ-resistant strains under BDQ stress and compared gene expression profiles with that of H37Rv reference strain after 72 h of BDQ pressure.

## RESULTS

Whole transcriptome sequencing of BDQ-resistant clinical isolates (*n* = 6) and H37Rv was conducted followed by differential gene expression analysis as presented in Fig. S1.

### Principal component analysis and distance matrix graph

**Fig 1:**
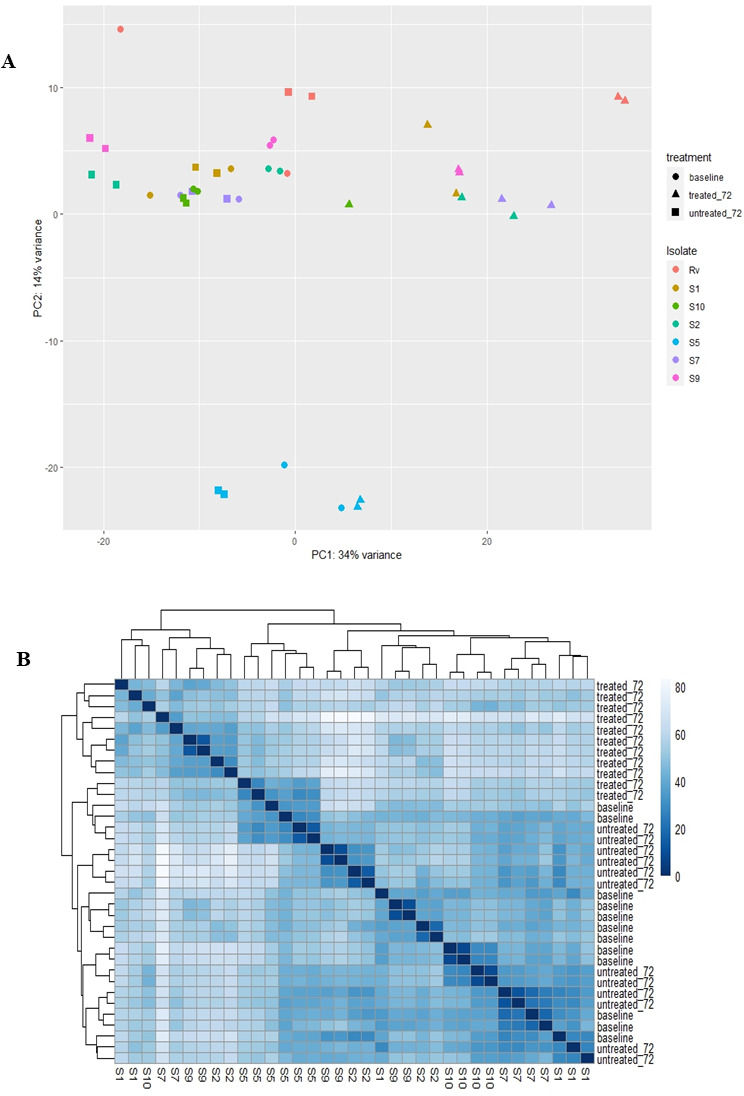
Visual assessment of transcriptomic data from Mtb study isolates (BDQ-resistant isolates; *n* = 6 and H37Rv) using PCA and distance matrix graph. (**A**) PCA plot. This plot shows variance between samples. It is also used as an initial assessment of clustering shown by Mtb isolates to experimental conditions; baseline (isolates at *t* = 0 h), control (BDQ-unexposed isolates, *t* = 72 h), after 72 h of BDQ exposure. Shapes depict experimental condition. Circle mid-log growth isolates before BDQ exposure, triangle = isolates after 72 h of BDQ exposure, square unexposed control isolates after 72 h of growth. Different colors have been assigned to differ. (**B**) Visual depiction of sample variation and clustering for isolates (including H37Rv) as shown in the key and experimental conditions as a distance matrix graph. Bioinformatics analysis was carried out using DESeq (36) in R studio vr. 4.3.0.

No significant variation between biological and experimental duplicates of Mtb isolates was seen in the principal component analysis (PCA) plot (Fig. 1A) except for clinical Mtb isolate S5, which was an outlier and presents as a separate cluster. There was low variation between isolates at baseline (*t* = 0 h) and controls (*t* = 72 h).

The PCA plot (Fig. 1A) and distance matrix (Fig. 1B) both show clustering of isolates based on BDQ exposure. BDQ-exposed isolates clustered as a separate group, while unexposed controls (*t* = 72 h) and baseline isolates (*t* = 0 h) did not show any distinct clustering.

### Clustering in hierarchical heat maps

The clustering of transcriptome data of isolates in the hierarchical heat maps also supported observations from the PCA and distance matrix graph. Isolates were separated into distinct BDQ exposed (*t* = 72 h) and unexposed controls groups (*t* = 72 h; Fig. S2). While random clustering of isolates was observed for unexposed controls (*t* = 72 h) and baseline isolates (*t* = 0 h; Fig. S2).

### Differential gene expression analysis in Mtb isolates

#### Transcriptional profiles post 72 h of growth in 7H9 liquid media without BDQ pressure

Transcriptional profiles of BDQ-resistant clinical isolates and H37Rv were studied (without BDQ pressure) at *t* = 72 h of growth and compared with their baseline growth at *t* = 0 h. Among BDQ-resistant clinical isolates, genes involved in starvation stress (*grpE*, *cysD*, *cysN*, and *mbtI*), heat stress (*hsp*, *dnaK*, *dnaJ2*, *sigB*, and *clgR*), oxidative stress (*hsp*, *dnaK*, *mmcO*, *cysD*, *cysN*, and *clgR*), pH (*clgR* and *prpD*), and genes encoding detoxification proteins (*mmcO* and *mazE6*) were significantly downregulated (Table 1; Table S1). Similarly, among H37Rv, stress response genes involved in starvation (*pckA*, *ppE51*, *tcrX*, and *pks2*), oxidative stress (*dsbF*), and pH (*lipF*) were significantly downregulated (Table 1; Table S1).

**TABLE 1:** Stress response genes differentially regulated (absoluteLog2FoldChange > 1, adjusted *P*-value < 0.05) in BDQ-resistant isolates (*n* = 6) and H37Rv after 72 h of *in vitro* growth in 7H9 liquid media^*a*^

Stress responses	BDQ-resistant isolates		H37Rv	
	Genes	L_2_Fc	Genes	L_2_Fc
Starvation	*grpE*	−1.12	*pckA*	−1.39
	*mbtI*	−1.06	*PPE51*	−3.54
	*cysD*	−1.31	*tcrX*	−1.28
	*cysK*	−1.14	*pks2*	−3.22
Oxidative stress	*hsp*	−1.17	*dsbF*	−1.73
	*dnaK*	−1.08		
	*clgR*	−1.30		
	*cysD*	−1.30		
	*cysK*	−1.14		
pH	*clgR*	−1.30	*lipF*	−2.98
	*prpD*	−1.08		
Heat stress	*hsp*	−1.17	NF	
	*dnaK*	−1.08		
	*dnaJ2*	−1.01		
	*sigB*	−1.38		
	*clgR*	−1.30		
Detoxification	*mazE6*	−1.06	NF	

^*a*^ L_2_Fc, Log2FoldChange; NF, not found.

#### Transcriptional profiles post 72 h of exposure to BDQ (3.75 µg/mL)

To investigate changes in genetic expression after 72 h of BDQ exposure, profiles of BDQ exposed isolates (*t* = 72 h) and unexposed controls (*t* = 72 h) were compared. Significantly differentially expressed genes (DEGs; absolute Log2FoldChange [L_2_Fc] >1, adjusted *P*-value [*P*-adjusted] <0.05) were identified.

**Fig 2:**
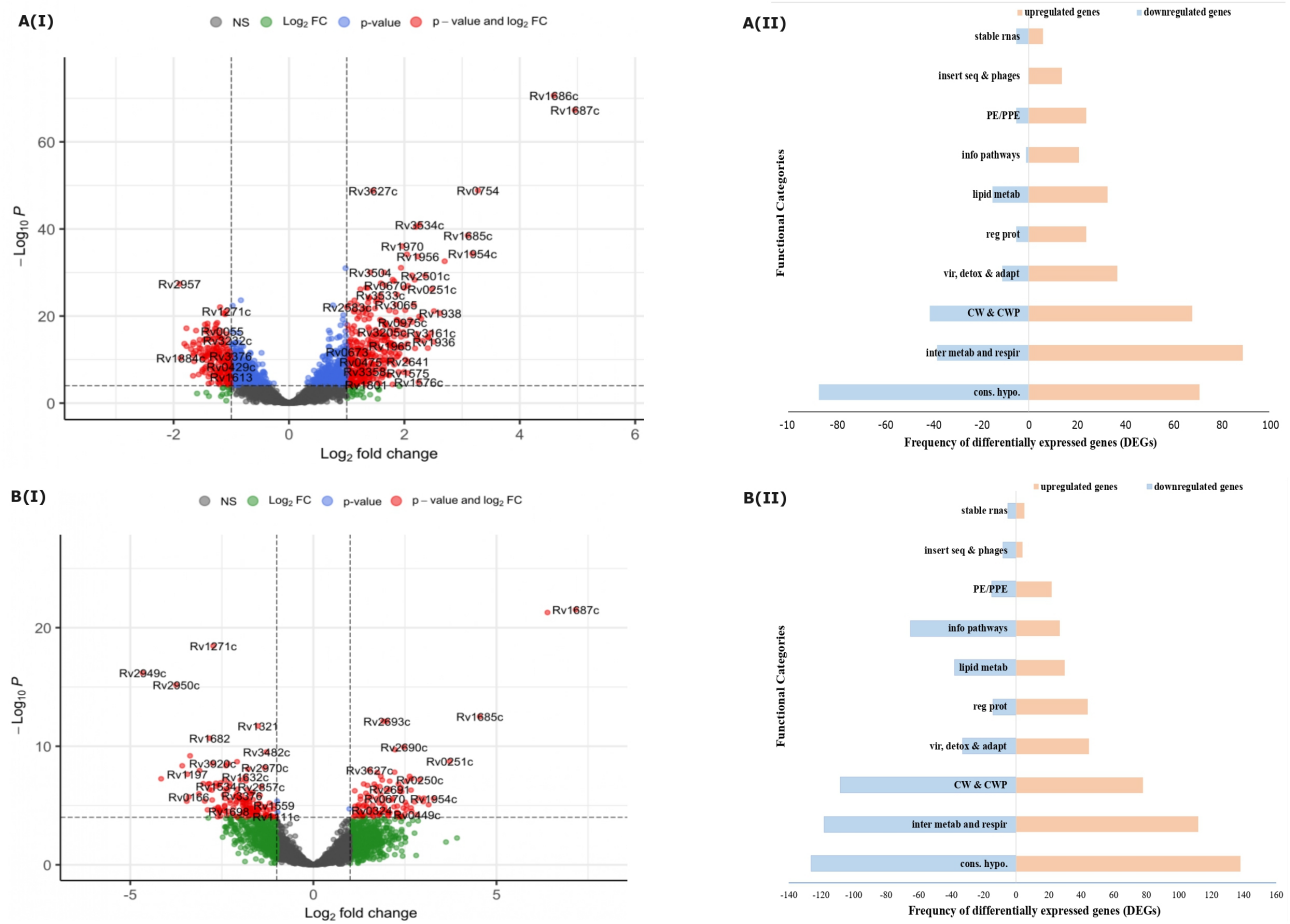
Distribution and categorization of significantly DEGs induced under BDQ pressure (3.75 µg/mL, *t* = 72 h) in Mtb isolates (BDQ-resistant clinical isolates; *n* = 6 and H37Rv). A(I) and B(I) depict volcano plots of DEGs in BDQ-resistant clinical isolates and H37Rv lab drug-susceptible strain, respectively, after 72 h of BDQ exposure (3.75 µg/mL) compared to the corresponding control (without BDQ exposure). Dotted lines represent the thresholds for significance of differential expression. Absolute log2 fold change (abs. L2Fc) and adjusted *P*-value (adj. *P*-value) thresholds for considering significantly DEGs. Vertical dotted lines indicate cut-off for abs. L2Fc set at >1. Horizontal dotted lines represent cut-offs for adj. *P*-value taken as <0.05. X-axis (Log2 fold change) indicates the mean of each DEG determined collectively for BDQ-exposed clinical isolates, including their biological duplicates (*N* = 6). For H37Rv, L2Fc indicates the mean of each DEG for BDQ-exposed biological duplicates. Each individual dot represents a single gene. These are color coded as follows: red dots are significantly differentially regulated DEGs (abs. L2Fc 1, adj. *P*-value < 0.05), and blue dots are DEGs with abs. L2Fc < 1 and adj. *P*-value < 0.05. Green dots represent genes with abs. L2Fc > 1 and adj. *P*-value > 0.05. In gray are genes with adj. *P*-value > 0.05 and abs. L2Fc < 1. Some of the significantly DEGs have been labeled in the volcano plot. **A**(II) and** B**(II) show the distribution and frequency of BDQ-induced significantly DEGs into functional categories using MycoBrowser (37) for BDQ-resistant clinical isolates and H37Rv. Stable rnas, stable ribosenucleic acids; insert seq and phages, insertion sequences and phages; PE/PPE, proline-glutamic acid/proline-proline-glutamic acid protein families; info pathways, information pathways; lipid metab, lipid metabolism; reg prot, regular proteins; vir, detox and adapt, virulence, detoxification and adaptation; CW and CWP, cell wall and cell wall processes; inter metab and respir, intermediate metabolism and respiration; cons. hypo., conserved hypotheticals.

Among H37Rv and BDQ-resistant isolates, the distribution of DEGs is visually presented: (i) across the defined thresholds by volcano plots (Fig. 2A[I] and Fig. 2B[I]) and into different functional categories, as defined by Mycobrowser (37) (Fig. 2A[II] and Fig. 2B[II]).

##### BDQ-resistant isolates

After 72 h of BDQ exposure, 595 genes were significantly differentially expressed in BDQ-exposed clinical isolates compared to their unexposed controls (Fig. 2; Table S2). Of the DEGs, 387 genes were upregulated, while 208 genes were downregulated (Fig. 2; Table S2). Functional categories with the highest distribution of genes included conserved hypotheticals (26.6%), intermediate metabolism and respiration (21.3%), and cell wall and cell processes (18.3%; Fig. 2; Table S2).

##### H37Rv

After 72 h of BDQ exposure, 1,035 genes were significantly differentially expressed (L_2_Fc > |1|, *P*-adjusted < 0.05; Fig. 2; Table S3). Of these, 505 genes were upregulated, while 530 genes were downregulated (Fig. 2; Table S3). The majority of these DEGs, aggregated into the functional categories of conserved hypotheticals (25.5%), intermediate metabolism and respiration (22.2%), and cell wall and cell processes (18.0%; Fig. 2; Table S3).

### Comparison of differential gene expression of BDQ-exposed clinical isolates with H37Rv

DEGs identified in BDQ-resistant clinical isolates and in H37Rv following 72 h of BDQ exposure were then compared. Significantly DEGs (*n* = 325) were identified in both BDQ-resistant clinical isolates and in H37Rv (upregulated genes = 208 and downregulated genes = 116; Fig. S3; Table S4[I]). These DEGs, while varying in magnitude of expression, did not show any difference in directionality of expression, whereas 140 DEGs (upregulated genes = 95 and downregulated genes = 43) were identified that were limited to BDQ-resistant isolates only (Table S4[II]).

### DEGs mapped onto pathways/systems

Genes indicated to be significantly differentially expressed under BDQ pressure were studied in context of their pathways and/or systems (Table 2).

**TABLE 2:** Pathways and systems to which DEGs (absolute Log2FoldChange > 1, adjusted *P*-value < 0.05) after 72 h of BDQ exposure (3.75 µg/mL) in H37Rv and BDQ-resistant clinical isolates (*n* = 6) were mapped^*a*^^,*b*^

Pathways/systems		DEGs					
		BDQ-resistant clinical isolates		Both H37Rv and BDQ-resistant isolates		H37Rv	
		Upreg	Downreg	Upreg	Downreg	Upreg	Downreg
Lipid/FA metabolism	β-oxidation	*fadE17*, *fadE12*, *fadE8*, *echA4*, and *fadE17*	–	*fadB3*, *fadE19*, *fadE13*, *fadE9*, *fadE5*, *echA13*, *echA7*, *fadE26*, *fadE27*, *fadE34*, *fabD2*, and *fadD7*	*fadD5* and *plcB*	*fadA5*, *fadB5*, *fadD19*, *fadD18*, *fadD24*, *fadD7*, *fadE15*, *fadE6*, and *fadE1*	*fadE35*, *fadE16*, *tesB1*, *echA1*, *echA6*, *fadD21*, *fadD25*, *fadD22*, *fadD29*, and *fadD30*
	Cholesterol deg.	*fadE29* and *fadE22*	–	*fadA5*, *fadB3*, *fadE34*, *fadD19*, *fadE26*, *fadE27*, *hsaE*, *hsaG*, and *hsaF*	–	–	–
	Propanoyl deg.	*accA2*, *accD1*, *accD2*, and *mutA*	–	*accA1* and *faBD2*	–	–	–
Amino acid metabolism		–	–	*dapA*, *serS*, *thrS*, *alaS*, and *gcvB*	*proC*	–	*asnB*
	Tyrosine biosyn.	*aroF* and *aroK*	–	–	–	–	–
	L-arginine biosyn.	*argF*, *carA*, and *carB*	–	*argC*, *argB*, *argD*, and *argJ*	–	–	–
	L-glutamine deg.	*hisF*	–	–	–	–	–
	L-his biosyn.	*hisF*	–	*hisD*	–	–	–
	L-tryp biosyn.	*aroE*, *aroK*, and *aroF*	–	–	*rv0948*, *rv2949c*, *trpC*, *trpA*, and *trpB*	–	*proA*, *proC*, and *aroG*
	L- val deg.	–	–	*bkdA*, *bkdB*, *bkdC*, *mmsA*, *mmsB*, and *rv2498*	–	–	–
	L-isoleucine and leucine biosyn.	–	–	–	–	*–*	*ilvA*, *ilvB2*, *ilvB1*, *ilvD*, *ilvN*, *leuC*, and *leuD*
	L-cysteine deg.	–	–	*moeB1*, *csd*, and *cysK2*	–	–	*mtn*
Protein synthesis	Protein synthesis	*rrs* and *rrf*	*rpmG2*, *rpmH*, *rpsG*, *rpsS*, *rpsQ*, *moeW*, *rv2959c*, and *moaC3*	*rrl*	*rv0053*, *rv0055*, *rv0056*, *rpsJ*, *rplW*, *rv0704*, *rpsS*, *rplV*, *rpsC*, *rv0708*, *rpmC*, *rpsQ*, *rv0717*, *rv0718*, *rv3924c*, *rpsN1*, and *rpsH*	–	*rpsF*, *rpsE*, *rpsR1*, *rpsA*, and *rv0714-16*
Cell wall biosynthesis	C.W biosynth. and periplasmic proteins	*Rv2533*, *ppm1*, *murF*, and *murE*	*dprE2*, *fadD23*, *mmaA2*, *papA1*, *rv2525c*, *mmpL10*, *mce3A*, *mce3D*, and *lprM*	*ldtA*, *mbtC*, *fadD19*, *pepD*, *htrA*, *bglS*, *mce3A-D*, *mce3F*, and *pks13*	*mmaA3*, *rv1433*, *ripA*, *rv3920c*, *mctB*, *mce1A-D*, *mce1R*, *mce1B*, and *fadD30*	*ufaA1* and *pks16*	*ripB*, *fbpA*, *rv2136c*, *ftsW*, *fadD26*, *drrB*, *papA5*, *fadD28*, *mmpL7*, *lppX*, *pks15*, *ddlA*, *hupB*, *ponA2*, *aftB*, *ubiA*, *mceR*, and *mmaA4*
	Phenophthiocerol and phthiocerol biosynth.	*ppsD*	*papA5*	–	*rv2949c*, *fadD29*, *rv2951c*, *rv2952*, and *rv2953*	–	*fadD22*
	pHBAD biosynthesis	–	*rv2959c*	–	*rv2949c*, *rv2954c*, *rv2955c*, *rv2956*, and *rv2957*	–	–
	dTDP-L-rhamnose biosynthesis	–	–	–	*rmlB* and *rmlC*	–	–
	Mycolate biosynthesis	–	*hadB*	*pks13*	*mmaA3*, *cmaA1*, *cmaA2*, *hadA*, and *hadC*	–	*fabG1*, *mmaA4*, *rv2509*, *otsB1*, *glft1*, *aftB*, *fbpA*, *fbpB*, *fbpC*, *mmpL3*, *otsB1*, and *accE5*
	Polyacyltrehalose synthesis	–	*papA3*	*–*	*–*	*–*	*–*
T.Rs	Transcriptional regulatory proteins	*rv1129c*, *rv1657*, *rv2250c*, *rv2912c*, and *rv3066*	*whiB2, rv0891c, rv3058c,*	*rv0275c*, *rv0324*, *rv0767c*, *rv1460*, *rv1956*, *rv1957*, *rv1985c*, *clgR*, *furA*, *rv2324*, *rv2642*, *rv3160c*, *rv3183*, *csoR*, *cmtR*, *pknK*, *mce1R*, and *mce3R*	*rv0144*, *rv0165c*, and *rv1830*	*rv1956*	*–*
Stress response	Sigma factors	*sigF*	–	*sigB* and *sigE*	*sigC*	*sigH*	*sigD*
	Osmotic stress	–	–	*hsp*	–	–	*dsbF*, *hspX*, and *otsA*
	Temperature stress	*dnaJ1*, *dnaJ2*, *groES*, and *sigF*	–	*hsp*, *clgR*, *groEL1*, *groEL2*, *sigB*, *sigE*, *clpB*, and *dnaK*	*sigC*	*hsp22.5* and *sigH*	*hspX*, *sigD*, and *otsA*
	DNA damage	–	–	*rv0142*, *end*, *udgB*, *dnaE2*, *rv2559c*, *rv3201c*, *rv3202c*, *rv3204*, and *ligB*	–	*recD*, *recB*, *recC*, *mpg*, *rv2464c*, *ligA*, *ligB*, *rv3201c*, and *rv3202c*	*recA*, *recF*, *ssb*, *dnaB*, *mku*, *ruvA*, *ruvC*, *rnhB*, *mutT1*, *otsA*, and *lexA*
	Detoxification	*panD1*, *adhD*, and *rv2195c*	–	*mmcO*, *ctpV*, *higB*, *mazE6*, and *rv2688c*	–	*rv0194*, *mca*, *parD1*, and *vapB20*	*ahpD*, *ahpE*, *rv1676*, *hpx*, *dsbF*, *sodC*, and *sodA*
	Oxidative stress	*sigF*, *rv2159c*, *rv2455c*, and *cysD*	–	*katG*, *hsp*, *dnaK*, *mmcO*, *sigE*, and *furA*	–	*rv2464c*, *ideR*, *sigH*, *lsr2*, *ftsH*, *dipZ*, *rv3177*, and *bpoA*	*ahpE* and *ahpD*
	pH stress	*icl1* and *prpD*	*rv3378c*	*clgR* and *lipV*	–	*virS*	*mprA*
	Starvation stress	*PE_PGRS33, sigF*	–	*grpE*, *rv2557*, *rv2558*, *mbtB*, and *htmA*	–	*relA* and *mbtA*	*hspX*, *pstS1*, and *hupB*
Fe-S complex biogenesis	SUF operon	–	–	*rv1461-rv1466*	–	–	–
Transporter proteins and efflux pumps		–	–	*rv1686c*, *rv1687c*, *mscL*, *rv1463*, *rv0037*, and *mmr*	*rv0072*, *rv1698*, *rv1857*, *rv2456*, and *rv0403*	–	–

^*a*^ BioCyc platform (38) was used for mapping DEGs onto pathways and systems.

^*b*^ FA, fatty acid; deg, degradation; biosyn, biosynthesis; his, histidine; tryp, tryptophan; val, valine; C.W., cell wall; dTP, deoxythymidine diphospho; pBAD, para hydroxybenzoic acid-derivatives; T.Rs, transcriptional regulators; Fe-S, iron-sulfur; –, not detected.

#### Metabolic pathways

Under BDQ pressure, upregulation of several genes involved in cholesterol degradation, fatty acid biosynthesis, and β-oleate oxidation was noted (Table 2; Tables S2 and S3). Furthermore, overexpression of malate quinone reductase encoding gene *mqo* was seen in both H37Rv and in BDQ-resistant isolates. Upregulation of isocitrate lyase (ICL; L_2_Fc: 2.21) was noted in BDQ-exposed clinical isolates, indicating a shift in metabolism from oxidative to substrate-level phosphorylation.

**Fig 3:**
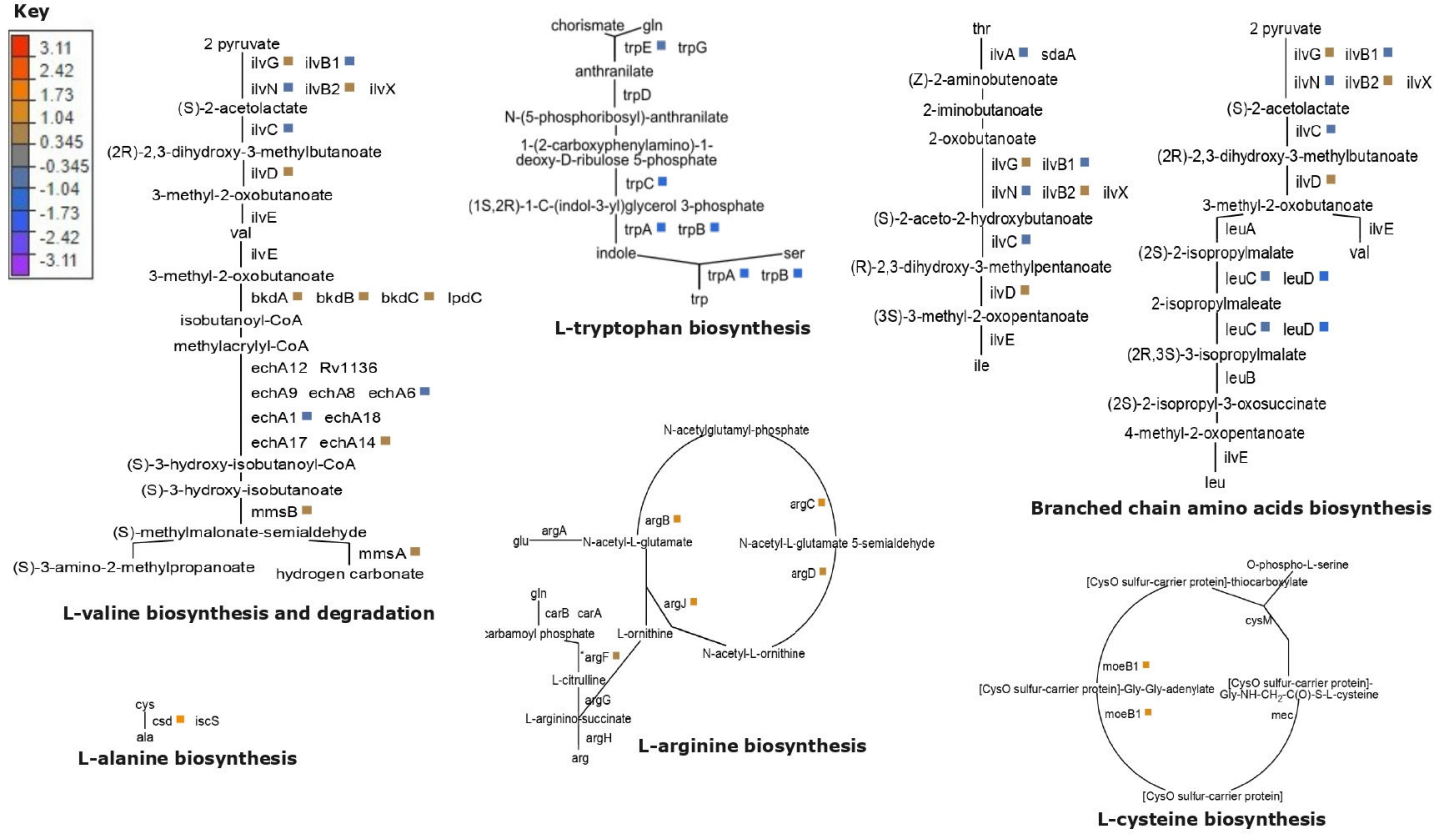
Genes from amino acid metabolic pathways differentially expressed after 72 h of BDQ exposure (3.75 g/mL) in study isolates (BDQ-resistant isolates and H37Rv). Genes were mapped onto pathways using BioCyс (38). Color keys in the figure represent the Log2FoldChange (L2Fc) in expression level of differentially regulated genes shown in the metabolic pathways.

At 72 h of BDQ pressure, differential expression of genes involved in amino acid metabolism was noted (Table 2). In both BDQ-resistant isolates and H37Rv, tryptophan biosynthesis was suppressed as indicated by the downregulation of *trpA-C* and *trpE* (L_2_Fc < −1, adj. *P* < 0.05; Table 2; Table S2; Fig. 3). While *csd* and *moeB1*, involved in the biosynthesis of L-alanine and L-cysteine, respectively, were significantly upregulated in BDQ-resistant isolates (*csd*; L_2_Fc: 2.03, *moeB1*; L_2_Fc: 1.60) and H37Rv (*csd*; L_2_Fc: 1.69, *moeB1*; L_2_Fc: 1.91; Fig. 3). Furthermore, biodegradation of L-valine was also indicated in BDQ-exposed clinical isolates (*mmsA*; L_2_Fc: 1.72, *mmsB*; L_2_Fc 1.72, *bkdA*; L_2_Fc: 1.91, *bkdB*; L_2_Fc: 1.82, *bkdC*; L_2_Fc: 1.41) and H37Rv (*mmsA*; L_2_Fc: 1.73, *mmsB*; L_2_Fc: 1.54, *bkdA*; L_2_Fc: 1.89, *bkdB*; L_2_Fc: 1.80, *bkdC*; L_2_Fc: 1.52; (Table 2; Table S2; Fig. 3).

Among study isolates (BDQ-resistant and H37Rv), L-arginine biosynthesis (BDQ-resistant isolates; *argB*; L_2_Fc: 1.70, *argC*; 1.40, *argD*; L_2_Fc: 1.40, *argF*; L_2_Fc: 1.15, *argJ*; L_2_Fc: 1.60, H37Rv; *argB*; L_2_Fc: 1.32, *argC;* 1.53, *argD*; L_2_Fc: 1.02, *argJ*; L_2_Fc: 1.34) was observed to be significantly upregulated (Fig. 3). Moreover, several genes (*aroE*; L_2_Fc: 1.1, *aroF;* L_2_Fc: 1.2, *aroK;* L_2_Fc: 1.08) from the shikimate pathway, involved in aromatic amino acid biosynthesis (39), were differentially upregulated in BDQ-resistant clinical isolates only.

Under BDQ pressure, protein synthesis was suppressed in both BDQ-resistant clinical isolates and H37Rv as indicated by the downregulation of genes encoding ribosomal RNA (rRNA) subunits (Table 2; Table S2). These included 17 genes in BDQ-resistant clinical isolates and 33 genes in H37Rv. Of the differentially expressed rRNA encoding genes, *rrs* (L_2_Fc: 1.80) and *rrl* (L_2_Fc: 1.95) were upregulated in BDQ-resistant isolates. While in H37Rv, only *rrl* (L_2_Fc: 1.52) was upregulated (Table 2; Table S3).

#### Cell wall biosynthesis

The downregulation of several genes involved in cell structure biosynthesis (Table 2; Tables S2 and S3) was indicated in BDQ-exposed study isolates. In BDQ-resistant isolates, *murE* (L_2_Fc: 1.6), *murF* (L_2_Fc: 1.1), *dapA* (L_2_Fc: 1.20) were significantly upregulated (Table 2; Table S4). *ldtA*, encoding L, D transpeptidases involved in catalyzing transpeptide linkages in the peptidoglycan cell wall (40), was noted to be differentially upregulated in both BDQ-exposed clinical isolates (L_2_Fc: 1.15) and H37Rv (L_2_Fc: 1.24; Table 2; Tables S2 and S3).

#### Transcriptional regulators and stress response genes

Several transcriptional regulators were indicated to be differentially regulated among BDQ-resistant isolates and H37Rv (Table 2; Tables S2 and S3). Additionally, several differential expressions of stress response genes, including those involved in nutritional, heat, oxidative, DNA damage, and pH stress, were identified to be upregulated (Table 2; Tables S2 and S3).

After 72 h of exposure to BDQ, genes encoding sigma factors; *σ^E^*, *σ^B^*, and *σ^F^* were significantly upregulated in both BDQ-resistant clinical isolates and H37Rv (Table 2; Tables S2 and S3). Similarly, stress response genes controlled by *σ^E^* including: *clgR* (BDQ-resistant isolates; L_2_Fc: 1.75, H37Rv; L_2_Fc: 3.70), *dnaK* (BDQ-resistant isolates; and L_2_Fc: 1.41, H37Rv; L_2_Fc: 1.08) and *hsp* (BDQ-resistant isolates; L_2_Fc: 2.48, H37Rv; L_2_Fc: 3.72) were also upregulated.

#### Fe-S complex biogenesis

DEGs in H37Rv and BDQ-resistant isolate genes mapped onto the suf operon of the Fe-S complex biogenesis pathway. The suf operon (sufRBDCSUT; *rv1461-rv1466*) was upregulated in both BDQ-resistant isolates and H37Rv (Table 2; Table S2; Fig. 3).

#### Transporter proteins

**Fig 4:**
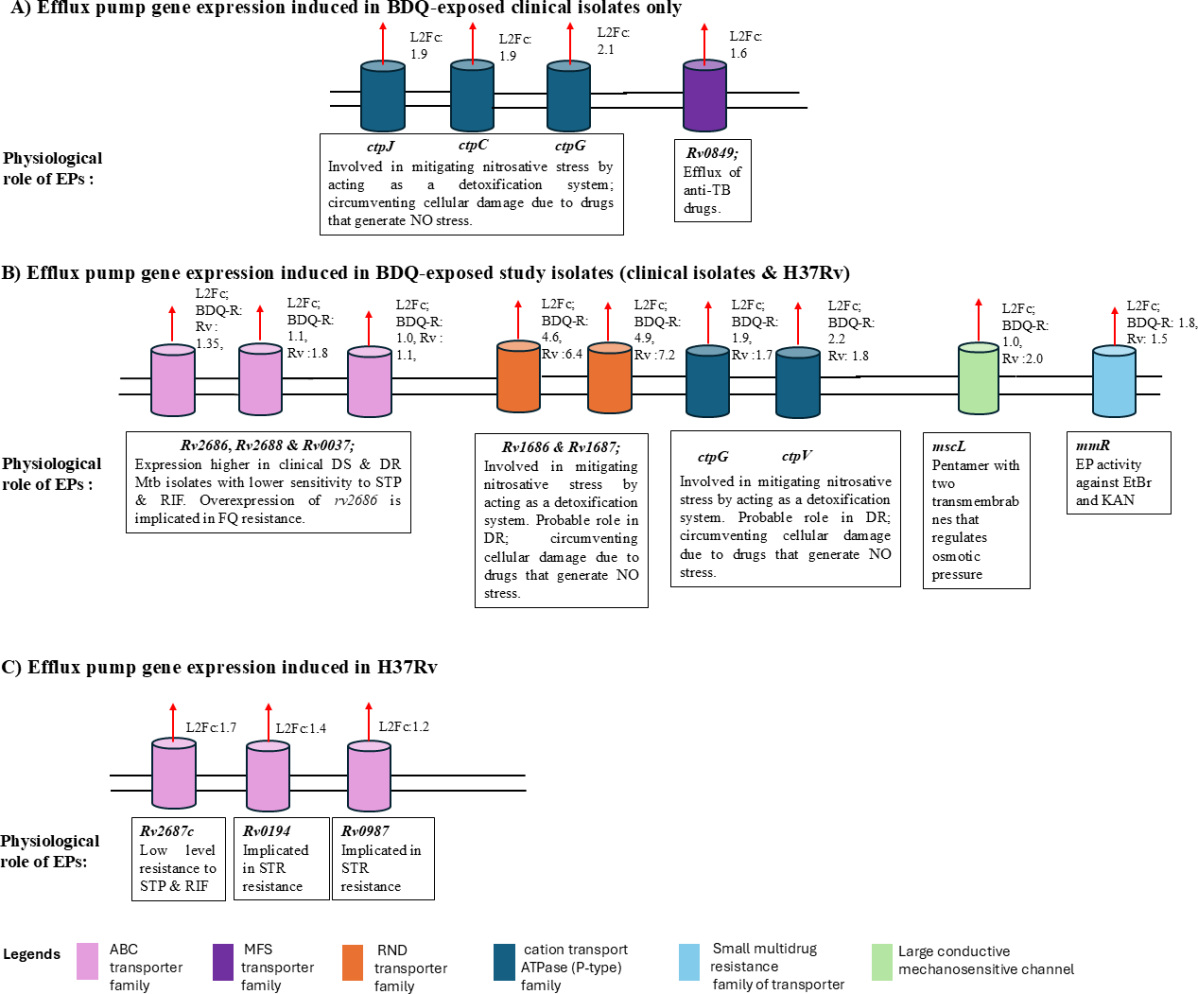
Efflux pump and transporter encoding genes significantly upregulated (Log2FoldChange >1, adjusted *P*-value <0.05) at 72 hours of BDQ exposure (conc. = 3.75 µg/mL) in: (A) BDQ-resistant clinical isolates (*n* = 6), (B) both BDQ-resistant isolates (*n* = 6) and H37Rv, (C) H37Rv only. EP, efflux pump; EtBr, ethidium bromide; KAN, kanamycin; L2Fc, log2Fold Change; DS, drug susceptible; DR, drug resistant; NO, nitrous oxide; TB, tuberculosis; FQ, fluoroquinolone; STR, streptomycin; BDQ, bedaquiline; CFZ, clofazimine; ABC, Adenosine Triphosphate Binding Cassette; MFS, Major Facilitator Superfamily; RND, root nodulating and division.

Several genes encoding transport proteins were studied and noted to have been upregulated in BDQ-exposed study isolates (Table 2; Tables S2 and S3; Fig. 4). For both BDQ-resistant isolates and H37Rv, among upregulated genes, the ABC transporter proteins; *rv1687c* (BDQ-resistant isolates L_2_Fc: 4.95, H37Rv L_2_Fc: 7.16) and *rv1686c* (BDQ-resistant isolates L_2_Fc: 4.59, H37Rv L_2_Fc: 6.39) had the highest differential expression. Other genes identified to be upregulated genes in study isolates included: the large conductance ion mechanosensitive channel (BDQ-resistant isolates; L_2_Fc: 1.13, H37Rv; L_2_Fc: 2.05), ABC transporter gene; *rv1463* (L_2_Fc: BDQ-resistant isolates; L_2_Fc: 1.95, H37Rv; L_2_Fc: 1.78), Major Facilitator Superfamily type transporter genes (*rv0037:* BDQ-resistant isolates L_2_Fc: 1.01, H37Rv L_2_Fc: 1.11, and *rv2688*: BDQ-resistant isolates L_2_Fc: 1.24, H37Rv L_2_Fc:1.75), and *ctpV* (BDQ-resistant isolates L_2_Fc: 2.18, H37Rv L_2_Fc:1.78; Tables S2 and S3). While downregulated genes encoding transporter proteins included: *rv1698* (BDQ-resistant isolates L_2_Fc: 1.45, H37Rv L_2_Fc: −2.31), *rv1857* (BDQ-resistant isolates L_2_Fc: 1.20, H37Rv L_2_Fc: −2.41), *rv2456* (BDQ-resistant isolates L_2_Fc: −1.26, H37Rv L_2_Fc: −1.74), and *rv0403* (BDQ-resistant isolates L_2_Fc: −1.13, H37Rv L_2_Fc: −1.05).

### Gene enrichment analysis

For BDQ-resistant clinical isolates, genes significantly overexpressed after 72 h of BDQ exposure were shown to enrich the following biological processes (FDR < 0.05): response to stress (heat and metal ions), amino acid catabolic processes (including L-arginine metabolic processes, branched chain, proteinogenic amino acids, and pyruvate family amino acids), fatty acid beta-oxidation, cholesterol metabolic process, lipid and fatty acid oxidation, and response to abiotic stimulus (Table S5).

While for H37Rv, genes significantly overexpressed after 72 h of BDQ exposure enriched the following biological processes (FDR < 0.05): response to stress (heat and metal ions), regulation of DNA-templated transcription, regulation of RNA biosynthetic and metabolic processes, regulation of primary metabolic processes, regulation of macromolecule biosynthetic processes, and carbohydrate derivative biosynthetic processes (Table S5).

### Genes differentially regulated under other antimycobacterial drug stress

Differentially upregulated genes in BDQ-exposed clinical isolates were compared with transcriptomic changes reported in Mtb for other antimycobacterials (24, 25, 34, 35, 41–48). Among genes differentially upregulated in BDQ-exposed clinical isolates (*n* = 6), 145 genes were identified to be similarly overexpressed in Mtb for other antimycobacterials (Table S6). These genes were involved in different stress responses, including hypoxia, surface tension, and metal ions. Furthermore, several genes involved in base excision repair, negative regulation of DNA templated transcription, RNA biosynthetic and metabolic processes, and tricarboxylic acid (TCA) cycle were also upregulated (Table S6).

## DISCUSSION

Mtb temporarily tolerates bactericidal activity of BDQ through metabolic rerouting to counteract the energy stress placed by inhibition of its ATP synthase enzyme (29). To further these findings (29, 32), BDQ-induced transcriptomic changes in BDQ-resistant clinical Mtb isolates (*n* = 6) were explored. Among notable findings, a cluster of upregulated genes mapped onto the suf operon from the iron-sulfur cluster biogenesis pathway in both BDQ-exposed H37Rv and clinical isolates. Furthermore, differential expression of several genes involved in amino acid biosynthesis and degradation was shown to be induced in study isolates. These genes were found to significantly enrich biological processes for clinical isolates exposed to BDQ.

Earlier reports (23, 25, 34) suggest that genes belonging to metabolism and respiration and to cell wall and cell processes are the most responsive to antimycobacterial drug pressure. This has been observed in Mtb for RIF (23), INH (25), delamanid (DLM) (34), and BDQ (29, 30, 32). Our data show that the majority of BDQ-induced DEGs in study isolates (H37Rv and BDQ-resistant clinical isolates) belonged to intermediate metabolism and respiration, and cell wall and cell processes support these earlier studies. These findings further suggest that the above functional processes may contribute to establishing tolerance as well as vulnerability to BDQ (29).

In drug-tolerant Mtb, metabolic slowdown has been observed (45, 49). Earlier, (45) reported a decrease in ATP synthase activity and a global decrease in protein synthesis as specific transcriptomic signatures of a drug-tolerant Mtb population (45). Keren et al. 2011 also reported similar findings in Mtb persisters formed after D-cycloserine exposure (49). A perturbed respiratory system, downregulation of ATP and protein syntheses, and upregulation of transcriptional factor *rv0324*, indicated in our study, are consistent with findings from earlier studies exploring BDQ-induced transcriptomics in H37Rv (29, 30, 32, 50). These transcriptional changes are likely to reflect metabolic rerouting to a less energy-intensive phase to balance the energy requirement and production capacity of Mtb under BDQ stress. Furthermore, glycolytic vulnerabilities observed in our study have previously been reported (32) as a collateral arising from inhibition of ATP synthase that further contributes to BDQ’s bactericidal activity.

Broadly, the study findings indicate that under BDQ pressure, metabolic reprogramming is induced. Thus, corroborating earlier observations (51–53) that highlight metabolic rerouting of the CCM as a key strategy to mitigate drug-induced stress in Mtb. Specifically, for the BDQ-exposed study isolates (H37Rv and clinical isolates), an enhanced lipid metabolism was indicated by the upregulation of genes from β-oxidation of fatty acids and cholesterol degradation pathways. Lipid metabolism has previously been implicated in mediating broad drug tolerance in Mtb (54) by favoring increased carbon flux through the glyoxalate shunt (19, 54) and allowing effective and carbon-saving acetyl co-A disposal (53). Furthermore, the upregulation of ICL, noted in the BDQ-resistant study isolates, indicates a shift from the oxidative arm of the TCA cycle to substrate-level phosphorylation (53). Collectively, these metabolic adaptations may play a similar mechanistic role in supporting Mtb’s survival under the energy-stressed state created by BDQ. The shift to the glyoxalate shunt has been reported to mitigate the harmful effect of respiratory radicals arising from TCA cycle-mediated NADH production and electron transport chain activity (53, 55). Based on published data suggesting the role of ICL in facilitating Mtb survival under inhibitory concentrations of several anti-TB drugs, including RIF, INH, and streptomycin (21, 22), metabolic shifts from the oxidative part of the TCA cycle may also reduce respiratory stress due to BDQ. It is, therefore, likely that ICL plays a potentially important role in establishing BDQ tolerance through metabolic re-routing (21, 22). Interestingly, we observed that amino acid biosynthesis and degradation pathways, including arginine metabolism, were enriched in BDQ-exposed clinical isolates. Amino acid metabolism is important for Mtb growth, survival, and pathogenesis within the host macrophage (39, 56, 57). These metabolic pathways serve to both generate energy and alter the host’s immune response (58). Moreover, in the absence of specific glutaminyl or asparaginyl tRNA, indirect tRNA aminoacylation pathway is used by many bacterial species including mycobacteria (57). This pathway has been linked to increased mistranslation and resulting gain-of-function variants in the β subunit of RNA polymerase, facilitating RIF tolerance (59). Additionally, activation of *de novo* arginine biosynthesis is one of the early responses to reactive oxygen species (ROS) mediated killing by INH and vitamin C in Mtb (60). Thus, pathways involving *de novo* biosynthesis of amino acids, specifically arginine, may serve as an alternate source of energy and mitigate BDQ-induced stress. Among genes from amino acid metabolism, *argF* and *argB* have previously been proposed as drug targets (57). Recently, Vilcheze et al. 2025 reported a significant reduction (six log-fold) in colony-forming units of MDR Mtb cultures treated with BDQ and vitamin C (61). Heightened sensitization to BDQ was attributed to increased ROS production by vitamin C rather than ATP depletion (61). Among upregulated metabolic pathways, arginine biosynthesis and *suf* operon from the Fe-S complex were found to be upregulated and proposed to be induced in response to ROS generation (61). *In vivo*, low BDQ concentration in intrapatient niches represents sites where resistant populations can emerge even for effective regimens (62, 63). Given this, the potential of concomitant use of Vitamin C or products leading to ROS production in BDQ-based regimens should be explored in preventing acquired BDQ resistance in clinical settings.

Of note, our analysis showed upregulation of several stress response genes. *σ* factors, including *σ^E^* and *σ^H^*, were indicated to be overexpressed in this study. Induction of these σ factors may be an adaptive strategy used by Mtb to counteract respiratory and growth stress caused by BDQ. For instance, during ATP depletion, *σ^E^*-mediated transcription of the stringent response gene *relA* facilitates persistence in Mtb (64). Furthermore, simultaneous overexpression of *rseA* and *σ^E^*, as observed in our study, is associated with decreased growth rate of Mtb (18, 18). Similarly, *σ^H^* is upregulated during heat, redox, nitrosative, and acid stress within the phagosome (65). Additionally, overexpression of the iron-sulfur cluster and *suf* operons indicates a possible response to nitrosative stress (34, 66, 67) generated under BDQ pressure due to respiratory perturbances. In Mtb, *sufR* from the *suf* operon responds to nitric oxide and regulates Fe–S cluster biogenesis, thus establishing persistence in mice (68). These stress response genes were only upregulated in BDQ-exposed isolates and not expressed by isolates grown in liquid media without BDQ pressure. We, therefore, propose that these stress responses may contribute to BDQ tolerance.

Genes found to be upregulated in BDQ-exposed clinical Mtb isolates from this study were reported to be similarly induced under other antimycobacterial drugs, including INH, DLM, linezolid, capreomycin, ethambutol, ethionamide, and standard drug susceptible (DS) TB therapy (24, 25, 34, 35, 41–46). Genes similarly upregulated under both BDQ and DLM pressure (34) suggest that, irrespective of the primary mode of drug activity, overlapping downstream antimycobacterial processes may be triggered such as respiratory stresses (69). Subsequently, activating common mechanisms to counteract these perturbances. Likewise, genes from our study and reported by others (24, 35, 42, 43) are involved in hypoxia (*groEL2*, *htmA*, *PE_PGRS11*, and *higB*) and metal ions (*mbtB*, *rv2025c*, *mymT*, *htmA*, *ctpV*, *ctpG*, and *cadI*).

These differentially regulated genes present themselves as possible targets for drug designing and as prognostic markers. Shared mechanisms contributing to tolerance among several antimycobacterials may explain the high probability of therapeutic failure occurring during MDR TB treatment (62) and acquired resistance during drug therapy (70, 71).

### Limitations and future directions

In this initial exploratory study, H37Rv was used as a DS standard laboratory strain. In view of our findings, we propose that future studies should also include DS clinical Mtb isolates. This would provide further insights into BDQ-induced transcriptomic changes in Mtb. Furthermore, biological replicates (*n* = 2) for each isolate were used. A greater number of biological replicates and a larger sample size would provide greater inferential confidence in detecting nuanced changes occurring due to drug pressure. As the replicate size for each isolate was low, our study does not factor in isolate-level transcriptomic changes.

As ours was an *in vitro* study, it does not completely replicate the environmental milieu of Mtb isolates. However, it is relevant for identifying transcriptional changes in Mtb in response to BDQ pressure and designing further antibiotic-induced transcriptomic studies. These pathways are highlighted and proposed as potentially being important because of their relevance in Mtb’s survival, as illustrated in previous studies.

Further knock-out and *in* vivo studies are needed to explore the potential of these pathways and genes differentially regulated under BDQ pressure as prognostic markers and drug targets. During drug therapy, transcriptional signatures of metabolic fluxes associated with tolerance can be used as indicators to gage the effectiveness of new or additional treatment regimens. This could be used in tandem with DS testing to flag TB regimens that are moving toward failure or help in the selection of additional drugs for treating prolonged and complex drug-resistant TB.

### Conclusion

Overall, our results suggest that amino acid and Fe-S cluster biosynthesis pathways are differentially expressed in response to ATP depletion due to BDQ stress. Further studies are required to understand the role of these pathways in generating energy and to establish tolerance to BDQ among clinical isolates.

## MATERIALS AND METHODS

A schematic representation of the methodology used in this study is presented in Fig. S1.

### Strain selection

Six BDQ-resistant clinical Mtb isolates (Tables S1, S2, S5, S7, S9, and S10), previously described (72), and H37Rv (ATCC 27294) as a reference isolate were selected. Phenotypic DS profiles of these study isolates are provided in Table S7.

### Culture growth

Frozen vials of selected BDQ-resistant isolates (*n* = 6) and H37Rv were thawed and cultured on Lowenstein-Jensen slants. Mid-log phase growth (not older than three weeks) was used to prepare a 1.0 McFarland suspension for each isolate. This was then transferred to polystyrene Falcon tubes containing 40 mL of 7H9 media supplemented with Oleic Acid-albumin-Dextrose-Catalase. Duplicate cultures were set up for each isolate.

The liquid growth cultures were then incubated at 37°C, 5% CO_2_ with intermittent mixing till mid-log phase of growth was reached at an optical density (OD_600_) of 0.4–0.5 (73). Growth at this point was defined as baseline (*t* = 0 h). BDQ was added to each isolate culture to achieve a final concentration of 3.75 µg/mL, equivalent to 7.5× minimum inhibitory concentration for BDQ-resistant isolates, a level at which mycobacterial ATP levels are reduced without any measurable effect on Mtb viability (30). Control duplicate cultures without any exposure to BDQ were also set up for each isolate.

### RNA extraction

RNA was extracted from Mtb cultures and H37Rv at baseline (*t* = 0 h) and after 72 h from BDQ-exposed and unexposed control conditions. RNA extraction was performed using the TRIzol reagent protocol described by Benjak et al. (74) with modifications. Briefly, TRIzol was added to cell pellets snap-frozen in liquid nitrogen. Cell debris was then removed using chloroform. Nucleic acid precipitation was conducted using 0.1 vol of sodium acetate (pH 5.2) and 0.7 vol of isopropanol at −20°C overnight and centrifuged at x16000 g for 30 min. The precipitated RNA was then washed with ethanol (70%) and resuspended in 96 µL of diethyl pyrocarbonate water to which 12 µL of 10× DNase buffer and 12 µL of 2 U/µL DNase were added. Phenol-chloroform-isoamyl alcohol extraction was performed, and the resulting aqueous phase was then incubated for at least 6 h with sodium acetate (pH 5.2) and isopropanol at −20°C followed by centrifugation to precipitate out RNA. RNA concentration was determined using a Nanodrop spectrophotometer (DeNovix DS-11 Spectrophotometer, USA; Table S8). RNA integrity was determined by running 200 ng/µL of samples on 1% agarose gel.

### RNA seq

Total RNA was sequenced by Macrogen Inc, Korea. RNA integrity was checked with Agilent 2100 Bioanalyzer. Paired-end RNA seq was performed on the NovaSeq6000 platform (Illumina). FastQ files generated were used for downstream analysis.

### Data analysis

A QC30/QC20 > 90% was considered for FastQ files quality (Table S8). Downstream bioinformatics analysis was performed using the protocol by (74) to obtain count files of raw data.

### PCA and distance matrix graph

Count data were analyzed using R studio vr. 4.3.0 and the Bioconductor package DESeq2 version (36). The count files were normalized using variance stabilization transformation and then used for plotting PCA and distance matrix plots as a visual exploration of the data from this study.

### Differential gene expression analysis

DEGs were analyzed using the DESeq2 package (36) for R vr. 4.3.0 (75). The transcriptomic profile was studied by fitting a model that included terms for strain type, condition, and the interaction between strain type and condition. The term strain type included H37Rv categorized as lab_susceptible and clinical isolates that were categorized as clinical_resistant.

For transcriptome analysis of genes differentially expressed in study isolates (H37Rv and BDQ-resistant clinical isolates) after 72 h of growth in liquid media without BDQ, condition included terms: baseline_growth (growth at baseline without BDQ exposure) and 72 h_growth (growth at 72 h without BDQ exposure).

For transcriptomic analysis of genes differentially expressed in study isolates after 72 h of BDQ exposure, the term condition included controls that were categorized as unexposed and BDQ-exposed isolates that were categorized as exposed.

### Transcriptional profiles post 72 h of growth in 7H9 liquid media without BDQ pressure

The transcriptome of the study isolates was profiled after 72 h of growth in liquid media without BDQ. Read counts after 72 h of growth in 7H9 media were compared. Read counts of Mtb isolates and of H37Rv after 72 h of BDQ exposure were compared with those of unexposed control isolates using the contrast function for Wald’s statistics in DESeq2 (36).

### Transcriptional profiles post 72 h of exposure to BDQ (3.75 µg/mL)

Read counts of Mtb isolates and of H37Rv after 72 h of BDQ exposure were compared with those of unexposed control isolates using the contrast function for Wald’s statistics in DESeq2 (36).

Based on observations from the PCA plot that showed S5 to cluster separately from other isolates, a sensitivity analysis was performed. In the sensitivity analysis, S5 was excluded from the overall DESeq analysis. DEGs from the sensitivity analysis were compared with the overall analysis to justify inclusion of S5 with the study isolates (Table S9).

### Thresholds for identifying significantly DEGs

To minimize false positives, an adjusted *P*-value, calculated using Benjamini-Hochberg’s multiple testing correction, was used for all analyses. Furthermore, to identify significantly expressed genes, the threshold values for adjusted *P*-value (*P*-adjusted) were set at <0.05 (76, 77) and for L_2_Fc > |1| (77, 78).

Analysis for DEGs (Wald’s statistics) was performed with H37Rv and BDQ-resistant isolates modeled in the same DESeq2 framework. In the preprocessing step, the lfcShrink function was used to exclude non-zero estimated effects arising due to noise.

### Comparison of differential gene expression of BDQ-exposed clinical isolates with H37Rv

The output analyses obtained for DEG in H37Rv and BDQ-resistant clinical isolates due to BDQ exposure were compared to identify genes common between both as well as those unique to BDQ-resistant clinical isolates. Functional annotation of genes was done using Mycobrowser (37)

### Hierarchical clustering of DEGs

Significantly DEGs were scaled using the *z*-score. Hierarchical clustering was conducted using an agglomerative approach. The top 50 DEGs (25 upregulated and 25 downregulated) were visualized using the pheatmap package (79). The resulting expression image tree was further characterized based on color, where red, white, and blue represented over, under, and equal levels of expression, respectively (26).

### Mapping DEGs onto metabolic pathways

DEGs significantly over- or underexpressed under BDQ stress in BDQ-resistant isolates and H37Rv strain were mapped onto metabolic pathways of Mtb H37Rv using the metabolic viewer tool in BioCyc (38).

### Gene enrichment analysis

The Gene Ontology Database (80, 81) was used to identify biological processes enriched in BDQ-exposed H37Rv and BDQ-resistant clinical isolates. Analysis involved the PANTHER Overrepresentation Test (released 20240807), Fisher’s Exact test, and false discovery rate value (*P* < 0.05).

## Data Availability

The data set supporting the conclusions of this article is available in the BioProject database. The accession number for Fastq files submitted in the BioProject database is PRJNA1120628 (82).

## SUPPLEMENTAL MATERIAL

Supplemental material spectrum.01455-25-s0001.pdf Table S1 to S9; Fig. S1 to S3.

ASM does not own the copyrights to Supplemental Material that may be linked to, or accessed through, an article. The authors have granted ASM a non-exclusive, world-wide license to publish the Supplemental Material files. Please contact the corresponding author directly for reuse.
