# Supplementary material for: Profiling of BDQ-induced transcriptome suggests amino acid metabolism and stress responses as alternate mechanisms contributing to BDQ tolerance in Mycobacterium tuberculosis
Source: Microbiol Spectr. 2025 Dec 16;14(2):e01455-25. doi: 10.1128/spectrum.01455-25 (PMC12889156; doi:10.1128/spectrum.01455-25)
Supplement: Supplemental material — Table S1 to S9; Fig. S1 to S3. [file spectrum.01455-25-s0001.pdf]

Supplementary Table 1. Differentially expressed genes (DEGs) in study isolates after 72 hours of growth (BDQ conc; 0 µg/ml). p-Walds test was performed comparing isolates at t= 72 h (no BDQ exposure) with isolates at t= 0 h.

(I) DEGs in Bedaquiline (BDQ)-resistant clincial isolates after 72 h of growth in liquid media (7H9)

Locus_Tag	Genes	Functional Category	baseMean	L2Fc	lfcSE	stat	pvalue	padj
**Rv2875**	major secreted immunogenic protein Mpt70(mpt70)	cell wall and cell processes	2062.56	1.97	0.42	4.72	2.41E-06	5.08E-05
**Rv2873**	cell surface lipoprotein(mpt83)	cell wall and cell processes	1468.35	1.59	0.35	4.54	5.64E-06	9.68E-05
**Rv3477**	PE family protein PE31(PE31)	PE/PPE	1985.44	1.34	0.27	4.94	7.84E-07	2.39E-05
**Rv3083**	FAD-containing monooxygenase MymA(Rv3083)	intermediary metabolism and respiration	1328.22	1.24	0.33	3.78	1.56E-04	1.25E-03
**Rv1901**	competence damage-inducible protein CinA(cinA)	intermediary metabolism and respiration	4280.00	1.18	0.30	3.87	1.10E-04	9.69E-04
**Rv3478**	PE family protein PPE60(PPE60)	PE/PPE	14496.89	1.14	0.20	5.84	5.35E-09	4.61E-07
**Rv0172**	Mce family protein Mce1D(mce1D)	virulence, detoxification and adaptation	8023.83	1.08	0.21	5.14	2.79E-07	1.12E-05
**Rv0173**	Mce family lipoprotein LprK(lprK)	cell wall and cell processes	4658.10	1.08	0.21	5.11	3.30E-07	1.24E-05
**Rv3395c**	hypothetical protein(Rv3395c)	conserved hypothetical	485.52	1.05	0.14	7.26	3.86E-13	1.53E-10
**Rv0169**	Mce family protein Mce1A(mce1A)	virulence, detoxification and adaptation	5107.91	1.04	0.21	5.06	4.19E-07	1.47E-05
**Rv0171**	Mce family protein Mce1C(mce1C)	virulence, detoxification and adaptation	7721.75	1.01	0.20	5.19	2.14E-07	9.44E-06
**Rv0167**	membrane protein(yrbE1A)	cell wall and cell processes	4512.54	1.01	0.19	5.27	1.34E-07	6.32E-06
**Rv0174**	Mce family protein Mce1F(mce1F)	virulence, detoxification and adaptation	14245.06	1.01	0.15	6.61	3.78E-11	7.34E-09
**Rv1995**	hypothetical protein(Rv1995)	conserved hypothetical	659.59	-1.00	0.19	-5.30	1.18E-07	5.63E-06
**Rv2819c**	CRISPR type III-associated RAMP protein Csm5(Rv2819c)	conserved hypothetical	685.84	-1.01	0.27	-3.70	2.17E-04	1.61E-03
**Rv0141c**	hypothetical protein(Rv0141c)	conserved hypothetical	3361.23	-1.02	0.22	-4.71	2.47E-06	5.18E-05
**Rv2618**	hypothetical protein(Rv2618)	conserved hypothetical	3367.98	-1.02	0.24	-4.21	2.57E-05	3.18E-04
**Rv2373c**	chaperone protein DnaJ(dnaJ2)	virulence, detoxification and adaptation	20423.25	-1.02	0.24	-4.21	2.58E-05	3.18E-04
**Rv1463**	ABC transporter ATP-binding protein(Rv1463)	cell wall and cell processes	7592.89	-1.03	0.21	-4.92	8.45E-07	2.52E-05
**Rv0246**	integral membrane protein(Rv0246)	cell wall and cell processes	2928.68	-1.04	0.18	-5.79	7.18E-09	5.81E-07
**Rv0248c**	succinate dehydrogenase flavoprotein subunit(Rv0248c)	intermediary metabolism and respiration	12899.82	-1.05	0.20	-5.36	8.31E-08	4.40E-06
**Rv2743c**	hypothetical protein(Rv2743c)	conserved hypothetical	11707.67	-1.05	0.25	-4.23	2.34E-05	3.00E-04
**Rv1466**	hypothetical protein(Rv1466)	conserved hypothetical	2590.71	-1.06	0.19	-5.49	4.08E-08	2.51E-06
**Rv2386c**	salicylate synthase(mbtI)	lipid metabolism	1940.50	-1.06	0.23	-4.69	2.77E-06	5.60E-05
**Rv1991A**	antitoxin MazE6(mazE6)	virulence, detoxification and adaptation	777.83	-1.07	0.19	-5.58	2.35E-08	1.56E-06

**Rv0847**	lipoprotein LpqS(lpqS)	cell wall and cell processes	11120.98	-1.08	0.18	-6.03	1.60E-09	1.81E-07
**Rv3054c**	hypothetical protein(Rv3054c)	conserved hypothetical	13037.80	-1.08	0.31	-3.50	4.63E-04	2.92E-03
**Rv2621c**	transcriptional regulator(Rv2621c)	regulatory protein	8896.07	-1.08	0.31	-3.54	3.95E-04	2.55E-03
**Rv0350**	chaperone protein DnaK(dnaK)	virulence, detoxification and adaptation	100300.95	-1.09	0.20	-5.33	9.95E-08	5.06E-06
**Rv1130**	2-methylcitrate dehydratase(prpD)	intermediary metabolism and respiration	8493.05	-1.09	0.34	-3.19	1.41E-03	6.96E-03
**Rv2744c**	hypothetical protein(35kd_ag)	conserved hypothetical	27460.04	-1.09	0.23	-4.69	2.67E-06	5.48E-05
**Rv1460**	transcriptional regulator(Rv1460)	regulatory protein	8118.06	-1.09	0.21	-5.08	3.69E-07	1.37E-05
**Rv2107**	PE family protein PE22(PE22)	PE/PPE	167.79	-1.09	0.22	-4.94	7.99E-07	2.42E-05
**Rv1465**	nitrogen fixation related protein(Rv1465)	intermediary metabolism and respiration	3683.11	-1.09	0.22	-4.96	6.98E-07	2.20E-05
**Rv0763c**	ferredoxin(Rv0763c)	intermediary metabolism and respiration	350.24	-1.10	0.30	-3.65	2.62E-04	1.84E-03
**Rv0351**	stress response protein GrpE(grpE)	virulence, detoxification and adaptation	36934.21	-1.12	0.23	-4.85	1.25E-06	3.31E-05
**Rv0565c**	monooxygenase(Rv0565c)	intermediary metabolism and respiration	1500.52	-1.13	0.16	-6.87	6.35E-12	1.57E-09
**Rv3081**	hypothetical protein(Rv3081)	conserved hypothetical	2119.38	-1.13	0.33	-3.42	6.18E-04	3.68E-03
**Rv0762c**	hypothetical protein(Rv0762c)	conserved hypothetical	2133.24	-1.14	0.30	-3.72	1.96E-04	1.49E-03
**Rv0848**	cysteine synthase CysK(cysK2)	intermediary metabolism and respiration	14536.07	-1.14	0.22	-5.32	1.05E-07	5.06E-06
**Rv2620c**	transmembrane protein(Rv2620c)	cell wall and cell processes	2700.99	-1.14	0.28	-4.04	5.28E-05	5.46E-04
**Rv0846c**	oxidase(Rv0846c)	intermediary metabolism and respiration	5426.64	-1.16	0.19	-6.21	5.37E-10	6.45E-08
**Rv2642**	ArsR family transcriptional regulator(Rv2642)	regulatory protein	9907.80	-1.16	0.24	-4.79	1.67E-06	4.07E-05
**Rv1461**	hypothetical protein(Rv1461)	conserved hypothetical	37120.77	-1.16	0.18	-6.29	3.08E-10	3.95E-08
**Rv0564c**	glycerol-3-phosphate dehydrogenase(gpdA1)	lipid metabolism	6615.82	-1.17	0.19	-6.32	2.69E-10	3.56E-08
**Rv3160c**	TetR family transcriptional regulator(Rv3160c)	regulatory protein	3870.00	-1.17	0.25	-4.77	1.89E-06	4.27E-05
**Rv0251c**	heat shock protein(hsp)	virulence, detoxification and adaptation	40335.06	-1.17	0.29	-4.11	3.91E-05	4.37E-04
**Rv2619c**	hypothetical protein(Rv2619c)	conserved hypothetical	2043.46	-1.23	0.25	-4.82	1.41E-06	3.61E-05
**Rv0190**	hypothetical protein(Rv0190)	conserved hypothetical	4715.73	-1.23	0.17	-7.42	1.19E-13	5.88E-11
**Rv1057**	hypothetical protein(Rv1057)	conserved hypothetical	7317.64	-1.24	0.24	-5.22	1.77E-07	8.15E-06
**Rv2643**	arsenic-transport integral membrane protein ArsC(arsC)	cell wall and cell processes	10933.96	-1.25	0.26	-4.90	9.77E-07	2.75E-05
**Rv2639c**	integral membrane protein(Rv2639c)	cell wall and cell processes	2098.92	-1.25	0.19	-6.49	8.57E-11	1.55E-08
**Rv3854c**	monooxygenase EthA(ethA)	intermediary metabolism and respiration	19532.57	-1.26	0.27	-4.66	3.13E-06	6.23E-05
**Rv1072**	transmembrane protein(Rv1072)	cell wall and cell processes	80040.70	-1.30	0.20	-6.37	1.83E-10	2.50E-08
**Rv2745c**	transcriptional regulator ClgR(clgR)	regulatory protein	14465.53	-1.31	0.25	-5.20	2.02E-07	9.04E-06

**Rv1285**	sulfate adenylyltransferase subunit 2(cysD)	intermediary metabolism and respiration	17623.60	-1.31	0.26	-5.10	3.31E-07	1.24E-05
**Rv0353**	heat shock protein transcriptional repressor HspR(hspR)	virulence, detoxification and adaptation	5928.74	-1.33	0.21	-6.42	1.34E-10	2.12E-08
**Rv2961**	transposase(Rv2961)	insertion seq and phages	325.33	-1.34	0.19	-6.99	2.82E-12	8.61E-10
**Rv0851c**	short-chain type dehydrogenase/reductase(Rv0851c)	intermediary metabolism and respiration	1714.35	-1.34	0.23	-5.83	5.58E-09	4.71E-07
**Rv1073**	hypothetical protein(Rv1073)	conserved hypothetical	22212.67	-1.34	0.17	-7.71	1.28E-14	9.85E-12
**Rv1129c**	transcriptional regulator(Rv1129c)	regulatory protein	2438.72	-1.35	0.31	-4.43	9.35E-06	1.46E-04
**Rv2053c**	transmembrane protein FxsA(fxsA)	cell wall and cell processes	11887.31	-1.36	0.26	-5.14	2.77E-07	1.12E-05
**Rv2962c**	PGL/p-HBAD biosynthesis rhamnosyltransferase(Rv2962c)	intermediary metabolism and respiration	2703.60	-1.39	0.21	-6.74	1.57E-11	3.47E-09
**Rv2710**	RNA polymerase sigma factor SigB(sigB)	information pathways	84070.26	-1.39	0.22	-6.28	3.36E-10	4.17E-08
**Rv0354c**	PPE family protein PPE7(PPE7)	PE/PPE	5172.70	-1.39	0.18	-7.69	1.49E-14	9.85E-12
**Rv2122c**	phosphoribosyl-ATP pyrophosphatase(hisE)	intermediary metabolism and respiration	150.86	-1.40	0.33	-4.22	2.42E-05	3.07E-04
**Rv0849**	MFS-type transporter(Rv0849)	cell wall and cell processes	5690.69	-1.41	0.28	-5.05	4.38E-07	1.51E-05
**Rv1288**	hypothetical protein(Rv1288)	conserved hypothetical	3004.92	-1.46	0.24	-6.02	1.79E-09	1.98E-07
**Rv0563**	protease HtpX(htpX)	virulence, detoxification and adaptation	13000.92	-1.46	0.24	-6.18	6.35E-10	7.40E-08
**Rv2964**	formyltetrahydrofolate deformylase(purU)	intermediary metabolism and respiration	1416.53	-1.48	0.20	-7.43	1.12E-13	5.88E-11
**Rv0249c**	succinate dehydrogenase membrane anchor subunit(Rv0249c)	intermediary metabolism and respiration	9045.37	-1.50	0.22	-6.94	3.84E-12	1.01E-09
**Rv1529**	fatty-acid--CoA ligase FadD24(fadD24)	lipid metabolism	2199.92	-1.52	0.21	-7.31	2.75E-13	1.21E-10
**Rv1286**	adenylyl-sulfate kinase(Rv1286)	intermediary metabolism and respiration	26248.01	-1.52	0.26	-5.93	2.94E-09	2.92E-07
**Rv1287**	HTH-type transcriptional regulator(Rv1287)	regulatory protein	4658.55	-1.54	0.21	-7.16	8.32E-13	2.99E-10
**Rv2640c**	ArsR family transcriptional regulator(Rv2640c)	regulatory protein	4619.17	-1.57	0.20	-7.75	9.52E-15	9.85E-12
**Rv0250c**	hypothetical protein(Rv0250c)	conserved hypothetical	10287.70	-1.65	0.21	-7.89	2.91E-15	5.77E-12
**Rv2963**	integral membrane protein(Rv2963)	cell wall and cell processes	7518.64	-1.84	0.24	-7.73	1.07E-14	9.85E-12

(II) DEGs in H37Rv after 72 h of growth in liquid media (7H9)

**Locus_tags**	**Genes**	**Functional Category**	**baseMean**	**L2Fc**	**lfcSE**	**stat**	**pvalue**	**padj**
Rv2077c	transmembrane protein(Rv2077c)	cell wall and cell wall processes	1646.42	6.06	0.28	21.82	1.52E-105	6.05E-102
Rv3407	antitoxin VapB47(vapB47)	virulence, detoxification, and adaptation	1410.33	3.75	0.39	9.64	5.41E-22	7.15E-19
Rv3408	ribonuclease VapC47(vapC47)	virulence, detoxification, and adaptation	2590.28	3.59	0.28	12.83	1.09E-37	2.15E-34
Rv3727	oxidoreductase(Rv3727)	intermediary metabolism and respiration	2215.51	3.50	0.61	5.74	9.34E-09	1.61E-06

Rv2159c	hypothetical protein(Rv2159c)	conserved hypotheticals	3046.89	3.39	0.50	6.78	1.19E-11	3.37E-09
Rv3728	membrane protein(Rv3728)	cell wall and cell wall processes	4446.62	2.82	0.66	4.27	1.95E-05	9.69E-04
Rv2076c	hypothetical protein(Rv2076c)	conserved hypotheticals	802.54	2.64	0.56	4.74	2.14E-06	1.74E-04
Rv2348c	hypothetical protein(Rv2348c)	conserved hypotheticals	1928.17	2.60	0.55	4.73	2.23E-06	1.77E-04
Rv1806	PE family protein PE20(PE20)	PE/PPE	302.42	2.50	0.63	3.99	6.58E-05	2.68E-03
Rv2347c	ESAT-6 like protein EsxP(esxP)	cell wall and cell wall processes	4229.10	2.50	0.57	4.40	1.06E-05	5.84E-04
Rv3750c	excisionase(Rv3750c)	insertion seqs and phages	900.67	2.43	0.43	5.61	2.05E-08	3.12E-06
Rv3143	response regulator(Rv3143)	regulatory proteins	473.55	2.41	0.26	9.17	4.58E-20	3.64E-17
Rv2161c	hypothetical protein(Rv2161c)	conserved hypotheticals	7519.28	2.37	0.47	5.05	4.51E-07	4.97E-05
Rv0048c	membrane protein(Rv0048c)	cell wall and cell wall processes	829.36	2.35	0.25	9.48	2.58E-21	2.55E-18
Rv2661c	hypothetical protein(Rv2661c)	conserved hypotheticals	2090.84	2.24	0.75	2.99	2.76E-03	4.56E-02
Rv2478c	hypothetical protein(Rv2478c)	conserved hypotheticals	132.21	2.17	0.67	3.22	1.26E-03	2.69E-02
Rv2600	integral membrane protein(Rv2600)	cell wall and cell wall processes	329.46	2.16	0.32	6.67	2.59E-11	6.84E-09
Rv3615c	ESX-1 secretion-associated protein EspC(espC)	cell wall and cell wall processes	3967.27	2.06	0.69	2.98	2.86E-03	4.62E-02
Rv3749c	hypothetical protein(Rv3749c)	conserved hypotheticals	656.09	2.05	0.23	8.77	1.84E-18	1.22E-15
Rv0691A	mycofactocin precursor(Rv0691A)	regulatory proteins	335.12	2.03	0.68	2.99	2.75E-03	4.56E-02
Rv1805c	hypothetical protein(Rv1805c)	conserved hypotheticals	1409.17	1.99	0.33	6.10	1.03E-09	2.04E-07
Rv1102c	mRNA interferase MazF3(mazF3)	virulence, detoxification, and adaptation	1055.24	1.99	0.29	6.81	9.96E-12	3.29E-09
Rv3409c	cholesterol oxidase(choD)	lipid metabolism	10288.62	1.97	0.29	6.86	6.85E-12	2.47E-09
Rv0842	integral membrane protein(Rv0842)	cell wall and cell wall processes	5555.55	1.96	0.52	3.76	1.68E-04	5.92E-03
Rv2158c	UDP-N-acetylmuramoylalanyl-D-glutamate--2,6-diaminopimelate ligase(murE)	cell wall and cell wall processes	3838.38	1.93	0.41	4.70	2.64E-06	1.97E-04
Rv2346c	ESAT-6 like protein EsxO(esxO)	cell wall and cell wall processes	3892.93	1.93	0.52	3.70	2.17E-04	7.49E-03
Rv0166	fatty-acid--CoA ligase FadD5(fadD5)	lipid metabolism	4183.45	1.89	0.53	3.59	3.36E-04	1.01E-02
Rv0654	carotenoid cleavage oxygenase(Rv0654)	intermediary metabolism and respiration	13765.57	1.89	0.63	2.99	2.76E-03	4.56E-02
Rv1682	hypothetical protein(Rv1682)	conserved hypotheticals	1425.94	1.86	0.40	4.60	4.16E-06	2.80E-04
Rv0694	mycofactocin system heme/flavin oxidoreductase MftD(Rv0694)	intermediary metabolism and respiration	10160.18	1.84	0.56	3.26	1.13E-03	2.50E-02
Rv2157c	UDP-N-acetylmuramoyl-tripeptide--D-alanyl-D-alanine ligase(murF)	conserved hypotheticals	1571.43	1.82	0.40	4.51	6.40E-06	3.91E-04

Rv2156c	phospho-N-acetylmuramoyl-pentappeptidetransferase(murX)	cell wall and cell wall processes	1434.82	1.81	0.38	4.82	1.41E-06	1.27E-04
Rv1103c	antitoxin MazE3(mazE3)	virulence, detoxification, and adaptation	731.68	1.79	0.29	6.13	8.91E-10	1.86E-07
Rv1808	PPE family protein PPE32(PPE32)	PE/PPE	1322.05	1.73	0.50	3.46	5.31E-04	1.38E-02
Rv1553	fumarate reductase iron-sulfur subunit(frdB)	conserved hypotheticals	109.15	1.73	0.48	3.60	3.16E-04	9.72E-03
Rv1524	glycosyltransferase(Rv1524)	intermediary metabolism and respiration	637.54	1.70	0.44	3.85	1.18E-04	4.43E-03
Rv2664	hypothetical protein(Rv2664)	conserved hypotheticals	1330.36	1.65	0.54	3.07	2.13E-03	3.83E-02
Rv0342	isoniazid inductible protein IniA(iniA)	cell wall and cell wall processes	3729.53	1.61	0.33	4.88	1.05E-06	9.91E-05
Rv1517	hypothetical protein(Rv1517)	cell wall and cell wall processes	264.70	1.59	0.44	3.59	3.26E-04	9.96E-03
Rv1552	fumarate reductase flavoprotein subunit(frdA)	intermediary respiration and metabolism	367.29	1.57	0.43	3.62	2.98E-04	9.52E-03
Rv0797	hypothetical protein(Rv2664)	insertion seqs and phages	161.92	1.57	0.39	4.05	5.08E-05	2.19E-03
Rv3346c	transmembrane protein(Rv3346c)	cell wall and cell wall processes	83.06	1.55	0.32	4.78	1.72E-06	1.48E-04
Rv1872c	L-lactate dehydrogenase(lldD2)	intermediary metabolism and respiration	61542.90	1.55	0.30	5.15	2.62E-07	3.05E-05
Rv0022c	transcriptional regulator WhiB5(whiB5)	regulatory proteins	60.18	1.54	0.48	3.18	1.46E-03	2.97E-02
Rv2779c	Lrp/AsnC family transcriptional regulator(Rv2779c)	conserved hypotheticals	481.24	1.54	0.40	3.90	9.70E-05	3.70E-03
Rv1884c	resuscitation-promoting factor RpfC(rpfC)	cell wall and cell wall processes	6764.61	1.54	0.51	3.04	2.38E-03	4.13E-02
Rv2667	ATP-dependent protease ATP-binding subunit ClpC(clpC2)	intermediary metabolism and respiration	445.80	1.50	0.50	3.00	2.69E-03	4.53E-02
Rv1870c	hypothetical protein(Rv1870c)	conserved hypotheticals	9407.96	1.50	0.39	3.87	1.11E-04	4.18E-03
Rv3347c	PPE family protein PPE55(PPE55)	PE/PPE	10139.77	1.49	0.19	7.78	7.43E-15	3.68E-12
Rv1101c	hypothetical protein(Rv1101c)	conserved hypotheticals	2000.74	1.49	0.22	6.79	1.09E-11	3.33E-09
Rv3558	PPE family protein PPE64(PPE64)	conserved hypotheticals	614.93	1.44	0.25	5.64	1.73E-08	2.74E-06
Rv1954c	hypothetical protein(Rv1954c)	conserved hypotheticals	6974.22	1.43	0.44	3.25	1.16E-03	2.52E-02
Rv2990c	hypothetical protein(Rv2990c)	conserved hypotheticals	8680.11	1.40	0.45	3.13	1.72E-03	3.36E-02
Rv0880	HTH-type transcriptional regulator(Rv0880)	regulatory proteins	241.67	1.38	0.33	4.13	3.68E-05	1.70E-03
Rv0655	ABC transporter ATP-binding protein(mkl)	cell wall and cell wall processes	25764.28	1.34	0.37	3.63	2.85E-04	9.29E-03
Rv3746c	PE family protein PE34(PE34)	PE/PPE	270.64	1.34	0.44	3.06	2.21E-03	3.90E-02

Rv0118c	oxalyl-CoA decarboxylase OxcA(oxcA)	intermediary respiration and metabolism	506.32	1.33	0.30	4.48	7.64E-06	4.45E-04
Rv1982A	antitoxin VapB36(vapB36)	virulence, detoxification, and adaptation	946.78	1.33	0.33	3.98	6.84E-05	2.74E-03
Rv1803c	PE-PGRS family protein PE_PGRS32(PE_PGRS32)	conserved hypotheticals	1016.99	1.32	0.33	4.05	5.18E-05	2.21E-03
Rv3096	hypothetical protein(Rv3096)	conserved hypotheticals	2218.45	1.32	0.32	4.09	4.23E-05	1.90E-03
Rv1871c	hypothetical protein(Rv1871c)	conserved hypotheticals	16846.24	1.31	0.28	4.75	2.02E-06	1.67E-04
Rv1026	hypothetical protein(Rv1026)	conserved hypotheticals	733.32	1.31	0.33	4.00	6.36E-05	2.63E-03
Rv1251c	antitoxin VapB36(vapB36)	conserved hypotheticals	1134.86	1.28	0.41	3.09	2.01E-03	3.66E-02
Rv1555	fumarate reductase membrane anchor subunit(frdD)	intermediary metabolism and respiration	115.83	1.25	0.36	3.43	6.02E-04	1.52E-02
Rv1040c	PE family protein PE8(PE8)	PE/PPE	736.90	1.24	0.38	3.26	1.12E-03	2.50E-02
Rv3095	HTH-type transcriptional regulator(Rv3095)	regulatory proteins	2390.92	1.23	0.38	3.25	1.14E-03	2.50E-02
Rv1397c	ribonuclease VapC10(vapC10)	virulence, detoxification, and adaptation	18115.85	1.18	0.35	3.32	9.11E-04	2.14E-02
Rv2386a	ribonuclease VapC10(vapC10)	lipid metabolism	960.83	1.18	0.31	3.76	1.69E-04	5.92E-03
Rv3052c	NrdI protein(nrdI)	information pathways	17987.80	1.12	0.36	3.09	2.02E-03	3.66E-02
Rv0442c	PPE family protein PPE10(PPE10)	PE/PPE	2483.06	1.10	0.19	5.83	5.56E-09	1.00E-06
Rv1403c	methyltransferase(Rv1403c)	intermediary metabolism and respiration	914.41	1.08	0.30	3.63	2.81E-04	9.29E-03
Rv1982c	ribonuclease VapC36(vapC36)	virulence, detoxification, and adaptation	1296.72	1.08	0.31	3.48	4.94E-04	1.31E-02
Rv1941	short-chain type dehydrogenase/reductase(Rv1941)	intermediary metabolism and respiration	3528.76	1.08	0.25	4.31	1.64E-05	8.79E-04
Rv0786c	hypothetical protein(Rv0786c)	conserved hypotheticals	232.42	1.06	0.25	4.21	2.59E-05	1.24E-03
Rv0109	PE-PGRS family protein PE_PGRS1(PE_PGRS1)	PE/PPE	520.49	1.04	0.30	3.45	5.68E-04	1.45E-02
Rv3908	mutator protein MutT(mutT4)	information pathways	1291.88	1.04	0.29	3.56	3.69E-04	1.05E-02
Rv1816	HTH-type transcriptional regulator(Rv1816)	regulatory proteins	2581.55	1.03	0.26	3.94	8.00E-05	3.11E-03
Rv3848	transmembrane protein(Rv3848)	cell wall and cell wall processes	1735.80	1.01	0.24	4.30	1.71E-05	8.90E-04
Rv1778c	hypothetical protein(Rv1778c)	conserved hypotheticals	942.45	-1.00	0.28	-3.55	3.86E-04	1.08E-02
Rv3396c	GMP synthase(guaA)	intermediary metabolism and respiration	3543.68	-1.01	0.26	-3.81	1.42E-04	5.11E-03
Rv1975	hypothetical protein(Rv1975)	conserved hypotheticals	610.81	-1.01	0.32	-3.11	1.87E-03	3.46E-02
Rv1911c	lipoprotein LppC(lppC)	cell wall and cell wall processes	2074.44	-1.01	0.20	-5.16	2.50E-07	3.00E-05

Rv2916c	signal recognition particle protein(ffh)	cell wall and cell wall processes	3194.93	-1.02	0.24	-4.28	1.86E-05	9.55E-04
Rv3778c	aminotransferase(Rv3778c)	intermediary metabolism and respiration	2661.51	-1.06	0.23	-4.63	3.59E-06	2.50E-04
Rv1915	isocitrate lyase AceAa(aceAa)	intermediary metabolism and respiration	3427.01	-1.07	0.31	-3.48	5.09E-04	1.33E-02
Rv3400	hydrolase(Rv3400)	intermediary metabolism and respiration	1829.34	-1.08	0.22	-4.99	6.15E-07	6.25E-05
Rv2974c	hypothetical protein(Rv2974c)	conserved hypotheticals	2106.29	-1.09	0.34	-3.18	1.50E-03	3.01E-02
Rv1121	glucose-6-phosphate 1-dehydrogenase(zwf1)	intermediary metabolism and respiration	4806.56	-1.09	0.33	-3.36	7.88E-04	1.91E-02
Rv2973c	ATP-dependent DNA helicase RecG(recG)	information pathways	2453.30	-1.10	0.30	-3.67	2.40E-04	8.19E-03
Rv3472	hypothetical protein(Rv3472)	conserved hypotheticals	358.54	-1.10	0.26	-4.26	2.03E-05	9.96E-04
Rv1751	oxidoreductase(Rv1751)	intermediary metabolism and respiration	4343.96	-1.12	0.35	-3.20	1.40E-03	2.89E-02
Rv2041c	sugar ABC transporter substrate-binding lipoprotein(Rv2041c)	cell wall and cell wall processes	1117.02	-1.13	0.35	-3.20	1.36E-03	2.85E-02
Rv1350	3-oxoacyl-ACP reductase FabG(fabG2)	lipid metabolism	481.30	-1.13	0.28	-4.03	5.48E-05	2.31E-03
Rv3498c	Mce family protein Mce4B(mce4B)	virulence, detoxification, and adaptation	1281.46	-1.15	0.38	-3.06	2.20E-03	3.90E-02
Rv0469	mycolic acid synthase UmaA(umaA)	lipid metabolism	2657.30	-1.16	0.37	-3.19	1.43E-03	2.92E-02
Rv2121c	ATP phosphoribosyltransferase(hisG)	intermediary metabolism and respiration	365.32	-1.17	0.35	-3.31	9.48E-04	2.20E-02
Rv3803c	MPT51/MPB51 antigen(fbpD)	lipid metabolism	6469.94	-1.20	0.31	-3.83	1.28E-04	4.76E-03
Rv1372	alpha-pyrone synthesis polyketide synthase-like protein(Rv1372)	conserved hypotheticals	759.60	-1.22	0.26	-4.68	2.87E-06	2.03E-04
Rv1491c	TVP38/TMEM64 family membrane protein(Rv1491c)	cell wall and cell wall processes	3953.59	-1.24	0.33	-3.81	1.41E-04	5.11E-03
Rv1058	fatty-acid--CoA ligase FadD14(fadD14)	lipid metabolism	1647.09	-1.25	0.39	-3.22	1.27E-03	2.70E-02
Rv0427c	exodeoxyribonuclease III protein XthA(xthA)	information pathways	836.80	-1.25	0.31	-4.06	4.91E-05	2.14E-03
Rv3878	ESX-1 secretion-associated protein EspJ(espJ)	cell wall and cell wall processes	7684.20	-1.26	0.42	-3.03	2.44E-03	4.19E-02
Rv3485c	short-chain type dehydrogenase/reductase(Rv3485c)	intermediary metabolism and respiration	1648.24	-1.26	0.34	-3.73	1.88E-04	6.55E-03
Rv1188	proline dehydrogenase(Rv1188)	intermediary metabolism and respiration	775.21	-1.28	0.41	-3.08	2.05E-03	3.70E-02
Rv3765c	two component transcriptional regulator TcrX(tcrX)	regulatory protein	668.06	-1.29	0.41	-3.11	1.88E-03	3.46E-02
Rv2042c	hypothetical protein(Rv2042c)	conserved hypotheticals	1652.53	-1.29	0.36	-3.57	3.58E-04	1.05E-02

Rv0552	hypothetical protein(Rv0552)	conserved hypotheticals	1541.61	-1.30	0.37	-3.50	4.73E-04	1.27E-02
Rv1140	integral membrane protein(Rv1140)	cell wall and cell wall processes	1767.12	-1.32	0.31	-4.27	1.94E-05	9.69E-04
Rv1639c	hypothetical protein(Rv1639c)	conserved hypotheticals	3845.92	-1.34	0.43	-3.12	1.81E-03	3.42E-02
Rv1691	hypothetical protein(Rv1691)	conserved hypotheticals	573.97	-1.35	0.28	-4.79	1.63E-06	1.44E-04
Rv1916	isocitrate lyase AceAb(aceAb)	intermediary metabolism and respiration	2573.87	-1.37	0.30	-4.62	3.87E-06	2.65E-04
Rv3082c	HTH-type transcriptional regulator VirS(virS)	virulence, detoxification, and adaptation	477.61	-1.37	0.42	-3.30	9.73E-04	2.24E-02
Rv1914c	hypothetical protein(Rv1914c)	conserved hypotheticals	229.25	-1.38	0.38	-3.59	3.31E-04	1.00E-02
Rv0468	3-hydroxybutyryl-CoA dehydrogenase(fadB2)	lipid metabolism	2077.20	-1.39	0.38	-3.60	3.13E-04	9.72E-03
Rv2277c	glycerolphosphodiesterase(Rv2277c)	intermediary metabolism and respiration	476.04	-1.39	0.39	-3.57	3.63E-04	1.05E-02
Rv0211	phosphoenolpyruvate carboxykinase(pckA)	intermediary metabolism and respiration	14620.63	-1.40	0.31	-4.52	6.11E-06	3.78E-04
Rv3812	PE-PGRS family protein PE_PGRS62(PE_PGRS62)	PE/PPE	2079.43	-1.41	0.42	-3.37	7.61E-04	1.85E-02
Rv3685c	cytochrome P450 Cyp137(cyp137)	intermediary metabolism and respiration	1863.07	-1.41	0.34	-4.20	2.72E-05	1.29E-03
Rv3486	hypothetical protein(Rv3486)	conserved hypotheticals	1028.43	-1.41	0.44	-3.20	1.38E-03	2.87E-02
Rv1976c	hypothetical protein(Rv1976c)	conserved hypotheticals	1210.36	-1.41	0.32	-4.45	8.74E-06	5.02E-04
Rv2063A	mRNA interferase MazF7(mazF7)	virulence, detoxification, and adaptation	312.21	-1.42	0.39	-3.66	2.55E-04	8.58E-03
Rv2872	ribonuclease VapC43(vapC43)	virulence, detoxification, and adaptation	428.39	-1.42	0.45	-3.12	1.80E-03	3.42E-02
Rv0592	Mce family protein Mce2D(mce2D)	virulence, detoxification, and adaptation	735.69	-1.43	0.40	-3.58	3.46E-04	1.03E-02
Rv3499c	Mce family protein Mce4A(mce4A)	virulence, detoxification, and adaptation	1399.23	-1.49	0.45	-3.29	9.90E-04	2.26E-02
Rv0466	hypothetical protein(Rv0466)	conserved hypotheticals	2239.60	-1.49	0.41	-3.67	2.42E-04	8.21E-03
Rv1873	hypothetical protein(Rv1873)	conserved hypotheticals	380.32	-1.53	0.44	-3.51	4.55E-04	1.24E-02
Rv0461	transmembrane protein(Rv0461)	cell wall and cell wall processes	1162.72	-1.53	0.44	-3.44	5.73E-04	1.46E-02
Rv3500c	integral membrane protein(yrbE4B)	virulence, detoxification, and adaptation	642.06	-1.57	0.44	-3.60	3.15E-04	9.72E-03
Rv0892	monooxygenase(Rv0892)	intermediary metabolism and respiration	1259.19	-1.60	0.52	-3.10	1.92E-03	3.52E-02
Rv2275	cyclo(L-tyrosyl-L-tyrosyl) synthase(Rv2275)	conserved hypotheticals	611.66	-1.60	0.50	-3.17	1.51E-03	3.01E-02
Rv3862c	transcriptional regulator WhiB6(whiB6)	regulatory protein	1046.48	-1.62	0.46	-3.56	3.72E-04	1.05E-02
Rv0784	hypothetical protein(Rv0784)	conserved hypotheticals	713.20	-1.63	0.46	-3.51	4.41E-04	1.21E-02
Rv0111	acyltransferase(Rv0111)	intermediary metabolism and respiration	3188.74	-1.65	0.43	-3.81	1.41E-04	5.11E-03

Rv0162c	zinc-type alcohol dehydrogenase subunit E(adhE1)	intermediary metabolism and respiration	901.54	-1.65	0.46	-3.63	2.86E-04	9.29E-03
Rv0047c	hypothetical protein(Rv0047c)	conserved hypotheticals	5829.84	-1.68	0.52	-3.22	1.26E-03	2.69E-02
Rv1687c	ABC transporter ATP-binding protein(Rv1687c)	cell wall and cell wall processes	351.64	-1.74	0.38	-4.52	6.10E-06	3.78E-04
Rv1677	lipoprotein DsbF(dsbF)	cell wall and cell wall processes	2383.83	-1.74	0.32	-5.44	5.19E-08	7.10E-06
Rv3479	transmembrane protein(Rv3479)	cell wall and cell wall processes	5610.74	-1.75	0.41	-4.22	2.46E-05	1.19E-03
Rv1219c	transcriptional regulator(Rv1219c)	regulatory protein	726.04	-1.76	0.45	-3.95	7.97E-05	3.11E-03
Rv1676	hypothetical protein(Rv1676)	conserved hypotheticals	2442.19	-1.77	0.32	-5.52	3.45E-08	5.06E-06
Rv2672	protease(Rv2672)	intermediary metabolism and respiration	2729.55	-1.78	0.36	-4.90	9.55E-07	9.24E-05
Rv1217c	tetronasin ABC transporter integral membrane protein(Rv1217c)	cell wall and cell wall processes	2087.15	-1.79	0.44	-4.07	4.73E-05	2.08E-03
Rv1182	acyltransferase papA3(papA3)	lipid metabolism	3408.77	-1.88	0.59	-3.21	1.35E-03	2.82E-02
RVnc0005	ncRNA(B55)	stable RNAs	187.69	-1.89	0.44	-4.32	1.60E-05	8.67E-04
Rv0738	hypothetical protein(Rv0738)	conserved hypotheticals	2159.39	-1.91	0.33	-5.70	1.19E-08	1.97E-06
RVnc0024	ncRNA(mcr7)	stable RNAs	287.14	-2.01	0.66	-3.06	2.19E-03	3.90E-02
Rv2765	hydrolase(Rv2765)	intermediary metabolism and respiration	284.54	-2.06	0.46	-4.50	6.88E-06	4.08E-04
Rv3620c	ESAT-6 like protein EsxW(esxW)	cell wall and cell wall processes	1250.31	-2.12	0.64	-3.29	9.89E-04	2.26E-02
Rv3653	PE-PGRS family-related protein PE_PGRS61(PE_PGRS61)	PE/PPE	388.84	-2.18	0.64	-3.42	6.26E-04	1.57E-02
Rv3767c	S-adenosylmethionine-dependent methyltransferase(Rv3767c)	lipid metabolism	1073.82	-2.19	0.65	-3.39	7.05E-04	1.74E-02
Rv1218c	tetronasin ABC transporter ATP-binding protein(Rv1218c)	cell wall and cell wall processes	1033.36	-2.21	0.47	-4.71	2.44E-06	1.86E-04
Rv0826	hypothetical protein(Rv0826)	conserved hypotheticals	12085.23	-2.24	0.72	-3.13	1.77E-03	3.42E-02
Rv1671	membrane protein(Rv1671)	cell wall and cell wall processes	244.50	-2.26	0.57	-3.99	6.63E-05	2.68E-03
Rv3478	PE family protein PPE60(PPE60)	PE/PPE	14496.89	-2.28	0.48	-4.76	1.98E-06	1.67E-04
Rv3473c	peroxidase BpoA(bpoA)	virulence, detoxification, and adaptation	613.77	-2.31	0.30	-7.70	1.32E-14	5.82E-12
Rv2396	acid and phagosome regulated protein AprC(PE_PGRS41)	PE/PPE	10352.15	-2.35	0.67	-3.50	4.74E-04	1.27E-02
Rv1185c	fatty-acid--CoA ligase FadD21(fadD21)	lipid metabolism	8900.68	-2.36	0.52	-4.58	4.65E-06	3.02E-04
Rv3137	histidinol-phosphatase(Rv3137)	intermediary metabolism and respiration	3059.71	-2.38	0.49	-4.88	1.08E-06	9.98E-05

Rv2122c	phosphoribosyl-ATP pyrophosphatase(hisE)	intermediary metabolism and respiration	150.86	-2.40	0.81	-2.96	3.13E-03	4.98E-02
Rv1371	membrane protein(Rv1371)	cell wall and cell wall processes	660.17	-2.44	0.49	-5.00	5.70E-07	5.95E-05
Rv1183	transmembrane transport protein MmpL10(mmpL10)	cell wall and cell wall processes	6705.44	-2.47	0.59	-4.16	3.22E-05	1.50E-03
Rv3416	redox-responsive transcriptional regulator WhiB3(whiB3)	regulatory protein	2243.10	-2.47	0.49	-5.02	5.05E-07	5.42E-05
Rv3379c	1-deoxy-D-xylulose-5-phosphate synthase(dxs2)	intermediary metabolism and respiration	1083.23	-2.52	0.77	-3.27	1.07E-03	2.43E-02
Rv3824c	acyltransferase(papA1)	lipid metabolism	3448.48	-2.56	0.71	-3.64	2.75E-04	9.17E-03
Rv3863	hypothetical protein(Rv3863)	conserved hypotheticals	7895.67	-2.58	0.49	-5.26	1.45E-07	1.91E-05
Rv3686c	hypothetical protein(Rv3686c)	conserved hypotheticals	1427.49	-2.65	0.70	-3.78	1.60E-04	5.70E-03
Rv0609A	hypothetical protein(Rv0609A)	conserved hypotheticals	853.20	-2.78	0.53	-5.25	1.50E-07	1.92E-05
Rv2331	hypothetical protein(Rv2331)	conserved hypotheticals	207.69	-2.81	0.81	-3.45	5.55E-04	1.43E-02
Rv1670	hypothetical protein(Rv1670)	conserved hypotheticals	306.15	-2.94	0.67	-4.42	1.01E-05	5.62E-04
Rv3487c	carboxylesterase LipF(lipF)	intermediary metabolism and respiration	3672.68	-2.99	0.66	-4.55	5.40E-06	3.45E-04
Rv1184c	hypothetical protein(Rv1184c)	conserved hypotheticals	3467.31	-3.08	0.66	-4.69	2.68E-06	1.97E-04
Rv0252	nitrite reductase large subunit NirB(nirB)	intermediary metabolism and respiration	2741.04	-3.11	0.86	-3.61	3.07E-04	9.66E-03
Rv3652	PE-PGRS family-related protein PE_PGRS60(PE_PGRS60)	PE/PPE	618.95	-3.13	0.63	-4.98	6.38E-07	6.33E-05
Rv3825c	phthioceranic/hydroxyphthioceranic acid synthase(pks2)	lipid metabolism	35484.75	-3.23	0.73	-4.42	1.01E-05	5.62E-04
Rv1181	polyketide beta-ketoacyl synthase(pks4)	lipid metabolism	10671.70	-3.25	0.62	-5.23	1.66E-07	2.06E-05
Rv2331A	hypothetical protein(Rv2331A)	conserved hypotheticals	157.06	-3.29	0.77	-4.27	1.93E-05	9.69E-04
Rv3839	hypothetical protein(Rv3839)	conserved hypotheticals	10409.72	-3.30	0.42	-7.92	2.29E-15	1.30E-12
Rv2671	bifunctional diaminohydroxyphosphoribosylaminopyrimidine deaminase/5-amino-6-(5-phosphoribosylamino)uracil reductase(ribD)	intermediary metabolism and respiration	823.81	-3.31	0.60	-5.50	3.70E-08	5.24E-06
Rv3136A	hypothetical protein(Rv3136A)	conserved hypotheticals	4301.67	-3.33	0.54	-6.20	5.49E-10	1.28E-07
Rv1180	polyketide beta-ketoacyl synthase(pks3)	lipid metabolism	2670.74	-3.36	0.73	-4.58	4.63E-06	3.02E-04
Rv0609	ribonuclease VapC28(vapC28)	virulence, detoxification, adaptation	1695.79	-3.37	0.55	-6.14	8.05E-10	1.77E-07

Rv3136	PPE family protein PPE51(PPE51)	PE/PPE	17696.48	-3.54	0.50	-7.13	1.02E-12	4.04E-10
Rv2395B	acid and phagosome regulated protein AprB(aprB)	conserved hypotheticals	4636.22	-3.61	0.71	-5.05	4.47E-07	4.97E-05
Rv2353c	PPE family protein PPE39(PPE39)	PE/PPE	2373.03	-3.74	1.25	-2.98	2.85E-03	4.62E-02
Rv3477	PE family protein PE31(PE31)	PE/PPE	1985.44	-4.04	0.67	-6.07	1.32E-09	2.49E-07
Rv0608	antitoxin VapB28(vapB28)	virulence, detoxification, adaptation	1844.78	-4.06	0.63	-6.45	1.09E-10	2.70E-08

Supplementary Table 2. Genes significantly differentially expressed (absolute Log2FoldChange (L2Fc)> 1, adj. p-value < 0.05) in Bedaquiline (BDQ) resistant clinical isolates (n=6) after 72 hours of BDQ exposure (3.75 µg/ml) compared to controls (BDQ unexposed isolates, t=72h).
(I) Significantly upregulated genes (L2Fc >1, adj. p-value < 0.05)

Functional Category	Locus Tags	Genes	baseMean	L2Fc	lfcSE	stat	pvalue	padj
cell wall and cell processes	Rv1687c	ABC transporter ATP-binding protein(Rv1687c)	3610.09	4.95	0.28	17.86	2.44E-71	4.84E-68
	Rv1686c	ABC transporter permease(Rv1686c)	2705.53	4.59	0.25	18.32	5.72E-75	2.27E-71
	Rv0188	transmembrane protein(Rv0188)	9961.73	2.26	0.23	9.73	2.21E-22	1.29E-20
	Rv2617c	transmembrane protein(Rv2617c)	3928.87	2.23	0.27	8.35	7.01E-17	1.76E-15
	Rv0969	copper-exporting ATPase(ctpV)	85241.84	2.18	0.28	7.68	1.64E-14	2.70E-13
	Rv1992c	cation transporter ATPase G(ctpG)	18472.67	2.05	0.26	8.02	1.05E-15	2.17E-14
	Rv1463	ABC transporter ATP-binding protein(Rv1463)	6972.26	1.95	0.21	9.31	1.29E-20	5.93E-19
	Rv3743c	cation transporter ATPase J(ctpJ)	1299.67	1.87	0.27	7.01	2.31E-12	2.53E-11
	Rv3065	multidrug resistance protein Mmr(mmr)	393.98	1.85	0.18	10.39	2.80E-25	2.61E-23
	Rv3289c	transmembrane protein(Rv3289c)	5817.79	1.85	0.18	9.99	1.75E-23	1.18E-21
	Rv2643	arsenic-transport integral membrane protein ArsC(arsC)	8917.44	1.79	0.27	6.76	1.37E-11	1.36E-10
	Rv1226c	transmembrane protein(Rv1226c)	6262.94	1.77	0.26	6.89	5.50E-12	5.68E-11
	Rv1965	integral membrane protein(yrbE3B)	459.99	1.75	0.22	7.86	3.94E-15	7.29E-14
	Rv2620c	transmembrane protein(Rv2620c)	1990.33	1.65	0.25	6.52	6.89E-11	6.00E-10
	Rv1227c	transmembrane protein(Rv1227c)	2027.50	1.61	0.20	8.25	1.53E-16	3.63E-15
	Rv0849	MFS-type transporter(Rv0849)	3837.57	1.61	0.31	5.12	2.98E-07	1.39E-06
	Rv2053c	transmembrane protein FxsA(fxsA)	7950.18	1.60	0.27	5.92	3.14E-09	2.12E-08
	Rv2158c	UDP-N-acetylmuramoylalanyl-D-glutamate--2,6-diaminopimelate ligase(murE)	4916.45	1.57	0.18	8.73	2.53E-18	8.09E-17

	Rv2025c	cation efflux system protein(Rv2025c)	1169.66	1.52	0.18	8.25	1.56E-16	3.69E-15
	Rv0064	transmembrane protein(Rv0064)	8521.73	1.52	0.14	10.70	1.03E-26	1.16E-24
	Rv0970	integral membrane protein(Rv0970)	7056.40	1.52	0.30	4.98	6.40E-07	2.84E-06
	Rv0847	lipoprotein LpqS(lpqS)	8314.80	1.46	0.26	5.61	2.05E-08	1.19E-07
	Rv1224	Sec-independent protein translocase protein TatB(tatB)	7011.02	1.39	0.26	5.38	7.40E-08	3.85E-07
	Rv1972	Mce associated membrane protein(Rv1972)	160.47	1.37	0.17	8.28	1.22E-16	2.91E-15
	Rv2963	integral membrane protein(Rv2963)	3986.15	1.30	0.24	5.35	8.61E-08	4.43E-07
	rv1517	Conserved hypothetical transmembrane protein	239.94	1.30	0.29	4.55	5.29E-06	1.98E-05
	Rv0475	heparin binding hemagglutinin HbhA(hbhA)	13217.16	1.24	0.19	6.61	3.84E-11	3.48E-10
	Rv2688c	antibiotic ABC transporter ATP-binding protein(Rv2688c)	1614.04	1.24	0.11	11.18	4.86E-29	6.64E-27
	Rv2051c	polyprenol-monophosphomannose synthase(ppm1)	16692.87	1.21	0.26	4.75	2.08E-06	8.38E-06
	Rv1004c	membrane protein(Rv1004c)	3554.47	1.21	0.16	7.62	2.44E-14	3.89E-13
	Rv1964	integral membrane protein(yrbE3A)	938.20	1.19	0.26	4.59	4.37E-06	1.66E-05
	Rv0290	ESX-3 secretion system protein EccD(eccD3)	20757.28	1.18	0.15	7.69	1.48E-14	2.45E-13
	Rv0116c	L,D-transpeptidase LdtA(ldtA)	1596.36	1.15	0.17	6.72	1.76E-11	1.70E-10
	Rv0985c	large-conductance ion mechanosensitive channel(mscL)	2492.86	1.13	0.18	6.45	1.14E-10	9.55E-10
	Rv1973	Mce associated membrane protein(Rv1973)	159.16	1.13	0.20	5.57	2.56E-08	1.44E-07
	Rv3270	manganese/zinc-exporting P-type ATPase(ctpC)	83226.26	1.11	0.26	4.24	2.20E-05	7.25E-05
	Rv0538	membrane protein(Rv0538)	6871.74	1.09	0.17	6.52	7.20E-11	6.20E-10
	Rv2729c	integral membrane protein(Rv2729c)	1563.79	1.07	0.22	4.88	1.05E-06	4.43E-06
	Rv2157c	UDP-N-acetylmuramoyl-tripeptide--D-alanyl-D-alanine ligase(murF)	1601.00	1.06	0.16	6.72	1.79E-11	1.72E-10
	Rv1072	transmembrane protein(Rv1072)	44494.87	1.06	0.14	7.45	9.55E-14	1.39E-12
	Rv2553c	membrane protein(Rv2553c)	7622.22	1.03	0.15	6.69	2.18E-11	2.07E-10
	Rv0037c	MFS-type transporter(Rv0037c)	1688.96	1.01	0.11	9.23	2.65E-20	1.17E-18
	Rv2834c	sn-glycerol-3-phosphate ABC transporter permease UgpE(ugpE)	239.61	0.99	0.22	4.59	4.51E-06	1.72E-05
	rv3728	Probable conserved two-domain membrane protein	3396.56	0.71	0.27	2.60	9.37E-03	1.75E-02
	Rv0411c	glutamine-binding lipoprotein GlnH(glnH)	3748.22	1.11	0.15	7.57	3.64E-14	5.64E-13

	Rv0841	transmembrane protein(Rv0841)	2955.83	1.09	0.27	3.96	7.57E-05	2.26E-04
	Rv0288	ESAT-6-like protein EsxH(esxH)	5324.81	1.04	0.17	6.02	1.80E-09	1.26E-08
	Rv0289	ESX-3 secretion-associated protein EspG3(espG3)	11826.83	1.03	0.21	4.91	8.90E-07	3.83E-06
**intermediary metabolism and respiration**	Rv1937	oxygenase(Rv1937)	4384.20	2.51	0.25	10.04	1.04E-23	7.40E-22
	Rv1936	monooxygenase(Rv1936)	3787.61	2.51	0.31	8.15	3.79E-16	8.49E-15
	Rv3161c	dioxygenase(Rv3161c)	10637.34	2.46	0.28	8.75	2.14E-18	7.06E-17
	Rv2501c	acetyl/propionyl-CoA carboxylase subuit alpha(accA1)	7427.99	2.36	0.20	11.84	2.50E-32	5.50E-30
	Rv3534c	4-hydroxy-2-oxovalerate aldolase(hsaF)	3913.40	2.26	0.16	14.01	1.42E-44	1.12E-41
	Rv0467	isocitrate lyase(icl1)	5878.31	2.22	0.25	8.69	3.65E-18	1.15E-16
	Rv3535c	acetaldehyde dehydrogenase(hsaG)	3264.40	2.20	0.16	13.92	5.05E-44	3.33E-41
	Rv0983	serine protease PepD(pepD)	35226.24	2.13	0.18	11.86	1.99E-32	4.63E-30
	Rv3536c	hydratase(hsaE)	3548.68	2.04	0.16	12.79	1.75E-37	6.94E-35
	Rv1464	cysteine desulfurase(csd)	11996.55	2.04	0.22	9.22	3.00E-20	1.29E-18
	Rv1939	oxidoreductase(Rv1939)	608.56	2.03	0.24	8.57	1.00E-17	2.96E-16
	Rv3203	lipase LipV(lipV)	2021.69	1.99	0.25	7.79	6.45E-15	1.14E-13
	Rv2497c	3-methyl-2-oxobutanoate dehydrogenase subunit alpha(bkdA)	11839.95	1.94	0.16	12.21	2.70E-34	8.25E-32
	Rv2503c	succinyl-CoA:3-ketoacid-CoA transferase subunit B(scoB)	2376.10	1.92	0.25	7.77	7.69E-15	1.34E-13
	Rv3290c	L-lysine-epsilon aminotransferase(lat)	64780.41	1.87	0.17	10.92	8.86E-28	1.13E-25
	Rv2499c	oxidase regulatory-like protein(Rv2499c)	1453.41	1.87	0.20	9.55	1.36E-21	7.10E-20
	Rv0560c	benzoquinone methyltransferase(Rv0560c)	1141.62	1.84	0.21	8.91	5.10E-19	1.91E-17
	Rv2496c	3-methyl-2-oxobutanoate dehydrogenase subunit beta(bkdB)	7756.21	1.82	0.16	11.58	5.30E-31	1.00E-28
	Rv2502c	acetyl-/propionyl-CoA carboxylase subunit beta(accD1)	4449.88	1.82	0.21	8.51	1.75E-17	4.96E-16
	Rv0984	pterin-4-alpha-carbinolamine dehydratase(moaB2)	9874.45	1.74	0.17	10.07	7.25E-24	5.23E-22
	Rv0848	cysteine synthase CysK(cysK2)	12056.68	1.73	0.26	6.63	3.27E-11	3.03E-10
	Rv3175	amidase(Rv3175)	2046.37	1.72	0.32	5.45	5.12E-08	2.75E-07
	Rv0753c	methylmalonate-semialdehyde dehydrogenase(mmsA)	10759.36	1.72	0.22	7.94	1.98E-15	3.85E-14
	Rv0751c	3-hydroxyisobutyrate dehydrogenase(mmsB)	3637.47	1.72	0.21	8.09	5.79E-16	1.25E-14

	Rv3406	dioxygenase(Rv3406)	2912.46	1.72	0.26	6.67	2.63E-11	2.47E-10
	Rv0186	beta-glucosidase BglS(bglS)	43068.79	1.70	0.20	8.38	5.14E-17	1.33E-15
	Rv1654	acetylglutamate kinase(argB)	1387.37	1.69	0.20	8.59	8.71E-18	2.62E-16
	Rv3206c	adenylyltransferase/sulfurtransferase MoeZ(moeB1)	19511.64	1.68	0.20	8.48	2.16E-17	6.01E-16
	Rv1940	riboflavin biosynthesis protein RibA(ribA1)	1660.04	1.66	0.22	7.43	1.09E-13	1.56E-12
	Rv1465	nitrogen fixation related protein(Rv1465)	2792.44	1.66	0.20	8.23	1.83E-16	4.23E-15
	Rv2504c	succinyl-CoA:3-ketoacid-CoA transferase subunit A(scoA)	4252.73	1.65	0.24	6.78	1.18E-11	1.18E-10
	Rv2161c	hypothetical protein(Rv2161c)	9564.36	1.64	0.27	6.13	8.81E-10	6.46E-09
	Rv2498c	citrate (pro-3S)-lyase subunit beta(citE)	1943.35	1.60	0.17	9.49	2.28E-21	1.15E-19
	Rv3502c	3-oxoacyl-ACP reductase(Rv3502c)	2423.10	1.59	0.14	11.48	1.61E-30	2.91E-28
	Rv1653	bifunctional glutamate N-acetyltransferase/amino-acid acetyltransferase(argJ)	1982.94	1.58	0.17	9.55	1.34E-21	7.10E-20
	Rv3503c	ferredoxin FdxD(fdxD)	747.14	1.57	0.18	8.75	2.17E-18	7.11E-17
	Rv0765c	oxidoreductase(Rv0765c)	4167.76	1.46	0.29	5.00	5.60E-07	2.52E-06
	Rv1652	N-acetyl-gamma-glutamyl-phoshate reductase(argC)	702.33	1.44	0.20	7.22	5.28E-13	6.55E-12
	Rv1655	acetylornithine aminotransferase(argD)	1443.37	1.43	0.17	8.33	7.99E-17	1.98E-15
	Rv2280	Rv2280	3736.63	1.42	0.18	7.93	2.26E-15	4.37E-14
	Rv2495c	branched-chain keto acid dehydrogenase E2 component(bkdC)	7508.40	1.41	0.16	8.63	6.11E-18	1.85E-16
	Rv3837c	phosphoglycerate mutase(Rv3837c)	8521.53	1.41	0.20	7.03	2.12E-12	2.35E-11
	Rv0374c	carbon monoxyde dehydrogenase small subunit(Rv0374c)	127.60	1.39	0.27	5.10	3.32E-07	1.53E-06
	Rv1599	histidinol dehydrogenase(hisD)	8047.80	1.37	0.13	10.50	8.43E-26	8.36E-24
	Rv0764c	lanosterol 14-alpha demethylase(cyp51)	5526.54	1.37	0.29	4.69	2.78E-06	1.10E-05
	Rv0291	membrane-anchored mycosin MycP(mycP3)	14827.64	1.34	0.22	6.14	8.18E-10	6.02E-09
	Rv0620	galactokinase(galK)	119.55	1.31	0.28	4.70	2.65E-06	1.05E-05
	Rv1832	glycine dehydrogenase(gcvB)	26037.31	1.28	0.13	9.96	2.26E-23	1.47E-21
	Rv0539	dolichyl-phosphate sugar synthase(Rv0539)	4456.42	1.28	0.13	10.23	1.53E-24	1.24E-22
	Rv2667	ATP-dependent protease ATP-binding subunit ClpC(clpC2)	548.87	1.28	0.27	4.67	3.08E-06	1.21E-05
	Rv2064	precorrin-3B synthase(cobG)	2098.42	1.25	0.17	7.32	2.46E-13	3.23E-12

	Rv2677c	protoporphyrinogen oxidase(hemY)	3528.40	1.24	0.17	7.21	5.40E-13	6.65E-12
	Rv0375c	carbon monoxyde dehydrogenase medium subunit(Rv0375c)	241.50	1.23	0.22	5.59	2.21E-08	1.26E-07
	Rv1326c	1,4-alpha-glucan branching protein(glgB)	3959.65	1.22	0.13	9.72	2.41E-22	1.38E-20
	Rv0162c	zinc-type alcohol dehydrogenase subunit E(adhE1)	901.28	1.22	0.21	5.72	1.05E-08	6.55E-08
	Rv0846c	oxidase(Rv0846c)	3623.86	1.21	0.23	5.25	1.49E-07	7.28E-07
	Rv1223	serine protease HtrA(htrA)	31198.89	1.21	0.22	5.41	6.28E-08	3.34E-07
	Rv2455c	2-oxoglutarate oxidoreductase subunit KorA(Rv2455c)	16091.54	1.20	0.12	9.98	1.83E-23	1.21E-21
	Rv0763c	ferredoxin(Rv0763c)	218.19	1.20	0.27	4.41	1.05E-05	3.69E-05
	Rv1605	imidazole glycerol phosphate synthase subunit HisF(hisF)	2320.19	1.19	0.19	6.29	3.08E-10	2.41E-09
	Rv2753c	4-hydroxy-tetrahydrodipicolinate synthase(dapA)	7429.85	1.19	0.19	6.29	3.17E-10	2.47E-09
	Rv0077c	oxidoreductase(Rv0077c)	414.98	1.19	0.22	5.29	1.26E-07	6.24E-07
	Rv2727c	tRNA delta(2)-isopentenylpyrophosphate transferase(miaA)	917.56	1.18	0.18	6.52	6.95E-11	6.03E-10
	Rv2540c	chorismate synthase(aroF)	11258.59	1.16	0.18	6.37	1.91E-10	1.54E-09
	Rv1256c	cytochrome P450 Cyp130(cyp130)	1975.83	1.15	0.13	8.82	1.12E-18	3.87E-17
	Rv1656	ornithine carbamoyltransferase(argF)	834.67	1.15	0.16	7.30	2.88E-13	3.72E-12
	Rv2552c	shikimate 5-dehydrogenase(aroE)	3345.39	1.15	0.18	6.33	2.43E-10	1.93E-09
	Rv0373c	carbon monoxyde dehydrogenase large subunit(Rv0373c)	573.84	1.14	0.20	5.61	1.98E-08	1.16E-07
	Rv2251	flavoprotein(Rv2251)	1275.39	1.14	0.17	6.61	3.84E-11	3.48E-10
	Rv2852c	malate:quinone oxidoreductase(mqo)	3472.51	1.13	0.11	10.62	2.30E-26	2.47E-24
	Rv2850c	magnesium chelatase(Rv2850c)	4757.00	1.13	0.11	10.10	5.49E-24	4.11E-22
	Rv0897c	oxidoreductase(Rv0897c)	2226.17	1.10	0.21	5.16	2.54E-07	1.19E-06
	Rv1327c	alpha-1,4-glucan:maltose-1-phosphate maltosyltransferase(glgE)	4731.40	1.09	0.13	8.70	3.41E-18	1.08E-16
	Rv2539c	shikimate kinase(aroK)	2768.93	1.08	0.20	5.45	5.14E-08	2.76E-07
	Rv1130	2-methylcitrate dehydratase(prpD)	4592.90	1.06	0.33	3.16	1.57E-03	3.53E-03
	rv1604	inositol-monophosphatase ImpA(impA)	2148.74	1.05	0.18	5.93	3.07E-09	2.07E-08
	Rv3086	alcohol dehydrogenase D(adhD)	2252.55	1.24	0.26	4.86	1.17E-06	4.91E-06

	Rv1621c	cytochrome biosyntheisis ABC transporter ATP-binding protein/permease CydD(cydD)	3409.38	1.22	0.20	5.96	2.59E-09	1.78E-08
	Rv0331	dehydrogenase/reductase(Rv0331)	1551.22	1.15	0.27	4.23	2.34E-05	7.64E-05
	Rv0766c	cytochrome P450 Cyp123(cyp123)	3732.70	1.07	0.25	4.23	2.29E-05	7.50E-05
	Rv2851c	GCN5-like N-acetyltransferase(Rv2851c)	1561.96	1.07	0.10	10.47	1.25E-25	1.21E-23
	Rv1285	sulfate adenylyltransferase subunit 2(cysD)	9115.19	1.04	0.20	5.33	9.82E-08	4.99E-07
	Rv1854c	NADH dehydrogenase(ndh)	4758.37	1.03	0.11	8.95	3.56E-19	1.37E-17
	Rv1622c	cytochrome D ubiquinol oxidase subunit II CydB(cydB)	1949.61	1.02	0.21	4.97	6.79E-07	2.99E-06
	Rv3545c	steroid C26-monooxygenase(cyp125)	1726.00	1.02	0.19	5.25	1.53E-07	7.49E-07
	Rv3537	3-oxosteroid 1-dehydrogenase(kstD)	1754.81	1.02	0.16	6.27	3.68E-10	2.85E-09
	Rv3538	dehydrogenase(Rv3538)	622.39	1.01	0.19	5.27	1.33E-07	6.55E-07
	Rv2583c	bifunctional (p)ppGpp synthase/hydrolase RelA(relA)	7787.80	1.00	0.10	10.26	1.05E-24	8.86E-23
	Rv1595	L-aspartate oxidase(nadB)	31912.17	1.00	0.24	4.21	2.56E-05	8.32E-05
**lipid metabolism**	Rv0244c	acyl-CoA dehydrogenase FadE5(fadE5)	16767.07	1.98	0.22	8.80	1.32E-18	4.52E-17
	Rv0975c	acyl-CoA dehydrogenase FadE13(fadE13)	1592.98	1.97	0.21	9.40	5.66E-21	2.77E-19
	Rv0752c	acyl-CoA dehydrogenase FadE9(fadE9)	5332.52	1.89	0.21	8.92	4.73E-19	1.79E-17
	Rv0973c	acetyl/propionyl-CoA carboxylase subuit alpha(accA2)	4198.24	1.79	0.23	7.81	5.73E-15	1.02E-13
	Rv0974c	acetyl-/propionyl-CoA carboxylase subunit beta(accD2)	1677.47	1.77	0.22	8.09	6.11E-16	1.30E-14
	Rv2500c	acyl-CoA dehydrogenase FadE19(fadE19)	2863.09	1.76	0.19	9.03	1.67E-19	6.76E-18
	Rv1935c	enoyl-CoA hydratase EchA13(echA13)	718.13	1.61	0.30	5.33	9.64E-08	4.90E-07
	Rv3515c	acyl-CoA synthetase(fadD19)	1993.63	1.59	0.21	7.61	2.67E-14	4.21E-13
	Rv0972c	acyl-CoA dehydrogenase fadE12(fadE12)	2131.75	1.42	0.22	6.55	5.64E-11	5.00E-10
	Rv3504	acyl-CoA dehydrogenase FadE26(fadE26)	1276.34	1.41	0.12	12.00	3.57E-33	9.43E-31
	Rv2382c	polyketide synthetase(mbtC)	1967.88	1.39	0.29	4.78	1.72E-06	7.01E-06
	Rv2484c	diacyglycerol O-acyltransferase(Rv2484c)	6063.06	1.39	0.13	10.78	4.45E-27	5.35E-25
	Rv3505	acyl-CoA dehydrogenase FadE27(fadE27)	967.18	1.38	0.13	10.73	7.46E-27	8.70E-25
	Rv0971c	enoyl-CoA hydratase EchA7(echA7)	3324.39	1.32	0.26	5.18	2.20E-07	1.04E-06
	Rv2383c	phenyloxazoline synthase(mbtB)	5603.01	1.32	0.25	5.27	1.37E-07	6.75E-07
	Rv1934c	acyl-CoA dehydrogenase FadE17(fadE17)	601.51	1.28	0.25	5.13	2.96E-07	1.38E-06

	Rv3546	acetyl-CoA acetyltransferase FadA(fadA5)	1379.48	1.28	0.17	7.53	4.98E-14	7.57E-13
	Rv0564c	glycerol-3-phosphate dehydrogenase(gpdA1)	4665.61	1.25	0.16	7.90	2.77E-15	5.27E-14
	Rv3573c	acyl-CoA dehydrogenase FadE34(fadE34)	919.68	1.17	0.14	8.13	4.27E-16	9.46E-15
	Rv1492	methylmalonyl-CoA mutase small subunit(mutA)	3774.85	1.15	0.14	8.25	1.65E-16	3.83E-15
	Rv1715	3-hydroxybutyryl-CoA dehydrogenase FadB(fadB3)	683.78	1.10	0.25	4.33	1.51E-05	5.10E-05
	Rv0649	malonyl CoA-acyl carrier protein transacylase(fabD2)	251.84	1.05	0.24	4.42	9.93E-06	3.51E-05
	rv2934	phthiocerol synthesis polyketide synthase type I PpsD(ppsD)	26566.82	0.92	0.30	3.10	1.95E-03	4.31E-03
	Rv3513c	fatty-acid--CoA ligase FadD18(fadD18)	314.67	1.12	0.17	6.73	1.65E-11	1.61E-10
	Rv3409c	cholesterol oxidase(choD)	8776.77	1.08	0.19	5.63	1.80E-08	1.05E-07
	Rv3061c	acyl-CoA dehydrogenase FadE22(fadE22)	6485.10	1.05	0.16	6.55	5.95E-11	5.23E-10
	Rv3543c	acyl-CoA dehydrogenase FadE29(fadE29)	1299.88	1.05	0.14	7.33	2.26E-13	3.00E-12
	Rv0672	acyl-CoA dehydrogenase FadE8(fadE8)	3805.97	1.04	0.13	8.13	4.34E-16	9.56E-15
	Rv2483c	bifunctional L-3-phosphoserine phosphatase/1-acyl-sn-glycerol-3-phosphate acyltransferase(plsC)	3224.48	1.02	0.13	7.92	2.39E-15	4.61E-14
	Rv0468	3-hydroxybutyryl-CoA dehydrogenase(fadB2)	1247.87	1.01	0.25	4.12	3.81E-05	1.20E-04
	Rv3800c	polyketide synthase(pks13)	95086.18	1.01	0.16	6.36	1.95E-10	1.57E-09
	Rv0673	enoyl-CoA hydratase EchA4(echA4)	1780.56	1.01	0.14	7.42	1.16E-13	1.65E-12
	Rv1493	methylmalonyl-CoA mutase large subunit(mutB)	3063.74	1.01	0.14	7.12	1.12E-12	1.31E-11
**virulence, detoxification, and adaptation**	Rv1955	toxin HigB(higB)	2049.08	2.70	0.22	12.50	7.80E-36	2.58E-33
	Rv1938	epoxide hydrolase EphB(ephB)	1126.50	2.62	0.26	9.92	3.56E-23	2.24E-21
	Rv0251c	heat shock protein(hsp)	54556.94	2.48	0.22	11.18	5.13E-29	6.78E-27
	Rv1956	antitoxin HigA(higA)	1513.52	2.23	0.18	12.70	5.83E-37	2.10E-34
	Rv1968	Mce family protein Mce3C(mce3C)	418.98	2.18	0.19	11.64	2.44E-31	4.83E-29
	Rv1967	Mce family protein Mce3B(mce3B)	373.63	2.16	0.21	10.39	2.83E-25	2.61E-23
	Rv0186A	metallothionein(mymT)	11197.76	2.09	0.22	9.54	1.49E-21	7.68E-20
	Rv0440	molecular chaperone GroEL(groEL2)	93262.37	2.00	0.18	11.25	2.41E-29	3.41E-27
	Rv3176c	epoxide hydrolase MesT(mesT)	2008.13	1.99	0.35	5.69	1.23E-08	7.56E-08
	Rv1970	Mce family lipoprotein LprM(lprM)	337.10	1.96	0.15	13.15	1.81E-39	8.95E-37

	Rv3417c	chaperonin GroEL(groEL1)	23938.68	1.80	0.15	11.66	2.11E-31	4.40E-29
	Rv1966	Mce family protein Mce3A(mce3A)	451.00	1.74	0.24	7.41	1.23E-13	1.74E-12
	Rv1908c	catalase-peroxidase(katG)	12211.63	1.65	0.30	5.45	4.94E-08	2.66E-07
	Rv0384c	chaperone protein ClpB(clpB)	38753.61	1.65	0.22	7.40	1.37E-13	1.93E-12
	Rv1991A	antitoxin MazE6(mazE6)	630.86	1.58	0.22	7.03	2.13E-12	2.35E-11
	Rv2546	ribonuclease VapC18(vapC18)	151.84	1.55	0.35	4.46	8.10E-06	2.91E-05
	Rv1991c	mRNA interferase MazF6(mazF6)	816.80	1.51	0.18	8.57	1.05E-17	3.08E-16
	Rv3418c	chaperonin GroES(groES)	4134.27	1.50	0.17	8.95	3.69E-19	1.41E-17
	Rv0351	stress response protein GrpE(grpE)	26476.62	1.50	0.21	7.14	9.60E-13	1.14E-11
	Rv1971	Mce family protein Mce3F(mce3F)	294.84	1.49	0.15	10.19	2.27E-24	1.80E-22
	Rv0550c	antitoxin VapB3(vapB3)	122.85	1.47	0.21	7.04	1.91E-12	2.13E-11
	Rv0563	protease HtpX(htpX)	9180.76	1.47	0.19	7.78	7.43E-15	1.30E-13
	Rv0352	chaperone protein DnaJ(dnaJ1)	31216.40	1.43	0.23	6.13	9.02E-10	6.60E-09
	Rv0350	chaperone protein DnaK(dnaK)	70186.51	1.42	0.17	8.17	3.15E-16	7.14E-15
	Rv0064A	antitoxin VapB1(vapB1)	202.88	1.37	0.19	7.11	1.16E-12	1.35E-11
	Rv1969	Mce family protein Mce3D(mce3D)	264.06	1.37	0.12	11.25	2.26E-29	3.32E-27
	Rv3358	toxin RelK(relK)	71.39	1.31	0.23	5.76	8.53E-09	5.42E-08
	Rv2373c	chaperone protein DnaJ(dnaJ2)	13457.95	1.30	0.22	5.84	5.09E-09	3.34E-08
	Rv0549c	ribonuclease VapC3(vapC3)	624.06	1.22	0.14	8.66	4.68E-18	1.46E-16
	Rv3181c	antitoxin VapB45(Rv3181c)	167.15	1.14	0.27	4.25	2.13E-05	7.05E-05
	Rv2374c	heat-inducible transcription repressor HrcA(hrcA)	4894.75	1.13	0.11	10.00	1.50E-23	1.03E-21
	rv3407	antitoxin VapB47(vapB47)	1242.66	1.12	0.19	5.84	5.12E-09	3.35E-08
	Rv3180c	ribonuclease VapC45(Rv3180c)	276.44	1.09	0.22	5.02	5.22E-07	2.36E-06
	Rv3357	antitoxin RelJ(relJ)	55.73	1.16	0.21	5.49	4.03E-08	2.22E-07
	Rv1960c	antitoxin ParD1(parD1)	222.96	1.11	0.22	4.97	6.63E-07	2.94E-06
	Rv1959c	toxin ParE1(parE1)	474.64	1.06	0.18	5.94	2.91E-09	1.99E-08
	Rv0353	heat shock protein transcriptional repressor HspR(hspR)	3105.28	1.04	0.19	5.37	7.85E-08	4.08E-07
**regulatory proteins**	Rv3160c	TetR family transcriptional regulator(Rv3160c)	3514.37	1.89	0.27	7.10	1.27E-12	1.47E-11
	Rv1957	SecB-like chaperone(Rv1957)	687.21	1.88	0.20	9.52	1.69E-21	8.58E-20

	Rv2642	ArsR family transcriptional regulator(Rv2642)	8580.67	1.82	0.29	6.37	1.88E-10	1.52E-09
	Rv0967	copper-sensing transcriptional repressor CsoR(csoR)	13465.76	1.77	0.24	7.31	2.73E-13	3.55E-12
	Rv2745c	transcriptional regulator ClgR(clgR)	11382.53	1.75	0.22	8.05	8.38E-16	1.77E-14
	Rv1985c	HTH-type transcriptional regulator(Rv1985c)	1388.35	1.66	0.20	8.48	2.22E-17	6.13E-16
	Rv3080c	serine/threonine-protein kinase PknK(pknK)	2318.68	1.65	0.14	11.99	4.02E-33	9.97E-31
	Rv3066	DeoR family transcriptional regulator(Rv3066)	867.88	1.60	0.15	10.62	2.52E-26	2.63E-24
	Rv2621c	transcriptional regulator(Rv2621c)	6515.70	1.57	0.23	6.70	2.04E-11	1.94E-10
	Rv0275c	transcriptional regulator(Rv0275c)	3753.98	1.49	0.17	8.65	5.15E-18	1.58E-16
	Rv1460	transcriptional regulator(Rv1460)	5763.11	1.46	0.25	5.84	5.26E-09	3.42E-08
	Rv2324	AsnC family transcriptional regulator(Rv2324)	575.45	1.39	0.14	9.61	7.31E-22	4.09E-20
	Rv0324	transcriptional regulator(Rv0324)	495.71	1.38	0.14	10.14	3.54E-24	2.70E-22
	Rv2912c	TetR family HTH-type transcriptional regulator(Rv2912c)	7341.08	1.29	0.19	6.71	1.99E-11	1.89E-10
	Rv2250c	HTH-type transcriptional regulator(Rv2250c)	170.54	1.25	0.16	7.59	3.25E-14	5.05E-13
	Rv1963c	transcriptional repressor Mce3R(mce3R)	841.33	1.24	0.20	6.33	2.52E-10	1.99E-09
	Rv0767c	HTH-type transcriptional regulator(Rv0767c)	2851.52	1.16	0.22	5.30	1.15E-07	5.77E-07
	Rv1994c	HTH-type transcriptional regulator CmtR(cmtR)	2785.94	1.12	0.25	4.55	5.35E-06	2.00E-05
	Rv1909c	ferric uptake regulation protein FurA(furA)	675.62	1.11	0.35	3.13	1.72E-03	3.84E-03
	Rv1657	arginine repressor(argR)	392.90	1.10	0.15	7.13	9.77E-13	1.16E-11
	rv1129c	transcriptional regulator(Rv1129c)	998.24	1.04	0.31	3.32	9.11E-04	2.15E-03
	Rv1028c	sensor protein KdpD(kdpD)	1552.24	1.07	0.13	8.32	8.45E-17	2.07E-15
	Rv2011c	hypothetical protein(Rv2011c)	1007.92	1.07	0.25	4.28	1.88E-05	6.28E-05
	Rv0410c	serine/threonine-protein kinase PknG(pknG)	7763.01	1.02	0.15	6.90	5.16E-12	5.38E-11
**information pathways**	Rv0670	endonuclease IV(end)	4682.21	1.67	0.15	11.37	5.67E-30	9.36E-28
	Rv2710	RNA polymerase sigma factor SigB(sigB)	59971.69	1.67	0.18	9.21	3.23E-20	1.38E-18
	Rv3834c	serine--tRNA ligase(serS)	994.71	1.62	0.24	6.73	1.73E-11	1.68E-10
	Rv2554c	Holliday junction resolvase(Rv2554c)	4109.39	1.60	0.19	8.44	3.09E-17	8.31E-16
	Rv3202c	ATP-dependent DNA helicase(Rv3202c)	6782.85	1.57	0.21	7.62	2.55E-14	4.05E-13
	Rv3287c	anti-sigma factor RsbW(rsbW)	3590.92	1.52	0.19	8.05	8.39E-16	1.77E-14

	Rv0233	ribonucleoside-diphosphate reductase subunit beta NrdB(nrdB)	3314.91	1.45	0.20	7.35	1.95E-13	2.64E-12
	Rv2555c	alanine--tRNA ligase(alaS)	33011.80	1.39	0.15	8.98	2.61E-19	1.01E-17
	Rv3204	DNA-methyltransferase(Rv3204)	638.26	1.38	0.19	7.37	1.68E-13	2.32E-12
	Rv3201c	ATP-dependent DNA helicase(Rv3201c)	3384.60	1.33	0.18	7.48	7.62E-14	1.12E-12
	Rv2838c	ribosome-binding factor RbfA(rbfA)	7256.76	1.31	0.20	6.55	5.95E-11	5.23E-10
	Rv1259	uracil DNA glycosylase(udgB)	548.56	1.28	0.22	5.92	3.30E-09	2.20E-08
	Rv2839c	translation initiation factor IF-2(infB)	50714.46	1.28	0.18	6.98	2.88E-12	3.11E-11
	Rv3370c	error-prone DNA polymerase(dnaE2)	5028.32	1.21	0.17	7.24	4.35E-13	5.46E-12
	Rv1221	ECF RNA polymerase sigma factor SigE(sigE)	25455.29	1.12	0.21	5.34	9.31E-08	4.75E-07
	Rv3286c	RNA polymerase sigma factor SigF(sigF)	2904.36	1.10	0.16	6.84	8.15E-12	8.26E-11
	Rv2614c	threonine--tRNA ligase(thrS)	3458.06	1.08	0.16	6.91	4.81E-12	5.02E-11
	Rv3420c	ribosomal-protein-alanine acetyltransferase RimI(rimI)	1114.95	1.07	0.20	5.45	5.10E-08	2.74E-07
	rv1406	methionyl-tRNA formyltransferase(fmt)	1367.34	1.07	0.14	7.86	3.79E-15	7.09E-14
	Rv3062	DNA ligase(ligB)	2938.41	1.07	0.22	4.85	1.22E-06	5.06E-06
	Rv1222	anti-sigma E factor RseA(rseA)	14296.68	1.05	0.19	5.61	2.08E-08	1.20E-07
**stable RNAs**	rnpB	miscRNA(rnpB)	1857749.90	2.40	0.28	8.47	2.36E-17	6.47E-16
	ssr	miscRNA(ssr)	3195926.47	2.06	0.18	11.35	7.35E-30	1.17E-27
	rrl	23S ribosomal RNA(rrl)	13716446.43	2.01	0.20	10.09	6.11E-24	4.49E-22
	rrs	16S ribosomal RNA(rrs)	41376.63	1.79	0.41	4.33	1.48E-05	5.02E-05
	MTS2823	ncRNA(MTS2823)	2834736.42	1.56	0.44	3.56	3.68E-04	9.42E-04
	rrf	5S ribosomal RNA(rrf)	14700.34	1.10	0.31	3.55	3.90E-04	9.92E-04
**PE/PPE**	Rv0754	PE-PGRS family protein PE_PGRS11(PE_PGRS11)	6154.59	3.28	0.21	15.28	1.05E-52	1.39E-49
	Rv0978c	PE-PGRS family protein PE_PGRS17(PE_PGRS17)	799.01	2.42	0.29	8.44	3.11E-17	8.31E-16
	Rv0834c	PE-PGRS family protein PE_PGRS14(PE_PGRS14)	21780.90	2.41	0.31	7.70	1.40E-14	2.36E-13
	Rv2615c	PE-PGRS family protein PE_PGRS45(PE_PGRS45)	2418.91	2.21	0.26	8.36	6.09E-17	1.54E-15
	Rv1808	PPE family protein PPE32(PPE32)	2095.70	1.79	0.23	7.86	3.94E-15	7.29E-14
	Rv1806	PE family protein PE20(PE20)	468.81	1.64	0.28	5.94	2.90E-09	1.98E-08
	Rv3533c	PPE family protein PPE62(PPE62)	1511.24	1.58	0.15	10.89	1.24E-27	1.54E-25
	Rv0833	PE-PGRS family protein PE_PGRS13(PE_PGRS13)	3136.03	1.53	0.19	7.91	2.53E-15	4.84E-14

	Rv0872c	PE-PGRS family protein PE_PGRS15(PE_PGRS15)	13847.14	1.51	0.14	10.55	4.95E-26	5.04E-24
	Rv0278c	PE-PGRS family protein PE_PGRS3(Rv0278c)	1289.71	1.38	0.15	9.10	9.22E-20	3.81E-18
	Rv3507	PE-PGRS family protein PE_PGRS53(PE_PGRS53)	3760.11	1.34	0.14	9.89	4.53E-23	2.81E-21
	Rv1801	PPE family protein PPE29(PPE29)	1613.66	1.33	0.31	4.28	1.88E-05	6.28E-05
	Rv1651c	PE-PGRS family protein PE_PGRS30(PE_PGRS30)	3110.65	1.33	0.13	10.17	2.63E-24	2.04E-22
	Rv1818c	PE-PGRS family protein PE_PGRS33(PE_PGRS33)	5209.51	1.27	0.18	7.18	6.98E-13	8.38E-12
	Rv1809	PPE family protein PPE33(PPE33)	2135.91	1.26	0.20	6.26	3.94E-10	3.04E-09
	Rv1840c	PE-PGRS family protein PE_PGRS34(PE_PGRS34)	1548.79	1.26	0.21	6.08	1.21E-09	8.70E-09
	Rv2634c	PE-PGRS family protein PE_PGRS46(PE_PGRS46)	690.54	1.19	0.12	9.61	7.28E-22	4.09E-20
	Rv3514	PE-PGRS family protein PE_PGRS57(PE_PGRS57)	3548.87	1.17	0.27	4.35	1.34E-05	4.63E-05
	Rv0915c	PPE family protein PPE14(PPE14)	459.36	1.16	0.27	4.31	1.61E-05	5.44E-05
	Rv0747	PE-PGRS family protein PE_PGRS10(PE_PGRS10)	4288.93	1.14	0.18	6.50	8.23E-11	7.07E-10
	Rv3367	PE-PGRS family protein PE_PGRS51(PE_PGRS51)	2529.32	1.13	0.12	9.17	4.58E-20	1.93E-18
	Rv1802	PPE family protein PPE30(PPE30)	1209.27	1.12	0.29	3.86	1.13E-04	3.26E-04
	Rv0279c	PE-PGRS family protein PE_PGRS4(PE_PGRS4)	1709.86	1.11	0.14	7.83	4.99E-15	9.12E-14
	Rv0124	PE-PGRS family protein PE_PGRS2(PE_PGRS2)	293.23	1.03	0.18	5.55	2.85E-08	1.61E-07
**conserved hypotheticals**	Rv1954c	hypothetical protein(Rv1954c)	21268.21	3.18	0.25	12.83	1.06E-37	4.67E-35
	Rv1685c	hypothetical protein(Rv1685c)	1523.29	3.10	0.23	13.58	5.63E-42	3.19E-39
	Rv2616	hypothetical protein(Rv2616)	4085.24	2.32	0.28	8.21	2.20E-16	5.04E-15
	Rv0976c	hypothetical protein(Rv0976c)	2808.58	2.29	0.24	9.60	8.04E-22	4.43E-20
	Rv3202a	hypothetical protein(Rv3202a)	626.09	2.14	0.23	9.41	4.94E-21	2.45E-19
	Rv2641	cadmium inducible protein CadI(cadI)	41898.69	2.06	0.31	6.65	2.96E-11	2.76E-10
	Rv3122	hypothetical protein(Rv3122)	2382.93	2.02	0.30	6.81	1.01E-11	1.02E-10
	Rv1462	hypothetical protein(Rv1462)	15631.14	1.94	0.23	8.49	2.14E-17	6.01E-16
	Rv0968	hypothetical protein(Rv0968)	8093.05	1.93	0.25	7.86	3.95E-15	7.29E-14
	Rv3123	hypothetical protein(Rv3123)	1785.99	1.91	0.26	7.27	3.56E-13	4.54E-12
	Rv2052c	hypothetical protein(Rv2052c)	23659.26	1.90	0.33	5.71	1.13E-08	6.97E-08
	Rv2614A	hypothetical protein(Rv2614A)	467.94	1.86	0.24	7.72	1.13E-14	1.94E-13
	Rv2086	hypothetical protein(Rv2086)	266.72	1.84	0.25	7.31	2.65E-13	3.46E-12
	Rv2159c	hypothetical protein(Rv2159c)	4177.31	1.82	0.23	8.00	1.20E-15	2.41E-14

	Rv2744c	hypothetical protein(35kd_ag)	24438.40	1.80	0.21	8.73	2.44E-18	7.87E-17
	Rv3288c	hypothetical protein(usfY)	6340.60	1.67	0.20	8.52	1.63E-17	4.65E-16
	Rv2558	hypothetical protein(Rv2558)	9581.46	1.67	0.18	9.37	7.23E-21	3.50E-19
	Rv1894c	hypothetical protein(Rv1894c)	5548.95	1.67	0.24	7.05	1.79E-12	2.02E-11
	Rv1461	hypothetical protein(Rv1461)	28202.79	1.66	0.18	9.24	2.36E-20	1.05E-18
	Rv3205c	hypothetical protein(Rv3205c)	8498.05	1.61	0.18	8.80	1.35E-18	4.58E-17
	Rv1993c	hypothetical protein(Rv1993c)	1344.36	1.56	0.26	6.01	1.90E-09	1.33E-08
	Rv3836	hypothetical protein(Rv3836)	2280.28	1.50	0.24	6.36	1.96E-10	1.57E-09
	Rv1670	hypothetical protein(Rv1670)	210.52	1.48	0.26	5.73	9.85E-09	6.16E-08
	Rv1466	hypothetical protein(Rv1466)	1862.02	1.47	0.18	8.02	1.03E-15	2.14E-14
	Rv1684	hypothetical protein(Rv1684)	234.88	1.47	0.17	8.58	9.20E-18	2.74E-16
	Rv3627c	hypothetical protein(Rv3627c)	5529.60	1.45	0.10	15.25	1.70E-52	1.69E-49
	Rv3863	hypothetical protein(Rv3863)	5710.78	1.44	0.23	6.41	1.42E-10	1.17E-09
	Rv2743c	hypothetical protein(Rv2743c)	8617.87	1.43	0.22	6.54	6.34E-11	5.55E-10
	Rv1225c	hypothetical protein(Rv1225c)	11237.53	1.43	0.27	5.27	1.39E-07	6.82E-07
	Rv0628c	hypothetical protein(Rv0628c)	4679.73	1.42	0.14	9.82	9.26E-23	5.57E-21
	Rv1958c	hypothetical protein(Rv1958c)	751.11	1.40	0.17	8.01	1.19E-15	2.40E-14
	Rv0142	hypothetical protein(Rv0142)	3019.73	1.39	0.25	5.58	2.45E-08	1.39E-07
	Rv0250c	hypothetical protein(Rv0250c)	6659.42	1.38	0.19	7.20	6.24E-13	7.57E-12
	Rv2619c	hypothetical protein(Rv2619c)	1269.28	1.37	0.22	6.22	4.91E-10	3.74E-09
	Rv2372c	rRNA small subunit methyltransferase E(Rv2372c)	5799.14	1.36	0.24	5.64	1.73E-08	1.02E-07
	Rv0311	hypothetical protein(Rv0311)	608.29	1.36	0.23	5.84	5.12E-09	3.35E-08
	Rv2728c	hypothetical protein(Rv2728c)	934.52	1.34	0.19	6.94	3.81E-12	4.08E-11
	Rv2559c	hypothetical protein(Rv2559c)	2284.58	1.34	0.12	11.26	2.11E-29	3.22E-27
	Rv2557	hypothetical protein(Rv2557)	5268.57	1.33	0.17	7.74	1.00E-14	1.74E-13
	Rv1831	hypothetical protein(Rv1831)	3924.62	1.31	0.14	9.55	1.36E-21	7.10E-20
	Rv2618	hypothetical protein(Rv2618)	2280.35	1.30	0.26	4.99	6.11E-07	2.72E-06
	Rv0325	hypothetical protein(Rv0325)	117.02	1.29	0.17	7.46	8.71E-14	1.28E-12
	Rv0063a	hypothetical protein(Rv0063a)	360.07	1.27	0.16	8.09	5.82E-16	1.25E-14
	Rv1950c	hypothetical protein(Rv1950c)	175.49	1.27	0.39	3.23	1.24E-03	2.86E-03

	Rv2644c	hypothetical protein(Rv2644c)	550.63	1.26	0.22	5.75	8.94E-09	5.63E-08
	Rv0123	hypothetical protein(Rv0123)	237.23	1.26	0.20	6.17	6.81E-10	5.10E-09
	Rv0762c	hypothetical protein(Rv0762c)	1328.50	1.25	0.25	5.04	4.55E-07	2.07E-06
	Rv3188	hypothetical protein(Rv3188)	373.69	1.24	0.21	5.92	3.26E-09	2.18E-08
	Rv2570	hypothetical protein(Rv2570)	215.65	1.21	0.21	5.72	1.06E-08	6.56E-08
	Rv0836c	hypothetical protein(Rv0836c)	1290.39	1.20	0.26	4.64	3.54E-06	1.38E-05
	Rv0326	hypothetical protein(Rv0326)	338.91	1.20	0.19	6.33	2.47E-10	1.96E-09
	Rv2323c	hypothetical protein(Rv2323c)	603.70	1.20	0.16	7.71	1.31E-14	2.21E-13
	Rv3189	hypothetical protein(Rv3189)	613.41	1.20	0.16	7.56	4.11E-14	6.35E-13
	Rv3626c	hypothetical protein(Rv3626c)	2225.02	1.18	0.11	10.39	2.89E-25	2.61E-23
	Rv1084	hypothetical protein(Rv1084)	3528.51	1.17	0.14	8.15	3.68E-16	8.29E-15
	Rv0376c	hypothetical protein(Rv0376c)	571.97	1.16	0.17	6.86	7.05E-12	7.17E-11
	Rv1706A	hypothetical protein(Rv1706A)	970.99	1.16	0.25	4.58	4.68E-06	1.77E-05
	Rv1717	hypothetical protein(Rv1717)	257.67	1.15	0.27	4.23	2.36E-05	7.70E-05
	Rv0898c	hypothetical protein(Rv0898c)	842.44	1.13	0.21	5.39	7.08E-08	3.72E-07
	Rv2742c	hypothetical protein(Rv2742c)	3504.68	1.13	0.19	5.82	5.85E-09	3.78E-08
	rv0790c	hypothetical protein(Rv0790c)	4836.33	1.12	0.26	4.34	1.39E-05	4.77E-05
	Rv0574c	hypothetical protein(Rv0574c)	2040.14	1.11	0.24	4.61	4.05E-06	1.56E-05
	Rv2647	hypothetical protein(Rv2647)	557.07	1.09	0.46	2.38	1.75E-02	3.06E-02
	Rv0874c	hypothetical protein(Rv0874c)	2185.03	1.09	0.12	9.02	1.83E-19	7.27E-18
	Rv3421c	hypothetical protein(Rv3421c)	1458.50	1.08	0.19	5.55	2.88E-08	1.62E-07
	Rv1429	hypothetical protein(Rv1429)	482.68	1.06	0.13	8.19	2.65E-16	6.04E-15
	Rv2897c	hypothetical protein(Rv2897c)	487.73	1.06	0.23	4.61	3.99E-06	1.53E-05
	Rv3222c	hypothetical protein(Rv3222c)	4129.54	1.05	0.19	5.58	2.46E-08	1.39E-07
	Rv0372c	hypothetical protein(Rv0372c)	114.00	1.05	0.25	4.24	2.28E-05	7.48E-05
	Rv0368c	hypothetical protein(Rv0368c)	209.70	1.05	0.21	4.93	8.09E-07	3.51E-06
	Rv1716	hypothetical protein(Rv1716)	695.96	1.02	0.24	4.24	2.21E-05	7.27E-05
**insertion seqs and phages**	Rv1576c	phage capsid protein(Rv1576c)	6762.00	2.25	0.49	4.61	4.07E-06	1.56E-05
	Rv1575	phage protein(Rv1575)	307.17	2.06	0.37	5.60	2.19E-08	1.26E-07
	Rv1577c	phage prohead protease(Rv1577c)	4821.80	1.91	0.47	4.09	4.22E-05	1.32E-04

	Rv1578c	phage protein(Rv1578c)	690.14	1.74	0.32	5.35	8.57E-08	4.42E-07
	Rv1574	phage protein(Rv1574)	170.78	1.57	0.35	4.49	7.08E-06	2.58E-05
	Rv2659c	prophage integrase(Rv2659c)	1513.75	1.55	0.41	3.74	1.85E-04	5.04E-04
	Rv2812	transposase(Rv2812)	115.98	1.50	0.23	6.42	1.35E-10	1.12E-09
	Rv2651c	prophage protease(Rv2651c)	4161.49	1.41	0.51	2.76	5.81E-03	1.14E-02
	Rv2658c	prophage protein(Rv2658c)	557.36	1.27	0.34	3.76	1.69E-04	4.66E-04
	Rv1586c	phage integrase(Rv1586c)	5878.88	1.23	0.45	2.72	6.44E-03	1.26E-02
	Rv2646	integrase(Rv2646)	770.89	1.18	0.43	2.71	6.82E-03	1.32E-02
	Rv1585c	phage protein(Rv1585c)	2089.19	1.13	0.41	2.77	5.61E-03	1.11E-02
	Rv1583c	phage protein(Rv1583c)	759.22	0.94	0.36	2.57	1.01E-02	1.87E-02
	Rv2653c	toxin(Rv2653c)	65.65	0.94	0.36	2.60	9.20E-03	1.73E-02

(II) Significantly downregulated genes (L2Fc <-1, adj. p-value < 0.05)

Functional Category	Locus Tags	Genes	baseMean	L2Fc	lfcSE	stat	pvalue	padj
cell wall and cell processes	Rv1980c	immunogenic protein Mpt64(mpt64)	2086.64	-1.02	0.16	-6.44	1.16E-10	9.70E-10
	Rv1508c	membrane protein(Rv1508c)	1330.04	-1.02	0.17	-5.90	3.62E-09	2.41E-08
	Rv3795	arabinosyltransferase B(embB)	3783.87	-1.03	0.34	-3.01	2.64E-03	5.67E-03
	Rv0732	preprotein translocase SecY(secY)	1311.03	-1.04	0.16	-6.62	3.60E-11	3.30E-10
	Rv2329c	nitrate/nitrite transporter(narK1)	757.96	-1.04	0.28	-3.74	1.83E-04	5.02E-04
	Rv2965c	phosphopantetheine adenylyltransferase(kdtB)	335.80	-1.05	0.15	-7.05	1.78E-12	2.01E-11
	Rv3789	GtrA family protein(Rv3789)	162.96	-1.05	0.20	-5.32	1.04E-07	5.24E-07
	Rv0010c	membrane protein(Rv0010c)	1236.28	-1.05	0.15	-7.10	1.23E-12	1.43E-11
	Rv3278c	transmembrane protein(Rv3278c)	486.70	-1.05	0.20	-5.27	1.36E-07	6.69E-07
	Rv0128	transmembrane protein(Rv0128)	275.99	-1.06	0.19	-5.59	2.31E-08	1.32E-07
	Rv0461	transmembrane protein(Rv0461)	510.45	-1.06	0.15	-7.03	2.06E-12	2.29E-11
	Rv2127	L-asparagine permease(ansP1)	2403.84	-1.07	0.17	-6.46	1.04E-10	8.85E-10
	Rv0011c	cell division protein CrgA(Rv0011c)	787.58	-1.08	0.15	-7.20	5.89E-13	7.19E-12
	Rv0431	tuberculin-like peptide(Rv0431)	473.04	-1.08	0.16	-6.61	3.73E-11	3.39E-10
	Rv1197	ESAT-6 like protein EsxK(esxK)	3976.73	-1.09	0.22	-4.99	6.03E-07	2.70E-06

	Rv3620c	ESAT-6 like protein EsxW(esxW)	583.28	-1.09	0.21	-5.18	2.17E-07	1.03E-06
	Rv0403c	membrane protein MmpS1(mmpS1)	89.44	-1.13	0.14	-7.98	1.48E-15	2.93E-14
	Rv0178	Mce associated membrane protein(Rv0178)	1714.54	-1.14	0.11	-9.92	3.30E-23	2.11E-21
	Rv3004	low molecular weight protein antigen 6(cfp6)	511.14	-1.15	0.15	-7.64	2.19E-14	3.49E-13
	rv3312A	pilin(Rv3312A)	211.51	-1.16	0.25	-4.58	4.60E-06	1.74E-05
	Rv2128	transmembrane protein(Rv2128)	314.68	-1.17	0.16	-7.52	5.50E-14	8.30E-13
	Rv0236A	hypothetical protein(Rv0236A)	783.41	-1.20	0.14	-8.41	4.20E-17	1.10E-15
	Rv1857	molybdate ABC transporter substrate-binding lipoprotein ModA(modA)	815.83	-1.20	0.25	-4.89	1.02E-06	4.29E-06
	Rv0173	Mce family lipoprotein LprK(lprK)	2525.29	-1.22	0.23	-5.35	8.60E-08	4.43E-07
	Rv1614	prolipoprotein diacylglyceryl transferase(lgt)	3121.37	-1.24	0.18	-6.99	2.77E-12	3.00E-11
	Rv0713	transmembrane protein(Rv0713)	727.62	-1.24	0.13	-9.37	7.45E-21	3.56E-19
	Rv0114	D-glycero-alpha-D-manno-heptose-1,7-bisphosphate 7-phosphatase(gmhB)	140.12	-1.25	0.16	-8.03	9.60E-16	2.01E-14
	Rv2147c	cell division protein SepF(Rv2147c)	2414.56	-1.26	0.17	-7.44	9.95E-14	1.44E-12
	Rv2456c	MFS-type transporter(Rv2456c)	1265.13	-1.26	0.14	-9.28	1.67E-20	7.59E-19
	Rv1371	membrane protein(Rv1371)	284.15	-1.27	0.24	-5.34	9.42E-08	4.80E-07
	Rv3823c	integral membrane transport protein MmpL8(mmpL8)	4999.52	-1.28	0.14	-9.03	1.73E-19	6.93E-18
	Rv0113	phosphoheptose isomerase(gmhA)	213.18	-1.31	0.17	-7.65	2.01E-14	3.27E-13
	Rv1183	transmembrane transport protein MmpL10(mmpL10)	3367.62	-1.33	0.18	-7.20	5.83E-13	7.14E-12
	Rv1690	lipoprotein LprJ(lprJ)	140.99	-1.39	0.18	-7.71	1.29E-14	2.19E-13
	Rv1440	protein-export membrane protein SecG(secG)	317.56	-1.41	0.15	-9.33	1.11E-20	5.18E-19
	Rv1698	copper transporter MctB(mctB)	917.39	-1.45	0.20	-7.39	1.44E-13	2.01E-12
	Rv0933	phosphate ABC transporter ATP-binding protein PstB(pstB)	789.33	-1.47	0.21	-7.10	1.29E-12	1.49E-11
	Rv3921c	membrane protein insertase YidC(Rv3921c)	3719.24	-1.52	0.20	-7.65	2.07E-14	3.36E-13
	Rv0112	GDP-mannose 4,6-dehydratase(gca)	300.91	-1.65	0.20	-8.12	4.83E-16	1.05E-14
	Rv1987	chitinase(Rv1987)	817.26	-1.81	0.23	-8.01	1.13E-15	2.31E-14
	Rv1884c	resuscitation-promoting factor RpfC(rpfC)	2845.12	-1.88	0.27	-6.93	4.17E-12	4.40E-11
**intermediary metabolism, and respiration**	Rv1613	tryptophan synthase subunit alpha(trpA)	2130.97	-1.00	0.19	-5.19	2.07E-07	9.89E-07
	Rv3145	NADH-quinone oxidoreductase subunit A(nuoA)	1197.04	-1.00	0.19	-5.17	2.34E-07	1.10E-06
	Rv2993c	2-hydroxyhepta-2,4-diene-1,7-dioate isomerase(Rv2993c)	719.37	-1.02	0.15	-6.86	6.88E-12	7.01E-11
	Rv2277c	glycerolphosphodiesterase(Rv2277c)	198.97	-1.03	0.17	-5.90	3.66E-09	2.42E-08

	rv0636	(3R)-hydroxyacyl-ACP dehydratase subunit HadB(hadB)	871.99	-1.03	0.14	-7.11	1.18E-12	1.38E-11
	Rv0694	mycofactocin system heme/flavin oxidoreductase MftD(Rv0694)	5304.24	-1.05	0.19	-5.42	6.04E-08	3.21E-07
	Rv2338c	molybdopterin biosynthesis protein MoeW(moeW)	313.93	-1.07	0.24	-4.50	6.84E-06	2.50E-05
	Rv3232c	polyphosphate kinase(ppk2)	573.06	-1.07	0.13	-8.25	1.61E-16	3.77E-15
	Rv3322c	methyltransferase(Rv3322c)	670.53	-1.08	0.37	-2.90	3.70E-03	7.65E-03
	Rv3155	NADH-quinone oxidoreductase subunit K(nuoK)	255.13	-1.10	0.25	-4.34	1.45E-05	4.93E-05
	Rv3316	succinate dehydrogenase cytochrome B-556 subunit(sdhC)	130.83	-1.12	0.17	-6.60	4.08E-11	3.67E-10
	Rv3465	dTDP-4-dehydrorhamnose 3,5-epimerase(rmlC)	778.74	-1.14	0.17	-6.86	6.71E-12	6.86E-11
	Rv2007c	ferredoxin(fdxA)	6550.99	-1.14	0.29	-3.88	1.05E-04	3.06E-04
	Rv1611	indole-3-glycerol phosphate synthase(trpC)	2686.76	-1.15	0.17	-6.62	3.69E-11	3.37E-10
	Rv3323c	MoaD-MoaE fusion protein MoaX(moaX)	661.41	-1.16	0.41	-2.81	5.00E-03	1.00E-02
	Rv2074	pyridoxamine 5'-phosphate oxidase(Rv2074)	1164.91	-1.16	0.17	-6.89	5.47E-12	5.67E-11
	Rv3727	oxidoreductase(Rv3727)	1112.82	-1.18	0.20	-5.96	2.52E-09	1.74E-08
	Rv3464	dTDP-glucose 4,6-dehydratase(rmlB)	2103.60	-1.18	0.14	-8.41	4.21E-17	1.10E-15
	Rv3378c	diterpene synthase(Rv3378c)	199.88	-1.19	0.24	-4.91	8.88E-07	3.83E-06
	Rv0691A	mycofactocin precursor(Rv0691A)	168.42	-1.20	0.27	-4.52	6.24E-06	2.29E-05
	Rv0948c	chorismate mutase(Rv0948c)	479.90	-1.20	0.17	-7.25	4.24E-13	5.36E-12
	Rv1882c	short-chain type dehydrogenase/reductase(Rv1882c)	961.50	-1.20	0.14	-8.53	1.51E-17	4.33E-16
	Rv0500	pyrroline-5-carboxylate reductase(proC)	456.46	-1.21	0.14	-8.48	2.17E-17	6.01E-16
	rv2959c	rhamnosyl O-methyltransferase(Rv2959c)	598.90	-1.22	0.19	-6.55	5.69E-11	5.02E-10
	Rv1612	tryptophan synthase subunit beta(trpB)	2004.76	-1.24	0.23	-5.47	4.49E-08	2.45E-07
	Rv2457c	ATP-dependent CLP protease ATP-binding subunit ClpX(clpX)	9060.33	-1.28	0.17	-7.53	4.95E-14	7.55E-13
	Rv2951c	phthiodiolone/phenolphthiodiolone dimycocerosates ketoreductase(Rv2951c)	1218.82	-1.29	0.19	-6.94	4.00E-12	4.25E-11
	Rv0111	acyltransferase(Rv0111)	1470.79	-1.30	0.23	-5.75	8.74E-09	5.51E-08
	Rv2276	cytochrome P450 Cyp121(cyp121)	394.69	-1.37	0.23	-5.94	2.85E-09	1.95E-08
	Rv3377c	type B diterpene cyclase(Rv3377c)	1034.07	-1.40	0.19	-7.26	3.87E-13	4.90E-12
	Rv2952	phthiotriol/phenolphthiotriol dimycocerosates methyltransferase(Rv2952)	646.49	-1.42	0.18	-7.74	1.02E-14	1.76E-13
	Rv2949c	chorismate pyruvate-lyase(Rv2949c)	571.06	-1.43	0.21	-6.74	1.59E-11	1.56E-10
	Rv1360	oxidoreductase(Rv1360)	1175.95	-1.43	0.20	-7.08	1.49E-12	1.71E-11
	Rv3324c	cyclic pyranopterin monophosphate synthase accessory protein(moaC3)	566.13	-1.43	0.46	-3.13	1.76E-03	3.91E-03

	Rv0635	(3R)-hydroxyacyl-ACP dehydratase subunit HadA(hadA)	597.49	-1.44	0.16	-8.83	1.06E-18	3.67E-17
	Rv2350c	membrane-associated phospholipase B(plcB)	798.47	-1.44	0.23	-6.20	5.80E-10	4.37E-09
	Rv0637	(3R)-hydroxyacyl-ACP dehydratase subunit HadC(hadC)	1182.31	-1.46	0.16	-9.27	1.91E-20	8.63E-19
	Rv2957	PGL/p-HBAD biosynthesis glycosyltransferase(Rv2957)	777.12	-1.90	0.17	-11.45	2.37E-30	4.09E-28
**lipid metabolism**	Rv2950c	long-chain-fatty-acid--AMP ligase FadD29(fadD29)	1979.66	-1.05	0.17	-6.02	1.71E-09	1.21E-08
	Rv3791	decaprenylphosphoryl-D-2-keto erythropentose reductase(dprE2)	420.01	-1.07	0.21	-5.12	2.98E-07	1.39E-06
	Rv3790	decaprenylphosphoryl-beta-D-ribose oxidase(dprE1)	487.28	-1.08	0.21	-5.19	2.06E-07	9.82E-07
	Rv0404	long-chain-fatty-acid--AMP ligase FadD30(fadD30)	601.79	-1.08	0.17	-6.45	1.10E-10	9.29E-10
	rv2289	CDP-diacylglycerol pyrophosphatase(cdh)	298.78	-1.08	0.22	-5.00	5.79E-07	2.59E-06
	Rv0644c	cyclopropane mycolic acid synthase CmaA(mmaA2)	509.55	-1.09	0.15	-7.15	8.56E-13	1.03E-11
	rv1182	acyltransferase papA3(papA3)	1749.40	-1.17	0.19	-6.05	1.44E-09	1.03E-08
	Rv1185c	fatty-acid--CoA ligase FadD21(fadD21)	3466.89	-1.19	0.20	-6.05	1.47E-09	1.05E-08
	Rv0503c	cyclopropane mycolic acid synthase(cmaA2)	1459.97	-1.26	0.15	-8.65	5.33E-18	1.63E-16
	Rv3826	long-chain-fatty-acid--CoA ligase FadD23(fadD23)	853.66	-1.33	0.15	-8.77	1.74E-18	5.83E-17
	Rv3392c	cyclopropane mycolic acid synthase CmaA(cmaA1)	251.48	-1.34	0.19	-7.20	6.17E-13	7.50E-12
	Rv0643c	methoxy mycolic acid synthase MmaA3(mmaA3)	829.03	-1.45	0.17	-8.32	8.66E-17	2.11E-15
	Rv2953	trans-acting enoyl reductase(Rv2953)	1003.16	-1.46	0.21	-7.01	2.39E-12	2.60E-11
	Rv0166	fatty-acid--CoA ligase FadD5(fadD5)	1998.28	-1.50	0.25	-5.92	3.15E-09	2.12E-08
	Rv3824c	acyltransferase(papA1)	1606.29	-1.51	0.21	-7.13	9.88E-13	1.17E-11
**virulence, detoxification and adaptation**	Rv1477	peptidoglycan endopeptidase RipA(ripA)	3315.28	-1.05	0.20	-5.33	9.56E-08	4.87E-07
	Rv0167	membrane protein(yrbE1A)	2437.49	-1.12	0.21	-5.23	1.67E-07	8.12E-07
	Rv3320c	ribonuclease VapC44(vapC44)	482.88	-1.17	0.20	-5.78	7.62E-09	4.89E-08
	Rv2428	alkyl hydroperoxide reductase subunit AhpC(ahpC)	2121.35	-1.18	0.48	-2.44	1.48E-02	2.64E-02
	Rv0909	antitoxin(Rv0909)	237.54	-1.23	0.17	-7.23	4.84E-13	6.06E-12
	Rv0172	Mce family protein Mce1D(mce1D)	4243.22	-1.35	0.22	-6.28	3.32E-10	2.58E-09
	Rv0169	Mce family protein Mce1A(mce1A)	2585.58	-1.55	0.21	-7.35	1.98E-13	2.67E-12
	Rv0170	Mce family protein Mce1B(mce1B)	2110.66	-1.56	0.20	-7.99	1.30E-15	2.61E-14
	Rv3321c	antitoxin VapB44(vapB44)	142.07	-1.56	0.29	-5.45	4.95E-08	2.66E-07
	Rv0168	membrane protein(yrbE1B)	1147.87	-1.62	0.22	-7.50	6.60E-14	9.85E-13
	Rv3922c	membrane protein insertion efficiency factor(Rv3922c)	601.67	-1.66	0.21	-7.96	1.76E-15	3.45E-14

**regulatory proteins**	Rv3260c	transcriptional regulator WhiB2(whiB2)	1584.33	-1.05	0.25	-4.22	2.39E-05	7.80E-05
	Rv3058c	TetR family transcriptional regulator(Rv3058c)	806.40	-1.08	0.16	-6.87	6.51E-12	6.67E-11
	Rv0891c	hypothetical protein(Rv0891c)	337.71	-1.23	0.27	-4.61	3.95E-06	1.52E-05
	Rv0144	transcriptional regulator(Rv0144)	2180.82	-1.33	0.19	-7.04	1.87E-12	2.09E-11
	Rv0165c	transcriptional regulator Mce1R(mce1R)	1090.80	-1.41	0.16	-9.11	8.06E-20	3.37E-18
**information pathways**	Rv0429c	polypeptide deformylase(def)	266.90	-1.01	0.16	-6.20	5.56E-10	4.20E-09
**stable rnas**	thrT	tRNA-Thr(thrT)	118.96	-1.02	0.23	-4.44	9.19E-06	3.27E-05
	Rv3675	membrane protein(Rv3675)	1454.32	-1.06	0.20	-5.29	1.24E-07	6.18E-07
	metT	tRNA-Met(metT)	99.51	-1.51	0.26	-5.75	8.74E-09	5.51E-08
	MTS2975	ncRNA(MTS2975)	96.33	-1.66	0.23	-7.13	9.76E-13	1.16E-11
**PE/PPE**	Rv2431c	PE family protein PE25(PE25)	1945.73	-1.06	0.19	-5.64	1.67E-08	9.89E-08
	Rv1196	PPE family protein PPE18(PPE18)	11926.53	-1.07	0.21	-5.23	1.71E-07	8.27E-07
	Rv1195	PE family protein PE13(PE13)	4268.93	-1.28	0.22	-5.90	3.57E-09	2.37E-08
	Rv3135	PPE family protein PPE50(PPE50)	1900.36	-1.34	0.22	-6.15	7.61E-10	5.62E-09
	Rv2353c	PPE family protein PPE39(PPE39)	898.81	-1.59	0.53	-2.98	2.91E-03	6.21E-03
**conserved hypotheticals**	Rv1948c	hypothetical protein(Rv1948c)	176.18	-1.01	0.20	-4.96	7.08E-07	3.10E-06
	Rv3376	phosphatase(Rv3376)	1259.64	-1.01	0.14	-7.09	1.33E-12	1.53E-11
	Rv0831c	hypothetical protein(Rv0831c)	2090.96	-1.01	0.16	-6.25	4.14E-10	3.18E-09
	Rv0053	30S ribosomal protein S6(rpsF)	678.01	-1.02	0.16	-6.21	5.17E-10	3.91E-09
	Rv2927c	hypothetical protein(Rv2927c)	2098.17	-1.03	0.17	-6.00	2.01E-09	1.40E-08
	Rv3210c	hypothetical protein(Rv3210c)	641.44	-1.03	0.14	-7.44	9.95E-14	1.44E-12
	Rv1508A	hypothetical protein(Rv1508A)	101.82	-1.03	0.24	-4.25	2.15E-05	7.11E-05
	Rv1815	hypothetical protein(Rv1815)	1924.19	-1.04	0.19	-5.48	4.21E-08	2.31E-07
	Rv0108c	hypothetical protein(Rv0108c)	2038.58	-1.05	0.20	-5.39	7.04E-08	3.70E-07
	Rv2331A	hypothetical protein(Rv2331A)	68.39	-1.06	0.27	-3.98	7.03E-05	2.11E-04
	Rv0706	50S ribosomal protein L22(rplV)	3325.76	-1.06	0.24	-4.39	1.12E-05	3.93E-05
	Rv3258c	hypothetical protein(Rv3258c)	1004.75	-1.06	0.19	-5.65	1.64E-08	9.74E-08
	Rv3528c	hypothetical protein(Rv3528c)	240.24	-1.06	0.19	-5.66	1.50E-08	9.00E-08
	rv3126c	hypothetical protein(Rv3126c)	84.06	-1.06	0.21	-4.98	6.39E-07	2.84E-06

	Rv0056	50S ribosomal protein L9(rplI)	745.84	-1.07	0.14	-7.47	8.08E-14	1.19E-12
	Rv0700	30S ribosomal protein S10(rpsJ)	4164.59	-1.07	0.19	-5.71	1.15E-08	7.04E-08
	Rv0138	hypothetical protein(Rv0138)	217.25	-1.07	0.14	-7.61	2.77E-14	4.35E-13
	Rv0177	Mce associated protein(Rv0177)	1363.62	-1.07	0.15	-6.94	3.85E-12	4.11E-11
	Rv0708	50S ribosomal protein L16(rplP)	2369.97	-1.08	0.24	-4.58	4.57E-06	1.74E-05
	Rv0433	carboxylate-amine ligase(Rv0433)	1140.48	-1.08	0.12	-8.66	4.80E-18	1.49E-16
	Rv0683	30S ribosomal protein S7(rpsG)	3288.11	-1.08	0.18	-5.93	3.07E-09	2.07E-08
	Rv1271c	hypothetical protein(Rv1271c)	418.37	-1.09	0.11	-10.03	1.11E-23	7.73E-22
	Rv3491	hypothetical protein(Rv3491)	1705.98	-1.09	0.14	-7.87	3.46E-15	6.51E-14
	Rv1116	hypothetical protein(Rv1116)	77.05	-1.09	0.19	-5.89	3.82E-09	2.53E-08
	Rv2237A	hypothetical protein(Rv2237A)	279.60	-1.10	0.18	-5.94	2.90E-09	1.98E-08
	Rv1513	hypothetical protein(Rv1513)	388.58	-1.10	0.15	-7.23	4.99E-13	6.20E-12
	Rv3714c	hypothetical protein(Rv3714c)	376.66	-1.11	0.11	-9.86	6.49E-23	3.96E-21
	Rv2525c	hypothetical protein(Rv2525c)	819.80	-1.11	0.20	-5.66	1.50E-08	8.99E-08
	Rv1433	hypothetical protein(Rv1433)	650.99	-1.11	0.18	-6.27	3.51E-10	2.73E-09
	Rv1365c	anti-sigma-F factor antagonist RsfA(rsfA)	174.84	-1.13	0.23	-4.93	8.09E-07	3.51E-06
	Rv3924c	50S ribosomal protein L34(rpmH)	271.99	-1.14	0.20	-5.62	1.86E-08	1.09E-07
	Rv2955c	hypothetical protein(Rv2955c)	529.09	-1.14	0.14	-8.02	1.02E-15	2.12E-14
	Rv2331	hypothetical protein(Rv2331)	83.71	-1.15	0.30	-3.82	1.33E-04	3.75E-04
	Rv3811	hypothetical protein(Rv3811)	2855.51	-1.15	0.14	-8.37	5.74E-17	1.47E-15
	Rv0707	30S ribosomal protein S3(rpsC)	4545.96	-1.15	0.22	-5.28	1.32E-07	6.54E-07
	Rv0055	30S ribosomal protein S18(rpsR1)	286.35	-1.16	0.13	-8.86	8.08E-19	2.91E-17
	Rv1919c	hypothetical protein(Rv1919c)	359.21	-1.17	0.18	-6.61	3.85E-11	3.48E-10
	Rv1547	DNA polymerase III subunit alpha(dnaE1)	3731.02	-1.19	0.12	-10.26	1.10E-24	9.07E-23
	Rv2387	hypothetical protein(Rv2387)	1107.30	-1.20	0.15	-7.95	1.88E-15	3.67E-14
	Rv0705	30S ribosomal protein S19(rpsS)	2649.95	-1.20	0.21	-5.64	1.71E-08	1.01E-07
	Rv3920c	hypothetical protein(Rv3920c)	2749.92	-1.20	0.16	-7.28	3.24E-13	4.16E-12
	Rv0718	30S ribosomal protein S8(rpsH)	1994.08	-1.20	0.18	-6.77	1.25E-11	1.25E-10
	Rv0704	50S ribosomal protein L2(rplB)	6348.85	-1.21	0.22	-5.57	2.51E-08	1.42E-07
	Rv1184c	hypothetical protein(Rv1184c)	1391.94	-1.21	0.22	-5.58	2.44E-08	1.39E-07

	Rv1830	HTH-type transcriptional regulator(Rv1830)	1718.89	-1.21	0.16	-7.44	1.04E-13	1.50E-12
	Rv2275	cyclo(L-tyrosyl-L-tyrosyl) synthase(Rv2275)	224.64	-1.21	0.28	-4.35	1.35E-05	4.64E-05
	Rv3796	hypothetical protein(Rv3796)	572.88	-1.21	0.25	-4.87	1.13E-06	4.73E-06
	Rv0057	hypothetical protein(Rv0057)	573.48	-1.23	0.17	-7.23	4.97E-13	6.20E-12
	Rv2189c	hypothetical protein(Rv2189c)	848.81	-1.24	0.22	-5.67	1.42E-08	8.57E-08
	Rv2069	ECF RNA polymerase sigma factor SigC(sigC)	406.23	-1.24	0.14	-8.84	9.53E-19	3.37E-17
	Rv3651	hypothetical protein(Rv3651)	783.71	-1.25	0.16	-7.64	2.17E-14	3.48E-13
	Rv2271	hypothetical protein(Rv2271)	225.68	-1.25	0.16	-7.74	1.01E-14	1.74E-13
	Rv1904	hypothetical protein(Rv1904)	1315.15	-1.26	0.16	-7.70	1.38E-14	2.34E-13
	Rv3492c	Mce associated protein(Rv3492c)	420.25	-1.26	0.14	-9.01	2.00E-19	7.87E-18
	Rv0634A	hypothetical protein(Rv0634A)	421.77	-1.28	0.21	-6.23	4.57E-10	3.50E-09
	Rv1507A	hypothetical protein(Rv1507A)	306.90	-1.30	0.16	-7.88	3.35E-15	6.32E-14
	Rv2778c	hypothetical protein(Rv2778c)	454.09	-1.30	0.16	-8.37	5.69E-17	1.47E-15
	Rv0709	50S ribosomal protein L29(rpmC)	784.52	-1.30	0.25	-5.30	1.15E-07	5.74E-07
	Rv2067c	hypothetical protein(Rv2067c)	658.20	-1.31	0.16	-8.34	7.69E-17	1.92E-15
	Rv3005c	hypothetical protein(Rv3005c)	2514.00	-1.33	0.18	-7.54	4.72E-14	7.26E-13
	Rv3686c	hypothetical protein(Rv3686c)	638.63	-1.34	0.29	-4.57	4.96E-06	1.86E-05
	Rv0703	50S ribosomal protein L23(rplW)	1872.38	-1.35	0.20	-6.61	3.92E-11	3.54E-10
	Rv2954c	hypothetical protein(Rv2954c)	1449.30	-1.36	0.21	-6.58	4.84E-11	4.31E-10
	Rv3612c	hypothetical protein(Rv3612c)	837.23	-1.36	0.25	-5.50	3.82E-08	2.10E-07
	Rv3717	hypothetical protein(Rv3717)	755.82	-1.37	0.19	-7.32	2.39E-13	3.16E-12
	Rv3424c	hypothetical protein(Rv3424c)	1390.39	-1.38	0.17	-8.29	1.12E-16	2.72E-15
	Rv0164	hypothetical protein(TB18.5)	627.41	-1.39	0.16	-8.44	3.12E-17	8.31E-16
	Rv2257c	hypothetical protein(2257c)	490.31	-1.39	0.57	-2.45	1.44E-02	2.58E-02
	Rv2816c	CRISPR-associated endoribonuclease Cas2(Rv2816c)	233.06	-1.39	0.31	-4.43	9.43E-06	3.35E-05
	Rv3613c	hypothetical protein(Rv3613c)	826.72	-1.39	0.27	-5.22	1.79E-07	8.65E-07
	Rv1870c	hypothetical protein(Rv1870c)	3851.43	-1.41	0.19	-7.34	2.15E-13	2.87E-12
	Rv1506c	hypothetical protein(Rv1506c)	143.77	-1.41	0.20	-7.14	9.51E-13	1.14E-11
	Rv1505c	hypothetical protein(Rv1505c)	203.03	-1.42	0.16	-8.90	5.76E-19	2.12E-17
	Rv0717	30S ribosomal protein S14(rpsN1)	625.16	-1.46	0.26	-5.65	1.61E-08	9.61E-08

	Rv0710	30S ribosomal protein S17(rpsQ)	1028.58	-1.46	0.24	-6.06	1.33E-09	9.50E-09
	Rv0634B	50S ribosomal protein L33(rpmG2)	258.43	-1.47	0.18	-8.25	1.64E-16	3.82E-15
	Rv2076c	hypothetical protein(Rv2076c)	479.46	-1.48	0.22	-6.89	5.41E-12	5.61E-11
	Rv2336	hypothetical protein(Rv2336)	527.69	-1.49	0.22	-6.91	4.81E-12	5.02E-11
	Rv3923c	ribonuclease P protein component(rnpA)	805.87	-1.55	0.19	-8.29	1.17E-16	2.81E-15
	Rv2956	hypothetical protein(Rv2956)	806.55	-1.55	0.20	-7.69	1.46E-14	2.44E-13
	Rv3209	hypothetical protein(Rv3209)	1088.55	-1.61	0.18	-8.89	6.14E-19	2.23E-17
	Rv0430	hypothetical protein(Rv0430)	340.93	-1.64	0.25	-6.65	2.85E-11	2.66E-10
	Rv1697	hypothetical protein(Rv1697)	2098.24	-1.65	0.23	-7.31	2.61E-13	3.42E-12
	Rv2817c	CRISPR-associated endonuclease Cas1(Rv2817c)	995.05	-1.66	0.31	-5.31	1.07E-07	5.40E-07
	Rv1883c	hypothetical protein(Rv1883c)	2261.60	-1.70	0.22	-7.62	2.61E-14	4.12E-13
	Rv0001	chromosomal replication initiator protein DnaA(dnaA)	3212.45	-1.78	0.23	-7.84	4.45E-15	8.17E-14
	Rv1507c	hypothetical protein(Rv1507c)	280.32	-1.78	0.20	-9.04	1.61E-19	6.58E-18

Supplementary Table 3. Genes significantly differentially expressed (absolute Log2FoldChange (L2Fc)> 1, adj. p-value < 0.05) in H37Rv after 72 hours of bedaquiline exposure (3.75 µg/ml) compared to control (unexposed H37Rv).
(I) Significantly upregulated genes (L2Fc >1, adj. p-value < 0.05)

Functional Category	Locus Tags	Genes	baseMean	L2Fc	lfc SE	stat	pvalue	padj
**cell wall and cell processes**	Rv1687c	ABC transporter ATP-binding protein(Rv1687c)	3610.09	7.16	0.68	10.51	7.64E-26	3.03E-22
	Rv1686c	ABC transporter permease(Rv1686c)	2705.53	6.39	0.61	10.39	2.64E-25	5.24E-22
	Rv2873	cell surface lipoprotein(mpt83)	2416.64	3.92	1.19	3.28	1.02E-03	5.52E-03
	Rv2875	major secreted immunogenic protein Mpt70(mpt70)	3816.96	3.62	1.21	2.99	2.79E-03	1.20E-02
	Rv2876	transmembrane protein(Rv2876)	1025.75	3.15	0.61	5.14	2.73E-07	8.52E-06
	Rv0188	transmembrane protein(Rv0188)	9961.73	3.00	0.57	5.28	1.29E-07	4.67E-06
	Rv1226c	transmembrane protein(Rv1226c)	6262.94	2.60	0.63	4.14	3.40E-05	3.81E-04

	Rv1227c	transmembrane protein(Rv1227c)	2027.50	2.51	0.48	5.25	1.54E-07	5.36E-06
	Rv2690c	integral membrane protein(Rv2690c)	1116.23	2.47	0.34	7.28	3.31E-13	1.19E-10
	Rv2874	integral membrane C-type cytochrome biogenesis protein DipZ(dipZ)	1012.75	2.41	0.63	3.84	1.21E-04	1.04E-03
	Rv2025c	cation efflux system protein(Rv2025c)	1169.66	2.29	0.45	5.07	3.91E-07	1.13E-05
	Rv2877c	integral membrane protein(Rv2877c)	2413.30	2.12	0.40	5.35	8.96E-08	3.45E-06
	Rv2617c	transmembrane protein(Rv2617c)	3928.87	2.08	0.65	3.19	1.42E-03	7.14E-03
	Rv2691	TRK system potassium uptake protein CeoB(ceoB)	864.79	2.08	0.36	5.80	6.48E-09	4.29E-07
	Rv0985c	large-conductance ion mechanosensitive channel(mscL)	2492.86	2.05	0.43	4.75	2.01E-06	3.93E-05
	Rv2693c	integral membrane protein(Rv2693c)	1623.62	2.00	0.25	7.97	1.58E-15	7.86E-13
	Rv2963	integral membrane protein(Rv2963)	3986.15	2.00	0.59	3.37	7.50E-04	4.32E-03
	Rv0986	adhesion component ABC transporter ATP-binding protein(Rv0986)	593.84	2.00	0.51	3.94	8.08E-05	7.56E-04
	Rv1258c	multidrug-efflux transporter(Rv1258c)	696.96	1.95	0.64	3.04	2.35E-03	1.04E-02
	Rv1228	lipoprotein LpqX(lpqX)	2068.30	1.94	0.39	5.04	4.71E-07	1.30E-05
	Rv0847	lipoprotein LpqS(lpqS)	8314.80	1.89	0.64	2.95	3.17E-03	1.33E-02
	Rv2692	TRK system potassium uptake protein CeoC(ceoC)	982.65	1.88	0.30	6.18	6.32E-10	7.16E-08
	Rv1973	Mce associated membrane protein(Rv1973)	159.16	1.85	0.50	3.73	1.93E-04	1.49E-03
	Rv1224	Sec-independent protein translocase protein TatB(tatB)	7011.02	1.82	0.63	2.87	4.14E-03	1.64E-02
	Rv2643	arsenic-transport integral membrane protein ArsC(arsC)	8917.44	1.80	0.65	2.77	5.69E-03	2.10E-02
	Rv0969	copper-exporting ATPase(ctpV)	85241.84	1.78	0.70	2.56	1.05E-02	3.41E-02

	Rv1463	ABC transporter ATP-binding protein(Rv1463)	6972.26	1.78	0.51	3.47	5.25E-04	3.24E-03
	Rv2544	lipoprotein LppB(lppB)	105.59	1.76	0.72	2.46	1.38E-02	4.24E-02
	Rv3017c	ESAT-6 like protein EsxQ(esxQ)	214.41	1.75	0.55	3.16	1.55E-03	7.63E-03
	Rv2688c	antibiotic ABC transporter ATP-binding protein(Rv2688c)	1614.04	1.75	0.27	6.46	1.06E-10	1.61E-08
	Rv1992c	cation transporter ATPase G(ctpG)	18472.67	1.74	0.63	2.78	5.40E-03	2.03E-02
	Rv1230c	membrane protein(Rv1230c)	1774.24	1.71	0.30	5.74	9.72E-09	5.75E-07
	Rv2687c	antibiotic ABC transporter permease(Rv2687c)	408.17	1.71	0.29	5.91	3.34E-09	2.50E-07
	Rv0064	transmembrane protein(Rv0064)	8521.73	1.68	0.35	4.82	1.44E-06	3.06E-05
	Rv3016	lipoprotein LpqA(lpqA)	674.47	1.68	0.39	4.32	1.53E-05	2.01E-04
	Rv3447c	ESX-4 secretion system protein EccC4(eccC4)	216.61	1.67	0.48	3.52	4.28E-04	2.75E-03
	Rv0446c	transmembrane protein(Rv0446c)	540.52	1.66	0.28	6.01	1.88E-09	1.56E-07
	Rv0369c	membrane oxidoreductase(Rv0369c)	95.36	1.66	0.44	3.74	1.82E-04	1.43E-03
	Rv2834c	sn-glycerol-3-phosphate ABC transporter permease UgpE(ugpE)	239.61	1.57	0.53	2.98	2.84E-03	1.22E-02
	Rv3448	ESX-4 secretion system protein EccD4(eccD4)	152.44	1.57	0.48	3.25	1.14E-03	6.00E-03
	Rv2333c	multidrug resistance protein(stp)	888.48	1.55	0.40	3.92	8.92E-05	8.08E-04
	Rv1972	Mce associated membrane protein(Rv1972)	160.47	1.53	0.41	3.76	1.67E-04	1.34E-03
	Rv3065	multidrug resistance protein Mmr(mmr)	393.98	1.51	0.44	3.48	5.04E-04	3.13E-03
	Rv0538	membrane protein(Rv0538)	6871.74	1.51	0.41	3.67	2.38E-04	1.75E-03
	Rv1235	trehalose ABC transporter substrate-binding lipoprotein LpqY(lpqY)	1481.21	1.48	0.25	5.96	2.50E-09	1.99E-07

	Rv0194	multidrug ABC transporter ATPase/permease(Rv0194)	625.39	1.45	0.34	4.29	1.78E-05	2.25E-04
	Rv1986	amino acid transporter(Rv1986)	553.70	1.44	0.50	2.86	4.23E-03	1.66E-02
	Rv2698	transmembrane protein(Rv2698)	778.71	1.43	0.43	3.31	9.20E-04	5.04E-03
	Rv1819c	vitamin B12 transport ATP-binding protein BacA(bacA)	552.67	1.42	0.37	3.82	1.33E-04	1.12E-03
	Rv3437	transmembrane protein(Rv3437)	495.63	1.42	0.41	3.49	4.83E-04	3.02E-03
	Rv0347	membrane protein(Rv0347)	1193.62	1.41	0.37	3.80	1.43E-04	1.18E-03
	Rv1234	transmembrane protein(Rv1234)	1269.09	1.41	0.31	4.50	6.79E-06	1.03E-04
	Rv3445c	ESAT-6 like protein EsxU(esxU)	67.70	1.40	0.52	2.72	6.49E-03	2.33E-02
	Rv3289c	transmembrane protein(Rv3289c)	5817.79	1.40	0.45	3.08	2.04E-03	9.34E-03
	Rv1239c	magnesium and cobalt transport transmembrane protein CorA(corA)	385.36	1.37	0.41	3.36	7.88E-04	4.48E-03
	Rv2686c	antibiotic ABC transporter permease(Rv2686c)	411.77	1.35	0.27	5.07	3.90E-07	1.13E-05
	Rv0671	lipoprotein LpqP(lpqP)	1794.98	1.32	0.31	4.29	1.82E-05	2.28E-04
	Rv0261c	nitrate/nitrite transporter(narK3)	197.93	1.32	0.40	3.26	1.12E-03	5.91E-03
	Rv0246	integral membrane protein(Rv0246)	1362.73	1.28	0.28	4.53	5.77E-06	8.97E-05
	Rv1922	lipoprotein(Rv1922)	471.23	1.28	0.36	3.55	3.88E-04	2.57E-03
	Rv1072	transmembrane protein(Rv1072)	44494.87	1.28	0.35	3.66	2.49E-04	1.82E-03
	Rv1231c	membrane protein(Rv1231c)	448.95	1.28	0.27	4.73	2.23E-06	4.22E-05
	Rv3666c	dipeptide ABC transporter substrate-binding lipoprotein DppA(dppA)	724.08	1.27	0.47	2.71	6.64E-03	2.36E-02
	Rv0116c	L,D-transpeptidase LdtA(ldtA)	1596.36	1.25	0.42	2.97	3.00E-03	1.27E-02

	Rv1250	MFS-type drug transporter(Rv1250)	838.98	1.24	0.27	4.61	4.08E-06	6.91E-05
	Rv2833c	sn-glycerol-3-phosphate ABC transporter substrate-binding lipoprotein UgpB(ugpB)	325.43	1.24	0.50	2.45	1.42E-02	4.34E-02
	Rv2709	transmembrane protein(Rv2709)	1615.73	1.18	0.39	3.05	2.29E-03	1.02E-02
	Rv0987	adhesion component ABC transporter permease(Rv0987)	1110.53	1.17	0.41	2.85	4.43E-03	1.72E-02
	Rv0219	transmembrane protein(Rv0219)	89.08	1.14	0.46	2.46	1.39E-02	4.24E-02
	Rv1236	sugar ABC transporter permease SugA(sugA)	572.82	1.13	0.26	4.29	1.75E-05	2.24E-04
	Rv0037c	MFS-type transporter(Rv0037c)	1688.96	1.11	0.27	4.10	4.15E-05	4.45E-04
	Rv0593	Mce family lipoprotein LprL(lprL)	532.10	1.09	0.35	3.13	1.75E-03	8.36E-03
	Rv3450c	ESX-4 secretion system protein EccB4(eccB4)	592.51	1.07	0.36	2.98	2.93E-03	1.25E-02
	Rv0379	protein translocase subunit SecE(secE2)	167.02	1.07	0.36	2.97	2.97E-03	1.26E-02
	Rv1146	transmembrane transport protein(Rv1146)	629.83	1.07	0.28	3.78	1.60E-04	1.30E-03
	Rv1892	membrane protein(Rv1892)	525.91	1.07	0.42	2.57	1.01E-02	3.31E-02
	Rv0318c	integral membrane protein(Rv0318c)	159.60	1.07	0.32	3.33	8.57E-04	4.78E-03
	Rv3610c	zinc metalloprotease FtsH(ftsH)	12037.77	1.02	0.24	4.32	1.57E-05	2.04E-04
**intermediary metabolism and respiration**	Rv0374c	carbon monoxyde dehydrogenase small subunit(Rv0374c)	127.60	2.86	0.66	4.33	1.49E-05	1.98E-04
	Rv3203	lipase LipV(lipV)	2021.69	2.71	0.62	4.35	1.34E-05	1.81E-04
	Rv0375c	carbon monoxyde dehydrogenase medium subunit(Rv0375c)	241.50	2.67	0.53	4.99	6.05E-07	1.59E-05
	Rv0373c	carbon monoxyde dehydrogenase large subunit(Rv0373c)	573.84	2.55	0.50	5.12	2.98E-07	9.17E-06
	Rv1936	monooxygenase(Rv1936)	3787.61	2.51	0.75	3.33	8.82E-04	4.88E-03

	Rv3161<br>c	dioxygenase(Rv3161c)	10637.<br>34	2.4<br>1	0.6<br>9	3.5<br>0	4.60E<br>-04	2.92E<br>-03
	Rv0648	alpha-mannosidase(Rv0648)	1665.7<br>3	2.3<br>9	0.4<br>1	5.8<br>7	4.25E<br>-09	3.01E<br>-07
	Rv0560<br>c	benzoquinone methyltransferase(Rv0560c)	1141.6<br>2	2.3<br>2	0.5<br>1	4.5<br>8	4.58E<br>-06	7.60E<br>-05
	Rv1937	oxygenase(Rv1937)	4384.2<br>0	2.2<br>6	0.6<br>1	3.6<br>9	2.28E<br>-04	1.68E<br>-03
	Rv0249<br>c	succinate dehydrogenase membrane anchor subunit(Rv0249c)	4452.4<br>8	2.1<br>7	0.4<br>0	5.4<br>1	6.15E<br>-08	2.62E<br>-06
	Rv0983	serine protease PepD(pepD)	35226.<br>24	2.0<br>9	0.4<br>4	4.7<br>4	2.11E<br>-06	4.06E<br>-05
	Rv3502<br>c	3-oxoacyl-ACP reductase(Rv3502c)	2423.1<br>0	2.0<br>8	0.3<br>4	6.1<br>3	8.67E<br>-10	8.82E<br>-08
	Rv2667	ATP-dependent protease ATP-binding subunit ClpC(clpC2)	548.87	2.0<br>7	0.6<br>7	3.1<br>0	1.96E<br>-03	9.09E<br>-03
	Rv2280	probable dehydrogenase (Rv2280)	3736.6<br>3	2.0<br>3	0.4<br>4	4.6<br>3	3.62E<br>-06	6.42E<br>-05
	Rv0984	pterin-4-alpha-carbinolamine dehydratase(moaB2)	9874.4<br>5	2.0<br>2	0.4<br>2	4.7<br>9	1.67E<br>-06	3.41E<br>-05
	Rv2501<br>c	acetyl/propionyl-CoA carboxylase subuit alpha(accA1)	7427.9<br>9	2.0<br>1	0.4<br>9	4.1<br>2	3.76E<br>-05	4.09E<br>-04
	Rv3534<br>c	4-hydroxy-2-oxovalerate aldolase(hsaF)	3913.4<br>0	1.9<br>4	0.4<br>0	4.9<br>0	9.48E<br>-07	2.19E<br>-05
	Rv3503<br>c	ferredoxin FdxD(fdxD)	747.14	1.9<br>4	0.4<br>4	4.4<br>3	9.61E<br>-06	1.37E<br>-04
	Rv3206<br>c	adenylyltransferase/sulfurtransferase MoeZ(moeB1)	19511.<br>64	1.9<br>1	0.4<br>9	3.9<br>3	8.55E<br>-05	7.91E<br>-04
	Rv2497<br>c	3-methyl-2-oxobutanoate dehydrogenase subunit alpha(bkdA)	11839.<br>95	1.8<br>8	0.3<br>9	4.8<br>4	1.30E<br>-06	2.83E<br>-05
	Rv0848	cysteine synthase CysK(cysK2)	12056.<br>68	1.8<br>5	0.6<br>4	2.8<br>9	3.82E<br>-03	1.53E<br>-02
	Rv2713	pyridine nucleotide transhydrogenase(sthA)	2004.0<br>8	1.8<br>4	0.3<br>3	5.5<br>9	2.27E<br>-08	1.19E<br>-06
	Rv0539	dolichyl-phosphate sugar synthase(Rv0539)	4456.4<br>2	1.8<br>1	0.3<br>1	5.9<br>2	3.29E<br>-09	2.50E<br>-07
	Rv0846<br>c	oxidase(Rv0846c)	3623.8<br>6	1.8<br>1	0.5<br>7	3.2<br>0	1.36E<br>-03	6.89E<br>-03

	Rv2496c	3-methyl-2-oxobutanoate dehydrogenase subunit beta(bkdB)	7756.21	1.81	0.39	4.69	2.76E-06	5.07E-05
	Rv2499c	oxidase regulatory-like protein(Rv2499c)	1453.41	1.80	0.48	3.75	1.78E-04	1.41E-03
	Rv0370c	oxidoreductase(Rv0370c)	138.86	1.80	0.50	3.57	3.59E-04	2.40E-03
	Rv3535c	acetaldehyde dehydrogenase(hsaG)	3264.40	1.79	0.39	4.62	3.92E-06	6.73E-05
	Rv1263	amidase AmiB(amiB2)	455.28	1.75	0.40	4.35	1.38E-05	1.87E-04
	Rv1223	serine protease HtrA(htrA)	31198.89	1.74	0.55	3.17	1.51E-03	7.48E-03
	Rv1256c	cytochrome P450 Cyp130(cyp130)	1975.83	1.74	0.32	5.42	5.81E-08	2.56E-06
	Rv0753c	methylmalonate-semialdehyde dehydrogenase(mmsA)	10759.36	1.73	0.53	3.26	1.12E-03	5.91E-03
	Rv0077c	oxidoreductase(Rv0077c)	414.98	1.70	0.55	3.10	1.95E-03	9.09E-03
	Rv1464	cysteine desulfurase(csd)	11996.55	1.68	0.54	3.10	1.91E-03	8.93E-03
	Rv3215	isochorismate synthase(entC)	1156.88	1.68	0.34	4.91	9.07E-07	2.15E-05
	Rv2498c	citrate (pro-3S)-lyase subunit beta(citE)	1943.35	1.65	0.41	3.99	6.63E-05	6.38E-04
	Rv0186	beta-glucosidase BglS(bglS)	43068.79	1.65	0.50	3.32	9.09E-04	5.00E-03
	Rv2671	bifunctional diaminohydroxyphosphoribosylaminopyrimidine deaminase/5-amino-6-(5-phosphoribosylamino)uracil reductase(ribD)	357.89	1.63	0.35	4.62	3.86E-06	6.69E-05
	Rv3729	transferase(Rv3729)	3630.46	1.59	0.52	3.07	2.11E-03	9.53E-03
	Rv3290c	L-lysine-epsilon aminotransferase(lat)	64780.41	1.58	0.42	3.76	1.69E-04	1.35E-03
	Rv3536c	hydratase(hsaE)	3548.68	1.56	0.39	4.00	6.45E-05	6.28E-04
	Rv1050	oxidoreductase(Rv1050)	88.65	1.56	0.51	3.05	2.30E-03	1.02E-02
	Rv2465c	ribose-5-phosphate isomerase B(rpiB)	2226.79	1.55	0.53	2.95	3.19E-03	1.34E-02

	Rv1652	N-acetyl-gamma-glutamyl-phoshate reductase(argC)	702.33	1.54	0.49	3.15	1.64E-03	7.94E-03
	Rv3837c	phosphoglycerate mutase(Rv3837c)	8521.53	1.53	0.49	3.12	1.84E-03	8.70E-03
	Rv0565c	monooxygenase(Rv0565c)	766.80	1.52	0.39	3.94	8.22E-05	7.66E-04
	Rv2495c	branched-chain keto acid dehydrogenase E2 component(bkdC)	7508.40	1.52	0.40	3.78	1.56E-04	1.27E-03
	Rv2669	GCN5-like N-acetyltransferase(Rv2669)	153.90	1.51	0.52	2.89	3.84E-03	1.54E-02
	Rv0751c	3-hydroxyisobutyrate dehydrogenase(mmsB)	3637.47	1.51	0.52	2.91	3.59E-03	1.46E-02
	Rv1594	quinolinate synthetase A(nadA)	18057.93	1.50	0.47	3.16	1.56E-03	7.63E-03
	Rv2072c	precorrin-6Y C(5,15)-methyltransferase(cobL)	349.67	1.49	0.27	5.53	3.13E-08	1.53E-06
	Rv1373	glycolipid sulfotransferase(Rv1373)	776.72	1.48	0.38	3.87	1.08E-04	9.44E-04
	Rv0161	oxidoreductase(Rv0161)	300.90	1.47	0.29	5.01	5.40E-07	1.44E-05
	Rv0380c	RNA methyltransferase(Rv0380c)	885.14	1.46	0.40	3.64	2.70E-04	1.93E-03
	Rv0189c	dihydroxy-acid dehydratase(ilvD)	5698.96	1.45	0.27	5.31	1.09E-07	4.10E-06
	Rv1714	oxidoreductase(Rv1714)	903.82	1.44	0.58	2.48	1.32E-02	4.09E-02
	Rv1372	alpha-pyrone synthesis polyketide synthase-like protein(Rv1372)	389.19	1.44	0.42	3.46	5.46E-04	3.36E-03
	Rv0881	tRNA/rRNA methyltransferase(Rv0881)	367.96	1.43	0.43	3.30	9.69E-04	5.29E-03
	Rv1726	oxidoreductase(Rv1726)	279.35	1.41	0.32	4.47	7.99E-06	1.16E-04
	Rv2607	pyridoxine/pyridoxamine 5'-phosphate oxidase(pdxH)	120.70	1.40	0.48	2.90	3.72E-03	1.50E-02
	Rv0382c	orotate phosphoribosyltransferase(pyrE)	3226.56	1.39	0.48	2.90	3.77E-03	1.52E-02
	Rv1465	nitrogen fixation related protein(Rv1465)	2792.44	1.39	0.49	2.82	4.79E-03	1.83E-02

	Rv0162c	zinc-type alcohol dehydrogenase subunit E(adhE1)	901.28	1.38	0.52	2.65	8.05E-03	2.74E-02
	Rv0650	sugar kinase(Rv0650)	301.91	1.38	0.45	3.03	2.47E-03	1.08E-02
	Rv1229c	multiple resistance/pH adaptation protein(mrp)	4493.16	1.37	0.23	6.01	1.81E-09	1.53E-07
	Rv0940c	oxidoreductase(Rv0940c)	951.63	1.35	0.35	3.93	8.60E-05	7.91E-04
	Rv0266c	5-oxoprolinase OplA(oplA)	2421.60	1.35	0.29	4.61	4.00E-06	6.82E-05
	Rv0851c	short-chain type dehydrogenase/reductase(Rv0851c)	823.41	1.34	0.55	2.41	1.58E-02	4.72E-02
	Rv1653	bifunctional glutamate N-acetyltransferase/amino-acid acetyltransferase(argJ)	1982.94	1.34	0.41	3.30	9.76E-04	5.32E-03
	Rv0248c	succinate dehydrogenase flavoprotein subunit(Rv0248c)	6370.78	1.34	0.29	4.56	5.00E-06	8.12E-05
	Rv2073c	oxidoreductase(Rv2073c)	294.86	1.33	0.31	4.27	1.94E-05	2.38E-04
	Rv0247c	succinate dehydrogenase iron-sulfur subunit(Rv0247c)	1302.40	1.33	0.29	4.65	3.31E-06	5.96E-05
	Rv1654	acetylglutamate kinase(argB)	1387.37	1.32	0.48	2.74	6.12E-03	2.22E-02
	Rv0989c	polyprenyl-diphosphate synthase GrcC(grcC2)	1096.36	1.30	0.52	2.48	1.33E-02	4.10E-02
	Rv0132c	F420-dependent glucose-6-phosphate dehydrogenase(fgd2)	481.93	1.29	0.35	3.74	1.81E-04	1.42E-03
	Rv2201	asparagine synthetase(asnB)	2684.24	1.28	0.41	3.13	1.75E-03	8.38E-03
	Rv3449	membrane-anchored mycosin(mycP4)	360.76	1.27	0.31	4.05	5.11E-05	5.17E-04
	Rv3591c	hydrolase(Rv3591c)	133.19	1.27	0.33	3.81	1.37E-04	1.15E-03
	Rv3078	hydroxylaminobenzene mutase(hab)	166.29	1.27	0.38	3.37	7.61E-04	4.37E-03
	Rv3545c	steroid C26-monooxygenase(cyp125)	1726.00	1.26	0.47	2.64	8.18E-03	2.78E-02
	Rv1895	zinc-binding alcohol dehydrogenase(Rv1895)	1276.98	1.25	0.49	2.57	1.03E-02	3.36E-02

	Rv2461c	ATP-dependent CLP protease proteolytic subunit 1(clpP1)	3069.19	1.24	0.38	3.30	9.75E-04	5.32E-03
	Rv3470c	acetolactate synthase large subunit(ilvB2)	246.71	1.22	0.37	3.31	9.30E-04	5.09E-03
	Rv1326c	1,4-alpha-glucan branching protein(glgB)	3959.65	1.22	0.31	3.96	7.44E-05	7.07E-04
	Rv2583c	bifunctional (p)ppGpp synthase/hydrolase RelA(relA)	7787.80	1.22	0.24	5.06	4.10E-07	1.16E-05
	Rv2251	flavoprotein(Rv2251)	1275.39	1.21	0.42	2.86	4.28E-03	1.67E-02
	Rv0197	oxidoreductase(Rv0197)	761.52	1.16	0.34	3.39	7.05E-04	4.11E-03
	Rv0091	5'-methylthioadenosine/S-adenosylhomocysteine nucleosidase(mtn)	188.93	1.16	0.35	3.30	9.79E-04	5.32E-03
	Rv1817	flavoprotein(Rv1817)	1601.06	1.14	0.24	4.79	1.66E-06	3.41E-05
	Rv0561c	oxidoreductase(Rv0561c)	569.06	1.14	0.22	5.07	3.99E-07	1.14E-05
	Rv0853c	alpha-keto-acid decarboxylase(pdc)	928.10	1.13	0.31	3.70	2.16E-04	1.61E-03
	Rv0306	oxidoreductase(Rv0306)	220.50	1.13	0.42	2.68	7.47E-03	2.58E-02
	Rv2852c	malate:quinone oxidoreductase(mqo)	3472.51	1.12	0.26	4.29	1.81E-05	2.28E-04
	Rv0224c	methyltransferase(Rv0224c)	385.45	1.12	0.32	3.53	4.23E-04	2.73E-03
	Rv1832	glycine dehydrogenase(gcvB)	26037.31	1.11	0.32	3.53	4.13E-04	2.68E-03
	Rv2458	homocysteine S-methyltransferase MmuM(mmuM)	295.62	1.11	0.28	3.99	6.64E-05	6.38E-04
	Rv0457c	peptidase(Rv0457c)	483.74	1.10	0.37	3.00	2.70E-03	1.17E-02
	Rv2455c	2-oxoglutarate oxidoreductase subunit KorA(Rv2455c)	16091.54	1.10	0.30	3.71	2.04E-04	1.54E-03
	Rv2850c	magnesium chelatase(Rv2850c)	4757.00	1.10	0.27	4.00	6.47E-05	6.28E-04
	Rv3214	phosphoglycerate mutase(gpm2)	773.33	1.09	0.27	4.07	4.70E-05	4.90E-04

	Rv1568	adenosylmethionine--8-amino-7-oxononanoate aminotransferase BioA(bioA)	844.52	1.08	0.34	3.16	1.56E-03	7.63E-03
	Rv2249c	glycerol-3-phosphate dehydrogenase(glpD1)	671.74	1.08	0.34	3.19	1.42E-03	7.14E-03
	Rv3010c	6-phosphofructokinase(pfkA)	1708.08	1.06	0.25	4.26	2.06E-05	2.49E-04
	Rv1381	dihydroorotase(pyrC)	993.21	1.05	0.34	3.08	2.07E-03	9.42E-03
	Rv1723	hydrolase(Rv1723)	396.63	1.05	0.28	3.69	2.22E-04	1.65E-03
	Rv2511	oligoribonuclease(orn)	305.25	1.04	0.36	2.87	4.16E-03	1.64E-02
	Rv0417	thiazole synthase(thiG)	300.16	1.04	0.31	3.39	6.95E-04	4.07E-03
	Rv1599	histidinol dehydrogenase(hisD)	8047.80	1.04	0.32	3.25	1.16E-03	6.09E-03
	Rv2870c	1-deoxy-D-xylulose 5-phosphate reductoisomerase(dxr)	2771.76	1.03	0.28	3.63	2.87E-04	2.01E-03
	Rv0809	phosphoribosylformylglycinamidine cyclo-ligase PurM(purM)	4366.97	1.02	0.28	3.66	2.49E-04	1.82E-03
	Rv0687	NAD-dependent oxidoreductase(Rv0687)	1807.62	1.02	0.36	2.82	4.81E-03	1.84E-02
	Rv1655	acetylornithine aminotransferase(argD)	1443.37	1.02	0.42	2.42	1.54E-02	4.62E-02
**lipid metabolism**	Rv0447c	cyclopropane-fatty-acyl-phospholipid synthase UfaA(ufaA1)	736.72	2.50	0.53	4.67	2.96E-06	5.42E-05
	Rv1180	polyketide beta-ketoacyl synthase(pks3)	1448.71	2.37	0.52	4.56	5.05E-06	8.14E-05
	Rv0564c	glycerol-3-phosphate dehydrogenase(gpdA1)	4665.61	2.32	0.39	6.01	1.81E-09	1.53E-07
	Rv0649	malonyl CoA-acyl carrier protein transacylase(fabD2)	251.84	2.13	0.59	3.64	2.78E-04	1.96E-03
	Rv2382c	polyketide synthetase(mbtC)	1967.88	1.83	0.71	2.57	1.03E-02	3.35E-02
	Rv2383c	phenyloxazoline synthase(mbtB)	5603.01	1.69	0.61	2.76	5.84E-03	2.15E-02
	Rv1715	3-hydroxybutyryl-CoA dehydrogenase FadB(fadB3)	683.78	1.68	0.62	2.71	6.64E-03	2.36E-02

	Rv3515c	acyl-CoA synthetase(fadD19)	1993.63	1.67	0.51	3.27	1.07E-03	5.72E-03
	Rv0752c	acyl-CoA dehydrogenase FadE9(fadE9)	5332.52	1.67	0.52	3.22	1.30E-03	6.65E-03
	Rv0244c	acyl-CoA dehydrogenase FadE5(fadE5)	16767.07	1.59	0.55	2.89	3.81E-03	1.53E-02
	Rv3504	acyl-CoA dehydrogenase FadE26(fadE26)	1276.34	1.58	0.29	5.49	4.03E-08	1.88E-06
	Rv0119	fatty-acid--CoA ligase FadD7(fadD7)	602.40	1.51	0.37	4.09	4.35E-05	4.61E-04
	Rv3546	acetyl-CoA acetyltransferase FadA(fadA5)	1379.48	1.51	0.42	3.62	2.89E-04	2.01E-03
	Rv3505	acyl-CoA dehydrogenase FadE27(fadE27)	967.18	1.50	0.32	4.75	2.02E-06	3.93E-05
	Rv1013	polyketide synthase(pks16)	4140.02	1.48	0.46	3.20	1.36E-03	6.90E-03
	Rv2500c	acyl-CoA dehydrogenase FadE19(fadE19)	2863.09	1.47	0.48	3.09	2.01E-03	9.24E-03
	Rv3573c	acyl-CoA dehydrogenase FadE34(fadE34)	919.68	1.43	0.35	4.07	4.80E-05	4.97E-04
	Rv0975c	acyl-CoA dehydrogenase FadE13(fadE13)	1592.98	1.38	0.51	2.69	7.21E-03	2.51E-02
	Rv3513c	fatty-acid--CoA ligase FadD18(fadD18)	314.67	1.32	0.41	3.25	1.16E-03	6.07E-03
	Rv2484c	diacyglycerol O-acyltransferase(Rv2484c)	6063.06	1.31	0.32	4.13	3.57E-05	3.98E-04
	Rv2384	2,3-dihydroxybenzoate-AMP ligase(mbtA)	348.66	1.29	0.50	2.58	9.93E-03	3.27E-02
	Rv0131c	acyl-CoA dehydrogenase FadE1(fadE1)	559.43	1.29	0.24	5.38	7.31E-08	2.96E-06
	Rv1814	membrane-bound C-5 sterol desaturase(erg3)	659.40	1.27	0.32	3.96	7.45E-05	7.07E-04
	Rv1912c	oxidoreductase FadB(fadB5)	262.09	1.20	0.40	3.03	2.41E-03	1.06E-02
	Rv1467c	acyl-CoA dehydrogenase(fadE15)	1579.01	1.19	0.36	3.32	8.85E-04	4.89E-03
	Rv0271c	acyl-CoA dehydrogenase FadE6(fadE6)	5724.08	1.13	0.20	5.74	9.62E-09	5.75E-07

	Rv1529	fatty-acid--CoA ligase FadD24(fadD24)	957.93	1.10	0.37	2.97	2.99E-03	1.27E-02
	Rv3800c	polyketide synthase(pks13)	95086.18	1.08	0.39	2.78	5.36E-03	2.01E-02
	Rv1181	polyketide beta-ketoacyl synthase(pks4)	6224.01	1.73	0.46	3.73	1.92E-04	1.49E-03
	Rv2613c	AP-4-A phosphorylase(Rv2613c)	692.19	1.03	0.32	3.24	1.22E-03	6.30E-03
**virulence, detoxification and adaptation**	Rv0251c	heat shock protein(hsp)	54556.94	3.72	0.54	6.86	7.00E-12	1.85E-09
	Rv1955	toxin HigB(higB)	2049.08	2.88	0.53	5.45	5.06E-08	2.34E-06
	Rv1956	antitoxin HigA(higA)	1513.52	2.68	0.43	6.24	4.37E-10	5.56E-08
	Rv0563	protease HtpX(htpX)	9180.76	2.67	0.46	5.77	8.01E-09	5.02E-07
	Rv2546	ribonuclease VapC18(vapC18)	151.84	2.51	0.84	2.98	2.87E-03	1.23E-02
	Rv0384c	chaperone protein ClpB(clpB)	38753.61	2.43	0.55	4.45	8.40E-06	1.22E-04
	Rv3181c	antitoxin VapB45(Rv3181c)	167.15	2.26	0.65	3.45	5.65E-04	3.45E-03
	Rv1938	epoxide hydrolase EphB(ephB)	1126.50	2.20	0.65	3.40	6.82E-04	4.00E-03
	Rv3358	toxin RelK(relK)	71.39	2.15	0.56	3.86	1.15E-04	1.00E-03
	Rv0186A	metallothionein(mymT)	11197.76	2.02	0.54	3.75	1.76E-04	1.40E-03
	Rv0549c	ribonuclease VapC3(vapC3)	624.06	1.99	0.35	5.78	7.58E-09	4.85E-07
	Rv0550c	antitoxin VapB3(vapB3)	122.85	1.99	0.51	3.92	8.80E-05	7.99E-04
	Rv0661c	ribonuclease VapC7(vapC7)	41.86	1.95	0.57	3.40	6.72E-04	3.97E-03
	Rv1970	Mce family lipoprotein LprM(lprM)	337.10	1.89	0.36	5.19	2.15E-07	6.88E-06
	Rv3082c	HTH-type transcriptional regulator VirS(virS)	347.94	1.87	0.42	4.48	7.32E-06	1.09E-04

	Rv3357	antitoxin RelJ(relJ)	55.73	1.82	0.52	3.53	4.20E-04	2.71E-03
	Rv1959c	toxin ParE1(parE1)	474.64	1.79	0.43	4.12	3.76E-05	4.09E-04
	Rv3473c	peroxidase BpoA(bpoA)	343.73	1.79	0.51	3.49	4.80E-04	3.01E-03
	Rv2549c	ribonuclease VapC20(vapC20)	254.37	1.76	0.51	3.49	4.80E-04	3.01E-03
	Rv0064A	antitoxin VapB1(vapB1)	202.88	1.72	0.48	3.63	2.88E-04	2.01E-03
	Rv0440	molecular chaperone GroEL(groEL2)	93262.37	1.72	0.43	3.95	7.66E-05	7.24E-04
	Rv2550c	antitoxin VapB20(vapB20)	165.39	1.71	0.53	3.21	1.34E-03	6.83E-03
	Rv3469c	4-hydroxy-2-oxovalerate aldolase MhpE(mhpE)	178.26	1.67	0.45	3.69	2.23E-04	1.65E-03
	Rv2865	antitoxin RelF(relF)	288.85	1.64	0.40	4.07	4.71E-05	4.90E-04
	Rv1960c	antitoxin ParD1(parD1)	222.96	1.61	0.54	2.96	3.10E-03	1.31E-02
	Rv2871	antitoxin VapB43(vapB43)	118.69	1.58	0.58	2.72	6.49E-03	2.33E-02
	Rv1971	Mce family protein Mce3F(mce3F)	294.84	1.55	0.36	4.31	1.65E-05	2.14E-04
	Rv1967	Mce family protein Mce3B(mce3B)	373.63	1.54	0.51	3.02	2.51E-03	1.10E-02
	Rv1991A	antitoxin MazE6(mazE6)	630.86	1.54	0.55	2.81	5.03E-03	1.91E-02
	Rv1991c	mRNA interferase MazF6(mazF6)	816.80	1.52	0.43	3.53	4.09E-04	2.66E-03
	Rv1968	Mce family protein Mce3C(mce3C)	418.98	1.47	0.46	3.20	1.38E-03	6.99E-03
	Rv1966	Mce family protein Mce3A(mce3A)	451.00	1.46	0.58	2.53	1.15E-02	3.65E-02
	Rv3177	peroxidase(Rv3177)	265.94	1.32	0.54	2.45	1.44E-02	4.38E-02
	Rv2374c	heat-inducible transcription repressor HrcA(hrcA)	4894.75	1.31	0.28	4.75	2.01E-06	3.93E-05

	Rv2872	ribonuclease VapC43(vapC43)	250.13	1.27	0.39	3.27	1.06E-03	5.65E-03
	Rv2801c	mRNA interferase MazF9(mazF9)	239.79	1.26	0.31	4.04	5.39E-05	5.40E-04
	Rv1969	Mce family protein Mce3D(mce3D)	264.06	1.25	0.30	4.21	2.54E-05	2.94E-04
	Rv2010	ribonuclease VapC15(vapC15)	2231.85	1.19	0.41	2.93	3.36E-03	1.39E-02
	Rv0582	ribonuclease VapC26(vapC26)	283.35	1.11	0.31	3.65	2.63E-04	1.90E-03
	Rv1962c	ribonuclease VapC35(vapC35)	446.55	1.11	0.32	3.50	4.57E-04	2.90E-03
	Rv1082	mycothiol S-conjugate amidase(mca)	1912.12	1.11	0.24	4.54	5.67E-06	8.89E-05
	Rv0350	chaperone protein DnaK(dnaK)	70186.51	1.08	0.42	2.54	1.10E-02	3.51E-02
	Rv3417c	chaperonin GroEL(groEL1)	23938.68	1.08	0.38	2.85	4.40E-03	1.71E-02
	Rv2063A	mRNA interferase MazF7(mazF7)	216.46	1.06	0.29	3.65	2.64E-04	1.90E-03
	Rv1962A	antitoxin VapB35(vapB35)	196.46	1.01	0.37	2.75	5.95E-03	2.17E-02
**regulatory proteins**	Rv0275c	transcriptional regulator(Rv0275c)	3753.98	2.41	0.42	5.70	1.20E-08	6.88E-07
	Rv1957	SecB-like chaperone(Rv1957)	687.21	2.29	0.48	4.76	1.96E-06	3.88E-05
	Rv1985c	HTH-type transcriptional regulator(Rv1985c)	1388.35	2.29	0.48	4.77	1.89E-06	3.77E-05
	Rv2745c	transcriptional regulator ClgR(clgR)	11382.53	2.09	0.53	3.91	9.15E-05	8.22E-04
	Rv2642	ArsR family transcriptional regulator(Rv2642)	8580.67	2.05	0.70	2.92	3.46E-03	1.42E-02
	Rv3160c	TetR family transcriptional regulator(Rv3160c)	3514.37	1.88	0.65	2.89	3.87E-03	1.55E-02

	Rv0767c	HTH-type transcriptional regulator(Rv0767c)	2851.52	1.82	0.54	3.38	7.37E-04	4.26E-03
	Rv0377	HTH-type transcriptional regulator(Rv0377)	702.71	1.80	0.35	5.21	1.87E-07	6.23E-06
	Rv1267c	transcriptional regulator EmbR(embR)	295.76	1.68	0.41	4.07	4.67E-05	4.89E-04
	Rv0967	copper-sensing transcriptional repressor CsoR(csoR)	13465.76	1.63	0.59	2.74	6.12E-03	2.22E-02
	Rv1994c	HTH-type transcriptional regulator CmtR(cmtR)	2785.94	1.62	0.60	2.68	7.39E-03	2.56E-02
	Rv0324	transcriptional regulator(Rv0324)	495.71	1.60	0.33	4.87	1.12E-06	2.51E-05
	Rv3080c	serine/threonine-protein kinase PknK(pknK)	2318.68	1.59	0.34	4.72	2.39E-06	4.49E-05
	Rv3334	MerR family transcriptional regulator(Rv3334)	910.17	1.59	0.49	3.22	1.26E-03	6.52E-03
	Rv1395	HTH-type transcriptional regulator(Rv1395)	2140.53	1.56	0.56	2.78	5.39E-03	2.02E-02
	Rv1049	transcriptional repressor(Rv1049)	110.17	1.53	0.41	3.75	1.75E-04	1.39E-03
	Rv1460	transcriptional regulator(Rv1460)	5763.11	1.52	0.61	2.49	1.29E-02	4.02E-02
	Rv2324	AsnC family transcriptional regulator(Rv2324)	575.45	1.50	0.35	4.26	2.06E-05	2.49E-04
	Rv3167c	TetR family transcriptional regulator(Rv3167c)	282.22	1.50	0.45	3.35	8.18E-04	4.61E-03
	Rv1963c	transcriptional repressor Mce3R(mce3R)	841.33	1.50	0.48	3.12	1.79E-03	8.50E-03
	Rv0273c	transcriptional regulator(Rv0273c)	513.09	1.49	0.32	4.63	3.71E-06	6.50E-05

	Rv3066	DeoR family transcriptional regulator(Rv3066)	867.88	1.48	0.37	4.02	5.74E-05	5.66E-04
	Rv3124	transcriptional regulator MoaR(moaR1)	355.77	1.45	0.47	3.05	2.29E-03	1.02E-02
	Rv0737	transcriptional regulator(Rv0737)	126.18	1.43	0.38	3.71	2.08E-04	1.56E-03
	Rv2250c	HTH-type transcriptional regulator(Rv2250c)	170.54	1.41	0.40	3.55	3.89E-04	2.57E-03
	Rv1255c	HTH-type transcriptional regulator(Rv1255c)	886.58	1.38	0.27	5.13	2.89E-07	8.96E-06
	Rv0602c	two component DNA binding transcriptional regulator TcrA(tcrA)	94.25	1.38	0.36	3.77	1.60E-04	1.30E-03
	Rv1931c	transcriptional regulator(Rv1931c)	185.11	1.37	0.38	3.65	2.62E-04	1.90E-03
	Rv3862c	transcriptional regulator WhiB6(whiB6)	711.48	1.37	0.46	2.98	2.85E-03	1.22E-02
	Rv0260c	transcriptional regulator(Rv0260c)	133.60	1.34	0.50	2.69	7.10E-03	2.48E-02
	Rv2887	HTH-type transcriptional regulator(Rv2887)	461.90	1.34	0.35	3.85	1.17E-04	1.01E-03
	Rv2711	iron-dependent repressor and activator IdeR(ideR)	9183.81	1.26	0.28	4.41	1.03E-05	1.45E-04
	Rv1266c	serine/threonine-protein kinase PknH(pknH)	2569.54	1.23	0.34	3.58	3.43E-04	2.31E-03
	Rv0880	HTH-type transcriptional regulator(Rv0880)	173.43	1.18	0.38	3.13	1.74E-03	8.32E-03
	Rv0196	HTH-type transcriptional regulator(Rv0196)	89.84	1.15	0.42	2.76	5.75E-03	2.12E-02
	Rv0348	transcriptional regulator(Rv0348)	778.12	1.14	0.32	3.51	4.50E-04	2.87E-03

	Rv2914c	serine/threonine-protein kinase PknI(pknI)	1369.57	1.12	0.31	3.58	3.40E-04	2.31E-03
	Rv2488c	LuxR family transcriptional regulator(Rv2488c)	1020.45	1.07	0.25	4.33	1.52E-05	2.01E-04
	Rv2232	protein tyrosine kinase transcriptional regulator PtkA(ptkA)	551.36	1.07	0.28	3.87	1.10E-04	9.65E-04
	Rv1028c	sensor protein KdpD(kdpD)	1552.24	1.06	0.32	3.37	7.44E-04	4.29E-03
	Rv0744c	transcriptional regulator(Rv0744c)	318.35	1.06	0.41	2.58	9.96E-03	3.27E-02
	Rv0328	transcriptional regulator(Rv0328)	1208.76	1.04	0.36	2.94	3.29E-03	1.37E-02
	Rv3055	TetR family transcriptional regulator(Rv3055)	502.75	1.03	0.31	3.35	8.04E-04	4.56E-03
	Rv0494	HTH-type transcriptional regulator(Rv0494)	382.25	1.01	0.31	3.22	1.29E-03	6.62E-03
**information pathways**	Rv1259	uracil DNA glycosylase(udgB)	548.56	2.40	0.53	4.54	5.53E-06	8.74E-05
	Rv1688	3-methyladenine DNA glycosylase(mpg)	79.78	2.40	0.56	4.28	1.83E-05	2.28E-04
	Rv3202c	ATP-dependent DNA helicase(Rv3202c)	6782.85	1.99	0.50	3.95	7.73E-05	7.28E-04
	Rv3204	DNA-methyltransferase(Rv3204)	638.26	1.98	0.46	4.33	1.49E-05	1.98E-04
	Rv0670	endonuclease IV(end)	4682.21	1.95	0.36	5.41	6.14E-08	2.62E-06
	Rv2555c	alanine--tRNA ligase(alaS)	33011.80	1.79	0.38	4.71	2.52E-06	4.67E-05
	Rv1222	anti-sigma E factor RseA(rseA)	14296.68	1.73	0.46	3.77	1.64E-04	1.32E-03
	Rv2710	RNA polymerase sigma factor SigB(sigB)	59971.69	1.70	0.44	3.83	1.28E-04	1.09E-03
	Rv2554c	Holliday junction resolvase(Rv2554c)	4109.39	1.68	0.47	3.60	3.15E-04	2.16E-03

	Rv3201c	ATP-dependent DNA helicase(Rv3201c)	3384.60	1.67	0.44	3.83	1.31E-04	1.11E-03
	Rv3370c	error-prone DNA polymerase(dnaE2)	5028.32	1.64	0.41	4.01	6.16E-05	6.05E-04
	Rv0233	ribonucleoside-diphosphate reductase subunit beta NrdB(nrdB)	3314.91	1.62	0.48	3.36	7.68E-04	4.40E-03
	Rv0631c	exonuclease V subunit gamma RecC(recC)	679.73	1.62	0.32	5.11	3.19E-07	9.58E-06
	Rv3223c	ECF RNA polymerase sigma factor SigH(sigH)	4478.74	1.58	0.44	3.58	3.41E-04	2.31E-03
	Rv3834c	serine--tRNA ligase(serS)	994.71	1.52	0.59	2.58	9.96E-03	3.27E-02
	Rv2464c	DNA glycosylase(Rv2464c)	2004.52	1.48	0.44	3.34	8.46E-04	4.73E-03
	Rv1221	ECF RNA polymerase sigma factor SigE(sigE)	25455.29	1.48	0.51	2.88	4.00E-03	1.59E-02
	Rv2614c	threonine--tRNA ligase(thrS)	3458.06	1.46	0.38	3.82	1.31E-04	1.11E-03
	Rv3597c	iron-regulated H-NS-like protein(lsr2)	1877.18	1.45	0.49	2.94	3.26E-03	1.36E-02
	Rv2839c	translation initiation factor IF-2(infB)	50714.46	1.37	0.45	3.07	2.12E-03	9.56E-03
	Rv3014c	DNA ligase A(ligA)	1229.30	1.35	0.34	3.97	7.07E-05	6.74E-04
	Rv3062	DNA ligase(ligB)	2938.41	1.32	0.54	2.45	1.43E-02	4.35E-02
	Rv0445c	ECF RNA polymerase sigma factor SigK(sigK)	523.99	1.29	0.23	5.52	3.47E-08	1.66E-06
	Rv2838c	ribosome-binding factor RbfA(rbfA)	7256.76	1.25	0.49	2.56	1.06E-02	3.42E-02
	Rv0629c	exonuclease V subunit alpha RecD(recD)	898.82	1.14	0.31	3.64	2.72E-04	1.94E-03
	Rv0630c	exonuclease V subunit beta RecB(recB)	684.16	1.06	0.28	3.79	1.48E-04	1.21E-03
	Rv3912	anti-sigma-M factor RsmA(Rv3912)	214.11	1.05	0.37	2.82	4.74E-03	1.82E-02
**stable rnas**	tyrT	tRNA-Tyr(tyrT)	9.36	3.38	0.86	3.92	8.68E-05	7.93E-04

	lysT	tRNA-Lys(lysT)	13.56	2.18	0.74	2.95	3.19E-03	1.34E-02
	rnpB	miscRNA(rnpB)	#######	1.88	0.70	2.70	6.88E-03	2.42E-02
	ssr	miscRNA(ssr)	#######	1.69	0.45	3.81	1.40E-04	1.16E-03
	rrl	23S ribosomal RNA(rrl)	#######	1.52	0.49	3.11	1.86E-03	8.78E-03
**PE/PPE**	Rv0754	PE-PGRS family protein PE_PGRS11(PE_PGRS11)	6154.59	2.76	0.52	5.27	1.39E-07	4.93E-06
	Rv0834c	PE-PGRS family protein PE_PGRS14(PE_PGRS14)	21780.90	2.76	0.77	3.60	3.12E-04	2.15E-03
	Rv1808	PPE family protein PPE32(PPE32)	2095.70	2.68	0.56	4.81	1.51E-06	3.15E-05
	Rv1801	PPE family protein PPE29(PPE29)	1613.66	2.51	0.76	3.28	1.03E-03	5.55E-03
	Rv1809	PPE family protein PPE33(PPE33)	2135.91	2.46	0.49	4.98	6.43E-07	1.65E-05
	Rv1802	PPE family protein PPE30(PPE30)	1209.27	2.33	0.71	3.28	1.04E-03	5.55E-03
	Rv1806	PE family protein PE20(PE20)	468.81	2.22	0.67	3.32	9.10E-04	5.00E-03
	Rv0978c	PE-PGRS family protein PE_PGRS17(PE_PGRS17)	799.01	2.22	0.70	3.16	1.55E-03	7.63E-03
	Rv2615c	PE-PGRS family protein PE_PGRS45(PE_PGRS45)	2418.91	1.96	0.65	3.02	2.53E-03	1.10E-02
	Rv0833	PE-PGRS family protein PE_PGRS13(PE_PGRS13)	3136.03	1.60	0.47	3.39	7.09E-04	4.12E-03
	Rv0977	PE-PGRS family protein PE_PGRS16(PE_PGRS16)	1419.52	1.44	0.25	5.64	1.67E-08	9.21E-07
	Rv0278c	PE-PGRS family protein PE_PGRS3(Rv0278c)	1289.71	1.41	0.37	3.82	1.32E-04	1.11E-03
	Rv0256c	PPE family protein PPE2(PPE2)	3649.83	1.30	0.34	3.81	1.37E-04	1.15E-03
	Rv1450c	PE-PGRS family protein PE_PGRS27(PE_PGRS27)	568.56	1.30	0.42	3.09	2.03E-03	9.29E-03
	Rv2634c	PE-PGRS family protein PE_PGRS46(PE_PGRS46)	690.54	1.25	0.30	4.16	3.17E-05	3.58E-04

	Rv1803c	PE-PGRS family protein PE_PGRS32(PE_PGRS32)	901.26	1.20	0.34	3.57	3.60E-04	2.41E-03
	Rv0152c	PE family protein PE2(PE2)	383.16	1.19	0.34	3.49	4.76E-04	2.99E-03
	Rv2487c	PE-PGRS family protein PE_PGRS42(PE_PGRS42)	246.78	1.15	0.42	2.77	5.64E-03	2.09E-02
	Rv0335c	PE family protein PE6(PE6)	334.67	1.14	0.38	3.01	2.65E-03	1.15E-02
	Rv2490c	PE-PGRS family protein PE_PGRS43(PE_PGRS43)	473.13	1.13	0.42	2.73	6.36E-03	2.29E-02
	Rv0279c	PE-PGRS family protein PE_PGRS4(PE_PGRS4)	1709.86	1.09	0.35	3.14	1.71E-03	8.23E-03
	Rv2162c	PE-PGRS family protein PE_PGRS38(PE_PGRS38)	14526.72	1.05	0.32	3.29	1.00E-03	5.43E-03
**conserved hypotheticals**	Rv1685c	hypothetical protein(Rv1685c)	1523.29	4.54	0.56	8.12	4.70E-16	3.11E-13
	Rv1954c	hypothetical protein(Rv1954c)	21268.21	3.28	0.61	5.41	6.26E-08	2.64E-06
	Rv3202a	hypothetical protein(Rv3202a)	626.09	3.02	0.55	5.44	5.25E-08	2.40E-06
	Rv0250c	hypothetical protein(Rv0250c)	6659.42	2.90	0.47	6.20	5.68E-10	6.82E-08
	Rv0449c	hypothetical protein(Rv0449c)	1506.84	2.83	0.61	4.65	3.34E-06	5.99E-05
	Rv1684	hypothetical protein(Rv1684)	234.88	2.63	0.42	6.33	2.53E-10	3.48E-08
	Rv0448c	hypothetical protein(Rv0448c)	360.52	2.54	0.41	6.14	8.39E-10	8.76E-08
	Rv0368c	hypothetical protein(Rv0368c)	209.70	2.53	0.52	4.90	9.39E-07	2.18E-05
	Rv3471c	hypothetical protein(Rv3471c)	64.44	2.41	0.64	3.74	1.87E-04	1.46E-03
	Rv0376c	hypothetical protein(Rv0376c)	571.97	2.36	0.41	5.72	1.04E-08	6.06E-07
	Rv3122	hypothetical protein(Rv3122)	2382.93	2.33	0.73	3.20	1.36E-03	6.89E-03
	Rv3188	hypothetical protein(Rv3188)	373.69	2.31	0.51	4.52	6.23E-06	9.58E-05

	Rv1225c	hypothetical protein(Rv1225c)	11237.53	2.26	0.66	3.40	6.62E-04	3.92E-03
	Rv2689c	hypothetical protein(Rv2689c)	560.10	2.23	0.31	7.20	5.97E-13	1.97E-10
	Rv2744c	hypothetical protein(35kd_ag)	24438.40	2.23	0.50	4.41	1.03E-05	1.45E-04
	Rv2694c	hypothetical protein(Rv2694c)	1762.76	2.22	0.34	6.47	9.75E-11	1.55E-08
	Rv2558	hypothetical protein(Rv2558)	9581.46	2.20	0.44	5.04	4.68E-07	1.30E-05
	Rv3123	hypothetical protein(Rv3123)	1785.99	2.16	0.64	3.35	8.16E-04	4.60E-03
	Rv2616	hypothetical protein(Rv2616)	4085.24	2.15	0.69	3.11	1.85E-03	8.74E-03
	Rv2641	cadmium inducible protein CadI(cadI)	41898.69	2.10	0.76	2.77	5.54E-03	2.07E-02
	Rv0371c	hypothetical protein(Rv0371c)	77.25	2.10	0.59	3.56	3.72E-04	2.48E-03
	Rv0372c	hypothetical protein(Rv0372c)	114.00	2.09	0.60	3.49	4.75E-04	2.99E-03
	Rv2670c	hypothetical protein(Rv2670c)	391.34	2.07	0.48	4.28	1.90E-05	2.34E-04
	Rv3205c	hypothetical protein(Rv3205c)	8498.05	2.05	0.45	4.58	4.58E-06	7.60E-05
	Rv3639c	hypothetical protein(Rv3639c)	86.50	1.93	0.52	3.72	1.96E-04	1.51E-03
	Rv1894c	hypothetical protein(Rv1894c)	5548.95	1.92	0.58	3.33	8.80E-04	4.88E-03
	Rv3079c	hypothetical protein(Rv3079c)	325.00	1.91	0.24	7.99	1.37E-15	7.77E-13
	Rv2491	hypothetical protein(Rv2491)	40.92	1.88	0.61	3.06	2.25E-03	1.00E-02
	Rv0378	hypothetical protein(Rv0378)	145.75	1.87	0.60	3.10	1.96E-03	9.09E-03
	Rv0614	hypothetical protein(Rv0614)	113.78	1.87	0.52	3.62	2.90E-04	2.02E-03
	Rv1670	hypothetical protein(Rv1670)	210.52	1.86	0.64	2.93	3.40E-03	1.41E-02

	Rv2559c	hypothetical protein(Rv2559c)	2284.58	1.84	0.29	6.32	2.54E-10	3.48E-08
	Rv1958c	hypothetical protein(Rv1958c)	751.11	1.84	0.43	4.32	1.55E-05	2.03E-04
	Rv2614A	hypothetical protein(Rv2614A)	467.94	1.83	0.59	3.11	1.89E-03	8.87E-03
	Rv3189	hypothetical protein(Rv3189)	613.41	1.82	0.39	4.70	2.57E-06	4.73E-05
	Rv0311	hypothetical protein(Rv0311)	608.29	1.82	0.57	3.18	1.49E-03	7.43E-03
	Rv2086	hypothetical protein(Rv2086)	266.72	1.79	0.61	2.92	3.51E-03	1.43E-02
	Rv2743c	hypothetical protein(Rv2743c)	8617.87	1.79	0.53	3.34	8.28E-04	4.65E-03
	Rv0826	hypothetical protein(Rv0826)	5981.88	1.79	0.59	3.05	2.29E-03	1.02E-02
	Rv3179	hypothetical protein(Rv3179)	678.63	1.78	0.43	4.11	3.97E-05	4.28E-04
	Rv3180c	ribonuclease VapC45(Rv3180c)	276.44	1.78	0.53	3.35	8.11E-04	4.60E-03
	Rv0968	hypothetical protein(Rv0968)	8093.05	1.77	0.60	2.94	3.24E-03	1.36E-02
	Rv2011c	hypothetical protein(Rv2011c)	1007.92	1.77	0.61	2.91	3.65E-03	1.48E-02
	Rv3218	hypothetical protein(Rv3218)	551.32	1.76	0.41	4.25	2.16E-05	2.57E-04
	Rv1706A	hypothetical protein(Rv1706A)	970.99	1.73	0.62	2.80	5.07E-03	1.92E-02
	Rv1462	hypothetical protein(Rv1462)	15631.14	1.72	0.56	3.06	2.19E-03	9.81E-03
	Rv3222c	hypothetical protein(Rv3222c)	4129.54	1.72	0.46	3.71	2.08E-04	1.57E-03
	Rv0381c	hypothetical protein(Rv0381c)	2823.63	1.71	0.46	3.71	2.04E-04	1.54E-03
	Rv0979c	hypothetical protein(Rv0979c)	27.97	1.71	0.52	3.28	1.02E-03	5.51E-03
	Rv2466c	hypothetical protein(Rv2466c)	6728.83	1.70	0.56	3.02	2.53E-03	1.10E-02

	Rv0976c	hypothetical protein(Rv0976c)	2808.58	1.70	0.58	2.90	3.68E-03	1.49E-02
	Rv2644c	hypothetical protein(Rv2644c)	550.63	1.70	0.54	3.16	1.56E-03	7.63E-03
	Rv0990c	hypothetical protein(Rv0990c)	3098.42	1.69	0.65	2.61	8.97E-03	3.00E-02
	Rv2712c	hypothetical protein(Rv2712c)	3366.04	1.68	0.52	3.26	1.12E-03	5.93E-03
	Rv0063a	hypothetical protein(Rv0063a)	360.07	1.68	0.39	4.36	1.31E-05	1.78E-04
	Rv1228a	hypothetical protein(Rv1228a)	534.64	1.65	0.42	3.93	8.65E-05	7.92E-04
	Rv1571	hypothetical protein(Rv1571)	364.99	1.64	0.52	3.14	1.67E-03	8.09E-03
	Rv3015c	hypothetical protein(Rv3015c)	532.88	1.63	0.36	4.55	5.40E-06	8.56E-05
	Rv3446c	hypothetical protein(Rv3446c)	84.95	1.62	0.61	2.63	8.45E-03	2.85E-02
	Rv2035	hypothetical protein(Rv2035)	1865.74	1.61	0.60	2.69	7.16E-03	2.50E-02
	Rv1805c	hypothetical protein(Rv1805c)	1403.32	1.59	0.45	3.52	4.30E-04	2.76E-03
	Rv1461	hypothetical protein(Rv1461)	28202.79	1.59	0.44	3.61	3.09E-04	2.13E-03
	Rv1062	hypothetical protein(Rv1062)	647.48	1.59	0.39	4.12	3.85E-05	4.17E-04
	Rv0898c	hypothetical protein(Rv0898c)	842.44	1.58	0.52	3.07	2.11E-03	9.54E-03
	Rv1961	hypothetical protein(Rv1961)	372.02	1.58	0.45	3.52	4.24E-04	2.73E-03
	Rv3839	hypothetical protein(Rv3839)	6132.64	1.57	0.60	2.61	9.02E-03	3.01E-02
	Rv0259c	hypothetical protein(Rv0259c)	165.56	1.55	0.43	3.60	3.22E-04	2.20E-03
	Rv2742c	hypothetical protein(Rv2742c)	3504.68	1.54	0.47	3.26	1.11E-03	5.90E-03
	Rv1893	hypothetical protein(Rv1893)	402.30	1.54	0.40	3.87	1.11E-04	9.66E-04

	Rv3627c	hypothetical protein(Rv3627c)	5529.60	1.53	0.23	6.54	6.03E-11	1.04E-08
	Rv0745	hypothetical protein(Rv0745)	57.98	1.52	0.41	3.74	1.80E-04	1.42E-03
	Rv0142	hypothetical protein(Rv0142)	3019.73	1.49	0.61	2.44	1.48E-02	4.48E-02
	Rv3836	hypothetical protein(Rv3836)	2280.28	1.47	0.58	2.56	1.05E-02	3.40E-02
	Rv0325	hypothetical protein(Rv0325)	117.02	1.47	0.41	3.59	3.36E-04	2.28E-03
	Rv2036	hypothetical protein(Rv2036)	2326.88	1.46	0.57	2.55	1.07E-02	3.44E-02
	Rv0991c	hypothetical protein(Rv0991c)	3990.02	1.46	0.60	2.45	1.43E-02	4.35E-02
	Rv1232c	hypothetical protein(Rv1232c)	1671.82	1.46	0.29	5.07	3.97E-07	1.14E-05
	Rv1725c	hypothetical protein(Rv1725c)	381.27	1.45	0.47	3.10	1.96E-03	9.09E-03
	Rv1716	hypothetical protein(Rv1716)	695.96	1.45	0.58	2.48	1.30E-02	4.05E-02
	Rv1051c	hypothetical protein(Rv1051c)	71.52	1.44	0.49	2.91	3.58E-03	1.46E-02
	Rv1075c	hypothetical protein(Rv1075c)	622.77	1.42	0.50	2.84	4.45E-03	1.72E-02
	Rv1466	hypothetical protein(Rv1466)	1862.02	1.41	0.45	3.14	1.71E-03	8.22E-03
	Rv1754c	hypothetical protein(Rv1754c)	648.27	1.41	0.37	3.81	1.38E-04	1.15E-03
	Rv1429	hypothetical protein(Rv1429)	482.68	1.39	0.32	4.40	1.08E-05	1.52E-04
	Rv0358	hypothetical protein(Rv0358)	178.09	1.38	0.38	3.65	2.60E-04	1.88E-03
	Rv2891	hypothetical protein(Rv2891)	91.73	1.37	0.47	2.90	3.74E-03	1.51E-02
	Rv2731	hypothetical protein(Rv2731)	1932.83	1.37	0.32	4.29	1.76E-05	2.24E-04
	Rv0274	hypothetical protein(Rv0274)	892.97	1.37	0.32	4.31	1.66E-05	2.15E-04

	Rv0140	hypothetical protein(Rv0140)	2162.72	1.36	0.51	2.65	8.07E-03	2.75E-02
	Rv2668	hypothetical protein(Rv2668)	258.25	1.36	0.48	2.84	4.53E-03	1.75E-02
	Rv1265	hypothetical protein(Rv1265)	3230.55	1.35	0.49	2.74	6.10E-03	2.22E-02
	Rv0115a	hypothetical protein(Rv0115a)	147.26	1.33	0.51	2.59	9.48E-03	3.14E-02
	Rv2695	hypothetical protein(Rv2695)	531.42	1.31	0.45	2.95	3.20E-03	1.34E-02
	Rv2323c	hypothetical protein(Rv2323c)	603.70	1.31	0.38	3.44	5.82E-04	3.52E-03
	Rv0123	hypothetical protein(Rv0123)	237.23	1.30	0.50	2.60	9.39E-03	3.12E-02
	Rv0141c	hypothetical protein(Rv0141c)	1891.02	1.30	0.49	2.66	7.79E-03	2.68E-02
	Rv0628c	hypothetical protein(Rv0628c)	4679.73	1.29	0.35	3.64	2.74E-04	1.94E-03
	Rv1951c	hypothetical protein(Rv1951c)	106.71	1.27	0.37	3.43	6.01E-04	3.63E-03
	Rv3090	hypothetical protein(Rv3090)	199.54	1.27	0.41	3.11	1.84E-03	8.71E-03
	Rv2717c	hypothetical protein(Rv2717c)	354.63	1.27	0.30	4.25	2.15E-05	2.57E-04
	Rv0810c	hypothetical protein(Rv0810c)	2962.34	1.27	0.43	2.98	2.92E-03	1.25E-02
	Rv3288c	hypothetical protein(usfY)	6340.60	1.27	0.48	2.63	8.43E-03	2.84E-02
	Rv0313	hypothetical protein(Rv0313)	3828.87	1.26	0.41	3.09	2.03E-03	9.29E-03
	Rv3916c	hypothetical protein(Rv3916c)	3453.85	1.24	0.26	4.86	1.18E-06	2.61E-05
	Rv0258c	hypothetical protein(Rv0258c)	282.22	1.24	0.24	5.14	2.69E-07	8.48E-06
	Rv1930c	hypothetical protein(Rv1930c)	53.21	1.22	0.39	3.10	1.96E-03	9.10E-03
	Rv2708c	hypothetical protein(Rv2708c)	588.83	1.22	0.47	2.58	9.74E-03	3.22E-02

	Rv0326	hypothetical protein(Rv0326)	338.91	1.21	0.46	2.63	8.63E-03	2.90E-02
	Rv3472	hypothetical protein(Rv3472)	220.96	1.21	0.39	3.07	2.15E-03	9.64E-03
	Rv2551c	hypothetical protein(Rv2551c)	873.09	1.20	0.40	3.02	2.54E-03	1.11E-02
	Rv1268c	hypothetical protein(Rv1268c)	192.94	1.20	0.29	4.12	3.77E-05	4.09E-04
	Rv2179c	3'-5' exoribonuclease(Rv2179c)	165.83	1.19	0.29	4.13	3.59E-05	3.99E-04
	Rv1063c	NTE family protein(Rv1063c)	1462.63	1.19	0.31	3.81	1.40E-04	1.16E-03
	Rv0329c	hypothetical protein(Rv0329c)	624.59	1.18	0.36	3.32	8.88E-04	4.90E-03
	Rv1831	hypothetical protein(Rv1831)	3924.62	1.17	0.34	3.49	4.92E-04	3.07E-03
	Rv2557	hypothetical protein(Rv2557)	5268.57	1.16	0.42	2.77	5.58E-03	2.08E-02
	Rv0323c	hypothetical protein(Rv0323c)	435.98	1.16	0.39	2.97	2.98E-03	1.26E-02
	Rv1084	hypothetical protein(Rv1084)	3528.51	1.15	0.35	3.28	1.04E-03	5.55E-03
	Rv2714	hypothetical protein(Rv2714)	2123.26	1.14	0.25	4.58	4.64E-06	7.66E-05
	Rv1976c	hypothetical protein(Rv1976c)	674.87	1.12	0.29	3.89	1.01E-04	8.94E-04
	RV1984a	hypothetical protein(RV1984a)	238.58	1.12	0.45	2.48	1.32E-02	4.09E-02
	Rv0383c	hypothetical protein(Rv0383c)	14645.26	1.12	0.44	2.55	1.07E-02	3.44E-02
	Rv1945	hypothetical protein(Rv1945)	1636.71	1.10	0.36	3.08	2.08E-03	9.45E-03
	Rv1073	hypothetical protein(Rv1073)	10195.44	1.10	0.38	2.87	4.05E-03	1.61E-02
	Rv1866	hypothetical protein(Rv1866)	441.07	1.09	0.28	3.88	1.06E-04	9.38E-04
	Rv3835	hypothetical protein(Rv3835)	3108.56	1.09	0.45	2.43	1.50E-02	4.53E-02

	Rv0330c	hypothetical protein(Rv0330c)	470.71	1.08	0.43	2.53	1.13E-02	3.60E-02
	Rv0612	hypothetical protein(Rv0612)	725.01	1.07	0.34	3.18	1.49E-03	7.41E-03
	Rv1382	hypothetical protein(Rv1382)	207.21	1.07	0.40	2.68	7.45E-03	2.58E-02
	Rv1727	hypothetical protein(Rv1727)	135.49	1.06	0.39	2.74	6.21E-03	2.25E-02
	Rv0493c	hypothetical protein(Rv0493c)	405.33	1.06	0.41	2.61	9.06E-03	3.02E-02
	Rv0190	hypothetical protein(Rv0190)	2243.38	1.06	0.40	2.62	8.81E-03	2.95E-02
	Rv0398c	hypothetical protein(Rv0398c)	721.72	1.04	0.34	3.07	2.14E-03	9.64E-03
	Rv1975	hypothetical protein(Rv1975)	363.21	1.03	0.30	3.42	6.38E-04	3.79E-03
	Rv3369	hypothetical protein(Rv3369)	404.54	1.02	0.25	4.10	4.21E-05	4.48E-04
	Rv0360c	hypothetical protein(Rv0360c)	238.31	1.01	0.36	2.81	4.90E-03	1.87E-02
	Rv3626c	hypothetical protein(Rv3626c)	2225.02	1.01	0.28	3.62	2.93E-04	2.03E-03
	Rv2134c	hypothetical protein(Rv2134c)	3532.06	1.00	0.35	2.87	4.12E-03	1.63E-02
**insertion seqs and phages**	Rv1574	phage protein(Rv1574)	170.78	2.40	0.85	2.82	4.87E-03	1.86E-02
	Rv1575	phage protein(Rv1575)	307.17	2.27	0.90	2.52	1.16E-02	3.67E-02
	Rv1578c	phage protein(Rv1578c)	690.14	2.14	0.79	2.70	6.85E-03	2.42E-02
	Rv3844	transposase(Rv3844)	235.55	1.09	0.26	4.10	4.11E-05	4.42E-04

(II) Significantly downregulated genes (L2Fc <-1, adj. p-value < 0.05)

**Functional Category**	**Locus_tags**	**Genes**	**baseMean**	**L2Fc**	**lfcSE**	**stat**	**pvalue**	**padj**
	Rv0431	tuberculin-like peptide(Rv0431)	473.04	-1.01	0.40	-2.51	1.21E-02	3.80E-02

**cell wall and cell processes**	Rv2543	lipoprotein LppA(lppA)	2334.90	-1.01	0.38	-2.66	7.84E-03	2.69E-02
	Rv2721c	hypothetical protein(Rv2721c)	4893.40	-1.02	0.33	-3.12	1.82E-03	8.61E-03
	Rv0227c	membrane protein(Rv0227c)	989.21	-1.02	0.38	-2.69	7.11E-03	2.48E-02
	Rv2127	L-asparagine permease(ansP1)	2403.84	-1.03	0.41	-2.54	1.12E-02	3.56E-02
	Rv1272c	drug ABC transporter ATP-binding protein(Rv1272c)	1354.15	-1.04	0.24	-4.26	2.04E-05	2.48E-04
	Rv2790c	lipid-transfer protein(ltp1)	3328.05	-1.05	0.38	-2.73	6.36E-03	2.29E-02
	Rv2144c	transmembrane protein(Rv2144c)	1786.94	-1.05	0.26	-4.06	4.94E-05	5.08E-04
	Rv0403c	membrane protein MmpS1(mmpS1)	89.44	-1.05	0.33	-3.21	1.35E-03	6.86E-03
	Rv2937	daunorubicin ABC transporter permease DrrB(drrB)	1295.58	-1.08	0.34	-3.17	1.50E-03	7.43E-03
	Rv3593	lipoprotein LpqF(lpqF)	2477.49	-1.09	0.22	-4.93	8.09E-07	1.98E-05
	Rv1508c	membrane protein(Rv1508c)	1330.04	-1.09	0.43	-2.55	1.07E-02	3.44E-02
	Rv0713	transmembrane protein(Rv0713)	727.62	-1.10	0.33	-3.33	8.55E-04	4.78E-03
	Rv2093c	Sec-independent protein translocase transmembrane protein TatC(tatC)	2426.97	-1.10	0.31	-3.60	3.20E-04	2.19E-03
	Rv0935	phosphate ABC transporter permease PstC(pstC1)	1949.99	-1.11	0.34	-3.23	1.22E-03	6.32E-03
	Rv3584	lipoprotein LpqE(lpqE)	1423.10	-1.11	0.34	-3.22	1.30E-03	6.65E-03
	Rv1458c	antibiotic ABC transporter ATP-binding protein(Rv1458c)	788.34	-1.12	0.34	-3.27	1.09E-03	5.81E-03
	Rv3277	transmembrane protein(Rv3277)	759.08	-1.12	0.41	-2.72	6.53E-03	2.33E-02
	Rv3682	bifunctional penicillin-insensitive transglycosylase/penicillin-sensitive transpeptidase(ponA2)	7436.66	-1.12	0.32	-3.51	4.48E-04	2.86E-03
	Rv2446c	integral membrane protein(Rv2446c)	284.39	-1.13	0.35	-3.22	1.29E-03	6.64E-03
	Rv3806c	decaprenyl-phosphate phosphoribosyltransferase(ubiA)	1543.21	-1.14	0.45	-2.54	1.12E-02	3.55E-02
	Rv0346c	L-asparagine permease(ansP2)	2090.73	-1.14	0.40	-2.87	4.13E-03	1.63E-02
	Rv3006	lipoprotein LppZ(lppZ)	2172.94	-1.14	0.30	-3.87	1.10E-04	9.65E-04
	Rv0206c	transmembrane transport protein MmpL3(mmpL3)	13379.86	-1.15	0.32	-3.59	3.30E-04	2.25E-03
	Rv0655	ABC transporter ATP-binding protein(mkl)	13349.15	-1.15	0.48	-2.40	1.65E-02	4.91E-02
	Rv2905	lipoprotein LppW(lppW)	1749.34	-1.15	0.34	-3.38	7.26E-04	4.21E-03
	Rv0870c	integral membrane protein(Rv0870c)	1262.89	-1.15	0.34	-3.37	7.63E-04	4.37E-03
	Rv0912	transmembrane protein(Rv0912)	232.15	-1.17	0.26	-4.57	4.91E-06	8.02E-05

Rv0051	transmembrane protein(Rv0051)	1036.78	-1.18	0.28	-4.14	3.44E-05	3.84E-04
Rv0961	integral membrane protein(Rv0961)	437.14	-1.18	0.40	-2.92	3.49E-03	1.43E-02
Rv2473	hypothetical protein(Rv2473)	464.44	-1.20	0.23	-5.21	1.90E-07	6.27E-06
Rv0732	preprotein translocase SecY(secY)	1311.03	-1.20	0.39	-3.10	1.91E-03	8.93E-03
Rv2942	transmembrane transport protein MmpL7(mmpL7)	16263.41	-1.21	0.44	-2.74	6.19E-03	2.24E-02
Rv3882c	ESX-1 secretion system protein EccE1(eccE1)	2650.19	-1.21	0.27	-4.49	7.07E-06	1.06E-04
Rv1174c	low molecular weight T-cell antigen(TB8.4)	6614.36	-1.21	0.32	-3.75	1.73E-04	1.38E-03
Rv0820	phosphate ABC transporter ATP-binding protein PhoT(phoT)	1332.51	-1.22	0.34	-3.60	3.19E-04	2.19E-03
Rv1980c	immunogenic protein Mpt64(mpt64)	2086.64	-1.22	0.39	-3.16	1.59E-03	7.75E-03
Rv3587c	membrane protein(Rv3587c)	1901.22	-1.23	0.37	-3.36	7.72E-04	4.41E-03
Rv1491c	TVP38/TMEM64 family membrane protein(Rv1491c)	1767.72	-1.23	0.42	-2.93	3.42E-03	1.41E-02
Rv2588c	membrane protein secretion factor YajC(yajC)	1067.64	-1.23	0.46	-2.69	7.10E-03	2.48E-02
Rv1797	ESX-5 type VII secretion system protein EccE(eccE5)	5675.19	-1.24	0.43	-2.89	3.81E-03	1.53E-02
Rv0175	Mce associated membrane protein(Rv0175)	4629.26	-1.25	0.31	-3.98	7.01E-05	6.70E-04
Rv1487	hypothetical protein(Rv1487)	584.52	-1.25	0.48	-2.61	9.02E-03	3.01E-02
Rv0506	membrane protein MmpS2(mmpS2)	305.57	-1.25	0.37	-3.43	6.07E-04	3.65E-03
Rv2136c	undecaprenyl-diphosphatase(Rv2136c)	1268.53	-1.26	0.36	-3.54	4.03E-04	2.65E-03
Rv1481	membrane protein(Rv1481)	3099.98	-1.29	0.28	-4.61	4.00E-06	6.82E-05
Rv0936	phosphate ABC transporter permease PstA(pstA2)	861.69	-1.29	0.40	-3.20	1.39E-03	7.01E-03
Rv1038c	ESAT-6 like protein EsxJ(esxJ)	409.55	-1.29	0.43	-2.99	2.81E-03	1.21E-02
Rv0545c	low-affinity inorganic phosphate transporter(pitA)	6697.49	-1.30	0.30	-4.26	2.02E-05	2.46E-04
Rv0519c	membrane protein(Rv0519c)	496.89	-1.31	0.46	-2.87	4.10E-03	1.63E-02
Rv2846c	MFS-type transporter EfpA(efpA)	17853.51	-1.33	0.50	-2.65	8.13E-03	2.77E-02
Rv1739c	sulfate ABC transporter permease(Rv1739c)	4563.06	-1.35	0.51	-2.66	7.83E-03	2.69E-02
Rv2081c	transmembrane protein(Rv2081c)	1143.86	-1.35	0.34	-3.94	8.27E-05	7.68E-04
Rv1565c	acyltransferase(Rv1565c)	2454.60	-1.35	0.33	-4.12	3.76E-05	4.09E-04
Rv3805c	terminal beta-(1->2)-arabinofuranosyltransferase(aftB)	2738.43	-1.37	0.39	-3.53	4.15E-04	2.69E-03
Rv0011c	cell division protein CrgA(Rv0011c)	787.58	-1.37	0.37	-3.71	2.04E-04	1.54E-03

Rv2903c	signal peptidase(lepB)	7601.20	-1.38	0.28	-4.89	1.01E-06	2.30E-05
Rv3884c	ESX-2 secretion system protein EccA(eccA2)	1168.04	-1.38	0.35	-3.92	8.77E-05	7.98E-04
Rv2936	daunorubicin ABC transporter ATP-binding protein DrrA(drrA)	3471.74	-1.38	0.33	-4.25	2.15E-05	2.57E-04
Rv2128	transmembrane protein(Rv2128)	314.68	-1.39	0.39	-3.58	3.45E-04	2.33E-03
Rv3918c	chromosome partitioning protein ParA(parA)	1986.14	-1.44	0.36	-4.05	5.03E-05	5.13E-04
Rv2150c	cell division protein FtsZ(ftsZ)	5788.23	-1.44	0.35	-4.14	3.40E-05	3.81E-04
Rv3236c	integral membrane transport protein(Rv3236c)	890.66	-1.44	0.41	-3.54	4.07E-04	2.66E-03
Rv1418	lipoprotein LprH(lprH)	750.01	-1.46	0.26	-5.56	2.74E-08	1.39E-06
Rv0178	Mce associated membrane protein(Rv0178)	1714.54	-1.46	0.28	-5.18	2.23E-07	7.08E-06
Rv3781	O-antigen/lipopolysaccharide ABC transporter ATP-binding protein RfbE(rfbE)	594.62	-1.47	0.35	-4.19	2.75E-05	3.16E-04
Rv2154c	lipid II flippase FtsW(ftsW)	3185.86	-1.47	0.51	-2.87	4.06E-03	1.61E-02
Rv0205	transmembrane protein(Rv0205)	796.94	-1.48	0.40	-3.71	2.09E-04	1.57E-03
Rv1798	ESX-5 type VII secretion system protein EccA(eccA5)	3945.51	-1.48	0.37	-4.03	5.49E-05	5.45E-04
Rv1863c	integral membrane protein(Rv1863c)	2269.57	-1.49	0.30	-4.92	8.71E-07	2.09E-05
Rv2938	daunorubicin ABC transporter permease DrrC(drrC)	1179.29	-1.49	0.32	-4.65	3.36E-06	6.01E-05
Rv2376c	low molecular weight antigen MTB12(cfp2)	1069.08	-1.53	0.38	-4.06	4.99E-05	5.11E-04
Rv3782	galactofuranosyl transferase GlfT(glfT1)	1709.69	-1.54	0.34	-4.48	7.55E-06	1.12E-04
Rv3000	transmembrane protein(Rv3000)	287.32	-1.54	0.57	-2.70	6.85E-03	2.42E-02
Rv0908	metal cation transporter ATPase E(ctpE)	1133.04	-1.57	0.44	-3.55	3.79E-04	2.52E-03
Rv1677	lipoprotein DsbF(dsbF)	1076.87	-1.60	0.42	-3.80	1.42E-04	1.18E-03
Rv2945c	lipoprotein LppX(lppX)	425.25	-1.66	0.35	-4.73	2.19E-06	4.18E-05
Rv1368	lipoprotein LprF(lprF)	1381.76	-1.67	0.43	-3.83	1.26E-04	1.07E-03
Rv1678	integral membrane protein(Rv1678)	1335.16	-1.70	0.36	-4.79	1.65E-06	3.41E-05
Rv0176	Mce associated transmembrane protein(Rv0176)	2456.81	-1.70	0.34	-4.98	6.36E-07	1.65E-05
Rv1522c	transmembrane transport protein MmpL12(mmpL12)	2375.86	-1.74	0.50	-3.51	4.55E-04	2.89E-03
Rv2346c	ESAT-6 like protein EsxO(esxO)	3027.80	-1.74	0.61	-2.86	4.27E-03	1.67E-02
Rv0229c	hypothetical protein(Rv0229c)	1803.59	-1.76	0.47	-3.73	1.93E-04	1.49E-03

	Rv0821c	phosphate-transport system transcriptional regulator PhoY2(phoY2)	1589.88	-1.81	0.31	-5.79	7.04E-09	4.58E-07
	Rv0934	phosphate ABC transporter substrate-binding lipoprotein PstS(pstS1)	1721.92	-1.81	0.45	-3.99	6.48E-05	6.28E-04
	Rv1417	membrane protein(Rv1417)	616.30	-1.83	0.35	-5.27	1.36E-07	4.85E-06
	Rv1435c	hypothetical protein(Rv1435c)	3622.60	-1.85	0.46	-4.04	5.28E-05	5.33E-04
	Rv3783	O-antigen/lipopolysaccharide ABC transporter permease RfbD(rfbD)	942.14	-1.85	0.41	-4.51	6.47E-06	9.87E-05
	Rv3615c	ESX-1 secretion-associated protein EspC(espC)	2247.26	-1.87	0.75	-2.48	1.32E-02	4.09E-02
	Rv3004	low molecular weight protein antigen 6(cfp6)	511.14	-1.93	0.37	-5.21	1.85E-07	6.21E-06
	Rv3616c	ESX-1 secretion-associated protein EspA(espA)	7093.39	-1.98	0.78	-2.54	1.11E-02	3.53E-02
	Rv3919c	16S rRNA (guanine(527)-N(7))-methyltransferase RsmG(gid)	1576.53	-1.99	0.37	-5.38	7.28E-08	2.96E-06
	Rv1793	ESAT-6 like protein EsxN(esxN)	1969.19	-2.00	0.62	-3.22	1.28E-03	6.58E-03
	Rv0173	Mce family lipoprotein LprK(lprK)	2525.29	-2.15	0.56	-3.84	1.24E-04	1.06E-03
	Rv0933	phosphate ABC transporter ATP-binding protein PstB(pstB)	789.33	-2.24	0.51	-4.38	1.16E-05	1.61E-04
	Rv1614	prolipoprotein diacylglyceryl transferase(lgt)	3121.37	-2.27	0.43	-5.22	1.82E-07	6.21E-06
	Rv2347c	ESAT-6 like protein EsxP(esxP)	2803.57	-2.39	0.54	-4.45	8.70E-06	1.26E-04
	Rv0073	glutamine ABC transporter ATP-binding protein(Rv0073)	804.74	-2.45	0.44	-5.58	2.45E-08	1.26E-06
	Rv1198	ESAT-6 like protein EsxL(esxL)	4428.12	-2.56	0.52	-4.90	9.40E-07	2.18E-05
	Rv3921c	membrane protein insertase YidC(Rv3921c)	3719.24	-2.84	0.49	-5.81	6.40E-09	4.29E-07
	Rv1197	ESAT-6 like protein EsxK(esxK)	3976.73	-3.44	0.54	-6.40	1.57E-10	2.31E-08
	Rv1884c	resuscitation-promoting factor RpfC(rpfC)	2845.12	-4.16	0.67	-6.24	4.48E-10	5.56E-08
	Rv3614c	ESX-1 secretion-associated protein EspD(espD)	4101.84	-1.81	0.69	-2.64	8.34E-03	2.82E-02
	Rv0010c	membrane protein(Rv0010c)	1236.28	-1.84	0.37	-5.03	4.84E-07	1.32E-05
	Rv1440	protein-export membrane protein SecG(secG)	317.56	-2.29	0.38	-6.05	1.43E-09	1.35E-07
	Rv1698	copper transporter MctB(mctB)	917.39	-2.31	0.48	-4.81	1.49E-06	3.13E-05
	Rv1857	molybdate ABC transporter substrate-binding lipoprotein ModA(modA)	815.83	-2.41	0.60	-3.99	6.58E-05	6.37E-04
	Rv0072	glutamine ABC transporter permease(Rv0072)	815.70	-2.42	0.39	-6.18	6.62E-10	7.29E-08
	Rv2773c	4-hydroxy-tetrahydrodipicolinate reductase(dapB)	751.88	-1.00	0.31	-3.26	1.13E-03	5.97E-03

**intermediary metabolism and respiration**	Rv0919	GCN5-like N-acetyltransferase(Rv0919)	586.86	-1.01	0.35	-2.85	4.33E-03	1.69E-02
	Rv2981c	D-alanine--D-alanine ligase(ddlA)	1878.64	-1.01	0.37	-2.72	6.53E-03	2.33E-02
	Rv3592	heme-degrading monooxygenase(Rv3592)	771.07	-1.02	0.42	-2.44	1.48E-02	4.49E-02
	Rv0363c	fructose-bisphosphate aldolase(fba)	2042.03	-1.03	0.32	-3.22	1.27E-03	6.57E-03
	Rv0501	UDP-glucose 4-epimerase GalE(galE2)	2852.72	-1.03	0.27	-3.74	1.82E-04	1.43E-03
	Rv1511	GDP-D-mannose dehydratase GmdA(gmdA)	916.23	-1.04	0.33	-3.18	1.50E-03	7.43E-03
	Rv2222c	glutamine synthetase(glnA2)	4232.74	-1.04	0.33	-3.13	1.73E-03	8.32E-03
	Rv3224	iron-regulated short-chain dehydrogenase/reductase(Rv3224)	1663.35	-1.04	0.39	-2.68	7.41E-03	2.57E-02
	Rv0730	GCN5-like N-acetyltransferase(Rv0730)	1175.24	-1.06	0.24	-4.37	1.27E-05	1.73E-04
	Rv1155	pyridoxine/pyridoxamine 5'-phosphate oxidase(Rv1155)	766.98	-1.07	0.39	-2.76	5.82E-03	2.14E-02
	Rv1559	threonine dehydratase IlvA(ilvA)	2510.64	-1.08	0.21	-5.11	3.30E-07	9.78E-06
	Rv0139	oxidoreductase(Rv0139)	790.24	-1.08	0.29	-3.71	2.05E-04	1.55E-03
	Rv1416	6,7-dimethyl-8-ribityllumazine synthase(ribH)	1359.10	-1.10	0.34	-3.20	1.38E-03	6.98E-03
	Rv2899c	formate dehydrogenase accessory protein FdhD(fdhD)	505.36	-1.10	0.37	-2.96	3.11E-03	1.31E-02
	Rv2112c	pup deamidase/depupylase(dop)	2372.50	-1.11	0.36	-3.11	1.86E-03	8.78E-03
	Rv1848	urease subunit gamma(ureA)	197.29	-1.12	0.44	-2.56	1.05E-02	3.40E-02
	Rv3247c	thymidylate kinase(tmk)	1273.05	-1.12	0.46	-2.44	1.48E-02	4.47E-02
	Rv0734	methionine aminopeptidase(mapA)	1268.19	-1.14	0.34	-3.36	7.78E-04	4.43E-03
	Rv1524	glycosyltransferase(Rv1524)	360.98	-1.15	0.39	-2.92	3.48E-03	1.43E-02
	Rv2509	short-chain type dehydrogenase/reductase(Rv2509)	995.70	-1.17	0.32	-3.67	2.38E-04	1.75E-03
	Rv1699	CTP synthase(pyrG)	3821.99	-1.18	0.33	-3.62	2.95E-04	2.04E-03
	Rv0228	acyltransferase(Rv0228)	1357.46	-1.18	0.24	-4.90	9.77E-07	2.23E-05
	Rv1017c	ribose-phosphate pyrophosphokinase(prsA)	1359.81	-1.19	0.35	-3.44	5.76E-04	3.50E-03
	Rv3117	thiosulfate sulfurtransferase(cysA3)	1855.78	-1.19	0.43	-2.75	5.99E-03	2.19E-02
	Rv1099c	fructose 1,6-bisphosphatase(glpX)	920.80	-1.19	0.35	-3.40	6.73E-04	3.97E-03
	Rv1324	thioredoxin(Rv1324)	5799.32	-1.20	0.35	-3.40	6.77E-04	3.99E-03
	Rv3248c	adenosylhomocysteinase(sahH)	12415.52	-1.21	0.30	-4.08	4.57E-05	4.82E-04
	Rv0868c	cyclic pyranopterin monophosphate synthase(moaD2)	386.07	-1.22	0.35	-3.54	4.01E-04	2.64E-03

Rv1173	FO synthase(fbiC)	10090.25	-1.23	0.27	-4.56	5.03E-06	8.14E-05
Rv0948c	chorismate mutase(Rv0948c)	479.90	-1.23	0.41	-3.01	2.61E-03	1.13E-02
Rv1856c	oxidoreductase(Rv1856c)	1895.84	-1.24	0.39	-3.16	1.59E-03	7.75E-03
Rv1751	oxidoreductase(Rv1751)	1942.15	-1.24	0.34	-3.63	2.89E-04	2.01E-03
Rv1523	methyltransferase(Rv1523)	365.81	-1.25	0.37	-3.36	7.85E-04	4.46E-03
Rv0733	adenylate kinase(adk)	759.46	-1.25	0.43	-2.89	3.80E-03	1.53E-02
Rv1941	short-chain type dehydrogenase/reductase(Rv1941)	1993.56	-1.26	0.28	-4.50	6.77E-06	1.03E-04
Rv1320c	adenylate cyclase(Rv1320c)	1594.03	-1.26	0.27	-4.65	3.31E-06	5.96E-05
Rv0805	3',5'-cyclic adenosine monophosphate phosphodiesterase CpdA(Rv0805)	3566.66	-1.27	0.35	-3.66	2.54E-04	1.85E-03
Rv3003c	acetolactate synthase large subunit IlvB(ilvB1)	5204.46	-1.27	0.50	-2.55	1.09E-02	3.48E-02
Rv2178c	phospho-2-dehydro-3-deoxyheptonate aldolase AroG(aroG)	2696.20	-1.27	0.33	-3.87	1.07E-04	9.40E-04
Rv1220c	methyltransferase(Rv1220c)	786.96	-1.29	0.47	-2.77	5.57E-03	2.07E-02
Rv1096	glycosyl hydrolase(Rv1096)	959.41	-1.32	0.26	-5.04	4.61E-07	1.29E-05
Rv3119	molybdopterin synthase catalytic subunit 1(moaE1)	366.09	-1.32	0.28	-4.67	3.08E-06	5.61E-05
Rv1475c	iron-regulated aconitate hydratase(acn)	9703.53	-1.32	0.29	-4.52	6.30E-06	9.65E-05
Rv2970c	lipase/esterase LipN(lipN)	1820.45	-1.33	0.20	-6.63	3.41E-11	6.44E-09
Rv2029c	6-phosphofructokinase PfkB(pfkB)	5391.23	-1.34	0.52	-2.58	9.75E-03	3.22E-02
Rv2200c	cytochrome C oxidase subunit II(ctaC)	3547.23	-1.34	0.44	-3.03	2.45E-03	1.08E-02
Rv2860c	glutamine synthetase(glnA4)	1082.99	-1.35	0.32	-4.21	2.53E-05	2.94E-04
Rv2074	pyridoxamine 5'-phosphate oxidase(Rv2074)	1164.91	-1.35	0.41	-3.28	1.03E-03	5.53E-03
Rv2988c	3-isopropylmalate dehydratase large subunit(leuC)	5845.89	-1.36	0.48	-2.85	4.43E-03	1.72E-02
Rv3001c	ketol-acid reductoisomerase(ilvC)	3099.48	-1.36	0.48	-2.83	4.65E-03	1.79E-02
Rv2445c	nucleoside diphosphate kinase(ndkA)	456.01	-1.36	0.41	-3.29	9.86E-04	5.36E-03
Rv2427c	gamma-glutamyl phosphate reductase(proA)	1816.09	-1.37	0.35	-3.96	7.50E-05	7.10E-04
Rv2334	O-acetylserine sulfhydrylase(cysK1)	2620.76	-1.37	0.44	-3.10	1.96E-03	9.10E-03
Rv2971	oxidoreductase(Rv2971)	1274.98	-1.39	0.37	-3.72	2.00E-04	1.53E-03
Rv0896	citrate synthase 1(gltA2)	3652.49	-1.40	0.43	-3.25	1.15E-03	6.03E-03

Rv0646c	lipase/esterase LipG(lipG)	1019.48	-1.41	0.40	-3.57	3.57E-04	2.40E-03
Rv0635	(3R)-hydroxyacyl-ACP dehydratase subunit HadA(hadA)	597.49	-1.41	0.40	-3.54	4.02E-04	2.64E-03
Rv3151	NADH-quinone oxidoreductase subunit G(nuoG)	9822.91	-1.42	0.54	-2.65	7.94E-03	2.71E-02
Rv2857c	3-oxoacyl-ACP reductase(Rv2857c)	511.66	-1.43	0.24	-5.90	3.67E-09	2.64E-07
Rv3149	NADH-quinone oxidoreductase subunit E(nuoE)	2274.93	-1.43	0.50	-2.88	4.02E-03	1.60E-02
Rv3232c	polyphosphate kinase(ppk2)	573.06	-1.44	0.32	-4.48	7.30E-06	1.09E-04
Rv1436	glyceraldehyde 3-phosphate dehydrogenase(gap)	2464.81	-1.44	0.38	-3.81	1.42E-04	1.17E-03
Rv1500	glycosyltransferase(Rv1500)	398.15	-1.44	0.54	-2.68	7.33E-03	2.55E-02
Rv1095	phosphate starvation-inducible protein PsiH(phoH2)	15520.16	-1.47	0.39	-3.75	1.80E-04	1.42E-03
Rv2682c	1-deoxy-D-xylulose 5-phosphate synthase(dxs1)	6791.66	-1.48	0.40	-3.73	1.93E-04	1.49E-03
Rv1106c	3 beta-hydroxysteroid dehydrogenase/delta 5-->4-isomerase(Rv1106c)	1231.60	-1.55	0.33	-4.74	2.11E-06	4.06E-05
Rv0637	(3R)-hydroxyacyl-ACP dehydratase subunit HadC(hadC)	1182.31	-1.56	0.39	-4.04	5.45E-05	5.43E-04
Rv1098c	fumarate hydratase(fum)	1633.05	-1.58	0.38	-4.21	2.53E-05	2.94E-04
Rv2993c	2-hydroxyhepta-2,4-diene-1,7-dioate isomerase(Rv2993c)	719.37	-1.59	0.37	-4.34	1.43E-05	1.92E-04
Rv1512	nucleotide-sugar epimerase EpiA(epiA)	760.17	-1.60	0.38	-4.24	2.27E-05	2.67E-04
Rv3741c	oxidoreductase(Rv3741c)	303.18	-1.61	0.48	-3.34	8.45E-04	4.73E-03
Rv0500	pyrroline-5-carboxylate reductase(proC)	456.46	-1.62	0.35	-4.60	4.30E-06	7.20E-05
Rv2350c	membrane-associated phospholipase B(plcB)	798.47	-1.63	0.57	-2.86	4.22E-03	1.66E-02
Rv0220	esterase LipC(lipC)	4152.66	-1.65	0.31	-5.37	7.91E-08	3.15E-06
Rv0696	mycofactocin biosynthesis glycosyltransferase MftF(Rv0696)	2067.69	-1.71	0.47	-3.64	2.69E-04	1.92E-03
Rv1437	phosphoglycerate kinase(pgk)	1992.07	-1.75	0.38	-4.62	3.77E-06	6.59E-05
Rv3157	NADH-quinone oxidoreductase subunit M(nuoM)	4088.95	-1.79	0.49	-3.65	2.66E-04	1.90E-03
Rv1631	dephospho-CoA kinase CoaE(coaE)	4168.78	-1.85	0.30	-6.15	7.85E-10	8.41E-08
Rv3002c	acetolactate synthase small subunit(ilvN)	1099.84	-1.86	0.53	-3.54	4.07E-04	2.66E-03
Rv3147	NADH-quinone oxidoreductase subunit C(nuoC)	1239.36	-1.88	0.46	-4.08	4.56E-05	4.82E-04
Rv1869c	reductase(Rv1869c)	3402.61	-1.90	0.44	-4.30	1.69E-05	2.17E-04
Rv2861c	methionine aminopeptidase(mapB)	1410.78	-1.91	0.36	-5.24	1.63E-07	5.62E-06

Rv3150	NADH-quinone oxidoreductase subunit F(nuoF)	2511.01	-1.92	0.56	-3.43	6.14E-04	3.68E-03
Rv0711	arylsulfatase AtsA(atsA)	3026.86	-1.96	0.43	-4.60	4.26E-06	7.16E-05
Rv1438	triosephosphate isomerase(tpi)	1236.15	-1.99	0.32	-6.18	6.27E-10	7.16E-08
Rv2987c	3-isopropylmalate dehydratase small subunit(leuD)	1548.91	-1.99	0.53	-3.80	1.46E-04	1.19E-03
Rv2952	phthiotriol/phenolphthiotriol dimycocerosates methyltransferase(Rv2952)	646.49	-2.02	0.45	-4.46	8.26E-06	1.20E-04
Rv3742c	oxidoreductase(Rv3742c)	435.00	-2.02	0.58	-3.47	5.15E-04	3.19E-03
Rv2951c	phthiodiolone/phenolphthiodiolone dimycocerosates ketoreductase(Rv2951c)	1218.82	-2.05	0.46	-4.48	7.62E-06	1.13E-04
Rv3154	NADH-quinone oxidoreductase subunit J(nuoJ)	1724.21	-2.05	0.53	-3.89	9.99E-05	8.88E-04
Rv2276	cytochrome P450 Cyp121(cyp121)	394.69	-2.09	0.57	-3.65	2.63E-04	1.90E-03
Rv2007c	ferredoxin(fdxA)	6550.99	-2.12	0.72	-2.94	3.26E-03	1.36E-02
Rv3148	NADH-quinone oxidoreductase subunit D(nuoD)	2822.74	-2.13	0.55	-3.90	9.71E-05	8.66E-04
Rv3377c	type B diterpene cyclase(Rv3377c)	1034.07	-2.19	0.47	-4.62	3.92E-06	6.73E-05
Rv3155	NADH-quinone oxidoreductase subunit K(nuoK)	255.13	-2.25	0.64	-3.53	4.19E-04	2.71E-03
Rv3156	NADH-quinone oxidoreductase subunit L(nuoL)	5156.83	-2.25	0.56	-4.03	5.49E-05	5.45E-04
Rv1611	indole-3-glycerol phosphate synthase(trpC)	2686.76	-2.28	0.43	-5.33	9.72E-08	3.71E-06
Rv1872c	L-lactate dehydrogenase(lldD2)	27812.08	-2.33	0.34	-6.77	1.31E-11	2.88E-09
Rv3152	NADH-quinone oxidoreductase subunit H(nuoH)	3130.18	-2.38	0.64	-3.71	2.04E-04	1.54E-03
Rv3146	NADH-quinone oxidoreductase subunit B(nuoB)	896.57	-2.42	0.47	-5.11	3.21E-07	9.58E-06
Rv3145	NADH-quinone oxidoreductase subunit A(nuoA)	1197.04	-2.43	0.48	-5.06	4.26E-07	1.20E-05
Rv0693	mycofactocin radical SAM maturase MftC(Rv0693)	5357.07	-2.59	0.54	-4.79	1.69E-06	3.43E-05
Rv3153	NADH-quinone oxidoreductase subunit I(nuoI)	1481.24	-2.61	0.58	-4.53	5.79E-06	8.98E-05
Rv3727	oxidoreductase(Rv3727)	1112.82	-2.77	0.48	-5.77	8.10E-09	5.02E-07
Rv1613	tryptophan synthase subunit alpha(trpA)	2130.97	-2.87	0.48	-6.02	1.72E-09	1.52E-07
Rv1144	oxidoreductase(Rv1144)	2213.88	-2.89	0.59	-4.89	1.03E-06	2.32E-05
Rv1612	tryptophan synthase subunit beta(trpB)	2004.76	-2.97	0.56	-5.30	1.13E-07	4.23E-06
Rv1360	oxidoreductase(Rv1360)	1175.95	-3.01	0.50	-6.04	1.57E-09	1.44E-07

	Rv0694	mycofactocin system heme/flavin oxidoreductase MftD(Rv0694)	5304.24	-3.11	0.48	-6.52	6.90E-11	1.14E-08
	Rv2949c	chorismate pyruvate-lyase(Rv2949c)	571.06	-4.66	0.51	-9.14	6.52E-20	6.46E-17
	Rv3465	dTDP-4-dehydrorhamnose 3,5-epimerase(rmlC)	778.74	-1.74	0.41	-4.25	2.13E-05	2.56E-04
	Rv2456c	MFS-type transporter(Rv2456c)	1265.13	-1.74	0.33	-5.21	1.85E-07	6.21E-06
	Rv0208c	tRNA (guanine-N(7)-)-methyltransferase(Rv0208c)	886.97	-1.76	0.33	-5.29	1.22E-07	4.45E-06
	Rv1882c	short-chain type dehydrogenase/reductase(Rv1882c)	961.50	-1.86	0.35	-5.36	8.33E-08	3.27E-06
	Rv2957	PGL/p-HBAD biosynthesis glycosyltransferase(Rv2957)	777.12	-2.35	0.41	-5.75	8.76E-09	5.34E-07
	Rv0691A	mycofactocin precursor(Rv0691A)	168.42	-2.39	0.66	-3.64	2.75E-04	1.95E-03
	Rv2457c	ATP-dependent CLP protease ATP-binding subunit ClpX(clpX)	9060.33	-2.54	0.42	-6.07	1.26E-09	1.24E-07
**lipid metabolism**	Rv2930	fatty-acid--CoA ligase FadD26(fadD26)	11586.40	-1.02	0.38	-2.66	7.88E-03	2.69E-02
	Rv1483	3-oxoacyl-ACP reductase FabG(fabG1)	1152.93	-1.04	0.34	-3.10	1.95E-03	9.09E-03
	Rv3234c	diacyglycerol O-acyltransferase(tgs3)	799.98	-1.05	0.26	-4.04	5.43E-05	5.42E-04
	Rv1425	diacyglycerol O-acyltransferase(Rv1425)	1295.07	-1.07	0.25	-4.25	2.17E-05	2.57E-04
	Rv1618	acyl-CoA thioesterase II(tesB1)	825.04	-1.07	0.29	-3.64	2.75E-04	1.95E-03
	Rv0146	S-adenosylmethionine-dependent methyltransferase(Rv0146)	1736.06	-1.09	0.41	-2.64	8.25E-03	2.80E-02
	Rv2941	long-chain-fatty-acid--AMP ligase FadD28(fadD28)	7914.98	-1.12	0.40	-2.78	5.48E-03	2.05E-02
	Rv0404	long-chain-fatty-acid--AMP ligase FadD30(fadD30)	601.79	-1.12	0.41	-2.75	5.90E-03	2.16E-02
	Rv0905	enoyl-CoA hydratase EchA6(echA6)	1070.75	-1.15	0.25	-4.60	4.18E-06	7.06E-05
	Rv1185c	fatty-acid--CoA ligase FadD21(fadD21)	3466.89	-1.21	0.48	-2.50	1.23E-02	3.85E-02
	Rv2377c	hypothetical protein(Rv2377c)	143.25	-1.22	0.45	-2.68	7.35E-03	2.55E-02
	Rv0469	mycolic acid synthase UmaA(umaA)	1158.23	-1.22	0.44	-2.78	5.41E-03	2.03E-02
	Rv3281	bifunctional protein acetyl-/propionyl-CoAcarboxylase subunit epsilon AccE(accE5)	1060.42	-1.22	0.34	-3.54	4.07E-04	2.66E-03
	Rv1679	acyl-CoA dehydrogenase FadE16(fadE16)	1201.98	-1.25	0.36	-3.50	4.68E-04	2.96E-03
	Rv3804c	diacylglycerol acyltransferase/mycolyltransferase Ag85A(fbpA)	8355.55	-1.29	0.30	-4.24	2.19E-05	2.59E-04
	Rv3391	acyl-CoA-reductase AcrA(acrA1)	805.42	-1.30	0.34	-3.85	1.17E-04	1.02E-03
	Rv1543	oxidoreductase(Rv1543)	1421.92	-1.33	0.33	-4.00	6.21E-05	6.08E-04

	Rv3280	propionyl-CoA carboxylase subunit beta(accD5)	2683.65	-1.37	0.36	-3.83	1.26E-04	1.07E-03
	Rv0222	enoyl-CoA hydratase EchA1(echA1)	772.41	-1.43	0.35	-4.08	4.60E-05	4.84E-04
	Rv0642c	hydroxymycolate synthase MmaA4(mmaA4)	2109.89	-1.51	0.38	-4.00	6.33E-05	6.19E-04
	Rv3797	acyl-CoA dehydrogenase FadE35(fadE35)	766.45	-1.58	0.56	-2.83	4.71E-03	1.81E-02
	Rv1544	ketoacyl reductase(Rv1544)	1108.80	-1.65	0.29	-5.68	1.37E-08	7.74E-07
	Rv0643c	methoxy mycolic acid synthase MmaA3(mmaA3)	829.03	-1.68	0.43	-3.92	9.04E-05	8.16E-04
	Rv3523	lipid carrier protein(ltp3)	1692.82	-1.74	0.34	-5.11	3.19E-07	9.58E-06
	Rv3229c	stearoyl-CoA 9-desaturase(desA3)	10572.73	-1.78	0.57	-3.13	1.77E-03	8.43E-03
	Rv3371	diacyglycerol O-acyltransferase(Rv3371)	2978.67	-1.81	0.51	-3.56	3.74E-04	2.49E-03
	Rv2939	phthiocerol/phthiodiolone dimycocerosyl transferase(papA5)	2969.27	-1.82	0.36	-5.02	5.20E-07	1.39E-05
	Rv1094	acyl-ACP desaturase DesA(desA2)	24399.85	-1.88	0.36	-5.20	2.01E-07	6.59E-06
	Rv0824c	acyl-ACP desaturase DesA(desA1)	16003.30	-1.93	0.38	-5.11	3.19E-07	9.58E-06
	Rv1521	fatty-acid--CoA ligase FadD25(fadD25)	2828.82	-1.93	0.45	-4.29	1.82E-05	2.28E-04
	Rv3392c	cyclopropane mycolic acid synthase CmaA(cmaA1)	251.48	-2.05	0.46	-4.47	7.85E-06	1.15E-04
	Rv0221	diacyglycerol O-acyltransferase(Rv0221)	1622.18	-2.07	0.38	-5.42	6.00E-08	2.61E-06
	Rv2947c	polyketide synthase(pks15)	674.12	-2.13	0.52	-4.13	3.68E-05	4.06E-04
	Rv2953	trans-acting enoyl reductase(Rv2953)	1003.16	-2.27	0.51	-4.44	9.08E-06	1.31E-04
	Rv2948c	p-hydroxybenzoyl--AMP ligase(fadD22)	1423.22	-3.37	0.48	-7.02	2.28E-12	6.45E-10
	Rv0166	fatty-acid--CoA ligase FadD5(fadD5)	1998.28	-3.40	0.62	-5.49	4.04E-08	1.88E-06
	Rv2950c	long-chain-fatty-acid--AMP ligase FadD29(fadD29)	1979.66	-3.75	0.42	-8.85	8.40E-19	6.66E-16
	Rv0503c	cyclopropane mycolic acid synthase(cmaA2)	1459.97	-1.76	0.36	-4.94	8.00E-07	1.97E-05
**virulence, detoxification, adaptation**	Rv2104c	antitoxin VapB37(vapB37)	317.39	-1.03	0.40	-2.56	1.05E-02	3.41E-02
	Rv2103c	ribonuclease VapC37(vapC37)	553.26	-1.04	0.25	-4.13	3.63E-05	4.02E-04
	Rv0798c	hypothetical protein(cfp29)	405.96	-1.06	0.36	-2.93	3.44E-03	1.42E-02
	Rv3171c	non-heme haloperoxidase Hpx(hpx)	3019.47	-1.07	0.24	-4.54	5.56E-06	8.75E-05
	Rv3758c	glycine betaine/carnitine/choline/L-proline ABC transporter ATP-binding protein ProV(proV)	1472.25	-1.09	0.44	-2.47	1.33E-02	4.11E-02
	Rv2530c	ribonuclease VapC39(vapC39)	241.17	-1.11	0.29	-3.80	1.45E-04	1.19E-03

	Rv0598c	ribonuclease VapC27(vapC27)	741.74	-1.13	0.34	-3.35	8.12E-04	4.60E-03
	Rv1982c	ribonuclease VapC36(vapC36)	660.58	-1.17	0.35	-3.34	8.23E-04	4.63E-03
	Rv0432	superoxide dismutase(sodC)	1168.46	-1.19	0.33	-3.63	2.89E-04	2.01E-03
	Rv3757c	glycine betaine/carnitine/choline/L-proline ABC transporter permease ProW(proW)	797.24	-1.25	0.51	-2.44	1.48E-02	4.49E-02
	Rv3846	superoxide dismutase(sodA)	732.94	-1.26	0.46	-2.74	6.05E-03	2.20E-02
	Rv1478	peptidoglycan endopeptidase RipB(Rv1478)	1226.78	-1.32	0.46	-2.85	4.42E-03	1.72E-02
	Rv0171	Mce family protein Mce1C(mce1C)	4678.51	-1.32	0.39	-3.38	7.12E-04	4.14E-03
	Rv3648c	cold shock protein A(cspA)	12178.29	-1.33	0.46	-2.89	3.87E-03	1.55E-02
	Rv2006	trehalose-6-phosphate phosphatase OtsB(otsB1)	9357.66	-1.34	0.53	-2.52	1.18E-02	3.74E-02
	Rv1477	peptidoglycan endopeptidase RipA(ripA)	3315.28	-1.36	0.48	-2.81	4.97E-03	1.89E-02
	Rv3756c	glycine betaine/carnitine/choline/L-proline ABC transporter permease ProZ(proZ)	740.99	-1.40	0.44	-3.20	1.39E-03	7.00E-03
	Rv2530A	antitoxin VapB39(vapB39)	114.34	-1.43	0.41	-3.45	5.51E-04	3.38E-03
	Rv2238c	peroxiredoxin(ahpE)	789.05	-1.50	0.37	-4.10	4.16E-05	4.45E-04
	Rv0959A	antitoxin VapB9(vapB9)	200.80	-1.54	0.62	-2.48	1.30E-02	4.04E-02
	Rv2028c	universal stress protein(Rv2028c)	3009.40	-1.57	0.52	-3.05	2.30E-03	1.02E-02
	Rv3490	trehalose-phosphate synthase(otsA)	11926.99	-1.60	0.44	-3.63	2.85E-04	2.01E-03
	Rv3134c	universal stress protein(Rv3134c)	10775.02	-1.73	0.55	-3.17	1.50E-03	7.44E-03
	Rv0960	ribonuclease VapC9(vapC9)	720.49	-1.76	0.46	-3.81	1.37E-04	1.15E-03
	Rv0170	Mce family protein Mce1B(mce1B)	2110.66	-1.96	0.48	-4.10	4.22E-05	4.49E-04
	Rv0168	membrane protein(yrbE1B)	1147.87	-2.19	0.53	-4.12	3.72E-05	4.09E-04
	Rv0172	Mce family protein Mce1D(mce1D)	4243.22	-2.24	0.53	-4.24	2.27E-05	2.67E-04
	Rv0169	Mce family protein Mce1A(mce1A)	2585.58	-2.27	0.52	-4.37	1.24E-05	1.70E-04
	Rv0174	Mce family protein Mce1F(mce1F)	8065.82	-2.29	0.39	-5.91	3.41E-09	2.50E-07
	Rv2031c	alpha-crystallin(hspX)	19457.73	-2.48	0.74	-3.34	8.41E-04	4.72E-03
	Rv0167	membrane protein(yrbE1A)	2437.49	-2.55	0.53	-4.84	1.27E-06	2.77E-05
	Rv3922c	membrane protein insertion efficiency factor(Rv3922c)	601.67	-2.60	0.52	-5.03	4.96E-07	1.35E-05
	Rv3320c	ribonuclease VapC44(vapC44)	482.88	-1.85	0.50	-3.67	2.40E-04	1.76E-03

**regulatory proteins**	Rv1626	two-component system transcriptional regulator(Rv1626)	1655.23	-1.03	0.36	-2.90	3.74E-03	1.51E-02
	Rv0158	transcriptional regulator(Rv0158)	377.63	-1.25	0.32	-3.88	1.06E-04	9.38E-04
	Rv0042c	transcriptional regulator(Rv0042c)	338.19	-1.26	0.38	-3.32	9.00E-04	4.96E-03
	Rv2720	repressor LexA(lexA)	3780.29	-1.34	0.38	-3.57	3.54E-04	2.38E-03
	Rv3246c	two component DNA-binding response regulator MtrA(mtrA)	3973.98	-1.36	0.35	-3.84	1.23E-04	1.05E-03
	Rv3295	TetR family transcriptional regulator(Rv3295)	2891.34	-1.39	0.36	-3.85	1.18E-04	1.02E-03
	Rv0981	two-component response regulator MrpA(mprA)	1793.87	-1.54	0.32	-4.82	1.43E-06	3.06E-05
	Rv3143	response regulator(Rv3143)	259.98	-1.62	0.34	-4.71	2.46E-06	4.58E-05
	Rv0823c	tRNA-dihydrouridine synthase(Rv0823c)	13292.20	-1.84	0.37	-4.92	8.86E-07	2.12E-05
	Rv3132c	two component sensor histidine kinase DevS(devS)	7870.11	-2.15	0.54	-3.94	8.05E-05	7.55E-04
	Rv3133c	two component transcriptional regulator DevR(devR)	4814.85	-2.27	0.54	-4.20	2.66E-05	3.08E-04
	Rv1534	transcriptional regulator(Rv1534)	1011.45	-2.66	0.45	-5.93	3.06E-09	2.38E-07
	Rv1830	HTH-type transcriptional regulator(Rv1830)	1718.89	-1.74	0.40	-4.37	1.26E-05	1.72E-04
	Rv0165c	transcriptional regulator Mce1R(mce1R)	1090.80	-1.90	0.38	-4.97	6.52E-07	1.66E-05
**information pathways**	Rv3646c	DNA topoisomerase I(topA)	4717.88	-1.03	0.30	-3.39	6.97E-04	4.07E-03
	Rv0003	DNA replication/repair protein RecF(recF)	671.26	-1.03	0.35	-2.98	2.89E-03	1.24E-02
	Rv0058	replicative DNA helicase(dnaB)	4185.72	-1.06	0.31	-3.45	5.66E-04	3.45E-03
	Rv1015c	50S ribosomal protein L25/general stress protein Ctc(rplY)	761.71	-1.07	0.35	-3.10	1.90E-03	8.91E-03
	Rv1080c	transcription elongation factor GreA(greA)	5451.91	-1.10	0.36	-3.08	2.04E-03	9.34E-03
	Rv0634B	50S ribosomal protein L33(rpmG2)	258.43	-1.11	0.44	-2.55	1.09E-02	3.49E-02
	Rv0054	single-strand DNA-binding protein(ssb)	1267.73	-1.13	0.26	-4.33	1.50E-05	1.98E-04
	Rv2101	helicase HelZ(helZ)	3350.98	-1.14	0.33	-3.42	6.18E-04	3.70E-03
	Rv0053	30S ribosomal protein S6(rpsF)	678.01	-1.15	0.40	-2.85	4.39E-03	1.71E-02
	Rv3414c	ECF RNA polymerase sigma factor SigD(sigD)	1563.14	-1.16	0.47	-2.47	1.34E-02	4.14E-02
	Rv3048c	ribonucleoside-diphosphate reductase subunit beta NrdF2(nrdF2)	3238.11	-1.17	0.37	-3.14	1.67E-03	8.09E-03
	Rv1547	DNA polymerase III subunit alpha(dnaE1)	3731.02	-1.22	0.29	-4.28	1.86E-05	2.30E-04
	Rv1388	integration host factor MihF(mihF)	3282.37	-1.25	0.35	-3.55	3.79E-04	2.52E-03

Rv2069	ECF RNA polymerase sigma factor SigC(sigC)	406.23	-1.26	0.35	-3.61	3.08E-04	2.12E-03
Rv0685	elongation factor Tu(tuf)	11036.14	-1.29	0.47	-2.77	5.64E-03	2.09E-02
Rv2845c	proline--tRNA ligase(proS)	2901.04	-1.31	0.51	-2.57	1.03E-02	3.35E-02
Rv2992c	glutamate--tRNA ligase(gltS)	1108.21	-1.37	0.37	-3.73	1.94E-04	1.50E-03
Rv2442c	50S ribosomal protein L21(rplU)	623.52	-1.38	0.51	-2.72	6.55E-03	2.34E-02
Rv0937c	non-homologous end joining protein Ku(mku)	602.04	-1.39	0.40	-3.45	5.61E-04	3.43E-03
Rv3687c	anti-anti-sigma factor RsfB(rsfB)	284.83	-1.42	0.32	-4.41	1.01E-05	1.43E-04
Rv1097c	hypothetical protein(Rv1097c)	1331.57	-1.44	0.34	-4.26	2.08E-05	2.51E-04
Rv1644	23S rRNA methyltransferase TsnR(tsnR)	6323.49	-1.46	0.39	-3.70	2.14E-04	1.60E-03
Rv2902c	ribonuclease HII(rnhB)	3425.93	-1.47	0.38	-3.81	1.39E-04	1.16E-03
Rv0009	iron-regulated peptidyl-prolyl cis-trans isomerase PpiA(ppiA)	2051.71	-1.47	0.46	-3.16	1.57E-03	7.65E-03
Rv2736c	regulatory protein RecX(recX)	848.61	-1.48	0.41	-3.60	3.20E-04	2.19E-03
Rv2904c	50S ribosomal protein L19(rplS)	2671.59	-1.49	0.29	-5.19	2.15E-07	6.88E-06
Rv0002	DNA polymerase III subunit beta(dnaN)	1026.49	-1.51	0.52	-2.92	3.54E-03	1.45E-02
Rv0979A	50S ribosomal protein L32(rpmF)	184.46	-1.51	0.39	-3.85	1.18E-04	1.02E-03
Rv0056	50S ribosomal protein L9(rplI)	745.84	-1.51	0.35	-4.28	1.85E-05	2.30E-04
Rv1108c	exodeoxyribonuclease VII large subunit(xseA)	2883.59	-1.53	0.46	-3.28	1.03E-03	5.53E-03
Rv0723	50S ribosomal protein L15(rplO)	3185.85	-1.53	0.43	-3.56	3.68E-04	2.46E-03
Rv2737c	recombinase A(recA)	7287.42	-1.55	0.39	-3.93	8.65E-05	7.92E-04
Rv2986c	DNA-binding protein HU(hupB)	11870.67	-1.55	0.41	-3.77	1.66E-04	1.33E-03
Rv1641	initiation factor IF-3(infC)	11219.45	-1.56	0.36	-4.34	1.42E-05	1.91E-04
Rv0055	30S ribosomal protein S18(rpsR1)	286.35	-1.58	0.33	-4.74	2.17E-06	4.15E-05
Rv1643	50S ribosomal protein L20(rplT)	6518.75	-1.60	0.36	-4.43	9.49E-06	1.36E-04
Rv0714	50S ribosomal protein L14(rplN)	1323.51	-1.69	0.36	-4.64	3.41E-06	6.06E-05
Rv0715	50S ribosomal protein L24(rplX)	1376.80	-1.69	0.44	-3.80	1.45E-04	1.19E-03
Rv2594c	crossover junction endodeoxyribonuclease RuvC(ruvC)	937.09	-1.74	0.35	-4.90	9.70E-07	2.22E-05
Rv2985	8-oxo-dGTP diphosphatase(mutT1)	1957.78	-1.79	0.27	-6.58	4.65E-11	8.39E-09
Rv0701	50S ribosomal protein L3(rplC)	8771.40	-1.82	0.47	-3.91	9.18E-05	8.24E-04

	Rv3923c	ribonuclease P protein component(rnpA)	805.87	-1.96	0.46	-4.27	1.93E-05	2.37E-04
	Rv0716	50S ribosomal protein L5(rplE)	2386.84	-1.96	0.48	-4.07	4.63E-05	4.86E-04
	Rv1642	50S ribosomal protein L35(rpmI)	3505.44	-1.97	0.36	-5.39	7.04E-08	2.91E-06
	Rv0721	30S ribosomal protein S5(rpsE)	4216.57	-1.98	0.45	-4.42	9.98E-06	1.42E-04
	Rv0702	50S ribosomal protein L4(rplD)	8982.65	-1.99	0.46	-4.30	1.70E-05	2.19E-04
	Rv0720	50S ribosomal protein L18(rplR)	1225.85	-2.03	0.45	-4.56	5.20E-06	8.34E-05
	Rv0708	50S ribosomal protein L16(rplP)	2369.97	-2.06	0.58	-3.57	3.54E-04	2.38E-03
	Rv1630	30S ribosomal protein S1(rpsA)	8673.62	-2.07	0.36	-5.81	6.08E-09	4.16E-07
	Rv0516c	anti-anti-sigma factor(Rv0516c)	1284.45	-2.14	0.52	-4.11	3.88E-05	4.19E-04
	Rv0706	50S ribosomal protein L22(rplV)	3325.76	-2.19	0.59	-3.72	2.01E-04	1.54E-03
	Rv0719	50S ribosomal protein L6(rplF)	3409.41	-2.20	0.47	-4.71	2.44E-06	4.57E-05
	Rv0707	30S ribosomal protein S3(rpsC)	4545.96	-2.24	0.54	-4.19	2.82E-05	3.24E-04
	Rv0722	50S ribosomal protein L30(rpmD)	501.26	-2.41	0.44	-5.43	5.76E-08	2.56E-06
	Rv0704	50S ribosomal protein L2(rplB)	6348.85	-2.56	0.53	-4.82	1.43E-06	3.06E-05
	Rv0705	30S ribosomal protein S19(rpsS)	2649.95	-2.60	0.52	-4.99	6.09E-07	1.59E-05
	Rv0718	30S ribosomal protein S8(rpsH)	1994.08	-2.66	0.44	-6.06	1.35E-09	1.31E-07
	Rv1107c	exodeoxyribonuclease VII small subunit(xseB)	306.04	-2.76	0.52	-5.35	8.86E-08	3.44E-06
	Rv0717	30S ribosomal protein S14(rpsN1)	625.16	-2.87	0.64	-4.47	7.66E-06	1.13E-04
	Rv3924c	50S ribosomal protein L34(rpmH)	271.99	-1.75	0.50	-3.50	4.73E-04	2.99E-03
	Rv0700	30S ribosomal protein S10(rpsJ)	4164.59	-1.81	0.46	-3.94	8.23E-05	7.66E-04
	Rv0710	30S ribosomal protein S17(rpsQ)	1028.58	-2.37	0.59	-4.01	6.17E-05	6.05E-04
	Rv0709	50S ribosomal protein L29(rpmC)	784.52	-2.41	0.60	-3.98	6.78E-05	6.49E-04
	Rv0703	50S ribosomal protein L23(rplW)	1872.38	-2.44	0.50	-4.87	1.12E-06	2.51E-05
	Rv0001	chromosomal replication initiator protein DnaA(dnaA)	3212.45	-2.54	0.56	-4.57	4.92E-06	8.02E-05
**stable rnas**	B11	ncRNA(B11)	1522.04	-1.06	0.42	-2.56	1.05E-02	3.41E-02
	thrT	tRNA-Thr(thrT)	118.96	-1.42	0.57	-2.47	1.35E-02	4.14E-02
	proY	tRNA-Pro(proY)	87.59	-1.42	0.54	-2.64	8.36E-03	2.82E-02
	metT	tRNA-Met(metT)	99.51	-1.66	0.65	-2.55	1.08E-02	3.46E-02

	ileT	tRNA-Ile(ileT)	44.55	-1.73	0.51	-3.40	6.85E-04	4.01E-03
**PE/PPE**	Rv1646	PE family protein PE17(PE17)	2149.65	-1.05	0.34	-3.08	2.08E-03	9.45E-03
	Rv2328	PE family protein PE23(PE23)	902.00	-1.18	0.43	-2.77	5.67E-03	2.10E-02
	Rv1788	PE family protein PE18(PE18)	165.46	-1.22	0.28	-4.37	1.23E-05	1.69E-04
	Rv3144c	PPE family protein PPE52(PPE52)	1522.06	-1.34	0.46	-2.93	3.35E-03	1.39E-02
	Rv2431c	PE family protein PE25(PE25)	1945.73	-1.34	0.46	-2.93	3.42E-03	1.41E-02
	Rv1791	PE family protein PE19(PE19)	836.46	-1.42	0.38	-3.76	1.68E-04	1.35E-03
	Rv1441c	PE-PGRS family protein PE_PGRS26(PE_PGRS26)	873.61	-1.42	0.47	-3.03	2.48E-03	1.09E-02
	Rv1361c	PPE family protein PPE19(PPE19)	3912.17	-1.54	0.57	-2.69	7.10E-03	2.48E-02
	Rv1387	PPE family protein PPE20(PPE20)	13943.66	-1.55	0.32	-4.86	1.20E-06	2.64E-05
	Rv3135	PPE family protein PPE50(PPE50)	1900.36	-1.66	0.54	-3.06	2.22E-03	9.92E-03
	Rv2430c	PPE family protein PPE41(PPE41)	2278.86	-1.73	0.31	-5.65	1.64E-08	9.14E-07
	Rv1386	PE family protein PE15(PE15)	1727.83	-1.87	0.40	-4.73	2.25E-06	4.25E-05
	Rv3425	PPE family protein PPE57(PPE57)	514.73	-2.12	0.55	-3.82	1.34E-04	1.13E-03
	Rv1196	PPE family protein PPE18(PPE18)	11926.53	-2.43	0.50	-4.83	1.34E-06	2.89E-05
	Rv1195	PE family protein PE13(PE13)	4268.93	-3.58	0.54	-6.69	2.24E-11	4.45E-09
**conserved hypotheticals**	Rv0910	toxin(Rv0910)	784.12	-1.00	0.37	-2.74	6.22E-03	2.25E-02
	Rv2927c	hypothetical protein(Rv2927c)	2098.17	-1.01	0.42	-2.40	1.63E-02	4.85E-02
	Rv0138	hypothetical protein(Rv0138)	217.25	-1.01	0.34	-2.95	3.14E-03	1.32E-02
	Rv2735c	hypothetical protein(Rv2735c)	194.93	-1.02	0.36	-2.84	4.45E-03	1.72E-02
	Rv1111c	hypothetical protein(Rv1111c)	1156.97	-1.02	0.22	-4.55	5.36E-06	8.56E-05
	Rv0786c	hypothetical protein(Rv0786c)	120.78	-1.02	0.40	-2.58	9.81E-03	3.24E-02
	Rv2102	hypothetical protein(Rv2102)	1151.43	-1.03	0.27	-3.77	1.62E-04	1.31E-03
	Rv0911	hypothetical protein(Rv0911)	474.36	-1.06	0.29	-3.63	2.86E-04	2.01E-03
	Rv3030	S-adenosylmethionine-dependent methyltransferase(Rv3030)	531.77	-1.08	0.44	-2.45	1.43E-02	4.35E-02
	Rv2955c	hypothetical protein(Rv2955c)	529.09	-1.10	0.35	-3.16	1.60E-03	7.76E-03
	Rv2432c	hypothetical protein(Rv2432c)	504.69	-1.10	0.45	-2.45	1.45E-02	4.39E-02
	Rv3142c	hypothetical protein(Rv3142c)	674.31	-1.10	0.38	-2.88	4.03E-03	1.60E-02

Rv1507A	hypothetical protein(Rv1507A)	306.90	-1.11	0.40	-2.77	5.57E-03	2.07E-02
Rv1489	hypothetical protein(Rv1489)	896.43	-1.13	0.29	-3.89	1.02E-04	8.99E-04
Rv0052	hypothetical protein(Rv0052)	395.08	-1.13	0.25	-4.52	6.20E-06	9.56E-05
Rv3705A	hypothetical protein(Rv3705A)	337.98	-1.14	0.39	-2.90	3.68E-03	1.49E-02
Rv1171	hypothetical protein(Rv1171)	844.05	-1.14	0.36	-3.13	1.77E-03	8.43E-03
Rv0295c	hypothetical protein(Rv0295c)	835.06	-1.15	0.29	-3.95	7.81E-05	7.34E-04
Rv3661	hypothetical protein(Rv3661)	1089.60	-1.15	0.28	-4.18	2.92E-05	3.34E-04
Rv3688c	hypothetical protein(Rv3688c)	406.84	-1.15	0.31	-3.70	2.14E-04	1.60E-03
Rv1693	hypothetical protein(Rv1693)	73.62	-1.16	0.43	-2.72	6.52E-03	2.33E-02
Rv2327	hypothetical protein(Rv2327)	2571.13	-1.17	0.29	-4.05	5.11E-05	5.17E-04
Rv2468A	hypothetical protein(Rv2468A)	181.35	-1.17	0.43	-2.72	6.61E-03	2.35E-02
Rv2239c	hypothetical protein(Rv2239c)	894.87	-1.19	0.39	-3.02	2.49E-03	1.09E-02
Rv3678c	hypothetical protein(Rv3678c)	1076.92	-1.19	0.32	-3.68	2.30E-04	1.70E-03
Rv0264c	hypothetical protein(Rv0264c)	523.60	-1.21	0.32	-3.74	1.81E-04	1.42E-03
Rv1558	hypothetical protein(Rv1558)	453.47	-1.21	0.33	-3.68	2.33E-04	1.72E-03
Rv0634A	hypothetical protein(Rv0634A)	421.77	-1.21	0.50	-2.40	1.66E-02	4.91E-02
Rv2425c	hypothetical protein(Rv2425c)	940.38	-1.24	0.40	-3.06	2.21E-03	9.86E-03
Rv3702c	amidohydrolase EgtC(egtC)	565.84	-1.24	0.47	-2.64	8.30E-03	2.81E-02
Rv2567	hypothetical protein(Rv2567)	1528.23	-1.25	0.31	-4.06	4.93E-05	5.08E-04
Rv0207c	hypothetical protein(Rv0207c)	2347.15	-1.26	0.26	-4.86	1.20E-06	2.64E-05
Rv3365c	hypothetical protein(Rv3365c)	4898.07	-1.26	0.50	-2.55	1.08E-02	3.46E-02
Rv2172c	hypothetical protein(Rv2172c)	4135.49	-1.28	0.42	-3.05	2.32E-03	1.03E-02
Rv2190c	endopeptidase(Rv2190c)	5312.52	-1.29	0.46	-2.79	5.27E-03	1.99E-02
Rv0698	hypothetical protein(Rv0698)	338.66	-1.30	0.39	-3.33	8.61E-04	4.79E-03
Rv1444c	hypothetical protein(Rv1444c)	363.83	-1.31	0.28	-4.63	3.69E-06	6.50E-05
Rv1513	hypothetical protein(Rv1513)	388.58	-1.32	0.37	-3.54	4.07E-04	2.66E-03
Rv1676	hypothetical protein(Rv1676)	1157.06	-1.33	0.40	-3.33	8.82E-04	4.88E-03
Rv0059	hypothetical protein(Rv0059)	588.55	-1.33	0.38	-3.49	4.78E-04	3.00E-03

Rv0455c	hypothetical protein(Rv0455c)	1404.49	-1.33	0.34	-3.91	9.12E-05	8.22E-04
Rv0434	hypothetical protein(Rv0434)	809.79	-1.33	0.27	-5.02	5.07E-07	1.37E-05
Rv3491	hypothetical protein(Rv3491)	1705.98	-1.34	0.34	-3.90	9.55E-05	8.53E-04
Rv1287	HTH-type transcriptional regulator(Rv1287)	1478.93	-1.34	0.50	-2.70	6.94E-03	2.44E-02
Rv3489	hypothetical protein(Rv3489)	1094.01	-1.34	0.51	-2.62	8.78E-03	2.94E-02
Rv0108c	hypothetical protein(Rv0108c)	2038.58	-1.35	0.48	-2.80	5.14E-03	1.94E-02
Rv2680	hypothetical protein(Rv2680)	1120.72	-1.37	0.39	-3.48	5.01E-04	3.11E-03
Rv3811	hypothetical protein(Rv3811)	2855.51	-1.40	0.34	-4.16	3.16E-05	3.58E-04
Rv3492c	Mce associated protein(Rv3492c)	420.25	-1.41	0.35	-4.04	5.32E-05	5.35E-04
Rv0074	hypothetical protein(Rv0074)	933.91	-1.43	0.39	-3.69	2.29E-04	1.69E-03
Rv2901c	hypothetical protein(Rv2901c)	1384.22	-1.43	0.32	-4.47	7.95E-06	1.16E-04
Rv2929	hypothetical protein(Rv2929)	175.37	-1.43	0.53	-2.70	6.86E-03	2.42E-02
Rv3362c	ATP/GTP-binding protein(Rv3362c)	417.69	-1.45	0.52	-2.77	5.61E-03	2.09E-02
Rv0430	hypothetical protein(Rv0430)	340.93	-1.48	0.61	-2.42	1.57E-02	4.71E-02
Rv0909	antitoxin(Rv0909)	237.54	-1.48	0.42	-3.53	4.14E-04	2.68E-03
Rv1321	endonuclease NucS(Rv1321)	1160.85	-1.51	0.19	-7.84	4.46E-15	1.96E-12
Rv0502	hypothetical protein(Rv0502)	6161.64	-1.51	0.31	-4.93	8.20E-07	1.98E-05
Rv2375	hypothetical protein(Rv2375)	578.83	-1.51	0.31	-4.91	9.20E-07	2.17E-05
Rv2313c	hypothetical protein(Rv2313c)	767.27	-1.52	0.37	-4.12	3.76E-05	4.09E-04
Rv2148c	hypothetical protein(Rv2148c)	887.92	-1.53	0.32	-4.78	1.78E-06	3.59E-05
Rv3210c	hypothetical protein(Rv3210c)	641.44	-1.54	0.34	-4.55	5.39E-06	8.56E-05
Rv3131	NAD(P)H nitroreductase(Rv3131)	26290.36	-1.55	0.56	-2.75	5.94E-03	2.17E-02
Rv2956	hypothetical protein(Rv2956)	806.55	-1.56	0.49	-3.16	1.57E-03	7.65E-03
Rv0433	carboxylate-amine ligase(Rv0433)	1140.48	-1.57	0.31	-5.10	3.45E-07	1.01E-05
Rv1506c	hypothetical protein(Rv1506c)	143.77	-1.58	0.48	-3.29	9.92E-04	5.38E-03
Rv2386a	hypothetical protein(Rv2386a)	477.18	-1.58	0.45	-3.49	4.83E-04	3.02E-03
Rv3701c	histidine-specific methyltransferase EtgD(egtD)	938.47	-1.58	0.45	-3.52	4.33E-04	2.78E-03
Rv2336	hypothetical protein(Rv2336)	527.69	-1.60	0.53	-3.03	2.47E-03	1.08E-02

Rv2137c	hypothetical protein(Rv2137c)	2513.79	-1.60	0.51	-3.11	1.89E-03	8.87E-03
Rv3364c	hypothetical protein(Rv3364c)	231.15	-1.60	0.50	-3.21	1.31E-03	6.72E-03
Rv1518	hypothetical protein(Rv1518)	149.28	-1.60	0.47	-3.38	7.37E-04	4.26E-03
Rv3705c	hypothetical protein(Rv3705c)	830.63	-1.61	0.46	-3.47	5.29E-04	3.26E-03
Rv3363c	hypothetical protein(Rv3363c)	187.65	-1.62	0.56	-2.86	4.17E-03	1.64E-02
Rv1057	hypothetical protein(Rv1057)	2279.46	-1.63	0.50	-3.26	1.10E-03	5.85E-03
Rv2778c	hypothetical protein(Rv2778c)	454.09	-1.64	0.38	-4.28	1.84E-05	2.29E-04
Rv1006	hypothetical protein(Rv1006)	4104.94	-1.64	0.34	-4.79	1.66E-06	3.41E-05
Rv3361c	hypothetical protein(Rv3361c)	364.53	-1.64	0.44	-3.72	2.02E-04	1.54E-03
Rv0997a	hypothetical protein(Rv0997a)	428.86	-1.66	0.44	-3.80	1.44E-04	1.19E-03
Rv0647c	hypothetical protein(Rv0647c)	1437.14	-1.68	0.34	-4.96	7.17E-07	1.79E-05
Rv3612c	hypothetical protein(Rv3612c)	837.23	-1.69	0.61	-2.77	5.55E-03	2.07E-02
Rv3241c	hypothetical protein(Rv3241c)	1866.25	-1.69	0.34	-4.98	6.46E-07	1.65E-05
Rv1505c	hypothetical protein(Rv1505c)	203.03	-1.71	0.39	-4.39	1.11E-05	1.55E-04
Rv1322A	hypothetical protein(Rv1322A)	9536.35	-1.71	0.42	-4.06	5.00E-05	5.11E-04
Rv0907	hypothetical protein(Rv0907)	776.38	-1.71	0.30	-5.62	1.93E-08	1.02E-06
Rv1507c	hypothetical protein(Rv1507c)	280.32	-1.74	0.48	-3.63	2.84E-04	2.00E-03
Rv2426c	hypothetical protein(Rv2426c)	834.40	-1.75	0.36	-4.82	1.47E-06	3.10E-05
Rv2067c	hypothetical protein(Rv2067c)	658.20	-1.77	0.39	-4.57	4.80E-06	7.90E-05
Rv0997	hypothetical protein(Rv0997)	537.94	-1.78	0.33	-5.43	5.57E-08	2.51E-06
Rv3749c	hypothetical protein(Rv3749c)	321.74	-1.78	0.36	-4.90	9.36E-07	2.18E-05
Rv1998c	hypothetical protein(Rv1998c)	1336.94	-1.79	0.54	-3.31	9.38E-04	5.13E-03
Rv1322	hypothetical protein(Rv1322)	1637.16	-1.80	0.36	-4.97	6.77E-07	1.71E-05
Rv3190A	hypothetical protein(Rv3190A)	484.90	-1.82	0.34	-5.37	7.95E-08	3.15E-06
Rv0057	hypothetical protein(Rv0057)	573.48	-1.83	0.42	-4.37	1.22E-05	1.68E-04
Rv3209	hypothetical protein(Rv3209)	1088.55	-1.84	0.44	-4.15	3.35E-05	3.77E-04
Rv1632c	hypothetical protein(Rv1632c)	683.88	-1.86	0.30	-6.30	2.91E-10	3.85E-08
Rv3662c	hypothetical protein(Rv3662c)	6524.45	-1.90	0.62	-3.07	2.11E-03	9.53E-03

Rv1904	hypothetical protein(Rv1904)	1315.15	-1.91	0.40	-4.76	1.89E-06	3.77E-05
Rv0695	mycofactocin system creatinine amidohydrolase family protein MftE(Rv0695)	2824.01	-1.92	0.40	-4.76	1.97E-06	3.88E-05
Rv3376	phosphatase(Rv3376)	1259.64	-1.95	0.35	-5.54	2.94E-08	1.48E-06
Rv3613c	hypothetical protein(Rv3613c)	826.72	-1.95	0.66	-2.98	2.92E-03	1.25E-02
Rv3127	hypothetical protein(Rv3127)	12905.14	-1.96	0.62	-3.14	1.70E-03	8.18E-03
Rv0822c	hypothetical protein(Rv0822c)	5533.30	-1.99	0.37	-5.41	6.36E-08	2.66E-06
Rv1738	hypothetical protein(Rv1738)	4691.11	-2.01	0.63	-3.17	1.53E-03	7.53E-03
Rv3484	hypothetical protein(Rv3484)	2601.96	-2.08	0.30	-6.84	8.13E-12	2.01E-09
Rv2387	hypothetical protein(Rv2387)	1107.30	-2.09	0.37	-5.63	1.75E-08	9.52E-07
Rv1109c	hypothetical protein(Rv1109c)	3053.63	-2.09	0.46	-4.54	5.76E-06	8.97E-05
Rv0831c	hypothetical protein(Rv0831c)	2090.96	-2.10	0.40	-5.26	1.40E-07	4.93E-06
Rv0177	Mce associated protein(Rv0177)	1363.62	-2.11	0.38	-5.54	3.07E-08	1.52E-06
Rv0164	hypothetical protein(TB18.5)	627.41	-2.12	0.41	-5.19	2.08E-07	6.77E-06
Rv1794	hypothetical protein(Rv1794)	3497.97	-2.13	0.39	-5.52	3.43E-08	1.66E-06
Rv2275	cyclo(L-tyrosyl-L-tyrosyl) synthase(Rv2275)	224.64	-2.15	0.69	-3.11	1.89E-03	8.87E-03
Rv2817c	CRISPR-associated endonuclease Cas1(Rv2817c)	995.05	-2.16	0.77	-2.80	5.06E-03	1.92E-02
Rv1433	hypothetical protein(Rv1433)	650.99	-2.18	0.44	-4.99	5.93E-07	1.57E-05
Rv2348c	hypothetical protein(Rv2348c)	1217.75	-2.36	0.60	-3.93	8.58E-05	7.91E-04
Rv3706c	hypothetical protein(Rv3706c)	587.02	-2.37	0.51	-4.62	3.82E-06	6.64E-05
Rv1871c	hypothetical protein(Rv1871c)	7818.68	-2.39	0.36	-6.70	2.09E-11	4.37E-09
Rv3005c	hypothetical protein(Rv3005c)	2514.00	-2.58	0.43	-5.96	2.50E-09	1.99E-07
Rv1883c	hypothetical protein(Rv1883c)	2261.60	-2.70	0.55	-4.93	8.18E-07	1.98E-05
Rv1271c	hypothetical protein(Rv1271c)	418.37	-2.74	0.28	-9.71	2.61E-22	3.46E-19
Rv3920c	hypothetical protein(Rv3920c)	2749.92	-2.76	0.41	-6.78	1.20E-11	2.81E-09
Rv1682	hypothetical protein(Rv1682)	645.79	-2.83	0.38	-7.52	5.33E-14	2.12E-11
Rv1870c	hypothetical protein(Rv1870c)	3851.43	-2.84	0.47	-6.03	1.62E-09	1.46E-07
Rv1697	hypothetical protein(Rv1697)	2098.24	-3.12	0.56	-5.62	1.92E-08	1.02E-06

	Rv0692	mycofactocin system protein MftB(Rv0692)	1221.87	-3.46	0.65	-5.30	1.17E-07	4.31E-06
	Rv2954c	hypothetical protein(Rv2954c)	1449.30	-2.28	0.51	-4.50	6.91E-06	1.04E-04
	Rv2147c	cell division protein SepF(Rv2147c)	2414.56	-2.41	0.42	-5.81	6.08E-09	4.16E-07
**Insertion seqs and phages**	Rv3828c	resolvase(Rv3828c)	265.01	-1.05	0.29	-3.62	3.00E-04	2.07E-03
	Rv1042c	IS2-like transposase(Rv1042c)	76.14	-1.14	0.45	-2.54	1.11E-02	3.53E-02
	Rv1701	tyrosine recombinase XerD(Rv1701)	1136.52	-1.16	0.43	-2.69	7.07E-03	2.48E-02
	Rv3191c	transposase(Rv3191c)	1297.87	-1.21	0.36	-3.38	7.19E-04	4.17E-03
	Rv2944	insertion sequence element IS1533 transposase(Rv2944)	148.43	-1.26	0.38	-3.33	8.73E-04	4.85E-03
	Rv0605	IS1536 family serine type transposase(Rv0605)	1047.24	-1.27	0.45	-2.85	4.39E-03	1.71E-02
	Rv3482c	membrane protein(Rv3482c)	619.99	-1.28	0.18	-7.13	1.00E-12	3.06E-10
	Rv3750c	excisionase(Rv3750c)	456.04	-2.08	0.68	-3.07	2.12E-03	9.57E-03

Supplementary Table 4 (I). Differentially expressed genes indicated to be similarly expressed amongst both H37Rv and bedaquiline (BDQ) resistant *Mycobacterium tuberculosis* (Mtb) clinical isolates due to BDQ stress (3.75 µg/ml, t=72 h).
Output analyses for H37Rv and BDQ resistant isolates were obtained by contrasting Mtb isolates after 72 hours of BDQ exposure to their respective controls (unexposed isolates, t= 72 h).

Functional Category	Locus Tag	Genes	BDQ-R clinical isolates (L2Fc)	H37Rv (L2Fc)
**cell wall and cell wall processes (n=127)**	Rv1687c	ABC transporter ATP-binding protein(Rv1687c)	4.95	7.16
	Rv1686c	ABC transporter permease(Rv1686c)	4.59	6.39
	Rv0188	transmembrane protein(Rv0188)	2.26	3.00
	Rv2617c	transmembrane protein(Rv2617c)	2.23	2.08
	Rv0969	copper-exporting ATPase(ctpV)	2.18	1.78
	Rv1992c	cation transporter ATPase G(ctpG)	2.05	1.74
	Rv1463	ABC transporter ATP-binding protein(Rv1463)	1.95	1.78
	Rv3065	multidrug resistance protein Mmr(mmr)	1.85	1.51
	Rv3289c	transmembrane protein(Rv3289c)	1.85	1.40
	Rv2643	arsenic-transport integral membrane protein ArsC(arsC)	1.79	1.80
	Rv1226c	transmembrane protein(Rv1226c)	1.77	2.60

	Rv1227c	transmembrane protein(Rv1227c)	1.61	2.51
	Rv2025c	cation efflux system protein(Rv2025c)	1.52	2.29
	Rv0064	transmembrane protein(Rv0064)	1.52	1.68
	Rv0847	lipoprotein LpqS(lpqS)	1.46	1.89
	Rv1224	Sec-independent protein translocase protein TatB(tatB)	1.39	1.82
	Rv1972	Mce associated membrane protein(Rv1972)	1.37	1.53
	Rv2963	integral membrane protein(Rv2963)	1.30	2.00
	Rv2688c	antibiotic ABC transporter ATP-binding protein(Rv2688c)	1.24	1.75
	Rv0116c	L,D-transpeptidase LdtA(ldtA)	1.15	1.25
	Rv0985c	large-conductance ion mechanosensitive channel(mscL)	1.13	2.05
	Rv1973	Mce associated membrane protein(Rv1973)	1.13	1.85
	Rv0538	membrane protein(Rv0538)	1.09	1.51
	Rv1072	transmembrane protein(Rv1072)	1.06	1.28
	Rv0037c	MFS-type transporter(Rv0037c)	1.01	1.11
	Rv2834c	sn-glycerol-3-phosphate ABC transporter permease UgpE(ugpE)	0.99	1.57
	Rv1980c	immunogenic protein Mpt64(mpt64)	-1.02	-1.22
	Rv1508c	membrane protein(Rv1508c)	-1.02	-1.09
	Rv0732	preprotein translocase SecY(secY)	-1.04	-1.20
	Rv0010c	membrane protein(Rv0010c)	-1.05	-1.84
	Rv2127	L-asparagine permease(ansP1)	-1.07	-1.03
	Rv0011c	cell division protein CrgA(Rv0011c)	-1.08	-1.37
	Rv0431	tuberculin-like peptide(Rv0431)	-1.08	-1.01
	Rv1197	ESAT-6 like protein EsxK(esxK)	-1.09	-3.44
	Rv0403c	membrane protein MmpS1(mmpS1)	-1.13	-1.05
	Rv0178	Mce associated membrane protein(Rv0178)	-1.14	-1.46
	Rv3004	low molecular weight protein antigen 6(cfp6)	-1.15	-1.93
	Rv2128	transmembrane protein(Rv2128)	-1.17	-1.39
	Rv1857	molybdate ABC transporter substrate-binding lipoprotein ModA(modA)	-1.20	-2.41
	Rv0173	Mce family lipoprotein LprK(lprK)	-1.22	-2.15

	Rv1614	prolipoprotein diacylglyceryl transferase(lgt)	-1.24	-2.27
	Rv0713	transmembrane protein(Rv0713)	-1.24	-1.10
	Rv2147c	cell division protein SepF(Rv2147c)	-1.26	-2.41
	Rv2456c	MFS-type transporter(Rv2456c)	-1.26	-1.74
	Rv1440	protein-export membrane protein SecG(secG)	-1.41	-2.29
	Rv1698	copper transporter MctB(mctB)	-1.45	-2.31
	Rv0933	phosphate ABC transporter ATP-binding protein PstB(pstB)	-1.47	-2.24
	Rv3921c	membrane protein insertase YidC(Rv3921c)	-1.52	-2.84
	Rv1884c	resuscitation-promoting factor RpfC(rpfC)	-1.88	-4.16
**intermediary metabolism and respiration (n=78)**	Rv1937	oxygenase(Rv1937)	2.51	2.26
	Rv1936	monooxygenase(Rv1936)	2.51	2.51
	Rv3161c	dioxygenase(Rv3161c)	2.46	2.41
	Rv2501c	acetyl/propionyl-CoA carboxylase subuit alpha(accA1)	2.36	2.01
	Rv3534c	4-hydroxy-2-oxovalerate aldolase(hsaF)	2.26	1.94
	Rv3535c	acetaldehyde dehydrogenase(hsaG)	2.20	1.79
	Rv0983	serine protease PepD(pepD)	2.13	2.09
	Rv3536c	hydratase(hsaE)	2.04	1.56
	Rv1464	cysteine desulfurase(csd)	2.04	1.68
	Rv3203	lipase LipV(lipV)	1.99	2.71
	Rv2497c	3-methyl-2-oxobutanoate dehydrogenase subunit alpha(bkdA)	1.94	1.88
	Rv3290c	L-lysine-epsilon aminotransferase(lat)	1.87	1.58
	Rv2499c	oxidase regulatory-like protein(Rv2499c)	1.87	1.80
	Rv0560c	benzoquinone methyltransferase(Rv0560c)	1.84	2.32
	Rv2496c	3-methyl-2-oxobutanoate dehydrogenase subunit beta(bkdB)	1.82	1.81
	Rv0984	pterin-4-alpha-carbinolamine dehydratase(moaB2)	1.74	2.02
	Rv0848	cysteine synthase CysK(cysK2)	1.73	1.85
	Rv0753c	methylmalonate-semialdehyde dehydrogenase(mmsA)	1.72	1.73
	Rv0751c	3-hydroxyisobutyrate dehydrogenase(mmsB)	1.72	1.51
	Rv0186	beta-glucosidase BglS(bglS)	1.70	1.65
	Rv1654	acetylglutamate kinase(argB)	1.69	1.32

	Rv3206c	adenylyltransferase/sulfurtransferase MoeZ(moeB1)	1.68	1.91
	Rv1465	nitrogen fixation related protein(Rv1465)	1.66	1.39
	Rv2498c	citrate (pro-3S)-lyase subunit beta(citE)	1.60	1.65
	Rv3502c	3-oxoacyl-ACP reductase(Rv3502c)	1.59	2.08
	Rv1653	bifunctional glutamate N-acetyltransferase/amino-acid acetyltransferase(argJ)	1.58	1.34
	Rv3503c	ferredoxin FdxD(fdxD)	1.57	1.94
	Rv1652	N-acetyl-gamma-glutamyl-phoshate reductase(argC)	1.44	1.54
	Rv1655	acetylornithine aminotransferase(argD)	1.43	1.02
	Rv2280	probable dehydrogenase (Rv2280)	1.42	2.03
	Rv2495c	branched-chain keto acid dehydrogenase E2 component(bkdC)	1.41	1.52
	Rv3837c	phosphoglycerate mutase(Rv3837c)	1.41	1.53
	Rv0374c	carbon monoxyde dehydrogenase small subunit(Rv0374c)	1.39	2.86
	Rv1599	histidinol dehydrogenase(hisD)	1.37	1.04
	Rv1832	glycine dehydrogenase(gcvB)	1.28	1.11
	Rv0539	dolichyl-phosphate sugar synthase(Rv0539)	1.28	1.81
	Rv2667	ATP-dependent protease ATP-binding subunit ClpC(clpC2)	1.28	2.07
	Rv0375c	carbon monoxyde dehydrogenase medium subunit(Rv0375c)	1.23	2.67
	Rv1326c	1,4-alpha-glucan branching protein(glgB)	1.22	1.22
	Rv0162c	zinc-type alcohol dehydrogenase subunit E(adhE1)	1.22	1.38
	Rv0846c	oxidase(Rv0846c)	1.21	1.81
	Rv1223	serine protease HtrA(htrA)	1.21	1.74
	Rv2455c	2-oxoglutarate oxidoreductase subunit KorA(Rv2455c)	1.20	1.10
	Rv0077c	oxidoreductase(Rv0077c)	1.19	1.70
	Rv1256c	cytochrome P450 Cyp130(cyp130)	1.15	1.74
	Rv0373c	carbon monoxyde dehydrogenase large subunit(Rv0373c)	1.14	2.55
	Rv2251	flavoprotein(Rv2251)	1.14	1.21
	Rv2852c	malate:quinone oxidoreductase(mqo)	1.13	1.12
	Rv2850c	magnesium chelatase(Rv2850c)	1.13	1.10
	Rv3545c	steroid C26-monooxygenase(cyp125)	1.02	1.26

	Rv2583c	bifunctional (p)ppGpp synthase/hydrolase RelA(relA)	1.00	1.22
	Rv1613	tryptophan synthase subunit alpha(trpA)	-1.00	-2.87
	Rv3145	NADH-quinone oxidoreductase subunit A(nuoA)	-1.00	-2.43
	Rv2993c	2-hydroxyhepta-2,4-diene-1,7-dioate isomerase(Rv2993c)	-1.02	-1.59
	Rv0694	mycofactocin system heme/flavin oxidoreductase MftD(Rv0694)	-1.05	-3.11
	Rv3232c	polyphosphate kinase(ppk2)	-1.07	-1.44
	Rv3155	NADH-quinone oxidoreductase subunit K(nuoK)	-1.10	-2.25
	Rv3465	dTDP-4-dehydrorhamnose 3,5-epimerase(rmlC)	-1.14	-1.74
	Rv2007c	ferredoxin(fdxA)	-1.14	-2.12
	Rv1611	indole-3-glycerol phosphate synthase(trpC)	-1.15	-2.28
	Rv2074	pyridoxamine 5'-phosphate oxidase(Rv2074)	-1.16	-1.35
	Rv3727	oxidoreductase(Rv3727)	-1.18	-2.77
	Rv0691A	mycofactocin precursor(Rv0691A)	-1.20	-2.39
	Rv0948c	chorismate mutase(Rv0948c)	-1.20	-1.23
	Rv1882c	short-chain type dehydrogenase/reductase(Rv1882c)	-1.20	-1.86
	Rv0500	pyrroline-5-carboxylate reductase(proC)	-1.21	-1.62
	Rv1612	tryptophan synthase subunit beta(trpB)	-1.24	-2.97
	Rv2457c	ATP-dependent CLP protease ATP-binding subunit ClpX(clpX)	-1.28	-2.54
	Rv2951c	phthiodiolone/phenolphthiodiolone dimycocerosates ketoreductase(Rv2951c)	-1.29	-2.05
	Rv2276	cytochrome P450 Cyp121(cyp121)	-1.37	-2.09
	Rv3377c	type B diterpene cyclase(Rv3377c)	-1.40	-2.19
	Rv2952	phthiotriol/phenolphthiotriol dimycocerosates methyltransferase(Rv2952)	-1.42	-2.02
	Rv2949c	chorismate pyruvate-lyase(Rv2949c)	-1.43	-4.66
	Rv1360	oxidoreductase(Rv1360)	-1.43	-3.01
	Rv0635	(3R)-hydroxyacyl-ACP dehydratase subunit HadA(hadA)	-1.44	-1.41
	Rv2350c	membrane-associated phospholipase B(plcB)	-1.44	-1.63
	Rv0637	(3R)-hydroxyacyl-ACP dehydratase subunit HadC(hadC)	-1.46	-1.56
	Rv2957	PGL/p-HBAD biosynthesis glycosyltransferase(Rv2957)	-1.90	-2.35

**virulence, detoxification and adaptation (n=28)**	Rv1955	toxin HigB(higB)	2.70	2.88
	Rv1938	epoxide hydrolase EphB(ephB)	2.62	2.20
	Rv0251c	heat shock protein(hsp)	2.48	3.72
	Rv1956	antitoxin HigA(higA)	2.23	2.68
	Rv1968	Mce family protein Mce3C(mce3C)	2.18	1.47
	Rv1967	Mce family protein Mce3B(mce3B)	2.16	1.54
	Rv0186A	metallothionein(mymT)	2.09	2.02
	Rv0440	molecular chaperone GroEL(groEL2)	2.00	1.72
	Rv1970	Mce family lipoprotein LprM(lprM)	1.96	1.89
	Rv3417c	chaperonin GroEL(groEL1)	1.80	1.08
	Rv1966	Mce family protein Mce3A(mce3A)	1.74	1.46
	Rv0384c	chaperone protein ClpB(clpB)	1.65	2.43
	Rv1991A	antitoxin MazE6(mazE6)	1.58	1.54
	Rv2546	ribonuclease VapC18(vapC18)	1.55	2.51
	Rv1991c	mRNA interferase MazF6(mazF6)	1.51	1.52
	Rv1971	Mce family protein Mce3F(mce3F)	1.49	1.55
	Rv0550c	antitoxin VapB3(vapB3)	1.47	1.99
	Rv0563	protease HtpX(htpX)	1.47	2.67
	Rv0350	chaperone protein DnaK(dnaK)	1.42	1.08
	Rv0064A	antitoxin VapB1(vapB1)	1.37	1.72
	Rv1969	Mce family protein Mce3D(mce3D)	1.37	1.25
	Rv3358	toxin RelK(relK)	1.31	2.15
	Rv0549c	ribonuclease VapC3(vapC3)	1.22	1.99
	Rv3181c	antitoxin VapB45(Rv3181c)	1.14	2.26
	Rv2374c	heat-inducible transcription repressor HrcA(hrcA)	1.13	1.31
	Rv3357	antitoxin RelJ(relJ)	1.16	1.82
	Rv1960c	antitoxin ParD1(parD1)	1.11	1.61
	Rv1959c	toxin ParE1(parE1)	1.06	1.79
**regulatory proteins (n=18)**	Rv3160c	TetR family transcriptional regulator(Rv3160c)	1.89	1.88
	Rv1957	SecB-like chaperone(Rv1957)	1.88	2.29

	Rv2642	ArsR family transcriptional regulator(Rv2642)	1.82	2.05
	Rv0967	copper-sensing transcriptional repressor CsoR(csoR)	1.77	1.63
	Rv2745c	transcriptional regulator ClgR(clgR)	1.75	2.09
	Rv1985c	HTH-type transcriptional regulator(Rv1985c)	1.66	2.29
	Rv3080c	serine/threonine-protein kinase PknK(pknK)	1.65	1.59
	Rv3066	DeoR family transcriptional regulator(Rv3066)	1.60	1.48
	Rv0275c	transcriptional regulator(Rv0275c)	1.49	2.41
	Rv1460	transcriptional regulator(Rv1460)	1.46	1.52
	Rv2324	AsnC family transcriptional regulator(Rv2324)	1.39	1.50
	Rv0324	transcriptional regulator(Rv0324)	1.38	1.60
	Rv2250c	HTH-type transcriptional regulator(Rv2250c)	1.25	1.41
	Rv1963c	transcriptional repressor Mce3R(mce3R)	1.24	1.50
	Rv0767c	HTH-type transcriptional regulator(Rv0767c)	1.16	1.82
	Rv1994c	HTH-type transcriptional regulator CmtR(cmtR)	1.12	1.62
	Rv1028c	sensor protein KdpD(kdpD)	1.07	1.06
	Rv0165c	transcriptional regulator Mce1R(mce1R)	-1.41	-1.90
**information pathways (n=37)**	Rv0670	endonuclease IV(end)	1.67	1.95
	Rv2710	RNA polymerase sigma factor SigB(sigB)	1.67	1.70
	Rv3834c	serine--tRNA ligase(serS)	1.62	1.52
	Rv2554c	Holliday junction resolvase(Rv2554c)	1.60	1.68
	Rv3202c	ATP-dependent DNA helicase(Rv3202c)	1.57	1.99
	Rv0233	ribonucleoside-diphosphate reductase subunit beta NrdB(nrdB)	1.45	1.62
	Rv2555c	alanine--tRNA ligase(alaS)	1.39	1.79
	Rv3204	DNA-methyltransferase(Rv3204)	1.38	1.98
	Rv3201c	ATP-dependent DNA helicase(Rv3201c)	1.33	1.67
	Rv2838c	ribosome-binding factor RbfA(rbfA)	1.31	1.25
	Rv1259	uracil DNA glycosylase(udgB)	1.28	2.40
	Rv2839c	translation initiation factor IF-2(infB)	1.28	1.37
	Rv3370c	error-prone DNA polymerase(dnaE2)	1.21	1.64
	Rv1221	ECF RNA polymerase sigma factor SigE(sigE)	1.12	1.48

	Rv2614c	threonine--tRNA ligase(thrS)	1.08	1.46
	Rv3062	DNA ligase(ligB)	1.07	1.32
	Rv1222	anti-sigma E factor RseA(rseA)	1.05	1.73
	Rv0634B	50S ribosomal protein L33(rpmG2)	-1.47	-1.11
	Rv3923c	ribonuclease P protein component(rnpA)	-1.55	-1.96
	Rv0001	chromosomal replication initiator protein DnaA(dnaA)	-1.78	-2.54
	Rv0053	30S ribosomal protein S6(rpsF)	-1.02	-1.15
	Rv0706	50S ribosomal protein L22(rplV)	-1.06	-2.19
	Rv0056	50S ribosomal protein L9(rplI)	-1.07	-1.51
	Rv0700	30S ribosomal protein S10(rpsJ)	-1.07	-1.81
	Rv0708	50S ribosomal protein L16(rplP)	-1.08	-2.06
	Rv3924c	50S ribosomal protein L34(rpmH)	-1.14	-1.75
	Rv0707	30S ribosomal protein S3(rpsC)	-1.15	-2.24
	Rv0055	30S ribosomal protein S18(rpsR1)	-1.16	-1.58
	Rv1547	DNA polymerase III subunit alpha(dnaE1)	-1.19	-1.22
	Rv0705	30S ribosomal protein S19(rpsS)	-1.20	-2.60
	Rv0718	30S ribosomal protein S8(rpsH)	-1.20	-2.66
	Rv0704	50S ribosomal protein L2(rplB)	-1.21	-2.56
	Rv2069	ECF RNA polymerase sigma factor SigC(sigC)	-1.24	-1.26
	Rv0709	50S ribosomal protein L29(rpmC)	-1.30	-2.41
	Rv0703	50S ribosomal protein L23(rplW)	-1.35	-2.44
	Rv0717	30S ribosomal protein S14(rpsN1)	-1.46	-2.87
	Rv0710	30S ribosomal protein S17(rpsQ)	-1.46	-2.37
**PE/PPE (17)**	Rv0754	PE-PGRS family protein PE_PGRS11(PE_PGRS11)	3.28	2.76
	Rv0978c	PE-PGRS family protein PE_PGRS17(PE_PGRS17)	2.42	2.22
	Rv0834c	PE-PGRS family protein PE_PGRS14(PE_PGRS14)	2.41	2.76
	Rv2615c	PE-PGRS family protein PE_PGRS45(PE_PGRS45)	2.21	1.96
	Rv1808	PPE family protein PPE32(PPE32)	1.79	2.68
	Rv1806	PE family protein PE20(PE20)	1.64	2.22
	Rv0833	PE-PGRS family protein PE_PGRS13(PE_PGRS13)	1.53	1.60

	Rv0278c	PE-PGRS family protein PE_PGRS3(Rv0278c)	1.38	1.41
	Rv1801	PPE family protein PPE29(PPE29)	1.33	2.51
	Rv1809	PPE family protein PPE33(PPE33)	1.26	2.46
	Rv2634c	PE-PGRS family protein PE_PGRS46(PE_PGRS46)	1.19	1.25
	Rv1802	PPE family protein PPE30(PPE30)	1.12	2.33
	Rv0279c	PE-PGRS family protein PE_PGRS4(PE_PGRS4)	1.11	1.09
	Rv2431c	PE family protein PE25(PE25)	-1.06	-1.34
	Rv1196	PPE family protein PPE18(PPE18)	-1.07	-2.43
	Rv1195	PE family protein PE13(PE13)	-1.28	-3.58
	Rv3135	PPE family protein PPE50(PPE50)	-1.34	-1.66
**conserved hypotheticals (n=95)**	Rv1954c	hypothetical protein(Rv1954c)	3.18	3.28
	Rv1685c	hypothetical protein(Rv1685c)	3.10	4.54
	Rv2616	hypothetical protein(Rv2616)	2.32	2.15
	Rv0976c	hypothetical protein(Rv0976c)	2.29	1.70
	Rv3202a	hypothetical protein(Rv3202a)	2.14	3.02
	Rv2641	cadmium inducible protein CadI(cadI)	2.06	2.10
	Rv3122	hypothetical protein(Rv3122)	2.02	2.33
	Rv1462	hypothetical protein(Rv1462)	1.94	1.72
	Rv0968	hypothetical protein(Rv0968)	1.93	1.77
	Rv3123	hypothetical protein(Rv3123)	1.91	2.16
	Rv2614A	hypothetical protein(Rv2614A)	1.86	1.83
	Rv2086	hypothetical protein(Rv2086)	1.84	1.79
	Rv2744c	hypothetical protein(35kd_ag)	1.80	2.23
	Rv3288c	hypothetical protein(usfY)	1.67	1.27
	Rv2558	hypothetical protein(Rv2558)	1.67	2.20
	Rv1894c	hypothetical protein(Rv1894c)	1.67	1.92
	Rv1461	hypothetical protein(Rv1461)	1.66	1.59
	Rv3205c	hypothetical protein(Rv3205c)	1.61	2.05
	Rv3836	hypothetical protein(Rv3836)	1.50	1.47
	Rv1670	hypothetical protein(Rv1670)	1.48	1.86

	Rv1466	hypothetical protein(Rv1466)	1.47	1.41
	Rv1684	hypothetical protein(Rv1684)	1.47	2.63
	Rv3627c	hypothetical protein(Rv3627c)	1.45	1.53
	Rv2743c	hypothetical protein(Rv2743c)	1.43	1.79
	Rv1225c	hypothetical protein(Rv1225c)	1.43	2.26
	Rv0628c	hypothetical protein(Rv0628c)	1.42	1.29
	Rv1958c	hypothetical protein(Rv1958c)	1.40	1.84
	Rv0142	hypothetical protein(Rv0142)	1.39	1.49
	Rv0250c	hypothetical protein(Rv0250c)	1.38	2.90
	Rv0311	hypothetical protein(Rv0311)	1.36	1.82
	Rv2559c	hypothetical protein(Rv2559c)	1.34	1.84
	Rv2557	hypothetical protein(Rv2557)	1.33	1.16
	Rv1831	hypothetical protein(Rv1831)	1.31	1.17
	Rv0325	hypothetical protein(Rv0325)	1.29	1.47
	Rv0063a	hypothetical protein(Rv0063a)	1.27	1.68
	Rv2644c	hypothetical protein(Rv2644c)	1.26	1.70
	Rv0123	hypothetical protein(Rv0123)	1.26	1.30
	Rv3188	hypothetical protein(Rv3188)	1.24	2.31
	Rv0326	hypothetical protein(Rv0326)	1.20	1.21
	Rv2323c	hypothetical protein(Rv2323c)	1.20	1.31
	Rv3189	hypothetical protein(Rv3189)	1.20	1.82
	Rv3626c	hypothetical protein(Rv3626c)	1.18	1.01
	Rv1084	hypothetical protein(Rv1084)	1.17	1.15
	Rv0376c	hypothetical protein(Rv0376c)	1.16	2.36
	Rv1706A	hypothetical protein(Rv1706A)	1.16	1.73
	Rv0898c	hypothetical protein(Rv0898c)	1.13	1.58
	Rv2742c	hypothetical protein(Rv2742c)	1.13	1.54
	Rv3180c	ribonuclease VapC45(Rv3180c)	1.09	1.78
	Rv1429	hypothetical protein(Rv1429)	1.06	1.39
	Rv2011c	hypothetical protein(Rv2011c)	1.07	1.77

	Rv3222c	hypothetical protein(Rv3222c)	1.05	1.72
	Rv0372c	hypothetical protein(Rv0372c)	1.05	2.09
	Rv0368c	hypothetical protein(Rv0368c)	1.05	2.53
	Rv1716	hypothetical protein(Rv1716)	1.02	1.45
	Rv3376	phosphatase(Rv3376)	-1.01	-1.95
	Rv0831c	hypothetical protein(Rv0831c)	-1.01	-2.10
	Rv2927c	hypothetical protein(Rv2927c)	-1.03	-1.01
	Rv3210c	hypothetical protein(Rv3210c)	-1.03	-1.54
	Rv0108c	hypothetical protein(Rv0108c)	-1.05	-1.35
	Rv0138	hypothetical protein(Rv0138)	-1.07	-1.01
	Rv0177	Mce associated protein(Rv0177)	-1.07	-2.11
	Rv0433	carboxylate-amine ligase(Rv0433)	-1.08	-1.57
	Rv1271c	hypothetical protein(Rv1271c)	-1.09	-2.74
	Rv3491	hypothetical protein(Rv3491)	-1.09	-1.34
	Rv1513	hypothetical protein(Rv1513)	-1.10	-1.32
	Rv1433	hypothetical protein(Rv1433)	-1.11	-2.18
	Rv2955c	hypothetical protein(Rv2955c)	-1.14	-1.10
	Rv3811	hypothetical protein(Rv3811)	-1.15	-1.40
	Rv2387	hypothetical protein(Rv2387)	-1.20	-2.09
	Rv3920c	hypothetical protein(Rv3920c)	-1.20	-2.76
	Rv2275	cyclo(L-tyrosyl-L-tyrosyl) synthase(Rv2275)	-1.21	-2.15
	Rv0057	hypothetical protein(Rv0057)	-1.23	-1.83
	Rv1904	hypothetical protein(Rv1904)	-1.26	-1.91
	Rv3492c	Mce associated protein(Rv3492c)	-1.26	-1.41
	Rv0634A	hypothetical protein(Rv0634A)	-1.28	-1.21
	Rv1507A	hypothetical protein(Rv1507A)	-1.30	-1.11
	Rv2778c	hypothetical protein(Rv2778c)	-1.30	-1.64
	Rv2067c	hypothetical protein(Rv2067c)	-1.31	-1.77
	Rv3005c	hypothetical protein(Rv3005c)	-1.33	-2.58
	Rv2954c	hypothetical protein(Rv2954c)	-1.36	-2.28

	Rv3612c	hypothetical protein(Rv3612c)	-1.36	-1.69
	Rv0164	hypothetical protein(TB18.5)	-1.39	-2.12
	Rv3613c	hypothetical protein(Rv3613c)	-1.39	-1.95
	Rv1870c	hypothetical protein(Rv1870c)	-1.41	-2.84
	Rv1506c	hypothetical protein(Rv1506c)	-1.41	-1.58
	Rv1505c	hypothetical protein(Rv1505c)	-1.42	-1.71
	Rv2336	hypothetical protein(Rv2336)	-1.49	-1.60
	Rv2956	hypothetical protein(Rv2956)	-1.55	-1.56
	Rv3209	hypothetical protein(Rv3209)	-1.61	-1.84
	Rv0430	hypothetical protein(Rv0430)	-1.64	-1.48
	Rv1697	hypothetical protein(Rv1697)	-1.65	-3.12
	Rv2817c	CRISPR-associated endonuclease Cas1(Rv2817c)	-1.66	-2.16
	Rv1883c	hypothetical protein(Rv1883c)	-1.70	-2.70
	Rv1507c	hypothetical protein(Rv1507c)	-1.78	-1.74
	Rv0909	antitoxin(Rv0909)	-1.23	-1.48
**insertion seqs and phages (n=3)**	Rv1575	phage protein(Rv1575)	2.06	2.27
	Rv1578c	phage protein(Rv1578c)	1.74	2.14
	Rv1574	phage protein(Rv1574)	1.57	2.40

Supplementary Table 4 (II). Genes indicated to be significantly differentially expressed (absolute Log2Fold Chnage >1, adjusted p-value < 0.05) in bedaquiline (BDQ) resistant *Mycobacterium tuberculosis* (Mtb) clinical isolates under BDQ stress (3.75 µg/ml, t=72 h).

**Functional Category**	**Locus Tag**	**Genes**	**BDQ-R clinical isolates (L2Fc)**
**cell wall and cell processes (n=89)**	Rv3743c	cation transporter ATPase J(ctpJ)	1.87
	Rv1965	integral membrane protein(yrbE3B)	1.75
	Rv2620c	transmembrane protein(Rv2620c)	1.65
	Rv0849	MFS-type transporter(Rv0849)	1.61
	Rv2053c	transmembrane protein FxsA(fxsA)	1.60
	Rv2158c	UDP-N-acetylmuramoylalanyl-D-glutamate--2,6-diaminopimelate ligase(murE)	1.57
	Rv0970	integral membrane protein(Rv0970)	1.52

Rv1517	Conserved hypothetical transmembrane protein	1.30
Rv0475	heparin binding hemagglutinin HbhA(hbhA)	1.24
Rv2051c	polyprenol-monophosphomannose synthase(ppm1)	1.21
Rv1004c	membrane protein(Rv1004c)	1.21
Rv1964	integral membrane protein(yrbE3A)	1.19
Rv0290	ESX-3 secretion system protein EccD(eccD3)	1.18
Rv3270	manganese/zinc-exporting P-type ATPase(ctpC)	1.11
Rv2729c	integral membrane protein(Rv2729c)	1.07
Rv2157c	UDP-N-acetylmuramoyl-tripeptide--D-alanyl-D-alanine ligase(murF)	1.06
Rv2553c	membrane protein(Rv2553c)	1.03
Rv3728	Probable conserved two-domain membrane protein	0.71
Rv0411c	glutamine-binding lipoprotein GlnH(glnH)	1.11
Rv0841	transmembrane protein(Rv0841)	1.09
Rv0288	ESAT-6-like protein EsxH(esxH)	1.04
Rv0289	ESX-3 secretion-associated protein EspG3(espG3)	1.03
Rv3795	arabinosyltransferase B(embB)	-1.03
Rv2329c	nitrate/nitrite transporter(narK1)	-1.04
Rv2965c	phosphopantetheine adenylyltransferase(kdtB)	-1.05
Rv3789	GtrA family protein(Rv3789)	-1.05
Rv3278c	transmembrane protein(Rv3278c)	-1.05
Rv0128	transmembrane protein(Rv0128)	-1.06
Rv0461	transmembrane protein(Rv0461)	-1.06
Rv3620c	ESAT-6 like protein EsxW(esxW)	-1.09
Rv3312A	pilin(Rv3312A)	-1.16
Rv0236A	hypothetical protein(Rv0236A)	-1.20
Rv0114	D-glycero-alpha-D-manno-heptose-1,7-bisphosphate 7-phosphatase(gmhB)	-1.25
Rv1371	membrane protein(Rv1371)	-1.27
Rv3823c	integral membrane transport protein MmpL8(mmpL8)	-1.28

	Rv0113	phosphoheptose isomerase(gmhA)	-1.31
	Rv1183	transmembrane transport protein MmpL10(mmpL10)	-1.33
	Rv1690	lipoprotein LprJ(lprJ)	-1.39
	Rv0112	GDP-mannose 4,6-dehydratase(gca)	-1.65
	Rv1987	chitinase(Rv1987)	-1.81
**intermediary metabolism and respiration (n=49)**	Rv0467	isocitrate lyase(icl1)	2.22
	Rv1939	oxidoreductase(Rv1939)	2.03
	Rv2503c	succinyl-CoA:3-ketoacid-CoA transferase subunit B(scoB)	1.92
	Rv2502c	acetyl-/propionyl-CoA carboxylase subunit beta(accD1)	1.82
	Rv3175	amidase(Rv3175)	1.72
	Rv3406	dioxygenase(Rv3406)	1.72
	Rv1940	riboflavin biosynthesis protein RibA(ribA1)	1.66
	Rv2504c	succinyl-CoA:3-ketoacid-CoA transferase subunit A(scoA)	1.65
	Rv2161c	hypothetical protein(Rv2161c)	1.64
	Rv0765c	oxidoreductase(Rv0765c)	1.46
	Rv0764c	lanosterol 14-alpha demethylase(cyp51)	1.37
	Rv0291	membrane-anchored mycosin MycP(mycP3)	1.34
	Rv0620	galactokinase(galK)	1.31
	Rv2064	precorrin-3B synthase(cobG)	1.25
	Rv2677c	protoporphyrinogen oxidase(hemY)	1.24
	Rv0763c	ferredoxin(Rv0763c)	1.20
	Rv1605	imidazole glycerol phosphate synthase subunit HisF(hisF)	1.19
	Rv2753c	4-hydroxy-tetrahydrodipicolinate synthase(dapA)	1.19
	Rv2727c	tRNA delta(2)-isopentenylpyrophosphate transferase(miaA)	1.18
	Rv2540c	chorismate synthase(aroF)	1.16
	Rv1656	ornithine carbamoyltransferase(argF)	1.15
	Rv2552c	shikimate 5-dehydrogenase(aroE)	1.15
	Rv0897c	oxidoreductase(Rv0897c)	1.10

	Rv1327c	alpha-1,4-glucan:maltose-1-phosphate maltosyltransferase(glgE)	1.09
	Rv2539c	shikimate kinase(aroK)	1.08
	Rv1130	2-methylcitrate dehydratase(prpD)	1.06
	Rv1604	inositol-monophosphatase ImpA(impA)	1.05
	Rv3086	alcohol dehydrogenase D(adhD)	1.24
	Rv1621c	cytochrome biosyntheisis ABC transporter ATP-binding protein/permease CydD(cydD)	1.22
	Rv0331	dehydrogenase/reductase(Rv0331)	1.15
	Rv0766c	cytochrome P450 Cyp123(cyp123)	1.07
	Rv2851c	GCN5-like N-acetyltransferase(Rv2851c)	1.07
	Rv1285	sulfate adenylyltransferase subunit 2(cysD)	1.04
	Rv1854c	NADH dehydrogenase(ndh)	1.03
	Rv1622c	cytochrome D ubiquinol oxidase subunit II CydB(cydB)	1.02
	Rv3537	3-oxosteroid 1-dehydrogenase(kstD)	1.02
	Rv3538	dehydrogenase(Rv3538)	1.01
	Rv1595	L-aspartate oxidase(nadB)	1.00
	Rv2277c	glycerolphosphodiesterase(Rv2277c)	-1.03
	Rv0636	(3R)-hydroxyacyl-ACP dehydratase subunit HadB(hadB)	-1.03
	Rv2338c	molybdopterin biosynthesis protein MoeW(moeW)	-1.07
	Rv3322c	methyltransferase(Rv3322c)	-1.08
	Rv3316	succinate dehydrogenase cytochrome B-556 subunit(sdhC)	-1.12
	Rv3323c	MoaD-MoaE fusion protein MoaX(moaX)	-1.16
	Rv3464	dTDP-glucose 4,6-dehydratase(rmlB)	-1.18
	Rv3378c	diterpene synthase(Rv3378c)	-1.19
	Rv2959c	rhamnosyl O-methyltransferase(Rv2959c)	-1.22
	Rv0111	acyltransferase(Rv0111)	-1.30
	Rv3324c	cyclic pyranopterin monophosphate synthase accessory protein(moaC3)	-1.43
**lipid metabolism (n=23)**	Rv0973c	acetyl/propionyl-CoA carboxylase subuit alpha(accA2)	1.79
	Rv0974c	acetyl-/propionyl-CoA carboxylase subunit beta(accD2)	1.77

	Rv1935c	enoyl-CoA hydratase EchA13(echA13)	1.61
	Rv0972c	acyl-CoA dehydrogenase fadE12(fadE12)	1.42
	Rv0971c	enoyl-CoA hydratase EchA7(echA7)	1.32
	Rv1934c	acyl-CoA dehydrogenase FadE17(fadE17)	1.28
	Rv1492	methylmalonyl-CoA mutase small subunit(mutA)	1.15
	Rv2934	phthiocerol synthesis polyketide synthase type I PpsD(ppsD)	0.92
	Rv3409c	cholesterol oxidase(choD)	1.08
	Rv3061c	acyl-CoA dehydrogenase FadE22(fadE22)	1.05
	Rv3543c	acyl-CoA dehydrogenase FadE29(fadE29)	1.05
	Rv0672	acyl-CoA dehydrogenase FadE8(fadE8)	1.04
	Rv2483c	bifunctional L-3-phosphoserine phosphatase/1-acyl-sn-glycerol-3-phosphate acyltransferase(plsC)	1.02
	Rv0468	3-hydroxybutyryl-CoA dehydrogenase(fadB2)	1.01
	Rv0673	enoyl-CoA hydratase EchA4(echA4)	1.01
	Rv1493	methylmalonyl-CoA mutase large subunit(mutB)	1.01
	Rv3791	decaprenylphosphoryl-D-2-keto erythropentose reductase(dprE2)	-1.07
	Rv3790	decaprenylphosphoryl-beta-D-ribose oxidase(dprE1)	-1.08
	Rv2289	CDP-diacylglycerol pyrophosphatase(cdh)	-1.08
	Rv0644c	cyclopropane mycolic acid synthase CmaA(mmaA2)	-1.09
	Rv1182	acyltransferase papA3(papA3)	-1.17
	Rv3826	long-chain-fatty-acid--CoA ligase FadD23(fadD23)	-1.33
	Rv3824c	acyltransferase(papA1)	-1.51
**virulence, detoxification, and adaptation (n=10)**	Rv3176c	epoxide hydrolase MesT(mesT)	1.99
	Rv1908c	catalase-peroxidase(katG)	1.65
	Rv3418c	chaperonin GroES(groES)	1.50
	Rv0351	stress response protein GrpE(grpE)	1.50
	Rv0352	chaperone protein DnaJ(dnaJ1)	1.43
	Rv2373c	chaperone protein DnaJ(dnaJ2)	1.30

	Rv3407	antitoxin VapB47(vapB47)	1.12
	Rv0353	heat shock protein transcriptional repressor HspR(hspR)	1.04
	Rv2428	alkyl hydroperoxide reductase subunit AhpC(ahpC)	-1.18
	Rv3321c	antitoxin VapB44(vapB44)	-1.56
**regulatory proteins (n=10)**	Rv2621c	transcriptional regulator(Rv2621c)	1.57
	Rv2912c	TetR family HTH-type transcriptional regulator(Rv2912c)	1.29
	Rv1909c	ferric uptake regulation protein FurA(furA)	1.11
	Rv1657	arginine repressor(argR)	1.10
	Rv1129c	transcriptional regulator(Rv1129c)	1.04
	Rv0410c	serine/threonine-protein kinase PknG(pknG)	1.02
	Rv3260c	transcriptional regulator WhiB2(whiB2)	-1.05
	Rv3058c	TetR family transcriptional regulator(Rv3058c)	-1.08
	Rv0891c	hypothetical protein(Rv0891c)	-1.23
	Rv0144	transcriptional regulator(Rv0144)	-1.33
**information pathways (n=5)**	Rv3287c	anti-sigma factor RsbW(rsbW)	1.52
	Rv3286c	RNA polymerase sigma factor SigF(sigF)	1.10
	Rv3420c	ribosomal-protein-alanine acetyltransferase RimI(rimI)	1.07
	Rv1406	methionyl-tRNA formyltransferase(fmt)	1.07
	Rv0429c	polypeptide deformylase(def)	-1.01
**stables rnas (n=1)**	Rvnr01	16S ribosomal RNA(rrs)	1.79
**PE/PPE (n=1)**	Rv3533c	PPE family protein PPE62(PPE62)	1.58
**conserved hypotheticals (n=1)**	Rv2052c	hypothetical protein(Rv2052c)	1.90

Supplementary Table 5: Biological processes significantly enriched (FDR < 0.05) in study isolates (BDQ-resistant clincial isolates; n=6 and H37Rv) after 72 hours of bedaquiline (BDQ; 3.75 µg/ml) exposure.

(I) Significantly enriched biological processes identified in clinical isolates exposed to bedaquiline (BDQ; 3.75 µg/ml, t= 72 h).

GO biological process complete	Fold Enrichment	p-value	FDR
protein refolding (GO:0042026)	11.22	4.82E-07	1.63E-04
response to cadmium ion (GO:0046686)	11.22	7.02E-04	3.86E-02
branched-chain amino acid catabolic process (GO:0009083)	9.35	3.05E-05	8.85E-03
pyruvate family amino acid catabolic process (GO:0009080)	7.48	8.05E-04	4.31E-02
response to copper ion (GO:0046688)	7.48	3.20E-05	8.14E-03
response to heat (GO:0009408)	7.01	1.38E-07	6.99E-05
response to temperature stimulus (GO:0009266)	6.17	1.95E-07	7.93E-05
L-arginine biosynthetic process (GO:0006526)	5.61	2.79E-04	1.89E-02
arginine metabolic process (GO:0006525)	5.18	4.80E-04	2.79E-02
protein folding (GO:0006457)	4.99	7.24E-05	9.82E-03
response to metal ion (GO:0010038)	4.21	1.25E-04	1.10E-02
proteinogenic amino acid catabolic process (GO:0170040)	4.13	8.24E-04	4.30E-02
amino acid catabolic process (GO:0009063)	3.88	2.54E-04	1.78E-02
fatty acid beta-oxidation using acyl-CoA dehydrogenase (GO:0033539)	3.59	1.02E-03	4.85E-02
cholesterol catabolic process (GO:0006707)	3.34	2.46E-04	1.85E-02
sterol catabolic process (GO:0016127)	3.34	2.46E-04	1.79E-02
sterol metabolic process (GO:0016125)	3.32	7.23E-05	1.05E-02
alcohol catabolic process (GO:0046164)	3.28	1.52E-04	1.29E-02
fatty acid beta-oxidation (GO:0006635)	3.27	4.47E-05	1.01E-02
steroid catabolic process (GO:0006706)	3.25	3.19E-04	2.09E-02
cholesterol metabolic process (GO:0008203)	3.21	1.96E-04	1.59E-02
secondary alcohol metabolic process (GO:1902652)	3.17	1.20E-04	1.11E-02
carboxylic acid catabolic process (GO:0046395)	3.15	1.00E-07	6.78E-05
lipid oxidation (GO:0034440)	3.14	7.36E-05	9.36E-03
fatty acid oxidation (GO:0019395)	3.14	7.36E-05	8.81E-03
lipid modification (GO:0030258)	3.14	7.36E-05	8.32E-03
organic acid catabolic process (GO:0016054)	3.14	6.11E-08	1.24E-04
fatty acid catabolic process (GO:0009062)	3.06	5.70E-05	9.67E-03
steroid metabolic process (GO:0008202)	3.02	1.18E-04	1.20E-02

monocarboxylic acid catabolic process (GO:0072329)	2.94	5.45E-05	1.11E-02
small molecule catabolic process (GO:0044282)	2.65	9.53E-08	9.69E-05
response to abiotic stimulus (GO:0009628)	2.38	1.19E-04	1.15E-02
lipid catabolic process (GO:0016042)	2.21	2.07E-04	1.62E-02
amino acid metabolic process (GO:0006520)	1.91	3.71E-04	2.29E-02
response to chemical (GO:0042221)	1.9	1.15E-04	1.23E-02
response to stress (GO:0006950)	1.88	5.64E-05	1.04E-02
catabolic process (GO:0009056)	1.7	4.34E-04	2.60E-02
carboxylic acid metabolic process (GO:0019752)	1.58	3.48E-04	2.21E-02
response to stimulus (GO:0050896)	1.54	6.37E-05	9.97E-03
organic acid metabolic process (GO:0006082)	1.53	9.36E-04	4.53E-02
phosphate-containing compound metabolic process (GO:0006796)	0.42	8.48E-04	4.21E-02
phosphorus metabolic process (GO:0006793)	0.41	8.47E-04	4.31E-02
organophosphate metabolic process (GO:0019637)	0.39	6.48E-04	3.66E-02

(II) Significantly enriched biological processes identified in H37Rv exposed to bedaquiline (BDQ; 3.75 µg/ml, t= 72 h).

**GO biological process complete**	**Fold Enrichment**	**p-value**	**FDR**
response to copper ion (GO:0046688)	5.5	1.87E-04	3.17E-02
response to heat (GO:0009408)	4.13	2.32E-04	3.38E-02
regulation of DNA-templated transcription (GO:0006355)	2.08	1.11E-08	2.26E-05
regulation of RNA biosynthetic process (GO:2001141)	2.08	1.11E-08	1.13E-05
regulation of RNA metabolic process (GO:0051252)	2.07	1.27E-08	8.59E-06
regulation of nucleobase-containing compound metabolic process (GO:0019219)	2	6.85E-08	3.48E-05
regulation of macromolecule biosynthetic process (GO:0010556)	1.94	7.01E-08	2.85E-05
regulation of gene expression (GO:0010468)	1.94	7.01E-08	2.38E-05
regulation of biosynthetic process (GO:0009889)	1.9	1.61E-07	4.67E-05
regulation of primary metabolic process (GO:0080090)	1.89	2.79E-07	6.32E-05
regulation of macromolecule metabolic process (GO:0060255)	1.88	1.93E-07	4.90E-05
regulation of metabolic process (GO:0019222)	1.8	1.04E-06	2.11E-04
regulation of cellular process (GO:0050794)	1.59	7.57E-05	1.40E-02
carbohydrate derivative biosynthetic process (GO:1901137)	0.33	2.17E-04	3.40E-02

Supplementary Table 6. Genes screened in previous transcriptomic studies exploring antimycobacterial drug exposure that had similar differential upregulation as the studied bedaquiline (BDQ) resistant isolates (n=6) after 72 hours of BDQ exposure (3.75 µg/ml).

Genes	Gene Onotology (GO) terms for biological process	Antibiotics	Exposure time	Strain	Ref
fabD2	long-chain fatty acid biosynthetic process	standard DS therapy (2-mo intensive phase of RIF, INH, PZA, & EMB, followed by a 4-mo continuation phase of RIF and INH.	after 72 hours of DS chemotherapy	DS clinical isolates collected from TB patients	[35]
sucC	succinyl-CoA metabolic process, growth, tricarboxylic acid cycle	DLM	6 & 24 hours of DLM exposure	H37Rv	[34]
mqo	tricarboxylic acid cycle				
Rv2455c	response to oxidative stress , tricarboxylic acid cycle				
		standard DS TB drugs	between 2 and 14 days post DS TB trt initiation	clinical isolates from DS TB patients	[45]
Rv2025c	monoatomic ion transmembrane transport including zinc and cadmium ion transmembrane transport	DLM, HRZE, standard DS TB therapy	6 & 24 hours of DLM exposure, between 2 and 14 days post DS TB trt initiation, after 4 & 8 days of *in vitro* exposure of Mtb to HRZE	H37Rv, Erdmann, clinical isolates from DS TB patients	[34,41 ,45]
rv2651c	Proteolysis				
Rv3087	response to acidic pH,nitric oxide, suppression by symbiont of host immune response, glycerolipid biosynthetic process, lipid metabolic process	standard DS therapy	after 72 hours of DS chemotherapy	DS clinical isolates collected from TB ptnts	[35]
pks13	secondary metabolite biosynthetic process , lipid metabolic process , DIM/DIP cell wall layer assembly	CAP	4 hours post CAP exposure.	H37Rv	[48]
		INH	6 hours of INH exposure.		[46]
rv3066	regulation of gene expression , regulation of DNA-templated transcription	standard DS TB chemotherapy	after 72 hours of DS chemotherapy	DS clinical isolates collected from TB patients	[35]
			between 2 and 14 days post DS TB trt initiation		[45]
		DLM	6 & 24 hours of DLM exposure	H37Rv	[34]
bkdC					[35,

Rv2688c	xenobiotic detoxification by transmembrane export across the plasma membrane, response to antibiotic	standard DS TB chemotherapy	after 72 hours of DS chemotherapy, between 2 and 14 days post DS TB trt initiation	DS clinical isolates collected from TB patients	45]
argF	citrulline and arginine biosynthetic process via ornithine , urea cycle , and growth				
icl1	response to: acidic pH ,hypoxia, host immune response. Involved in carboxylic acid metabolic process, isocitrate metabolic process , tricarboxylic acid cycle, glyoxylate cycle.		after 72 hours of DS chemotherapy		[35]
hisF	involved in histidine biosynthesis				
groEL2	chaperone cofactor-dependent protein refolding , positive regulation of transcription regulatory region DNA binding , 'de novo' protein folding , adhesion of symbiont to host , protein refolding , response to heat and hypoxia	LZD	post 4 hours exposure	H37Rv	[42]
		EMB	24 and 48 hours		[47]
		CAP	4 hours post CAP exposure.		[43]
		INH	6 hours of INH exposure.		[46]
clpB	cellular response to heat , protein refolding , growth , response to heat	LZD	post 4 hours exposure		[42]
		standard DS TB chemotherapy	between 2 and 14 days post DS TB trt initiation	DS clinical isolates collected from TB patients	[45]
Rv2466c	protein homotetramerization , cell redox homeostasis	CAP	4 hours post CAP exposure.	H37Rv	[43]
		INH	6 hours of INH exposure.	H37Rv	[46]
Rv0188	-	standard DS TB chemotherapy	between 2 and 14 days post DS TB trt initiation	DS clinical isolates collected from TB patients	[45]
		DLM	6 & 24 hours of DLM exposure	H37Rv	[35]
		INH	6 hours of INH exposure.		[46]
Rv0837c					
Rv2044c					
glnH	Growth	DLM	6 & 24 hours of DLM exposure		[35]
		INH	6 hours of INH exposure		[23]
Rv0064	-	standard DS TB chemotherapy, DLM	between 2 and 14 days post DS TB trt initiation, 6 & 24 hours of DLM exposure	DS clinical isolates collected from	[34,35]
Rv0311	Growth				
Rv0324	response to antibiotic , regulation of DNA-templated transcription				
vapB1	regulation of DNA-templated transcription				

vapC3	nucleic acid phosphodiester bond hydrolysis , response to host , positive regulation of growth			TB patients, H37Rv	
vapB3	response to host , negative regulation of growth				
Rv0123	-				
Rv0188	-				
Rv0968	-				
Rv1227c	-				
ctpV	detoxification of copper ion, monoatomic cation transmembrane transport, copper ion homeostasis.				
Rv1227c	-				
cyp130	cholesterol catabolic process				
hisD	growth ,histidine biosynthetic process				
argC	growth arginine biosynthetic process				
Rv1687c	response to organic substance	standard DS TB chemotherapy, triclosan (x1 &x5 MIC)	between 2 and 14 days post DS TB trt initiation, 2 or 6 h incubation	DS clinical isolates collected from TB patients, H37Rv	[34, 35,46]
Rv1985c	negative regulation of DNA replication , both positive and negative regulation of DNA-templated transcription				
PPE29	response to host immune response				
furA	regulation of secondary metabolite biosynthetic process , negative regulation of DNA-templated transcription , response to: iron ion, oxidative stress.				
cmtR	response to cadmium and lead ions, regulation of gene expression through DNA-templated transcription				
rv2557	cellular response to starvation				
rv2558					
rv2570	-				
cadI	response to cadmium ion				
udgB	DNA damage response , DNA repair , base-excision repair				
accA2	Growth	DLM	6 & 24 hours of DLM exposure	H37Rv	[34]
Rv0628c	Growth				
ldtA	cell wall organization; peptidoglycan-protein cross-linking, regulation of cell shape				
mymT	response to metal ions including: copper, cadmium, nickel and zinc ion. Involved in sequestering of copper ions.				
Rv0142	DNA dealkylation involved in DNA repair , base-excision repair, AP site formation , base-excision repair				

Rv0275c	regulation of gene expression				
Rv0307c	-				
Rv0325	biological process involved in interaction with host		6 & 24 hours of DLM exposure	Erdmann	[34]
Rv0326	Methylation	standard DS TB chemotherapy	between 2 and 14 days post DS TB trt initiation	DS clinical isolates collected from TB patients	[42]
Rv0376c	-	LZD	4 hours post LZD exposure.	H37Rv	[39]
clpB	cellular response to heat , protein refolding , growth	DLM	6 & 24 hours of DLM exposure		[34, 45]
		standard DS TB chemotherapy	between 2 and 14 days post DS TB trt initiation	DS clinical isolates collected from TB patients	
htmA	methylation , cellular response to iron ion starvation , response to: salicylic acid and hypoxia	INH	6 hours of INH exposure.	H37Rv	[46]
end	nucleic acid phosphodiester bond hydrolysis , DNA damage response , base-excision repair , DNA repair	DLM	6 & 24 hours of DLM exposure		[34]
PE_PGRS11	glycolytic process, response to hypoxia				
csoR	negative regulation of DNA-templated transcription , response to copper ion , response to silver ion , regulation of DNA-templated transcription				
Rv0898c	-				
Rv0836c	-				
Rv0898c	-				
PPE14	response to host immune response				
csoR	negative regulation of DNA-templated transcription , response to copper ion , response to silver ion , regulation of DNA-templated transcription				
Rv0968	-	INH	30 mins of drug exposure		[44]
Rv0976c	-	standard DS TB chemotherapy	between 2 and 14 days post DS TB trt initiation	DS clinical isolates collected from TB patients	[45]

kdpD	detection of chemical stimulus , peptidyl-histidine phosphorylation , phosphorylation , signal transduction, phosphorelay signal transduction system	DLM	6 & 24 hrs of DLM	H37Rv	[34]
sigE	regulation of DNA-templated transcription initiation, regulation of chaperone-mediated protein complex assembly , response to host immune response , biological process involved in interaction with host , response to: abiotic stimulus, hydrogen peroxide, xenobiotic stimulus, heat				
rseA	positive regulation of DNA-templated transcription , regulation of DNA-templated transcription				
Rv1227c	-				
cyp130	cholesterol catabolic process				
udgB	DNA damage response , DNA repair , base-excision repair				
Rv1462	response to host immune response , growth , iron-sulfur cluster assembly				
Rv1985c	negative regulation of DNA replication , both positive and negative regulation of DNA-templated transcription				
csd	growth , cysteine metabolic process				
Rv1831	-				
higB	detoxification , nucleic acid phosphodiester bond hydrolysis , positive regulation of growth , negative regulation of growth , response to hypoxia	standard DS TB chemotherapy	between 2 and 14 days post DS TB trt initiation	DS clinical isolates collected from TB patients	[45]
		INH, ETH	4 hours post drug exposure	H37Rv	[48]
ctpG	zinc, and copper ion transport, transmembrane transport	DLM	6 & 24 hrs of DLM		[34]
Rv1993c	-				
rv2011c	negative regulation of DNA-templated transcription				
rv2025c	monoatomic ion transmembrane transport including zinc and cadmium ion transmembrane transport,				
rv2035	-				
rv2324	response to amino acid				
hrcA	negative regulation of DNA-templated transcription , regulation of DNA-templated transcription				
mbtB	organic cyclic compound biosynthetic process including fatty acid and amide biosynthetic processes, amino acid activation for				

	nonribosomal peptide biosynthetic process, carboxylic acid metabolic process, lipid biosynthetic process.				
rv2455c	response to oxidative stress , tricarboxylic acid cycle				
bkdA	acetyl-CoA biosynthetic process from pyruvate				
rv2515c	-				
rv2660c	-	DLM, HRZE, standard DS therapy	6 & 24 hours of DLM exposure, between 2 and 14 days post DS TB trt initiation	H37Rv, Erdmann DS clinical isolates collected from TB patients	[34, 41, 45]
rv1227c		HRZE, standard DS therapy	between 2 and 14 days post DS TB trt initiation		[34,41 ,45]
ligB	lagging strand elongation , DNA biosynthetic process, cell division , DNA ligation involved in DNA repair , cell cycle, DNA damage response				
PE_PGRS11	glycolytic process, response to hypoxia				
ctpV	intracellular copper ion homeostasis				
PPE29	response to host immune response		6 & 24 hours of DLM exposure, after 4 & 8 days of *in vitro* exposure of Mtb to HRZE	Erdmann, DS clinical isolates collected from TB patients	
rv1985c	regulation of DNA-templated transcription				
rv2688c	xenobiotic detoxification by transmembrane export across the plasma membrane, response to antibiotic				
rv2963					
rv1517, rv0790c					
accD1	L-leucine catabolic process				
rv3502c	cholesterol catabolic process				
rv0765c	steroid metabolic process				
fadA5	lipid metabolic process				
PPE62	response to host immune response				
relK	global gene silencing by mRNA cleavage & negative regulation of translational initiation				
rv1670, rv0762c					
rv2323c	arginine deiminase pathway				
ppm1	symbiont-mediated perturbation of host immune response & glycolipid biosynthetic process				
rsmE	rRNA base methylation // methylation // rRNA processing				

rv2559c	DNA synthesis involved in DNA repair				
rv1429					
rimI	mycothiol biosynthetic process				
rv2658c					
lipV	fatty acid catabolic process				
mmsB	branched-chain amino acid catabolic process		post exposure to HRZE: for 4, and 8 days in invitro & 28 and 48 days *in vivo* (mice)	H37Rv and Erdmann	[41]
mshA	glycolipid biosynthetic process				
PE_PGRS51					
rv0374c	electron transport chain				
rv0970					
tatB	protein transport by the Tat complex				
rv1225c	Dephosphorylation				
rv1226c					
fadB3	fatty acid beta-oxidation				
mce3A	biological process involved in interaction with host				
mce3B	biological process involved in interaction with host				
mce3C	biological process involved in interaction with host				
lprM	biological process involved in interaction with host				
mce3F	biological process involved in interaction with host				
mazE6	detoxification				
mazF6	symbiont-mediated perturbation of host process , rRNA & mRNA catabolic process				
rv2052c, rv2614A, rv2644c, rv2742c, rv2616, rv3836					
rv2646	DNA integration				
rv2653c	negative regulation of growth				
clpC2					
ugpE	biological process involved in interaction with host // transmembrane transport		post 28 and 48 days exposure to HRZE *in vivo* (mice)		
rv2897c	Growth				
rv3204	methylation // DNA repair				
arsC	transmembrane transport				
sigF	response to antibiotic, nitrogen starvation and oxidative stress				

Rv0077c		1x or 5x MIC of triclosan	2 or 6 hrs of exposure	H37Rv	[46]
Rv1685c	regulation of gene expression and DNA templated transcription				
Rv1686c	response to antibiotics and transmembrane transport				
Rv3160c	regulation of gene expression and DNA templated transcription				
Rv3161c	cellular metabolic processes				
Rv0250c	-	INH: 0.2 μg/ml	Log phase 3 days, NRP: 6 & 21 days		[25]

Abbreviations: DS= drug susceptible, mo= months, RIF= rifampicin, INH= isoniazid, EMB= ethambutol, PZA= pyrazinamide, TB= tuberculosis, DLM= delamanid, HRZE= isoniazid, rifampicin, pyrazinamide, ethambutol, CAP= capreomycin, LZD= linezolid, MIC= minimum inhibitory concentration, NRP= non-replicating persisters.

Supplementary Table 7. Minimum Inhibitory Concentrations (MIC) of bedaquiline (BDQ)-resistant *Mycobacterium tuberculosis* (Mtb) isolates (n=6) selected for BDQ-induced whole transcriptome study.

Study ID	SubType (e.g. MDR/ Pre-XDR/ XDR-TB)	Drug Susceptibility Testing Method Used																						
		7H10 Agar proportion method									7H10 Agar Dil.	Broth Microdilution Method (Frozen plate) (µg/ml)												MGIT 960
		RIF (1µg/ml)*	INH (0.2µg/ml)	EMB (5 µg/ml)	LVX (1 µg/ml)	ETH (5 µg/ml)	AMK (2 µg/ml)	KAN (5 µg/ml)β	PZA (100 µg/ml)	BDQ (>0.25µg/ml)	BDQ (µg/ml)	BDQ	RIF	INH	OFX	LVX	MOX	LZD	CFZ	CAP	KAN	AMK	EMB	BDQ CC (1µg/ml) R/S
S1	XDR	R	R	R	S	S	S	S	R	R	0.5	0.5	4	>4	0.5	0.5	4	1	0.5	2	8	1	8	R
S2	XDR	R	R	R	S	S	S	S	R	R	0.5	0.5	8	4	0.5	0.5	0.25	2	0.5	2	1	0.12	8	R
S5	XDR	R	R	R	S	S	S	S	R	R	0.5	0.5	8	>4	1	0.5	0.5	2	0.5	2	1	0.12	8	R
S7	XDR	R	R	S	S	S	S	S	R	R	0.5	0.5	4	0.5	0.25	0.25	1	>4	0.5	≤0.06	2	2	0.5	R
S9	XDR	R	R	R	S	S	S	S	R	R	0.5	0.5	4	>4	1	0.5	4	4	1	2	8	1	16	R
S10	XDR	R	R	S	S	S	S	S	R	R	0.5	0.5	1	>4	0.5	0.12	0.5	2	0.5	2	1	0.12	4	R
H37Rv	control	S	S	S	S	S	S	S	S	S	0.06	<0.015	0.25	0.03	0.5	0.25	0.12	1	0.015	1	1	0.5	0.5	S

Legends: Drug susceptibility testing were performed using 7H10 Agar proportion method using following drug concentrations; rifampicin (RIF): 1µg/ml, isoniazid (INH): 0.2µg/ml, ethambutol (EMB): 5µg/ml, levofloxacin (LVX): 1µg/ml, ethionamide (ETH): 5µg/ml, amikacin (AMK): 2µg/ml [1] and kanamycin (KAN): 5µg/ml [2]. Pyrazinamide (PZA) sensitivity was determined by the MGIT 960 method using 100 µg/ml [3]. Bedaquiline (BDQ) MICs for MDR (RIF- and INH-resistant) isolates were performed using: (i) 7H10 agar dilution method [4]. Isolates with MIC >0.25 µg/ml [5] were considered as resistant. Isolates showing resistance or intermediate resistance to BDQ were further confirmed using following cut-offs: (i) 7H10 agar proportion method; MIC > 0.25 µg/ml. (ii) MGIT 960; cc= 1µg/ml [6]. BDQ MICs determined using broth microdilution method (BMD) were interpreted as follows: resistance ≥0.5 µg/ml, intermediate=0.25 µg/ml and susceptible ≤0.12 µg/ml [6].

Abbreviations: pre-XDR= pre-extensively drug resistant, DS= drug susceptible, N.T= not tested, APM= agar proportion method, OFX= ofloxacin, MOX= moxifloxacin, LZD; linezolid, CFZ= clofazamine, CAP= capreomycin, AMK= amikacin.

References

1. WHO. Technical Manual for drug susceptibility using of medicines used in the treatment of tuberculosis. Geneva: World Health Organization; 2018. [Available from: https://www.who.int/tb/publications/2018/WHO_technical_drug_susceptibility_testing/en/]. Last Accessed 4 February 2021.

2. WHO. Updated interim critical concentrations for first-line and second-line DST (as of May 2012). [Avaialble from: https://www.stoptb.org/wg/gli/assets/documents/Updated%20critical%20concentration%20table_1st%20and%202nd%20line%20drugs.pdf]. Last Accessed 10 November 2021.

3. Woods GL, Brown-Elliott BA, Conville PS, Desmond EP, Hall GS, Lin G, Pfyffer GE, Ridderhof JC, Siddiqi SH, Wallace RJ, Jr. et al. In: Susceptibility Testing of Mycobacteria, Nocardiae, and Other Aerobic Actinomycetes. Edited by nd. Wayne (PA); 2011.

4. Kaniga K, Cirillo DM, Hoffner S, Ismail NA, Kaur D, Lounis N, Metchock B, Pfyffer GE, Venter A: A Multilaboratory, Multicountry Study To Determine Bedaquiline MIC Quality Control Ranges for Phenotypic Drug Susceptibility Testing. J Clin Microbiol. 2016;54(12):2956-62.

5. Kaniga K, Hasan R, Jou R, Vasiliauskiene E, Chuchottaworn C, Ismail N, et al. Bedaquiline Drug Resistance Emergence Assessment in MDR-TB (DREAM): a 5-Year Prospective In-Vitro Surveillance Study of Bedaquiline and Other Second-Line Drug-Susceptibility Testing in MDR-TB Isolates. J Clin Microbiol. 2021; doi: 10.1128/JCM.02919-20.

6. Kaniga K, Aono A, Borroni E, Cirillo DM, Desmaretz C, Hasan R, et al. Validation of Bedaquiline Phenotypic Drug Susceptibility Testing Methods and Breakpoints: a Multilaboratory, Multicountry Study. J Clin Microbiol. 2020; doi:10.1128/JCM.01677-19.

Supplementary Table 8. ***Mycobacterium tuberculosis*** **(Mtb) isolates with genomic variants in** ***rv0678*****, quality of RNA extracted and output QC indicated by RNA concentration, A280, and Q20(%)/Q30(%) of raw Fastq files obtained from Illumina HiSeq platform, respectively.**

S.No	ID	Isolate	Variants in *rv0678*	Experimental Condition (conc. BDQ (µg/ml), time (hrs))	Conc (ng/µl)	A280	Q20(%)/Q30(%)
1	7	Rv	Not applicable	0x BDQ 0 hrs	123.686	1.96	98.2/95.2
2	S1	Rv		0x BDQ 0 hrs	80.708	1.964	98.4/95.6
3	15	Rv		0X BDQ, 72 hrs	502.591	1.954	98.6/95.9
4	16	Rv		0X BDQ, 72 hrs	459.817	2,017	98.2/95.2
5	13	Rv		7.5X BDQ, 72 hrs	780.33	2.035	98.1/95.1
6	14	Rv		7.5X BDQ, 72 hrs	305.105	1.923	98.6/95.8
7	25	S1		0X BDQ, 0hrs	409.463	1.908	98.1/95.2
8	S1	S1		0X BDQ, 0hrs	356.221	2.01	98.4/95.6
9	36	S1		0X BDQ, 72 hrs	96.2	1.8	98.4/95.5
10	38	S1		0X BDQ, 72 hrs	109.462	1.851	97.5/94.4
11	S1	S1		7.5X BDQ, 72 hrs	306.964	1.92	97.8/94.8
12	S1_49	S1	Not detected	7.5X BDQ, 72 hrs	409.463	1.908	98.1/95.2
13	41	S2	Not detected	0X BDQ, 72 hrs	152.407	1.997	98/94.9
14	42	S2		0X BDQ, 72 hrs	52.08	1.864	98.6/95.9
15	48	S2		7.5X BDQ, 72 hrs	234.274	1.95	98.4/95.8
16	49	S2		7.5X BDQ, 72 hrs	118.026	1.85	98.4/95.8
17	56	S2		0X BDQ, 72 hrs	259.596	1.97	98.4/95.5
18	57	S2		0X BDQ, 72 hrs	313.895	2	98.3/95.4
19	27	S7	Not detected	0X BDQ, 0hrs	175.422	2.05	98.5/95.9
20	28	S7		0X BDQ, 0hrs	122.539	1.99	98.2/95.2
21	32	S7		0X BDQ, 72 hrs	333.12	1.9	98.4/95.6
22	33	S7		0X BDQ, 72 hrs	363.503	1.95	98.3/95.2
23	27B	S7		7.5X BDQ, 72 hrs	240.48	1.918	98.3/95
24	26B	S7		7.5X BDQ, 72 hrs	192.893	1.928	98.4/95.8
25	39	S5		0X BDQ, 0hrs	199.061	1.981	97.8/94.3
26	40	S5		0X BDQ, 0hrs	424.976	1.957	97.8/94.3
27	50	S5	L40F	0X BDQ, 72 hrs	448.146	1.98	97.9/94.6

28	55	S5		0X BDQ, 72 hrs	79.311	1.86	97.9/94.7
29	51	S5		7.5X BDQ, 72 hrs	136.855	1.91	98.6/96.2
30	54	S5		7.5X BDQ, 72 hrs	172.839	1.9	97.9/94.7
31	43	S9	R107C	0X BDQ, 0hrs	263.023	1.92	98/94.3
32	44	S9		0X BDQ, 0hrs	306.565	1.937	97.9/94.5
33	52	S9		0X BDQ, 72 hrs	79.31	1.86	97.9/94.6
34	53	S9		0X BDQ, 72 hrs	162.479	1.91	98/94.8
35	46	S9		7.5X BDQ, 72 hrs	192.613	1.95	98/94.8
36	47	S9		7.5X BDQ, 72 hrs	254.402	1.85	98/94.8
37	29	S10	G25VCRVfs	0X BDQ, 0hrs	189.508	2.032	97.8/94.6
38	30	S10		0X BDQ, 0hrs	256.92	2.08	97.9/94.6
39	34	S10		0X BDQ, 72 hrs	247.4	1.93	97.9/94.7
40	35	S10		0X BDQ, 72 hrs	173.39	1.95	97.8/94.3
41	S10, 7.5X,72	S10		7.5X BDQ, 72 hrs	221.85	1.94	98.2/95.2

Supplementary Table 9. Sensitivity analysis of differentially expressed genes (absolute Log2Fold Change >1, adjusted p-value < 0.05) for BDQ-induced genes in BDQ-resistant clinical isolates (n=6).

Locus_Tags	baseMean	L2Fc	lfcSE	stat	pvalue	padj
Rv1687c	3874.55	5.13	0.24	20.99	7.66E-98	1.01E-94
Rv1686c	2876.86	4.79	0.22	21.82	1.49E-105	5.89E-102
Rv0754	6447.60	3.47	0.16	21.22	6.71E-100	1.33E-96
Rv1954c	22693.55	3.30	0.19	17.61	2.17E-69	1.23E-66
Rv1685c	1596.66	3.28	0.20	16.40	2.06E-60	8.18E-58
Rv1938	1109.64	2.98	0.26	11.45	2.49E-30	1.65E-28
Rv1936	3791.27	2.88	0.32	9.07	1.16E-19	2.91E-18
Rv1937	4320.49	2.87	0.24	11.79	4.29E-32	3.47E-30
Rv3161c	10722.15	2.82	0.29	9.80	1.11E-22	3.83E-21
Rv1955	2145.63	2.79	0.17	16.24	2.82E-59	9.32E-57
Rv0251c	55855.15	2.69	0.21	12.78	2.21E-37	2.74E-35
Rvns01	1873283.11	2.68	0.29	9.21	3.14E-20	8.24E-19
Rv2501c	7276.50	2.63	0.19	13.64	2.44E-42	3.87E-40
Rv0978c	796.39	2.58	0.32	7.94	1.96E-15	2.78E-14
Rv1576c	6563.21	2.57	0.56	4.56	5.02E-06	1.75E-05
Rv3534c	3841.86	2.55	0.13	19.84	1.23E-87	1.22E-84
Rv0834c	22699.06	2.52	0.32	7.83	4.79E-15	6.46E-14
Rv1967	356.65	2.51	0.19	13.51	1.36E-41	2.07E-39
Rv0976c	2686.15	2.47	0.26	9.39	5.85E-21	1.66E-19
Rv1968	404.32	2.47	0.17	14.63	1.81E-48	3.99E-46
Rv3535c	3208.69	2.46	0.13	18.45	4.76E-76	3.14E-73
Rv0969	78469.74	2.44	0.31	7.86	3.69E-15	5.11E-14
Rv2616	4133.76	2.43	0.32	7.69	1.50E-14	1.81E-13
Rv0188	10318.29	2.38	0.23	10.52	6.89E-26	3.21E-24
Rv2617c	3964.39	2.34	0.30	7.87	3.58E-15	4.98E-14
Rv1575	303.95	2.34	0.47	5.00	5.62E-07	2.28E-06
Rv0983	35230.91	2.33	0.18	13.19	1.00E-39	1.37E-37

Rv2615c	2398.78	2.33	0.30	7.72	1.13E-14	1.41E-13
Rv0186A	11152.59	2.33	0.22	10.46	1.29E-25	5.81E-24
Rv1956	1562.89	2.31	0.14	16.33	5.92E-60	2.14E-57
Rv1939	588.15	2.31	0.24	9.47	2.67E-21	7.84E-20
Rv0467	6027.89	2.31	0.27	8.68	4.08E-18	8.31E-17
Rv3202a	632.65	2.29	0.25	9.21	3.12E-20	8.24E-19
Rv3536c	3527.52	2.29	0.13	17.00	7.96E-65	3.95E-62
Rv1992c	17747.03	2.26	0.28	8.03	9.88E-16	1.52E-14
Rv1464	11654.34	2.24	0.24	9.33	1.05E-20	2.94E-19
Rv1577c	4742.59	2.23	0.54	4.13	3.62E-05	1.08E-04
RVnc0046	3227177.44	2.23	0.17	13.25	4.41E-40	6.25E-38
Rv2641	39776.93	2.22	0.35	6.34	2.36E-10	1.60E-09
Rv3160c	3434.62	2.20	0.28	7.85	4.01E-15	5.51E-14
Rv0968	7526.74	2.18	0.26	8.35	7.03E-17	1.27E-15
Rv3176c	2056.52	2.18	0.38	5.70	1.21E-08	6.37E-08
Rvnr02	13981364.18	2.18	0.18	12.43	1.78E-35	1.96E-33
Rv3122	2411.19	2.16	0.33	6.54	6.10E-11	4.48E-10
Rv1966	425.49	2.15	0.21	10.18	2.40E-24	9.19E-23
Rv2052c	22585.80	2.15	0.37	5.84	5.34E-09	2.95E-08
Rv1970	323.28	2.15	0.14	14.83	9.31E-50	2.31E-47
Rv1463	6794.77	2.14	0.23	9.47	2.86E-21	8.34E-20
Rv2503c	2310.56	2.13	0.27	7.92	2.33E-15	3.27E-14
Rv1462	15488.58	2.13	0.24	8.72	2.76E-18	5.76E-17
Rv0975c	1499.54	2.13	0.23	9.27	1.83E-20	5.04E-19
Rv2499c	1408.36	2.12	0.20	10.88	1.44E-27	7.80E-26
Rv3203	2037.54	2.11	0.29	7.30	2.90E-13	3.03E-12
Rv0244c	17242.23	2.11	0.21	10.08	6.85E-24	2.51E-22
Rv2497c	11806.66	2.10	0.15	14.09	4.29E-45	8.50E-43
Rv0752c	5216.06	2.10	0.23	9.25	2.14E-20	5.82E-19
Rv0440	92720.93	2.09	0.18	11.52	1.11E-30	7.70E-29

Rv3743c	1318.22	2.07	0.27	7.60	2.95E-14	3.54E-13
Rv1965	430.40	2.07	0.22	9.47	2.91E-21	8.42E-20
Rv2502c	4243.52	2.05	0.23	9.10	8.66E-20	2.17E-18
Rv3289c	5696.64	2.05	0.20	10.50	8.37E-26	3.86E-24
Rv3290c	65362.88	2.02	0.17	12.21	2.83E-34	2.74E-32
Rv2744c	24517.60	2.02	0.20	10.22	1.63E-24	6.39E-23
Rv3123	1775.71	2.02	0.30	6.74	1.64E-11	1.29E-10
Rv2745c	11446.73	2.01	0.19	10.33	5.11E-25	2.15E-23
Rv2159c	4328.94	2.00	0.25	8.05	8.05E-16	1.27E-14
Rv3417c	22696.05	1.98	0.16	12.54	4.30E-36	4.87E-34
Rv2500c	2803.11	1.98	0.19	10.24	1.26E-24	5.04E-23
Rv0967	12883.61	1.97	0.27	7.33	2.33E-13	2.47E-12
Rv2496c	7609.92	1.97	0.16	12.37	3.75E-35	3.72E-33
Rvnr01	42911.26	1.97	0.32	6.13	8.98E-10	5.52E-09
Rv2086	275.68	1.96	0.25	7.99	1.34E-15	1.97E-14
Rv2642	8252.65	1.96	0.33	6.02	1.78E-09	1.06E-08
Rv1957	710.79	1.94	0.17	11.39	4.54E-30	2.95E-28
Rv0973c	3827.44	1.93	0.25	7.74	1.01E-14	1.29E-13
Rv0751c	3536.18	1.93	0.23	8.46	2.65E-17	5.13E-16
Rv3065	341.83	1.92	0.10	18.52	1.46E-76	1.16E-73
Rv1226c	6020.25	1.92	0.29	6.69	2.25E-11	1.75E-10
Rv1465	2706.11	1.91	0.19	9.80	1.17E-22	4.01E-21
Rv0984	9922.72	1.91	0.17	11.31	1.17E-29	7.50E-28
Rv0974c	1547.19	1.91	0.24	7.93	2.25E-15	3.16E-14
Rv2643	8681.08	1.90	0.30	6.28	3.40E-10	2.25E-09
Rv1808	2096.38	1.90	0.26	7.33	2.23E-13	2.38E-12
Rv1935c	689.95	1.90	0.33	5.82	5.78E-09	3.17E-08
Rv0560c	1127.89	1.90	0.24	8.03	9.79E-16	1.51E-14
Rv0765c	3542.51	1.89	0.25	7.59	3.22E-14	3.83E-13
Rv3206c	19030.02	1.88	0.21	8.92	4.71E-19	1.13E-17

Rv2614A	448.51	1.88	0.28	6.75	1.48E-11	1.18E-10
Rv1578c	661.62	1.88	0.38	4.99	6.02E-07	2.44E-06
Rv0753c	10744.36	1.88	0.23	8.06	7.39E-16	1.17E-14
Rv1940	1611.32	1.87	0.24	7.74	9.67E-15	1.24E-13
Rv0384c	38283.70	1.87	0.23	8.07	7.02E-16	1.12E-14
Rv3175	2072.57	1.87	0.35	5.35	8.69E-08	4.03E-07
Rv3288c	6227.92	1.87	0.21	8.92	4.78E-19	1.14E-17
Rv1654	1340.65	1.85	0.21	8.60	7.66E-18	1.52E-16
Rv2504c	4214.53	1.85	0.27	6.97	3.22E-12	2.82E-11
Rv2710	59214.06	1.84	0.19	9.63	5.71E-22	1.83E-20
Rv3406	2988.56	1.84	0.26	6.97	3.08E-12	2.71E-11
Rv0848	11267.33	1.84	0.30	6.18	6.49E-10	4.09E-09
Rv2558	9642.72	1.83	0.16	11.26	2.06E-29	1.28E-27
Rv1894c	5696.55	1.83	0.23	7.95	1.81E-15	2.60E-14
Rv0186	42514.68	1.82	0.23	8.00	1.21E-15	1.81E-14
Rv0670	4724.62	1.81	0.12	15.21	2.81E-52	7.97E-50
Rv2498c	1876.09	1.81	0.17	10.80	3.43E-27	1.84E-25
Rv2053c	7772.07	1.79	0.30	6.01	1.81E-09	1.07E-08
Rv1461	27963.95	1.79	0.19	9.30	1.41E-20	3.90E-19
Rv2161c	9809.64	1.79	0.29	6.15	7.81E-10	4.84E-09
Rv1991A	610.74	1.78	0.24	7.34	2.14E-13	2.29E-12
Rv1985c	1405.86	1.78	0.20	8.95	3.59E-19	8.78E-18
Rv2554c	3786.43	1.78	0.20	9.11	8.50E-20	2.15E-18
Rv3080c	2239.27	1.77	0.14	13.03	8.26E-39	1.09E-36
Rv0849	3481.36	1.77	0.35	5.04	4.61E-07	1.91E-06
Rv1908c	12342.04	1.76	0.33	5.26	1.48E-07	6.66E-07
Rv3205c	8307.45	1.75	0.20	8.65	5.06E-18	1.02E-16
Rv1574	171.66	1.75	0.40	4.42	9.93E-06	3.29E-05
Rv1806	473.82	1.75	0.31	5.56	2.66E-08	1.32E-07
Rv3533c	1476.52	1.74	0.15	11.64	2.53E-31	1.89E-29

Rv0970	6165.03	1.74	0.32	5.38	7.53E-08	3.53E-07
Rv0764c	4754.62	1.73	0.27	6.42	1.35E-10	9.46E-10
Rv3515c	2025.81	1.73	0.21	8.41	4.24E-17	8.00E-16
Rv3287c	3405.48	1.73	0.19	9.03	1.79E-19	4.45E-18
Rv3834c	995.88	1.72	0.27	6.50	7.88E-11	5.69E-10
Rv3502c	2404.03	1.72	0.13	13.01	1.05E-38	1.35E-36
Rv3503c	755.82	1.72	0.17	10.32	5.66E-25	2.35E-23
Rv2158c	4987.29	1.72	0.20	8.80	1.38E-18	3.06E-17
Rv1653	1936.41	1.71	0.18	9.67	3.88E-22	1.28E-20
Rv1993c	1360.11	1.71	0.29	5.95	2.61E-09	1.51E-08
Rv0351	24828.85	1.70	0.22	7.71	1.27E-14	1.58E-13
Rv0563	9251.82	1.70	0.18	9.64	5.60E-22	1.81E-20
Rv1224	6301.23	1.69	0.25	6.78	1.23E-11	9.94E-11
Rv1227c	1955.75	1.69	0.22	7.59	3.09E-14	3.69E-13
Rv3418c	4037.19	1.69	0.17	9.73	2.24E-22	7.53E-21
Rv3066	785.76	1.68	0.11	14.81	1.29E-49	3.00E-47
Rv0833	3133.59	1.68	0.20	8.45	2.89E-17	5.57E-16
Rv2025c	1174.18	1.68	0.19	8.85	8.45E-19	1.91E-17
Rv1971	281.61	1.68	0.13	12.55	3.78E-36	4.41E-34
Rv0064	8520.63	1.67	0.12	14.22	6.65E-46	1.39E-43
Rv0872c	13817.77	1.66	0.12	13.65	2.07E-42	3.56E-40
Rv1225c	10340.67	1.66	0.28	5.85	5.04E-09	2.79E-08
Rv0352	28109.12	1.66	0.23	7.16	8.18E-13	7.91E-12
RVnc0036a	2792035.86	1.65	0.50	3.29	1.01E-03	2.27E-03
Rv1466	1837.45	1.65	0.17	9.49	2.38E-21	7.06E-20
Rv2620c	1847.43	1.65	0.28	5.88	4.21E-09	2.35E-08
Rv3202c	6622.51	1.65	0.24	6.94	4.03E-12	3.49E-11
Rv2659c	1546.72	1.65	0.48	3.41	6.61E-04	1.54E-03
Rv3836	2216.46	1.63	0.26	6.18	6.53E-10	4.10E-09
Rv1991c	779.29	1.63	0.19	8.37	5.68E-17	1.05E-15

Rv2546	152.71	1.62	0.40	4.07	4.78E-05	1.39E-04
Rv2651c	4155.33	1.61	0.60	2.69	7.11E-03	1.34E-02
Rv2743c	8429.47	1.61	0.23	6.91	4.75E-12	4.07E-11
Rv1670	218.40	1.61	0.22	7.40	1.35E-13	1.49E-12
Rv0550c	126.50	1.61	0.18	8.94	3.86E-19	9.38E-18
Rv2621c	6267.01	1.61	0.26	6.20	5.73E-10	3.65E-09
Rv3837c	8265.39	1.61	0.21	7.56	3.91E-14	4.60E-13
Rv1684	238.89	1.60	0.16	10.32	5.68E-25	2.35E-23
Rv1655	1373.06	1.60	0.18	8.75	2.04E-18	4.40E-17
Rv0233	3193.77	1.59	0.22	7.32	2.44E-13	2.59E-12
Rv0275c	3891.93	1.59	0.13	12.38	3.28E-35	3.34E-33
Rv0847	8331.55	1.58	0.29	5.46	4.80E-08	2.29E-07
Rv0972c	2007.65	1.57	0.24	6.40	1.54E-10	1.07E-09
Rv3627c	5283.52	1.57	0.10	16.14	1.41E-58	4.29E-56
Rv3504	1213.81	1.56	0.11	13.64	2.20E-42	3.64E-40
Rv0142	2815.05	1.55	0.28	5.60	2.19E-08	1.10E-07
Rv1652	683.80	1.55	0.22	6.94	3.92E-12	3.41E-11
Rv2324	562.06	1.55	0.14	10.78	4.30E-27	2.28E-25
Rv2495c	7314.43	1.55	0.17	8.91	5.29E-19	1.25E-17
Rv2555c	31487.01	1.55	0.16	9.51	1.87E-21	5.57E-20
Rv0350	66973.35	1.55	0.19	8.09	5.97E-16	9.67E-15
Rv1460	5943.55	1.54	0.25	6.28	3.31E-10	2.20E-09
Rv2484c	5878.93	1.54	0.13	12.18	3.90E-34	3.59E-32
Rv0762c	1161.88	1.54	0.23	6.76	1.34E-11	1.07E-10
Rv2372c	5491.69	1.54	0.27	5.81	6.34E-09	3.46E-08
Rv0324	486.51	1.54	0.14	11.18	5.33E-29	3.20E-27
Rv0628c	4658.99	1.54	0.13	11.64	2.63E-31	1.93E-29
Rv1934c	569.63	1.54	0.27	5.70	1.22E-08	6.40E-08
Rv0278c	1266.16	1.53	0.15	9.93	3.20E-23	1.11E-21
Rv3863	5665.52	1.53	0.24	6.35	2.11E-10	1.44E-09

Rv0291	12725.60	1.53	0.20	7.76	8.32E-15	1.09E-13
Rv0250c	6779.75	1.52	0.19	8.01	1.18E-15	1.78E-14
Rv1599	7715.64	1.52	0.14	11.13	8.83E-29	5.22E-27
Rv0311	618.17	1.50	0.22	6.70	2.04E-11	1.60E-10
Rv3505	905.24	1.50	0.14	11.06	1.87E-28	1.08E-26
Rv2812	117.51	1.50	0.26	5.74	9.72E-09	5.19E-08
Rv2382c	1909.72	1.49	0.33	4.55	5.43E-06	1.88E-05
Rv2963	3895.02	1.49	0.27	5.58	2.35E-08	1.18E-07
Rv1964	885.28	1.49	0.27	5.47	4.58E-08	2.19E-07
Rv3507	3680.95	1.49	0.13	11.27	1.91E-29	1.20E-27
Rv1950c	180.60	1.49	0.39	3.85	1.19E-04	3.24E-04
Rv2373c	12606.42	1.49	0.24	6.21	5.33E-10	3.42E-09
Rv0971c	2995.01	1.49	0.28	5.30	1.16E-07	5.27E-07
Rv1223	28257.07	1.47	0.21	7.04	1.97E-12	1.78E-11
Rv2280	3799.53	1.47	0.16	9.12	7.57E-20	1.94E-18
Rv2728c	900.00	1.47	0.22	6.74	1.62E-11	1.28E-10
Rv1958c	768.27	1.47	0.16	9.44	3.86E-21	1.11E-19
Rv1832	24411.80	1.46	0.12	12.19	3.40E-34	3.21E-32
Rv1972	155.52	1.46	0.18	8.09	5.75E-16	9.34E-15
Rv0620	120.02	1.46	0.31	4.68	2.83E-06	1.02E-05
Rv0064A	206.11	1.46	0.16	8.86	8.17E-19	1.86E-17
Rv1831	3825.90	1.45	0.14	10.34	4.59E-25	1.98E-23
Rv1651c	3111.32	1.45	0.11	13.34	1.43E-40	2.09E-38
Rv2838c	7022.90	1.45	0.22	6.47	9.97E-11	7.11E-10
Rv3204	634.57	1.45	0.21	6.84	8.06E-12	6.67E-11
Rv1969	254.49	1.44	0.13	11.01	3.30E-28	1.87E-26
Rv0763c	195.06	1.44	0.28	5.11	3.27E-07	1.40E-06
Rv2559c	2237.00	1.44	0.12	11.72	1.03E-31	7.99E-30
Rv2557	5117.29	1.43	0.19	7.58	3.41E-14	4.05E-13
Rv3201c	3239.25	1.43	0.20	7.06	1.62E-12	1.49E-11

Rv0063a	362.06	1.43	0.13	11.00	3.82E-28	2.13E-26
Rv1621c	3177.11	1.42	0.21	6.63	3.34E-11	2.51E-10
Rv2912c	6729.86	1.42	0.21	6.64	3.13E-11	2.38E-10
Rv1818c	4965.10	1.41	0.20	7.16	7.86E-13	7.68E-12
Rv1840c	1468.02	1.40	0.23	6.03	1.68E-09	9.98E-09
Rv3081	1493.51	1.40	0.34	4.13	3.69E-05	1.09E-04
Rv0374c	127.93	1.40	0.31	4.50	6.95E-06	2.37E-05
Rv2064	1945.61	1.40	0.18	7.70	1.38E-14	1.70E-13
Rv0539	4442.78	1.40	0.12	12.15	6.00E-34	5.41E-32
Rv1605	2106.18	1.39	0.19	7.29	3.13E-13	3.24E-12
Rv0564c	4578.71	1.39	0.17	8.16	3.47E-16	5.81E-15
Rv3546	1372.55	1.39	0.17	7.95	1.87E-15	2.68E-14
Rv1963c	825.64	1.39	0.21	6.50	7.78E-11	5.63E-10
Rvnr03	14553.23	1.39	0.32	4.36	1.32E-05	4.27E-05
Rv1586c	5609.13	1.38	0.54	2.57	1.02E-02	1.86E-02
Rv2839c	49575.52	1.38	0.21	6.66	2.67E-11	2.04E-10
Rv2383c	5471.78	1.38	0.28	4.93	8.35E-07	3.28E-06
Rv1259	542.45	1.38	0.24	5.63	1.80E-08	9.20E-08
Rv0162c	899.77	1.38	0.20	6.73	1.70E-11	1.34E-10
Rv2688c	1605.59	1.37	0.08	16.47	6.25E-61	2.75E-58
Rv2570	221.95	1.37	0.16	8.35	6.83E-17	1.25E-15
Rv1801	1630.10	1.37	0.36	3.83	1.31E-04	3.52E-04
Rv2619c	1190.73	1.37	0.24	5.61	2.02E-08	1.02E-07
Rv2677c	3263.02	1.37	0.19	7.24	4.41E-13	4.45E-12
Rv1326c	3834.11	1.37	0.12	11.51	1.23E-30	8.39E-29
Rv1517	244.49	1.36	0.30	4.50	6.70E-06	2.29E-05
Rv2644c	541.59	1.36	0.25	5.45	5.16E-08	2.45E-07
Rv2658c	568.10	1.36	0.38	3.59	3.34E-04	8.25E-04
Rv2051c	15940.12	1.36	0.29	4.70	2.56E-06	9.32E-06
Rv3358	71.90	1.36	0.23	5.92	3.19E-09	1.82E-08

Rv0767c	2782.44	1.34	0.24	5.53	3.26E-08	1.59E-07
Rv0325	112.72	1.34	0.20	6.67	2.59E-11	1.99E-10
Rv2250c	172.15	1.33	0.16	8.53	1.49E-17	2.92E-16
Rv3370c	4781.71	1.33	0.19	7.15	8.90E-13	8.57E-12
Rv2618	2152.98	1.33	0.29	4.56	5.02E-06	1.75E-05
Rv2753c	7324.47	1.33	0.19	6.86	7.00E-12	5.88E-11
Rv2667	559.84	1.32	0.31	4.24	2.24E-05	6.95E-05
Rv0475	12779.67	1.32	0.21	6.18	6.47E-10	4.08E-09
Rv1004c	3431.67	1.32	0.18	7.38	1.61E-13	1.77E-12
Rv1809	2084.48	1.32	0.23	5.66	1.49E-08	7.69E-08
Rv3357	55.60	1.32	0.22	6.00	1.98E-09	1.17E-08
Rv0549c	628.54	1.32	0.12	10.57	3.97E-26	1.92E-24
Rv3188	384.17	1.31	0.19	6.98	2.87E-12	2.55E-11
Rv0123	243.02	1.31	0.17	7.69	1.48E-14	1.80E-13
Rv0326	327.45	1.31	0.21	6.21	5.17E-10	3.34E-09
Rv0846c	3658.11	1.31	0.25	5.19	2.08E-07	9.12E-07
Rv3407	1333.70	1.30	0.18	7.08	1.41E-12	1.31E-11
Rv1492	3448.11	1.30	0.13	10.26	1.02E-24	4.19E-23
Rv2646	778.45	1.30	0.51	2.57	1.01E-02	1.84E-02
Rv2323c	594.16	1.30	0.16	7.94	2.05E-15	2.90E-14
Rv1256c	1926.67	1.29	0.13	9.76	1.68E-22	5.69E-21
Rv0375c	239.39	1.29	0.26	5.04	4.68E-07	1.93E-06
Rv2727c	864.23	1.29	0.21	6.28	3.37E-10	2.24E-09
Rv2552c	3018.09	1.29	0.18	7.17	7.36E-13	7.25E-12
Rv2455c	15782.52	1.29	0.12	10.89	1.32E-27	7.27E-26
Rv1656	795.93	1.29	0.17	7.44	1.04E-13	1.17E-12
Rv1084	3278.89	1.29	0.16	8.31	9.18E-17	1.63E-15
Rv3189	610.73	1.29	0.16	8.12	4.83E-16	7.95E-15
Rv1973	158.75	1.29	0.21	6.25	4.15E-10	2.70E-09
Rv0766c	3300.72	1.29	0.26	4.97	6.59E-07	2.64E-06

Rv0915c	439.67	1.29	0.31	4.16	3.17E-05	9.57E-05
Rv3626c	2087.80	1.29	0.12	10.52	6.65E-26	3.14E-24
Rv0077c	426.11	1.28	0.21	6.12	9.54E-10	5.85E-09
Rv3514	3558.20	1.28	0.30	4.27	1.95E-05	6.13E-05
Rv0747	4220.27	1.28	0.19	6.86	7.04E-12	5.89E-11
Rv1585c	2020.74	1.28	0.49	2.63	8.59E-03	1.59E-02
Rv3367	2461.00	1.28	0.12	10.73	7.23E-27	3.72E-25
Rv0836c	1336.50	1.28	0.25	5.05	4.38E-07	1.82E-06
Rv0290	18074.11	1.28	0.14	9.23	2.72E-20	7.33E-19
Rv2540c	10501.02	1.28	0.20	6.26	3.96E-10	2.60E-09
Rv2515c	1360.00	1.27	0.27	4.71	2.53E-06	9.22E-06
Rv3181c	175.44	1.27	0.21	6.20	5.67E-10	3.62E-09
Rv3549c	1699.04	1.27	0.16	8.15	3.76E-16	6.26E-15
Rv0898c	867.52	1.27	0.17	7.26	3.81E-13	3.87E-12
Rv1706A	988.04	1.26	0.28	4.57	4.86E-06	1.70E-05
Rv3573c	906.02	1.26	0.16	8.12	4.54E-16	7.50E-15
Rv2634c	689.97	1.26	0.11	11.45	2.40E-30	1.61E-28
Rv1221	24970.16	1.26	0.24	5.30	1.18E-07	5.39E-07
Rv3270	73528.43	1.25	0.28	4.43	9.49E-06	3.16E-05
Rv3513c	312.07	1.25	0.17	7.32	2.48E-13	2.62E-12
Rv1960c	231.49	1.25	0.17	7.36	1.87E-13	2.03E-12
Rv3180c	284.83	1.25	0.17	7.27	3.55E-13	3.63E-12
Rv0116c	1630.33	1.25	0.10	12.41	2.20E-35	2.35E-33
Rv2251	1287.95	1.24	0.16	7.99	1.32E-15	1.97E-14
Rv1657	373.43	1.24	0.17	7.48	7.35E-14	8.35E-13
Rv0841	2979.53	1.24	0.31	4.03	5.54E-05	1.60E-04
Rv0279c	1693.39	1.24	0.15	8.50	1.94E-17	3.78E-16
Rv2850c	4477.02	1.23	0.12	10.18	2.41E-24	9.19E-23
Rv2539c	2539.37	1.23	0.22	5.72	1.09E-08	5.80E-08
Rv3286c	2750.54	1.23	0.17	7.18	6.75E-13	6.68E-12

Rv3543c	1218.76	1.23	0.13	9.22	2.91E-20	7.80E-19
Rv2374c	4812.01	1.22	0.11	11.52	1.10E-30	7.70E-29
Rv3538	568.37	1.22	0.18	6.77	1.31E-11	1.05E-10
Rv0373c	563.55	1.22	0.23	5.20	2.03E-07	8.91E-07
Rv2742c	3404.40	1.22	0.22	5.52	3.39E-08	1.65E-07
Rv3409c	8571.91	1.22	0.22	5.60	2.10E-08	1.06E-07
Rv1072	43751.41	1.22	0.14	8.73	2.49E-18	5.23E-17
Rv0874c	2132.60	1.21	0.12	10.19	2.18E-24	8.47E-23
Rv0837c	2538.89	1.21	0.34	3.54	4.05E-04	9.86E-04
Rv0791c	8305.10	1.21	0.29	4.13	3.70E-05	1.10E-04
Rv2647	549.14	1.21	0.55	2.21	2.69E-02	4.45E-02
Rv0985c	2533.37	1.20	0.17	6.95	3.58E-12	3.12E-11
Rv2852c	3287.38	1.20	0.12	9.93	3.04E-23	1.07E-21
Rv0769	1648.36	1.20	0.32	3.80	1.43E-04	3.80E-04
Rv0353	2883.09	1.20	0.20	5.94	2.90E-09	1.66E-08
Rv3061c	6239.75	1.20	0.18	6.82	9.28E-12	7.60E-11
Rv0790c	4629.06	1.20	0.30	4.02	5.80E-05	1.67E-04
Rv1028c	1554.33	1.20	0.08	14.90	3.36E-50	8.89E-48
Rv1604	1971.13	1.20	0.19	6.32	2.67E-10	1.79E-09
Rv3062	3009.31	1.19	0.22	5.39	7.08E-08	3.32E-07
Rv0376c	562.87	1.19	0.20	6.09	1.11E-09	6.67E-09
Rv0411c	3525.91	1.19	0.17	7.12	1.06E-12	1.00E-11
Rv1327c	4616.36	1.18	0.13	9.17	4.74E-20	1.23E-18
Rv1994c	2853.76	1.18	0.27	4.40	1.08E-05	3.55E-05
Rv1222	13394.79	1.18	0.20	5.82	5.87E-09	3.22E-08
Rv1429	471.47	1.18	0.14	8.72	2.78E-18	5.77E-17
Rv1493	2813.57	1.18	0.13	8.79	1.54E-18	3.40E-17
Rv0124	297.45	1.18	0.17	6.88	5.93E-12	5.06E-11
Rv0538	6663.79	1.18	0.19	6.09	1.10E-09	6.63E-09
Rv2553c	7004.97	1.18	0.15	7.78	7.21E-15	9.49E-14

Rv0897c	2318.13	1.18	0.14	8.25	1.58E-16	2.74E-15
Rv2729c	1534.16	1.18	0.25	4.78	1.72E-06	6.45E-06
Rv2011c	1045.20	1.18	0.25	4.79	1.67E-06	6.26E-06
Rv2035	1904.26	1.17	0.26	4.56	5.15E-06	1.79E-05
Rv1802	1198.05	1.17	0.34	3.46	5.41E-04	1.28E-03
Rv3563	405.39	1.16	0.24	4.91	9.18E-07	3.56E-06
Rv3421c	1326.79	1.16	0.22	5.38	7.58E-08	3.55E-07
Rv1909c	704.09	1.16	0.38	3.09	1.98E-03	4.23E-03
Rv1622c	1879.51	1.16	0.22	5.34	9.18E-08	4.24E-07
Rv2483c	3100.12	1.16	0.13	8.88	6.97E-19	1.63E-17
Rv1285	8252.42	1.16	0.21	5.51	3.54E-08	1.72E-07
Rv3545c	1729.91	1.16	0.20	5.91	3.46E-09	1.97E-08
Rv1595	29672.50	1.15	0.27	4.33	1.52E-05	4.86E-05
Rv3420c	1036.91	1.15	0.22	5.27	1.40E-07	6.32E-07
Rv3537	1650.55	1.15	0.18	6.51	7.36E-11	5.36E-10
Rv0331	1542.59	1.15	0.31	3.69	2.21E-04	5.69E-04
Rv2157c	1566.64	1.15	0.18	6.42	1.35E-10	9.46E-10
Rv0574c	2019.42	1.15	0.27	4.30	1.74E-05	5.51E-05
Rv1583c	683.51	1.14	0.45	2.55	1.06E-02	1.91E-02
Rv1959c	485.28	1.14	0.16	7.26	3.91E-13	3.96E-12
Rv3800c	85428.97	1.14	0.16	7.23	4.75E-13	4.77E-12
Rv0329c	621.07	1.13	0.15	7.44	1.01E-13	1.14E-12
Rv0649	241.43	1.13	0.28	4.08	4.58E-05	1.34E-04
Rv2466c	6746.58	1.13	0.26	4.43	9.33E-06	3.11E-05
Rv0288	4830.20	1.13	0.19	5.90	3.55E-09	2.01E-08
Rv1406	1325.74	1.13	0.15	7.35	1.96E-13	2.12E-12
Rv1395	2124.61	1.12	0.26	4.40	1.08E-05	3.55E-05
Rv2811	72.60	1.12	0.31	3.68	2.34E-04	6.01E-04
Rv2851c	1479.18	1.12	0.12	9.54	1.40E-21	4.27E-20
Rv0672	3707.67	1.12	0.14	7.97	1.58E-15	2.30E-14

Rv1854c	4682.78	1.12	0.11	10.40	2.49E-25	1.10E-23
Rv2583c	7608.35	1.12	0.08	13.71	8.95E-43	1.61E-40
Rv0382c	3050.46	1.11	0.22	5.17	2.34E-07	1.02E-06
Rv0486	9100.94	1.11	0.16	6.81	9.81E-12	8.01E-11
Rv2687c	400.48	1.11	0.12	9.54	1.50E-21	4.54E-20
Rv2614c	3341.58	1.11	0.18	6.24	4.49E-10	2.91E-09
Rv0577	2629.95	1.11	0.14	8.08	6.27E-16	1.01E-14
Rv0289	9967.63	1.11	0.19	5.81	6.11E-09	3.34E-08
Rv2036	2325.41	1.11	0.26	4.28	1.84E-05	5.82E-05
Rv0493c	393.48	1.11	0.18	5.98	2.18E-09	1.28E-08
Rv0491	2071.85	1.10	0.15	7.16	8.04E-13	7.82E-12
Rv2134c	3422.99	1.10	0.15	7.40	1.33E-13	1.47E-12
Rv2234	393.00	1.10	0.10	10.68	1.29E-26	6.48E-25
Rv0410c	7211.77	1.10	0.17	6.67	2.61E-11	2.00E-10
Rv3222c	4045.75	1.10	0.22	5.12	3.12E-07	1.34E-06
Rv0865	1247.64	1.10	0.15	7.16	8.19E-13	7.91E-12
Rv0037c	1596.92	1.10	0.12	8.87	7.65E-19	1.75E-17
Rv2731	1934.35	1.10	0.10	10.77	4.71E-27	2.46E-25
Rv2934	21836.91	1.10	0.30	3.69	2.26E-04	5.81E-04
Rv0942	374.65	1.09	0.18	6.18	6.59E-10	4.13E-09
Rv3609c	1781.97	1.09	0.14	7.81	5.65E-15	7.51E-14
Rv0292	3311.56	1.09	0.14	7.97	1.64E-15	2.39E-14
Rv1130	4852.78	1.08	0.32	3.38	7.16E-04	1.65E-03
Rv3552	737.07	1.08	0.22	5.00	5.66E-07	2.30E-06
Rv0372c	111.07	1.08	0.29	3.73	1.92E-04	5.00E-04
Rv3879c	18522.92	1.08	0.18	5.96	2.55E-09	1.48E-08
Rv0654	9865.00	1.08	0.29	3.72	2.02E-04	5.25E-04
Rv1717	229.89	1.08	0.29	3.68	2.35E-04	6.02E-04
Rv0673	1709.14	1.07	0.16	6.84	8.17E-12	6.73E-11
Rv1129c	1025.14	1.07	0.34	3.20	1.37E-03	3.02E-03

Rv1683	5968.59	1.07	0.12	8.81	1.30E-18	2.92E-17
Rv0271c	5421.79	1.07	0.08	13.83	1.69E-43	3.19E-41
Rv0940c	947.16	1.07	0.13	8.32	8.76E-17	1.56E-15
Rv1671	132.48	1.07	0.20	5.43	5.72E-08	2.71E-07
Rv3835	3009.52	1.07	0.21	5.09	3.52E-07	1.49E-06
Rv2162c	14341.38	1.07	0.13	8.16	3.36E-16	5.65E-15
Rv3544c	1146.57	1.07	0.14	7.50	6.15E-14	7.11E-13
Rv2897c	449.65	1.07	0.26	4.11	4.03E-05	1.18E-04
Rv0368c	204.22	1.07	0.25	4.31	1.63E-05	5.18E-05
Rv3433c	1028.82	1.06	0.15	7.13	1.01E-12	9.58E-12
Rv2660c	5961.99	1.06	0.45	2.34	1.92E-02	3.27E-02
Rv0468	1247.28	1.06	0.27	3.94	8.10E-05	2.27E-04
Rv0671	1777.47	1.06	0.12	8.71	3.01E-18	6.19E-17
Rv0122	113.10	1.05	0.18	5.73	9.82E-09	5.24E-08
Rv3564	378.82	1.05	0.22	4.83	1.39E-06	5.27E-06
Rv0381c	2666.34	1.05	0.21	4.99	6.10E-07	2.47E-06
Rv2922A	121.04	1.05	0.14	7.31	2.62E-13	2.76E-12
Rv3347c	7881.43	1.05	0.20	5.19	2.09E-07	9.16E-07
Rv1899c	4488.00	1.04	0.17	6.32	2.65E-10	1.78E-09
Rv0492c	1715.89	1.04	0.16	6.66	2.66E-11	2.04E-10
Rv1715	613.20	1.04	0.26	3.98	6.86E-05	1.96E-04
Rv1584c	1175.19	1.04	0.46	2.25	2.47E-02	4.10E-02
Rv0792c	8843.77	1.04	0.28	3.67	2.44E-04	6.23E-04
Rv3422c	853.32	1.04	0.21	4.95	7.44E-07	2.95E-06
Rv2913c	18124.61	1.04	0.21	4.84	1.32E-06	5.00E-06
Rv0615	962.59	1.03	0.15	6.96	3.45E-12	3.02E-11
Rv0230c	716.38	1.03	0.30	3.40	6.81E-04	1.58E-03
Rv3597c	1888.93	1.03	0.19	5.33	9.72E-08	4.47E-07
Rv1600	2702.08	1.03	0.15	6.70	2.10E-11	1.64E-10
Rv0307c	347.75	1.03	0.23	4.46	8.09E-06	2.72E-05

Rv3608c	1822.43	1.03	0.18	5.66	1.47E-08	7.60E-08
Rv2834c	227.35	1.03	0.25	4.12	3.72E-05	1.10E-04
Rv1391	9365.17	1.03	0.09	12.04	2.22E-33	1.91E-31
Rv0768	5430.57	1.03	0.28	3.61	3.04E-04	7.58E-04
Rv2841c	4119.27	1.03	0.16	6.36	2.01E-10	1.38E-09
Rv0578c	11481.07	1.03	0.23	4.56	5.15E-06	1.79E-05
Rv3838c	5945.17	1.03	0.26	3.90	9.69E-05	2.67E-04
Rv3699	1420.92	1.03	0.13	7.71	1.28E-14	1.58E-13
Rv1817	1568.02	1.03	0.10	10.57	3.98E-26	1.92E-24
Rv3729	3613.28	1.02	0.24	4.34	1.40E-05	4.49E-05
Rv0977	1385.49	1.02	0.11	9.11	8.35E-20	2.12E-18
Rv2063A	200.01	1.02	0.12	8.70	3.46E-18	7.08E-17
Rv3610c	11550.50	1.02	0.11	9.56	1.23E-21	3.82E-20
Rv0648	1550.82	1.02	0.19	5.44	5.42E-08	2.57E-07
Rv3625c	776.10	1.01	0.16	6.39	1.68E-10	1.17E-09
Rv3898c	634.67	1.01	0.14	7.03	2.06E-12	1.86E-11
Rv1573	211.72	1.01	0.47	2.17	3.01E-02	4.93E-02
Rv3607c	832.15	1.01	0.16	6.13	8.69E-10	5.37E-09
Rv3553	821.53	1.01	0.20	4.94	7.65E-07	3.02E-06
Rv1217c	1161.94	1.01	0.28	3.64	2.71E-04	6.85E-04
Rv1945	1615.99	1.01	0.14	7.02	2.26E-12	2.02E-11
Rv1725c	386.15	1.00	0.17	5.92	3.17E-09	1.81E-08
Rv1407	1493.41	1.00	0.15	6.87	6.29E-12	5.33E-11
Rv1601	1191.90	1.00	0.17	5.95	2.69E-09	1.56E-08
Rv0234c	2058.41	1.00	0.08	12.62	1.74E-36	2.09E-34
Rv2870c	2654.70	1.00	0.12	8.01	1.13E-15	1.73E-14
Rv2837c	5832.10	1.00	0.22	4.47	7.67E-06	2.59E-05
Rv3179	692.84	1.00	0.15	6.80	1.08E-11	8.74E-11
Rv2910c	276.78	1.00	0.25	4.05	5.16E-05	1.49E-04
Rv1467c	1573.53	1.00	0.13	7.76	8.59E-15	1.12E-13

Rv0770	1105.54	1.00	0.32	3.10	1.92E-03	4.12E-03
Rv0840c	2771.86	0.99	0.27	3.62	2.96E-04	7.40E-04
Rv1450c	563.42	0.99	0.19	5.16	2.51E-07	1.09E-06
Rv0383c	14050.14	0.99	0.20	4.97	6.61E-07	2.64E-06
Rv0877	3998.76	0.99	0.14	6.87	6.48E-12	5.47E-11
Rv0256c	3531.06	0.99	0.14	7.08	1.42E-12	1.32E-11
Rv3079c	317.44	0.99	0.09	11.10	1.20E-28	7.01E-27
Rv0412c	5151.06	0.98	0.16	6.33	2.40E-10	1.62E-09
Rv2800	2268.52	0.98	0.14	7.12	1.05E-12	1.00E-11
Rv2653c	64.03	0.98	0.42	2.34	1.94E-02	3.30E-02
Rv3167c	284.64	0.98	0.18	5.49	3.96E-08	1.91E-07
Rv2131c	3500.18	0.98	0.17	5.71	1.15E-08	6.05E-08
Rv3580c	4752.59	0.98	0.20	4.95	7.45E-07	2.95E-06
Rv2371	498.80	0.98	0.30	3.24	1.19E-03	2.64E-03
Rv2853	922.02	0.98	0.13	7.28	3.24E-13	3.33E-12
Rv3345c	739.70	0.98	0.25	3.96	7.63E-05	2.15E-04
Rv1623c	4689.28	0.97	0.20	4.84	1.33E-06	5.05E-06
Rv0465c	1075.46	0.97	0.16	6.25	4.01E-10	2.62E-09
Rv3448	151.13	0.97	0.22	4.33	1.51E-05	4.82E-05
Rv1495	144.46	0.96	0.14	7.01	2.33E-12	2.08E-11
Rv1974	111.31	0.96	0.18	5.25	1.52E-07	6.81E-07
Rv1820	2211.69	0.96	0.14	6.75	1.49E-11	1.18E-10
Rv2454c	6793.95	0.96	0.11	8.82	1.18E-18	2.66E-17
Rv3606c	1238.91	0.96	0.18	5.20	1.95E-07	8.61E-07
Rv1803c	884.45	0.96	0.16	6.08	1.18E-09	7.12E-09
Rv3140	6278.70	0.96	0.18	5.17	2.30E-07	1.00E-06
Rv0939	3902.31	0.95	0.10	9.42	4.37E-21	1.25E-19
Rv3735	566.02	0.95	0.16	5.99	2.11E-09	1.24E-08
Rv0065	896.72	0.95	0.19	4.91	9.07E-07	3.52E-06
Rv1842c	3042.22	0.95	0.20	4.71	2.51E-06	9.16E-06

Rv1716	629.23	0.95	0.26	3.68	2.29E-04	5.89E-04
Rv1381	933.27	0.95	0.16	5.78	7.30E-09	3.95E-08
Rv1405c	3206.95	0.95	0.21	4.43	9.39E-06	3.13E-05
Rv3540c	614.07	0.94	0.15	6.45	1.13E-10	7.99E-10
Rv3271c	10118.87	0.94	0.33	2.88	3.99E-03	7.95E-03
Rv1135c	585.86	0.94	0.09	10.60	2.86E-26	1.42E-24
Rv3539	530.75	0.94	0.14	6.77	1.29E-11	1.04E-10
Rv3082c	344.61	0.94	0.19	4.87	1.12E-06	4.29E-06
Rv2678c	2386.19	0.94	0.16	5.79	7.05E-09	3.82E-08
Rv3551	721.82	0.93	0.19	4.84	1.30E-06	4.95E-06
Rv3541c	245.63	0.93	0.18	5.33	9.75E-08	4.48E-07
Rv3471c	60.04	0.93	0.29	3.22	1.29E-03	2.86E-03
Rv2232	546.79	0.93	0.08	12.02	2.67E-33	2.25E-31
Rv1019	1082.94	0.93	0.13	7.16	7.96E-13	7.76E-12
Rv1063c	1401.20	0.93	0.14	6.59	4.47E-11	3.33E-10
Rv3657c	416.26	0.93	0.36	2.62	8.81E-03	1.62E-02
Rv0916c	152.88	0.93	0.24	3.90	9.81E-05	2.69E-04
Rv0293c	2550.24	0.93	0.12	7.50	6.28E-14	7.24E-13
Rv3408	2115.41	0.93	0.13	6.97	3.08E-12	2.71E-11
Rv0189c	5503.26	0.93	0.12	7.75	9.02E-15	1.17E-13
Rv2320c	490.05	0.93	0.15	6.30	3.03E-10	2.02E-09
Rv2164c	3836.54	0.93	0.13	7.12	1.09E-12	1.03E-11
Rv1325c	1254.65	0.93	0.21	4.39	1.12E-05	3.65E-05
Rv0249c	4382.24	0.93	0.18	5.15	2.61E-07	1.13E-06
Rv2465c	2208.91	0.92	0.25	3.77	1.61E-04	4.25E-04
Rv2978c	3923.48	0.92	0.20	4.72	2.31E-06	8.47E-06
Rv3843c	7749.09	0.92	0.12	7.95	1.89E-15	2.70E-14
Rv3230c	7571.57	0.92	0.19	4.75	2.01E-06	7.43E-06
Rv3139	8115.96	0.92	0.15	6.15	7.98E-10	4.95E-09
Rv2933	33785.42	0.92	0.28	3.27	1.08E-03	2.43E-03

Rv1714	848.14	0.92	0.27	3.43	6.10E-04	1.43E-03
Rv3833	774.04	0.92	0.18	5.00	5.61E-07	2.28E-06
Rv1843c	5774.19	0.92	0.15	6.28	3.38E-10	2.24E-09
Rv0118c	482.50	0.92	0.22	4.23	2.33E-05	7.20E-05
Rv3159c	2089.40	0.92	0.23	3.94	8.07E-05	2.26E-04
Rv1082	1854.75	0.92	0.10	9.58	9.81E-22	3.09E-20
Rv1602	983.91	0.91	0.18	5.11	3.18E-07	1.36E-06
Rv0274	888.85	0.91	0.13	7.24	4.52E-13	4.55E-12
Rv0287	3568.85	0.91	0.21	4.39	1.15E-05	3.75E-05
Rv3223c	4423.35	0.91	0.20	4.55	5.41E-06	1.88E-05
Rv3334	924.87	0.91	0.20	4.59	4.51E-06	1.59E-05
Rv3410c	2314.29	0.91	0.14	6.47	1.01E-10	7.16E-10
Rv1620c	1455.80	0.91	0.20	4.58	4.59E-06	1.61E-05
Rv3508	5070.90	0.91	0.29	3.13	1.73E-03	3.76E-03
Rv0328	1216.35	0.91	0.14	6.50	8.07E-11	5.81E-10
Rv2016	649.32	0.90	0.21	4.31	1.60E-05	5.10E-05
Rv0141c	1827.57	0.90	0.23	3.94	8.01E-05	2.25E-04
Rv2249c	654.06	0.90	0.13	6.87	6.44E-12	5.45E-11
Rv1131	2846.63	0.90	0.31	2.86	4.21E-03	8.32E-03
Rv0576	2451.77	0.89	0.18	5.09	3.53E-07	1.49E-06
Rv3341	4182.53	0.89	0.20	4.41	1.05E-05	3.44E-05
Rv1192	6833.02	0.89	0.23	3.89	9.83E-05	2.70E-04
Rv1593c	3596.49	0.89	0.14	6.35	2.18E-10	1.49E-09
Rv1161	45851.64	0.89	0.18	5.04	4.69E-07	1.93E-06
Rv2780	4235.96	0.89	0.22	3.98	6.84E-05	1.95E-04
Rv2263	432.33	0.89	0.14	6.42	1.37E-10	9.54E-10
Rv1148c	1887.48	0.88	0.13	6.67	2.48E-11	1.92E-10
Rv3842c	18996.94	0.88	0.23	3.88	1.05E-04	2.88E-04
Rv3916c	3380.33	0.88	0.10	8.78	1.69E-18	3.68E-17
Rv0986	605.56	0.88	0.22	4.06	4.89E-05	1.42E-04

Rv1603	1710.84	0.88	0.18	4.99	6.17E-07	2.49E-06
Rv0990c	3121.26	0.88	0.30	2.96	3.06E-03	6.24E-03
Rv1529	951.36	0.88	0.16	5.65	1.64E-08	8.43E-08
Rv3516	435.27	0.88	0.20	4.34	1.43E-05	4.58E-05
Rv0386	1378.15	0.88	0.15	5.71	1.12E-08	5.90E-08
Rv2974c	1576.40	0.87	0.22	3.96	7.50E-05	2.12E-04
Rv3590c	1022.10	0.87	0.21	4.17	3.10E-05	9.36E-05
Rv0575c	580.86	0.87	0.19	4.48	7.31E-06	2.48E-05
Rv3517	101.95	0.87	0.34	2.59	9.53E-03	1.74E-02
Rv2017	726.71	0.87	0.19	4.66	3.24E-06	1.16E-05
Rv2829c	1442.54	0.87	0.26	3.37	7.46E-04	1.72E-03
Rv0629c	862.34	0.87	0.15	5.94	2.83E-09	1.63E-08
Rv3437	500.66	0.87	0.13	6.56	5.47E-11	4.06E-10
Rv3574	1649.88	0.87	0.16	5.37	7.81E-08	3.64E-07
Rv3212	10798.58	0.87	0.18	4.88	1.05E-06	4.05E-06
Rv0313	3855.95	0.86	0.14	6.08	1.21E-09	7.26E-09
Rv1382	189.79	0.86	0.17	4.93	8.09E-07	3.18E-06
Rv1841c	2131.04	0.86	0.23	3.74	1.81E-04	4.73E-04
Rv1124	484.47	0.86	0.17	5.16	2.49E-07	1.08E-06
Rv3542c	689.11	0.86	0.15	5.88	4.00E-09	2.25E-08
Rv2550c	163.13	0.85	0.23	3.62	2.89E-04	7.25E-04
Rv0330c	463.77	0.85	0.20	4.22	2.45E-05	7.54E-05
Rv1265	3298.13	0.85	0.19	4.36	1.32E-05	4.27E-05
Rv0761c	3020.83	0.84	0.13	6.50	7.89E-11	5.69E-10
Rv2358	1287.50	0.84	0.15	5.71	1.11E-08	5.90E-08
Rv2461c	3067.10	0.84	0.14	6.11	9.75E-10	5.97E-09
Rv1718	453.00	0.84	0.23	3.64	2.68E-04	6.79E-04
Rv0266c	2286.55	0.84	0.14	6.13	8.91E-10	5.49E-09
Rv0580c	734.80	0.84	0.20	4.21	2.61E-05	7.95E-05
Rv2801c	234.95	0.84	0.13	6.35	2.21E-10	1.50E-09

Rv2494	869.65	0.84	0.17	4.87	1.13E-06	4.34E-06
Rv0140	2115.11	0.84	0.24	3.54	4.08E-04	9.92E-04
Rv0487	1172.85	0.84	0.24	3.51	4.44E-04	1.07E-03
Rv2381c	2091.35	0.84	0.26	3.22	1.30E-03	2.89E-03
Rv1890c	157.38	0.84	0.20	4.15	3.30E-05	9.89E-05
Rv0982	7314.19	0.83	0.11	7.28	3.22E-13	3.31E-12
Rv0810c	2964.96	0.83	0.17	5.04	4.72E-07	1.94E-06
Rv0492A	84.45	0.83	0.17	5.01	5.57E-07	2.27E-06
Rv2488c	967.48	0.83	0.11	7.84	4.57E-15	6.18E-14
Rv1162	12260.51	0.83	0.18	4.70	2.54E-06	9.26E-06
Rv1931c	184.10	0.83	0.14	5.82	5.77E-09	3.17E-08
Rv0197	742.28	0.83	0.16	5.10	3.46E-07	1.48E-06
Rv2980	388.95	0.82	0.12	7.04	1.87E-12	1.71E-11
Rv3562	459.43	0.82	0.19	4.41	1.05E-05	3.44E-05
Rv0784	422.23	0.82	0.16	5.15	2.60E-07	1.12E-06
Rv3581c	5622.26	0.82	0.16	5.07	3.88E-07	1.62E-06
Rv0650	287.29	0.82	0.21	3.90	9.53E-05	2.63E-04
Rv0746	634.13	0.82	0.17	4.90	9.77E-07	3.78E-06
Rv1087	1734.12	0.82	0.19	4.31	1.63E-05	5.18E-05
Rv1091	1468.77	0.82	0.18	4.65	3.40E-06	1.22E-05
Rv1430	1407.29	0.82	0.12	6.60	4.22E-11	3.16E-10
Rv1013	4129.22	0.82	0.20	4.12	3.74E-05	1.11E-04
Rv3010c	1636.31	0.82	0.11	7.22	5.14E-13	5.13E-12
Rv2527	330.80	0.82	0.23	3.60	3.13E-04	7.80E-04
Rv3854c	8943.39	0.82	0.26	3.19	1.45E-03	3.18E-03
Rv3340	3521.43	0.82	0.21	3.83	1.27E-04	3.42E-04
Rv1767	570.63	0.82	0.18	4.65	3.39E-06	1.22E-05
Rv0297	7641.59	0.82	0.27	3.02	2.53E-03	5.25E-03
Rv1331	1256.71	0.82	0.24	3.37	7.45E-04	1.72E-03
Rv2923c	190.16	0.82	0.15	5.46	4.73E-08	2.26E-07

Rv1336	2341.09	0.81	0.19	4.30	1.73E-05	5.48E-05
Rv3530c	620.02	0.81	0.26	3.13	1.72E-03	3.74E-03
Rv3419c	1899.36	0.81	0.21	3.92	8.79E-05	2.44E-04
Rv1726	276.26	0.81	0.11	7.14	9.19E-13	8.78E-12
Rv1075c	636.74	0.81	0.20	4.02	5.80E-05	1.67E-04
Rv1383	460.61	0.81	0.18	4.36	1.29E-05	4.19E-05
Rv1638	6328.58	0.81	0.08	9.63	5.88E-22	1.87E-20
Rv1627c	5386.26	0.80	0.14	5.82	5.77E-09	3.17E-08
Rv3531c	2193.35	0.80	0.22	3.68	2.37E-04	6.06E-04
Rv1380	334.80	0.80	0.19	4.17	3.02E-05	9.13E-05
Rv1255c	854.97	0.80	0.12	6.65	2.90E-11	2.21E-10
Rv0343	2219.88	0.80	0.10	8.22	2.07E-16	3.58E-15
Rv2224c	3814.96	0.80	0.14	5.53	3.22E-08	1.58E-07
Rv0851c	769.16	0.80	0.26	3.10	1.95E-03	4.18E-03
Rv1286	9438.35	0.80	0.26	3.08	2.05E-03	4.36E-03
Rv1266c	2590.71	0.80	0.13	6.20	5.75E-10	3.66E-09
Rv1893	399.43	0.79	0.16	4.87	1.11E-06	4.29E-06
Rv3658c	696.69	0.79	0.23	3.43	6.06E-04	1.42E-03
Rv3841	20610.91	0.79	0.23	3.42	6.23E-04	1.46E-03
Rv0398c	703.45	0.79	0.14	5.53	3.20E-08	1.57E-07
Rv0150c	232.64	0.79	0.19	4.28	1.89E-05	5.95E-05
Rv0327c	822.18	0.79	0.28	2.88	3.99E-03	7.95E-03
Rv2964	679.19	0.79	0.21	3.71	2.07E-04	5.38E-04
Rv1422	1962.84	0.79	0.15	5.25	1.50E-07	6.75E-07
Rv1332	2776.82	0.79	0.18	4.28	1.85E-05	5.85E-05
Rv1594	16923.79	0.79	0.22	3.62	2.98E-04	7.45E-04
Rv2865	290.66	0.79	0.17	4.62	3.90E-06	1.38E-05
Rv0875c	1386.02	0.79	0.14	5.57	2.61E-08	1.29E-07
Rv3862c	704.34	0.79	0.20	3.92	8.78E-05	2.44E-04
Rv1986	567.31	0.79	0.20	3.97	7.22E-05	2.05E-04

Rv3093c	2053.95	0.79	0.26	2.98	2.92E-03	5.98E-03
Rv0347	1170.67	0.79	0.16	4.77	1.81E-06	6.75E-06
Rv2340c	1872.76	0.78	0.11	7.35	2.04E-13	2.18E-12
Rv2711	8867.16	0.78	0.13	5.96	2.58E-09	1.50E-08
Rv0400c	1567.29	0.78	0.11	6.84	8.13E-12	6.72E-11
Rv3656c	88.99	0.78	0.22	3.53	4.18E-04	1.02E-03
Rv1805c	1448.10	0.78	0.19	4.16	3.21E-05	9.65E-05
Rv3639c	85.90	0.78	0.24	3.24	1.20E-03	2.66E-03
Rv1305	1865.29	0.78	0.21	3.66	2.54E-04	6.48E-04
Rv0211	7785.41	0.78	0.13	5.90	3.74E-09	2.12E-08
Rv1782	8161.95	0.78	0.12	6.70	2.15E-11	1.67E-10
Rv2516c	3760.30	0.77	0.25	3.05	2.25E-03	4.76E-03
Rv1496	705.03	0.77	0.12	6.33	2.44E-10	1.64E-09
Rv3595c	3134.57	0.77	0.22	3.57	3.59E-04	8.84E-04
Rv0258c	272.03	0.77	0.11	6.85	7.44E-12	6.19E-11
Rv2977c	1833.23	0.77	0.22	3.44	5.84E-04	1.38E-03
Rv0811c	2475.18	0.77	0.10	7.98	1.51E-15	2.21E-14
Rv0332	1062.13	0.77	0.20	3.93	8.50E-05	2.37E-04
Rv2092c	5585.17	0.77	0.28	2.76	5.79E-03	1.11E-02
Rv0505c	1834.23	0.77	0.10	8.04	9.31E-16	1.45E-14
Rv1845c	3882.53	0.77	0.17	4.53	5.83E-06	2.02E-05
Rv0121c	214.48	0.76	0.13	5.71	1.11E-08	5.87E-08
Rv2319c	216.23	0.76	0.12	6.55	5.58E-11	4.13E-10
Rv2065	596.31	0.76	0.19	3.94	8.20E-05	2.30E-04
Rv0674	862.00	0.76	0.20	3.83	1.27E-04	3.42E-04
RVnc0008	723.71	0.76	0.18	4.28	1.89E-05	5.94E-05
Rv0922	10368.11	0.76	0.14	5.56	2.71E-08	1.34E-07
Rv2843	482.81	0.76	0.13	5.71	1.15E-08	6.05E-08
Rv0876c	3339.09	0.76	0.13	5.76	8.47E-09	4.55E-08
Rv3446c	84.65	0.76	0.25	3.00	2.71E-03	5.59E-03

Rv1635c	1603.61	0.76	0.10	7.76	8.68E-15	1.13E-13
Rv2235	726.74	0.75	0.08	8.91	5.09E-19	1.21E-17
Rv3897c	902.97	0.75	0.13	6.03	1.63E-09	9.73E-09
Rv2034	1620.75	0.75	0.25	3.06	2.20E-03	4.66E-03
Rv0389	844.27	0.75	0.09	8.40	4.45E-17	8.37E-16
Rv0991c	4099.70	0.75	0.24	3.08	2.05E-03	4.36E-03
Rv2524c	89069.74	0.75	0.27	2.79	5.19E-03	1.01E-02
Rv2153c	2669.95	0.75	0.15	4.96	7.17E-07	2.85E-06
Rv2712c	3249.01	0.74	0.25	3.02	2.50E-03	5.21E-03
Rv1086	1853.79	0.74	0.12	6.35	2.18E-10	1.49E-09
Rv0614	115.86	0.74	0.20	3.69	2.26E-04	5.81E-04
Rv1149	900.87	0.74	0.15	5.04	4.70E-07	1.94E-06
Rv0441c	1505.18	0.74	0.09	8.14	3.94E-16	6.54E-15
Rv2887	449.97	0.74	0.16	4.76	1.95E-06	7.23E-06
Rv0261c	194.66	0.74	0.19	3.94	8.18E-05	2.29E-04
Rv3673c	1487.61	0.74	0.22	3.30	9.58E-04	2.17E-03
Rv0190	2165.28	0.74	0.18	4.02	5.70E-05	1.64E-04
Rv2544	105.54	0.74	0.33	2.20	2.79E-02	4.60E-02
Rv2830c	347.70	0.74	0.15	4.76	1.96E-06	7.24E-06
Rv0842	4374.60	0.73	0.16	4.60	4.33E-06	1.53E-05
Rv3350c	3957.65	0.73	0.10	7.72	1.13E-14	1.41E-13
Rv1333	2499.37	0.73	0.16	4.72	2.35E-06	8.63E-06
Rv1020	4372.11	0.73	0.16	4.49	7.08E-06	2.41E-05
Rv3582c	16619.89	0.73	0.10	7.05	1.85E-12	1.70E-11
Rv2849c	883.85	0.73	0.12	6.18	6.52E-10	4.10E-09
Rv1929c	1030.71	0.73	0.17	4.26	2.03E-05	6.32E-05
Rv2946c	6066.82	0.73	0.27	2.65	8.10E-03	1.51E-02
Rv3342	2351.09	0.73	0.22	3.32	9.01E-04	2.04E-03
Rv1159A	540.56	0.73	0.24	3.03	2.46E-03	5.13E-03
Rv1073	10023.42	0.73	0.16	4.42	9.65E-06	3.20E-05

Rv0530	4477.65	0.73	0.11	6.36	2.03E-10	1.39E-09
Rv1926c	4256.43	0.73	0.26	2.80	5.04E-03	9.81E-03
Rv1569	1215.36	0.72	0.18	3.92	8.71E-05	2.42E-04
Rv2133c	1797.81	0.72	0.13	5.48	4.23E-08	2.03E-07
Rv2151c	1717.68	0.72	0.18	4.03	5.63E-05	1.62E-04
Rv2638	915.72	0.72	0.18	4.01	6.04E-05	1.73E-04
Rv2333c	883.44	0.72	0.18	3.96	7.64E-05	2.15E-04
Rv3169	661.29	0.72	0.20	3.61	3.12E-04	7.77E-04
Rv1540	2809.97	0.72	0.15	4.84	1.30E-06	4.95E-06
Rv0185	1461.68	0.72	0.25	2.92	3.44E-03	6.95E-03
Rv2551c	842.05	0.72	0.19	3.83	1.26E-04	3.40E-04
Rv0209	491.35	0.72	0.13	5.73	9.99E-09	5.33E-08
Rv0864	2570.22	0.72	0.15	4.71	2.44E-06	8.93E-06
Rv1628c	1388.80	0.72	0.10	7.02	2.16E-12	1.95E-11
Rv0132c	480.77	0.72	0.13	5.52	3.36E-08	1.64E-07
Rv3728	3263.62	0.71	0.31	2.33	1.99E-02	3.38E-02
Rv2911	2341.30	0.71	0.20	3.49	4.90E-04	1.17E-03
Rv1330c	655.83	0.71	0.12	5.83	5.68E-09	3.14E-08
Rv3568c	2338.99	0.71	0.17	4.07	4.69E-05	1.37E-04
Rv1306	3723.18	0.71	0.21	3.36	7.66E-04	1.76E-03
Rv1278	2825.27	0.71	0.13	5.47	4.52E-08	2.17E-07
Rv1234	1237.86	0.70	0.14	5.06	4.27E-07	1.78E-06
Rv2918c	2961.85	0.70	0.24	2.98	2.84E-03	5.84E-03
Rv0413	999.69	0.70	0.13	5.58	2.42E-08	1.21E-07
Rv2156c	1168.52	0.70	0.16	4.47	7.75E-06	2.62E-05
Rv0191	2932.70	0.70	0.24	2.90	3.77E-03	7.55E-03
Rv1568	806.89	0.70	0.16	4.29	1.83E-05	5.78E-05
Rv0581	163.40	0.70	0.24	2.87	4.15E-03	8.22E-03
Rv0348	771.72	0.70	0.13	5.30	1.14E-07	5.21E-07
Rv2449c	1897.35	0.70	0.11	6.52	7.04E-11	5.14E-10

Rv3844	231.19	0.70	0.13	5.36	8.40E-08	3.89E-07
Rv1468c	1117.14	0.69	0.18	3.95	7.94E-05	2.23E-04
Rv3605c	1005.47	0.69	0.16	4.28	1.89E-05	5.95E-05
Rv2801A	77.44	0.69	0.18	3.82	1.35E-04	3.62E-04
Rv1658	1103.09	0.69	0.16	4.22	2.45E-05	7.55E-05
Rv1228	2042.95	0.69	0.18	3.85	1.16E-04	3.14E-04
Rv3423c	1643.14	0.69	0.17	4.13	3.64E-05	1.08E-04
Rv0553	352.44	0.69	0.17	4.16	3.19E-05	9.62E-05
Rv0022c	57.68	0.69	0.30	2.27	2.30E-02	3.85E-02
Rv0727c	544.76	0.69	0.09	7.75	9.33E-15	1.20E-13
Rv3653	177.63	0.69	0.27	2.51	1.21E-02	2.16E-02
Rv3708c	2874.35	0.69	0.17	4.16	3.17E-05	9.57E-05
Rv1163	2714.30	0.69	0.19	3.58	3.50E-04	8.62E-04
Rv2694c	1921.56	0.69	0.15	4.48	7.41E-06	2.51E-05
Rv0941c	767.08	0.69	0.19	3.59	3.34E-04	8.25E-04
Rv1335	812.07	0.69	0.14	4.95	7.54E-07	2.98E-06
Rv1152	736.13	0.69	0.17	3.97	7.28E-05	2.06E-04
Rv2252	416.85	0.68	0.13	5.28	1.30E-07	5.90E-07
Rv0808	7706.96	0.68	0.13	5.26	1.44E-07	6.48E-07
Rv1263	442.93	0.68	0.19	3.56	3.77E-04	9.25E-04
Rv0024	723.99	0.68	0.14	4.85	1.23E-06	4.70E-06
Rv1235	1423.32	0.68	0.12	5.86	4.67E-09	2.60E-08
Rv0194	601.70	0.68	0.16	4.36	1.31E-05	4.25E-05
Rv0612	706.07	0.68	0.16	4.31	1.67E-05	5.30E-05
Rv0276	4089.53	0.68	0.22	3.09	2.02E-03	4.31E-03
Rv2921c	2129.65	0.68	0.10	6.80	1.08E-11	8.74E-11
Rv0262c	557.62	0.68	0.15	4.51	6.46E-06	2.21E-05
Rv2216	1702.92	0.68	0.13	5.14	2.78E-07	1.20E-06
Rv3450c	559.07	0.67	0.17	4.06	4.89E-05	1.42E-04
Rv3238c	583.55	0.67	0.16	4.18	2.96E-05	8.96E-05

Rv2670c	390.24	0.67	0.22	3.00	2.67E-03	5.51E-03
Rv2707	8628.83	0.67	0.23	2.90	3.72E-03	7.45E-03
Rv0616A	655.73	0.67	0.09	7.57	3.76E-14	4.45E-13
Rv1133c	30038.50	0.67	0.17	4.03	5.68E-05	1.64E-04
Rv2871	123.22	0.67	0.17	3.81	1.41E-04	3.76E-04
Rv3250c	856.19	0.66	0.31	2.17	2.99E-02	4.90E-02
Rv2613c	644.31	0.66	0.15	4.47	7.96E-06	2.68E-05
Rv2207	2013.15	0.66	0.17	3.93	8.66E-05	2.41E-04
Rv2281	535.96	0.66	0.25	2.68	7.31E-03	1.37E-02
Rv0283	12426.20	0.66	0.22	3.02	2.52E-03	5.25E-03
Rv2996c	2591.97	0.66	0.11	6.19	6.00E-10	3.81E-09
Rv2979c	1250.07	0.66	0.19	3.53	4.10E-04	9.96E-04
Rv0602c	92.32	0.66	0.18	3.68	2.35E-04	6.01E-04
Rv2264c	978.80	0.66	0.09	7.30	2.80E-13	2.93E-12
Rv0371c	71.75	0.66	0.27	2.47	1.36E-02	2.39E-02
Rv0212c	1223.94	0.66	0.19	3.55	3.82E-04	9.34E-04
Rv1329c	1359.66	0.66	0.13	4.99	5.97E-07	2.42E-06
Rv3293	1497.68	0.66	0.12	5.55	2.87E-08	1.41E-07
Rv0245	818.69	0.66	0.10	6.75	1.52E-11	1.21E-10
Rv0449c	1578.40	0.66	0.26	2.54	1.11E-02	2.00E-02
Rv0511	15922.62	0.66	0.22	2.95	3.15E-03	6.40E-03
Rv3881c	7021.78	0.65	0.14	4.59	4.38E-06	1.54E-05
Rv1323	9328.08	0.65	0.13	4.93	8.17E-07	3.21E-06
Rv1853	629.91	0.65	0.12	5.59	2.21E-08	1.11E-07
Rv2523c	1343.50	0.65	0.29	2.26	2.39E-02	3.98E-02
Rv3556c	2413.40	0.65	0.13	5.00	5.66E-07	2.30E-06
Rv3411c	7774.82	0.65	0.10	6.19	5.91E-10	3.76E-09
Rv0259c	162.34	0.65	0.20	3.18	1.45E-03	3.18E-03
Rv3296	5707.41	0.65	0.19	3.42	6.35E-04	1.48E-03
Rv1570	671.09	0.65	0.22	2.93	3.42E-03	6.91E-03

Rv0196	89.11	0.65	0.19	3.41	6.55E-04	1.52E-03
Rv1375	2362.01	0.65	0.19	3.41	6.40E-04	1.49E-03
Rv0306	219.75	0.65	0.16	3.95	7.75E-05	2.18E-04
Rv1337	1678.18	0.64	0.23	2.78	5.39E-03	1.04E-02
Rv2517c	1111.64	0.64	0.24	2.63	8.52E-03	1.58E-02
Rv1961	377.41	0.64	0.18	3.67	2.46E-04	6.28E-04
Rv0092	1689.57	0.64	0.11	5.93	3.10E-09	1.78E-08
Rv0744c	318.35	0.64	0.16	4.05	5.21E-05	1.51E-04
Rv0903c	2550.27	0.64	0.13	4.79	1.64E-06	6.14E-06
Rv1068c	166.97	0.64	0.26	2.49	1.29E-02	2.30E-02
Rv0339c	1511.26	0.64	0.13	4.78	1.78E-06	6.64E-06
Rv3895c	922.63	0.64	0.12	5.53	3.19E-08	1.56E-07
Rv0378	146.69	0.64	0.28	2.30	2.14E-02	3.60E-02
Rv2379c	2827.27	0.64	0.19	3.39	6.99E-04	1.62E-03
Rv2848c	1391.15	0.63	0.13	4.77	1.89E-06	7.00E-06
Rv3861	158.50	0.63	0.24	2.68	7.30E-03	1.37E-02
Rv3269	10018.10	0.63	0.20	3.13	1.74E-03	3.76E-03
Rv3654c	319.61	0.63	0.22	2.84	4.46E-03	8.74E-03
Rv1539	1985.65	0.63	0.19	3.41	6.39E-04	1.49E-03
Rv1779c	2781.40	0.63	0.15	4.20	2.63E-05	8.03E-05
Rv0728c	499.68	0.63	0.18	3.45	5.70E-04	1.34E-03
Rv3447c	214.14	0.63	0.22	2.85	4.31E-03	8.49E-03
Rv1040c	462.14	0.63	0.22	2.87	4.11E-03	8.15E-03
Rv0232	1677.29	0.62	0.22	2.84	4.44E-03	8.73E-03
Rv1449c	7472.50	0.62	0.10	6.50	8.14E-11	5.85E-10
Rv1922	463.69	0.62	0.15	4.11	4.02E-05	1.18E-04
Rv3104c	1699.30	0.62	0.12	5.19	2.09E-07	9.16E-07
Rv1384	1911.84	0.62	0.15	4.13	3.70E-05	1.10E-04
Rv0160c	247.56	0.62	0.16	4.00	6.22E-05	1.78E-04
Rv2326c	7566.84	0.62	0.19	3.33	8.61E-04	1.96E-03

Rv0528	3262.19	0.62	0.25	2.44	1.48E-02	2.59E-02
Rv0755c	924.96	0.62	0.17	3.74	1.85E-04	4.83E-04
Rv2708c	586.18	0.62	0.21	2.89	3.88E-03	7.76E-03
Rv0349	623.12	0.62	0.15	4.19	2.85E-05	8.65E-05
RVnc0013	201.16	0.62	0.27	2.32	2.06E-02	3.48E-02
Rv0425c	15218.83	0.62	0.15	4.13	3.60E-05	1.07E-04
Rv2202c	7643.77	0.62	0.12	5.08	3.80E-07	1.60E-06
Rv2833c	311.85	0.62	0.24	2.57	1.02E-02	1.84E-02
Rv3227	1422.92	0.62	0.18	3.48	4.99E-04	1.19E-03
Rv1390	4848.07	0.62	0.16	3.75	1.77E-04	4.65E-04
Rv2835c	421.91	0.62	0.27	2.28	2.25E-02	3.77E-02
Rv2138	1875.63	0.61	0.13	4.81	1.49E-06	5.64E-06
Rv3506	367.47	0.61	0.13	4.75	2.06E-06	7.60E-06
Rv2528c	405.06	0.61	0.16	3.93	8.49E-05	2.37E-04
Rv1634	2366.55	0.61	0.14	4.24	2.19E-05	6.81E-05
Rv2230c	736.22	0.61	0.14	4.41	1.02E-05	3.37E-05
Rv1083	367.05	0.61	0.09	6.57	5.05E-11	3.76E-10
Rv0341	5394.42	0.61	0.25	2.47	1.35E-02	2.39E-02
Rv0530A	359.58	0.61	0.17	3.56	3.65E-04	8.96E-04
Rv1990c	550.74	0.61	0.13	4.57	4.96E-06	1.73E-05
Rv1396c	10298.54	0.61	0.24	2.57	1.01E-02	1.84E-02
Rv0415	1002.58	0.61	0.11	5.62	1.88E-08	9.58E-08
Rv2676c	1042.21	0.61	0.09	6.43	1.26E-10	8.85E-10
Rv1452c	499.85	0.61	0.19	3.19	1.42E-03	3.13E-03
Rv0561c	558.19	0.61	0.09	6.50	8.27E-11	5.93E-10
Rv0397A	273.07	0.61	0.14	4.49	7.10E-06	2.42E-05
Rv2359	755.92	0.61	0.13	4.81	1.53E-06	5.78E-06
Rv3178	79.03	0.61	0.22	2.73	6.30E-03	1.20E-02
Rv2125	1895.95	0.61	0.15	4.07	4.61E-05	1.35E-04
Rv3306c	1856.97	0.61	0.15	3.98	6.92E-05	1.97E-04

Rv0414c	480.93	0.61	0.13	4.53	5.92E-06	2.04E-05
Rv1647	1532.33	0.61	0.18	3.40	6.66E-04	1.55E-03
Rv1895	1287.45	0.60	0.20	3.00	2.70E-03	5.57E-03
Rv3094c	1906.86	0.60	0.25	2.45	1.41E-02	2.48E-02
Rv3866	5378.83	0.60	0.12	5.24	1.60E-07	7.16E-07
Rv3548c	1159.89	0.60	0.13	4.80	1.59E-06	5.99E-06
Rv0477	2740.05	0.60	0.12	4.86	1.16E-06	4.46E-06
Rv3356c	1029.97	0.60	0.12	4.84	1.31E-06	5.00E-06
Rv2842c	3147.29	0.60	0.16	3.67	2.42E-04	6.18E-04
Rv2549c	247.76	0.60	0.24	2.55	1.08E-02	1.94E-02
Rv3851	194.98	0.60	0.23	2.59	9.70E-03	1.77E-02
Rv0958	1525.12	0.60	0.12	4.83	1.36E-06	5.14E-06
Rv1962A	195.65	0.60	0.15	4.13	3.58E-05	1.07E-04
Rv0512	6693.46	0.60	0.26	2.35	1.86E-02	3.18E-02
Rv1494	235.83	0.60	0.16	3.76	1.67E-04	4.41E-04
Rv2460c	3627.32	0.60	0.12	4.99	6.04E-07	2.44E-06
Rv0687	1779.05	0.60	0.16	3.75	1.77E-04	4.64E-04
Rv1976c	656.47	0.60	0.11	5.19	2.09E-07	9.15E-07
Rv2867c	6639.52	0.60	0.12	4.98	6.43E-07	2.58E-06
Rv1193	2504.48	0.59	0.25	2.40	1.66E-02	2.88E-02
Rv2010	2258.97	0.59	0.16	3.59	3.26E-04	8.08E-04
Rv1187	1000.54	0.59	0.20	2.98	2.85E-03	5.85E-03
Rv0938	3748.48	0.59	0.12	4.77	1.83E-06	6.81E-06
Rv3559c	304.46	0.59	0.11	5.37	7.87E-08	3.66E-07
Rv0758	3456.46	0.59	0.08	7.29	3.09E-13	3.20E-12
Rv1930c	51.92	0.59	0.19	3.03	2.48E-03	5.16E-03
Rv0342	2630.12	0.59	0.14	4.08	4.56E-05	1.33E-04
Rv1364c	1851.03	0.59	0.08	7.04	1.94E-12	1.77E-11
Rv2215	5906.45	0.58	0.14	4.29	1.81E-05	5.74E-05
Rv3291c	461.83	0.58	0.20	2.96	3.10E-03	6.31E-03

Rv2639c	844.12	0.58	0.20	2.92	3.51E-03	7.07E-03
Rv0668	59870.36	0.58	0.21	2.78	5.45E-03	1.05E-02
Rv0143c	2325.49	0.58	0.16	3.68	2.37E-04	6.06E-04
Rv3348	231.41	0.58	0.14	4.22	2.50E-05	7.66E-05
Rv2395	1262.66	-0.58	0.18	-3.28	1.04E-03	2.35E-03
Rv1932	629.29	-0.58	0.16	-3.57	3.60E-04	8.84E-04
Rv3678A	226.21	-0.58	0.22	-2.69	7.20E-03	1.35E-02
Rv2532c	533.46	-0.58	0.14	-4.21	2.56E-05	7.82E-05
Rv3101c	575.78	-0.58	0.13	-4.57	4.81E-06	1.69E-05
Rv0013	952.20	-0.58	0.10	-5.71	1.15E-08	6.05E-08
Rv0470A	170.21	-0.58	0.16	-3.64	2.72E-04	6.86E-04
Rv3133c	4604.43	-0.58	0.23	-2.56	1.05E-02	1.90E-02
Rv3481c	182.31	-0.59	0.13	-4.45	8.79E-06	2.94E-05
Rv3032A	130.37	-0.59	0.13	-4.40	1.06E-05	3.48E-05
Rv1521	2679.03	-0.59	0.22	-2.72	6.47E-03	1.23E-02
Rv0913c	687.84	-0.59	0.13	-4.44	9.03E-06	3.02E-05
Rv3495c	931.27	-0.59	0.13	-4.43	9.26E-06	3.09E-05
Rv3768	101.08	-0.59	0.20	-2.94	3.33E-03	6.74E-03
Rv3818	666.70	-0.59	0.16	-3.76	1.70E-04	4.47E-04
Rv3435c	910.14	-0.59	0.12	-4.95	7.55E-07	2.99E-06
Rv3457c	7087.72	-0.59	0.14	-4.29	1.79E-05	5.66E-05
Rv2166c	2849.53	-0.60	0.22	-2.74	6.22E-03	1.19E-02
Rv1675c	56.16	-0.60	0.20	-3.04	2.40E-03	5.03E-03
Rv0885	3342.04	-0.60	0.27	-2.22	2.64E-02	4.37E-02
Rv2361c	702.80	-0.60	0.13	-4.54	5.69E-06	1.97E-05
Rv3032	619.40	-0.61	0.15	-4.02	5.86E-05	1.68E-04
Rv2259	1235.88	-0.61	0.12	-5.23	1.67E-07	7.46E-07
Rv3488	134.25	-0.61	0.18	-3.35	8.10E-04	1.85E-03
Rv3792	698.84	-0.61	0.23	-2.65	7.98E-03	1.49E-02
Rv0733	715.92	-0.61	0.20	-3.01	2.60E-03	5.39E-03

Rv3191c	1224.65	-0.61	0.17	-3.63	2.81E-04	7.07E-04
Rv1564c	1008.43	-0.61	0.09	-6.41	1.45E-10	1.01E-09
Rv0058	3849.83	-0.61	0.14	-4.51	6.52E-06	2.23E-05
Rv1425	1229.18	-0.61	0.12	-5.11	3.31E-07	1.41E-06
Rv1567c	164.04	-0.61	0.21	-2.86	4.21E-03	8.32E-03
Rv0220	3847.14	-0.61	0.14	-4.38	1.21E-05	3.94E-05
Rv2632c	314.49	-0.61	0.18	-3.45	5.58E-04	1.32E-03
Rv1158c	1155.27	-0.61	0.23	-2.64	8.27E-03	1.54E-02
Rv0205	687.44	-0.61	0.12	-4.95	7.61E-07	3.00E-06
Rv3769	621.78	-0.61	0.18	-3.44	5.92E-04	1.39E-03
Rv2881c	1836.50	-0.61	0.14	-4.36	1.29E-05	4.19E-05
Rv3127	13030.99	-0.61	0.27	-2.30	2.16E-02	3.63E-02
Rv0665	56.10	-0.61	0.19	-3.25	1.15E-03	2.58E-03
Rv3404c	642.67	-0.61	0.14	-4.31	1.62E-05	5.16E-05
Rv1272c	1249.38	-0.62	0.10	-6.47	1.00E-10	7.12E-10
Rv1552	224.40	-0.62	0.18	-3.39	6.97E-04	1.61E-03
Rv2376c	1000.85	-0.62	0.17	-3.58	3.49E-04	8.61E-04
Rv2806	51.84	-0.62	0.15	-4.02	5.77E-05	1.66E-04
Rv3828c	251.97	-0.62	0.14	-4.42	9.97E-06	3.30E-05
Rv1701	969.71	-0.62	0.14	-4.55	5.25E-06	1.83E-05
Rv2860c	964.05	-0.62	0.10	-5.90	3.64E-09	2.06E-08
Rv1201c	1207.69	-0.62	0.19	-3.35	8.22E-04	1.88E-03
Rv2080	1006.68	-0.62	0.10	-6.03	1.59E-09	9.52E-09
Rv0446c	533.88	-0.62	0.13	-4.84	1.29E-06	4.92E-06
Rv1292	1045.48	-0.62	0.11	-5.76	8.28E-09	4.46E-08
Rv1523	353.40	-0.62	0.17	-3.63	2.84E-04	7.13E-04
Rv3156	4151.10	-0.63	0.18	-3.48	4.98E-04	1.19E-03
Rv2334	2348.48	-0.63	0.17	-3.64	2.71E-04	6.85E-04
Rvnt15	61.47	-0.63	0.22	-2.83	4.68E-03	9.17E-03
Rv3315c	75.32	-0.63	0.18	-3.45	5.60E-04	1.32E-03

Rv0997a	399.69	-0.63	0.19	-3.33	8.74E-04	1.99E-03
Rv0867c	2877.68	-0.63	0.15	-4.08	4.52E-05	1.33E-04
Rv1366	136.52	-0.63	0.15	-4.28	1.85E-05	5.85E-05
Rv3576	389.58	-0.63	0.12	-5.26	1.47E-07	6.62E-07
Rv1290A	106.39	-0.63	0.16	-3.88	1.06E-04	2.90E-04
Rv1143	664.70	-0.63	0.13	-4.96	7.08E-07	2.81E-06
Rv0359	211.31	-0.63	0.13	-4.77	1.86E-06	6.93E-06
Rv2434c	322.30	-0.63	0.12	-5.08	3.82E-07	1.61E-06
Rv3361c	309.59	-0.63	0.14	-4.46	8.09E-06	2.72E-05
Rv1261c	496.75	-0.63	0.15	-4.27	1.96E-05	6.14E-05
Rv0646c	901.96	-0.64	0.15	-4.21	2.53E-05	7.72E-05
Rv2509	913.44	-0.64	0.14	-4.47	7.65E-06	2.59E-05
Rv0735	222.92	-0.64	0.13	-4.79	1.68E-06	6.28E-06
Rv3460c	3721.24	-0.64	0.17	-3.81	1.39E-04	3.72E-04
Rv1029	361.30	-0.64	0.11	-5.96	2.48E-09	1.44E-08
Rv0163	91.24	-0.64	0.16	-4.02	5.94E-05	1.70E-04
Rv3100c	274.02	-0.64	0.12	-5.37	7.80E-08	3.64E-07
Rv0171	4355.47	-0.65	0.19	-3.44	5.74E-04	1.35E-03
Rv1319c	1249.26	-0.65	0.15	-4.26	2.02E-05	6.32E-05
Rv0695	2531.25	-0.65	0.16	-3.98	6.90E-05	1.97E-04
Rv2112c	2131.72	-0.65	0.15	-4.45	8.61E-06	2.89E-05
Rv2531c	2329.55	-0.65	0.10	-6.70	2.11E-11	1.64E-10
Rv3371	2631.55	-0.65	0.21	-3.14	1.67E-03	3.63E-03
Rv3669	356.75	-0.65	0.15	-4.45	8.69E-06	2.91E-05
Rv3484	2540.38	-0.65	0.13	-5.12	3.04E-07	1.31E-06
Rv2798c	145.22	-0.65	0.14	-4.73	2.28E-06	8.40E-06
Rv0686	1610.31	-0.65	0.21	-3.08	2.10E-03	4.45E-03
Rv1514c	134.60	-0.65	0.16	-4.11	4.00E-05	1.18E-04
Rv2248	1214.29	-0.65	0.18	-3.63	2.88E-04	7.23E-04
Rv0815c	2070.35	-0.65	0.19	-3.46	5.40E-04	1.28E-03

Rv0995	487.14	-0.65	0.10	-6.25	4.10E-10	2.68E-09
Rv1876	917.91	-0.66	0.17	-3.81	1.41E-04	3.77E-04
Rv2119	275.40	-0.66	0.16	-4.11	3.93E-05	1.16E-04
Rv3719	980.84	-0.66	0.15	-4.52	6.27E-06	2.15E-05
Rv2901c	1263.93	-0.66	0.14	-4.73	2.25E-06	8.29E-06
Rv3820c	618.09	-0.66	0.14	-4.70	2.58E-06	9.39E-06
Rv0432	1083.56	-0.66	0.15	-4.44	8.80E-06	2.95E-05
Rv0052	368.83	-0.66	0.09	-7.23	4.81E-13	4.82E-12
Rv2292c	82.14	-0.66	0.16	-4.03	5.60E-05	1.62E-04
Rv2530A	108.33	-0.66	0.19	-3.53	4.22E-04	1.02E-03
Rv0222	685.92	-0.66	0.13	-4.93	8.23E-07	3.23E-06
Rv1283c	507.39	-0.67	0.15	-4.35	1.33E-05	4.29E-05
Rv2740	482.13	-0.67	0.17	-3.90	9.77E-05	2.69E-04
Rv1436	2361.55	-0.67	0.18	-3.79	1.52E-04	4.04E-04
Rv2272	268.79	-0.67	0.14	-4.64	3.47E-06	1.24E-05
Rv1220c	747.14	-0.67	0.22	-3.01	2.60E-03	5.39E-03
Rv2055c	166.50	-0.67	0.14	-4.64	3.49E-06	1.25E-05
Rv3479	2839.35	-0.67	0.12	-5.47	4.63E-08	2.22E-07
Rv1315	1601.05	-0.67	0.11	-6.31	2.78E-10	1.86E-09
Rv3117	1792.96	-0.67	0.19	-3.49	4.89E-04	1.17E-03
Rv0179c	1857.81	-0.67	0.20	-3.34	8.29E-04	1.89E-03
Rv0997	505.16	-0.67	0.15	-4.62	3.88E-06	1.38E-05
Rv2737c	6470.42	-0.68	0.14	-4.92	8.57E-07	3.36E-06
Rv2211c	871.33	-0.68	0.18	-3.74	1.85E-04	4.84E-04
Rv0647c	1276.79	-0.68	0.11	-6.33	2.50E-10	1.68E-09
Rv2282c	111.56	-0.68	0.29	-2.33	1.98E-02	3.35E-02
Rv3241c	1732.19	-0.68	0.16	-4.27	1.96E-05	6.16E-05
Rv2199c	1443.22	-0.68	0.18	-3.76	1.70E-04	4.47E-04
Rv0445c	510.13	-0.68	0.10	-6.48	8.93E-11	6.39E-10
Rv2154c	2757.68	-0.68	0.19	-3.65	2.57E-04	6.54E-04

Rv1856c	1708.60	-0.68	0.17	-3.93	8.35E-05	2.33E-04
Rv0129c	2626.31	-0.68	0.28	-2.44	1.47E-02	2.57E-02
Rv0003	623.48	-0.68	0.16	-4.24	2.22E-05	6.91E-05
Rv1798	3464.41	-0.68	0.13	-5.10	3.47E-07	1.48E-06
Rv2349c	653.11	-0.68	0.21	-3.23	1.23E-03	2.73E-03
Rv2972c	582.49	-0.69	0.13	-5.15	2.65E-07	1.14E-06
Rv3744	366.46	-0.69	0.12	-5.68	1.34E-08	6.93E-08
Rv1081c	200.34	-0.69	0.13	-5.24	1.61E-07	7.21E-07
Rv3884c	1023.59	-0.69	0.11	-6.19	6.01E-10	3.81E-09
Rv0320	188.88	-0.69	0.20	-3.43	5.96E-04	1.40E-03
Rv3499c	635.71	-0.69	0.13	-5.31	1.08E-07	4.93E-07
Rv3593	2343.33	-0.69	0.09	-7.35	1.99E-13	2.14E-12
Rv0937c	562.98	-0.69	0.19	-3.57	3.64E-04	8.93E-04
Rv1111c	1114.44	-0.69	0.10	-6.98	2.89E-12	2.56E-11
Rv3485c	612.17	-0.69	0.16	-4.43	9.23E-06	3.08E-05
Rv0572c	383.50	-0.69	0.23	-3.04	2.36E-03	4.96E-03
Rv3822	1549.51	-0.69	0.17	-4.12	3.81E-05	1.13E-04
Rv2784c	1201.50	-0.69	0.14	-4.88	1.04E-06	4.02E-06
Rv3747	294.76	-0.69	0.21	-3.25	1.15E-03	2.57E-03
Rv1092c	568.55	-0.69	0.16	-4.36	1.30E-05	4.20E-05
Rv1984c	376.08	-0.70	0.16	-4.28	1.90E-05	5.96E-05
Rv3661	1050.53	-0.70	0.13	-5.51	3.51E-08	1.70E-07
Rv0734	1189.34	-0.70	0.16	-4.37	1.25E-05	4.06E-05
Rv3318	613.66	-0.70	0.15	-4.72	2.36E-06	8.65E-06
Rv1515c	143.40	-0.70	0.20	-3.49	4.90E-04	1.17E-03
Rv2902c	2956.35	-0.70	0.11	-6.11	9.81E-10	5.99E-09
Rv2313c	713.50	-0.70	0.18	-3.99	6.63E-05	1.89E-04
Rv1906c	1697.55	-0.70	0.17	-4.13	3.70E-05	1.10E-04
Rv3793	2331.26	-0.70	0.19	-3.78	1.55E-04	4.09E-04
Rv3753c	696.62	-0.70	0.18	-3.95	7.78E-05	2.19E-04

Rv1426c	614.33	-0.71	0.20	-3.53	4.21E-04	1.02E-03
Rv3688c	371.21	-0.71	0.14	-5.11	3.26E-07	1.40E-06
Rv3716c	633.29	-0.71	0.17	-4.05	5.10E-05	1.48E-04
Rv3132c	7418.43	-0.71	0.24	-2.92	3.53E-03	7.10E-03
Rv0237	832.47	-0.71	0.17	-4.23	2.39E-05	7.37E-05
Rv1788	158.96	-0.71	0.13	-5.62	1.91E-08	9.73E-08
Rv0696	1783.52	-0.71	0.17	-4.10	4.21E-05	1.24E-04
Rv1558	411.49	-0.71	0.14	-5.03	4.85E-07	1.99E-06
Rv3263	547.72	-0.71	0.12	-6.17	6.81E-10	4.24E-09
Rv3198c	1608.56	-0.71	0.13	-5.57	2.50E-08	1.24E-07
Rv0822c	5200.38	-0.71	0.17	-4.22	2.39E-05	7.39E-05
Rv2904c	2508.43	-0.71	0.13	-5.33	9.84E-08	4.52E-07
Rv0500A	949.04	-0.71	0.21	-3.45	5.66E-04	1.34E-03
Rv2420c	232.02	-0.71	0.13	-5.31	1.13E-07	5.15E-07
Rv2485c	578.89	-0.71	0.20	-3.51	4.43E-04	1.07E-03
Rv0035	346.59	-0.72	0.18	-3.88	1.05E-04	2.86E-04
Rv3662c	5710.14	-0.72	0.28	-2.59	9.51E-03	1.74E-02
Rv3805c	2373.36	-0.72	0.14	-5.22	1.77E-07	7.87E-07
Rv0427c	357.34	-0.72	0.13	-5.70	1.23E-08	6.44E-08
Rv2171	994.32	-0.72	0.13	-5.53	3.22E-08	1.58E-07
Rv2536	1459.18	-0.72	0.11	-6.84	8.01E-12	6.65E-11
RVnc0021	3440.46	-0.72	0.31	-2.33	2.00E-02	3.39E-02
Rv0655	12663.22	-0.72	0.23	-3.14	1.67E-03	3.64E-03
Rv0315	1622.83	-0.72	0.10	-6.89	5.75E-12	4.91E-11
Rv3136A	1995.53	-0.72	0.23	-3.17	1.51E-03	3.31E-03
Rv0806c	1588.37	-0.72	0.14	-5.20	1.97E-07	8.72E-07
Rv3148	2388.75	-0.72	0.21	-3.52	4.37E-04	1.05E-03
Rv1280c	1135.01	-0.72	0.13	-5.37	7.78E-08	3.63E-07
Rv0229c	1729.24	-0.73	0.22	-3.25	1.16E-03	2.60E-03
Rv2468A	175.80	-0.73	0.19	-3.89	1.01E-04	2.76E-04

Rv0042c	335.70	-0.73	0.16	-4.55	5.27E-06	1.83E-05
Rv1458c	708.76	-0.73	0.13	-5.64	1.73E-08	8.86E-08
Rv3623	375.73	-0.73	0.14	-5.39	6.93E-08	3.25E-07
Rv1282c	306.41	-0.73	0.15	-4.92	8.75E-07	3.42E-06
Rv3782	1600.09	-0.73	0.15	-4.74	2.09E-06	7.71E-06
Rv1097c	1193.73	-0.73	0.12	-5.88	4.07E-09	2.28E-08
Rv3784	533.65	-0.73	0.18	-4.14	3.49E-05	1.04E-04
Rv2260	623.81	-0.73	0.12	-5.96	2.59E-09	1.50E-08
Rv1015c	742.42	-0.73	0.14	-5.08	3.81E-07	1.60E-06
Rv1112	149.96	-0.73	0.14	-5.09	3.54E-07	1.50E-06
Rv0936	737.59	-0.73	0.12	-6.18	6.43E-10	4.06E-09
Rv2593c	568.93	-0.74	0.18	-4.12	3.79E-05	1.12E-04
Rv1260	564.12	-0.74	0.17	-4.27	1.97E-05	6.16E-05
Rv1098c	1441.84	-0.74	0.13	-5.69	1.30E-08	6.79E-08
Rv1738	4606.70	-0.74	0.29	-2.52	1.17E-02	2.10E-02
Rv0110	280.84	-0.74	0.11	-6.97	3.20E-12	2.82E-11
Rv1608c	348.92	-0.74	0.17	-4.49	7.12E-06	2.42E-05
Rv2081c	1122.61	-0.74	0.13	-5.62	1.92E-08	9.74E-08
Rv2427c	1574.09	-0.74	0.09	-8.62	6.98E-18	1.39E-16
Rv2807	84.97	-0.75	0.18	-4.13	3.60E-05	1.07E-04
Rv3403c	772.76	-0.75	0.20	-3.81	1.40E-04	3.73E-04
Rv3043c	2463.74	-0.75	0.19	-3.83	1.26E-04	3.40E-04
Rv2421c	315.48	-0.75	0.13	-5.63	1.82E-08	9.30E-08
Rv3121	500.63	-0.75	0.17	-4.40	1.09E-05	3.58E-05
Rv0155	433.54	-0.75	0.15	-4.89	1.01E-06	3.92E-06
Rv2734	242.74	-0.75	0.15	-4.98	6.30E-07	2.53E-06
Rv1886c	1729.43	-0.75	0.29	-2.56	1.06E-02	1.91E-02
Rv3646c	4248.32	-0.75	0.11	-6.74	1.62E-11	1.28E-10
Rv2144c	1688.46	-0.75	0.12	-6.42	1.35E-10	9.46E-10
Rv2220	2872.58	-0.75	0.21	-3.60	3.15E-04	7.84E-04

Rv3150	2061.51	-0.75	0.20	-3.84	1.23E-04	3.34E-04
Rv3797	739.21	-0.75	0.26	-2.87	4.13E-03	8.18E-03
Rv1848	183.82	-0.75	0.19	-4.01	6.03E-05	1.73E-04
Rv0507	1078.08	-0.75	0.20	-3.70	2.17E-04	5.61E-04
Rv2425c	796.38	-0.75	0.09	-8.11	4.96E-16	8.13E-15
Rv1038c	389.03	-0.75	0.20	-3.78	1.59E-04	4.21E-04
Rv1439c	157.88	-0.75	0.18	-4.11	3.88E-05	1.14E-04
Rv2082	2556.44	-0.76	0.12	-6.27	3.56E-10	2.35E-09
Rv1014c	242.59	-0.76	0.17	-4.44	9.11E-06	3.04E-05
Rv3629c	366.95	-0.76	0.14	-5.59	2.32E-08	1.16E-07
Rv1096	908.32	-0.76	0.12	-6.35	2.18E-10	1.49E-09
Rv3147	1100.68	-0.76	0.19	-3.96	7.52E-05	2.12E-04
Rv3391	774.92	-0.76	0.15	-5.04	4.54E-07	1.88E-06
Rv0799c	471.78	-0.76	0.15	-5.01	5.51E-07	2.25E-06
Rv3393	289.35	-0.76	0.15	-5.02	5.18E-07	2.12E-06
Rv0051	925.40	-0.76	0.09	-8.78	1.61E-18	3.52E-17
Rv3706c	514.79	-0.77	0.20	-3.75	1.74E-04	4.57E-04
Rv1142c	1338.15	-0.77	0.25	-3.03	2.48E-03	5.17E-03
Rv1548c	671.75	-0.77	0.12	-6.62	3.56E-11	2.67E-10
Rv3614c	3999.01	-0.77	0.32	-2.40	1.66E-02	2.88E-02
Rv0466	939.70	-0.77	0.17	-4.60	4.27E-06	1.51E-05
Rv3198A	178.83	-0.77	0.12	-6.38	1.78E-10	1.23E-09
Rv1618	770.16	-0.77	0.13	-5.89	3.97E-09	2.23E-08
Rv0516c	1230.28	-0.77	0.24	-3.15	1.62E-03	3.53E-03
Rv3819	129.79	-0.77	0.23	-3.30	9.63E-04	2.18E-03
Rv3281	978.50	-0.77	0.15	-5.09	3.67E-07	1.55E-06
Rv3304	178.72	-0.78	0.15	-5.20	2.03E-07	8.91E-07
Rv2120c	263.82	-0.78	0.18	-4.43	9.52E-06	3.16E-05
Rv1176c	223.89	-0.78	0.17	-4.49	7.16E-06	2.43E-05
Rv3846	706.46	-0.78	0.21	-3.65	2.61E-04	6.63E-04

Rv0321	212.49	-0.78	0.17	-4.73	2.26E-06	8.32E-06
Rv0566c	114.98	-0.78	0.20	-3.96	7.46E-05	2.11E-04
Rv0697	550.27	-0.78	0.18	-4.43	9.62E-06	3.20E-05
Rv3685c	847.24	-0.78	0.13	-6.12	9.17E-10	5.63E-09
RVnc0024	126.22	-0.78	0.23	-3.40	6.82E-04	1.58E-03
Rv3809c	654.88	-0.78	0.15	-5.06	4.14E-07	1.73E-06
Rv0890c	914.17	-0.78	0.14	-5.68	1.36E-08	7.03E-08
Rv0882	120.12	-0.78	0.16	-5.05	4.53E-07	1.88E-06
Rv2803	94.87	-0.79	0.15	-5.23	1.69E-07	7.54E-07
Rv2779c	263.18	-0.79	0.22	-3.58	3.39E-04	8.37E-04
Rv2567	1375.36	-0.79	0.11	-6.89	5.60E-12	4.80E-11
Rv3781	544.48	-0.79	0.14	-5.68	1.35E-08	6.98E-08
Rv1080c	4984.43	-0.79	0.14	-5.74	9.55E-09	5.12E-08
Rv3236c	778.38	-0.79	0.14	-5.52	3.31E-08	1.61E-07
Rv2339	1339.21	-0.79	0.17	-4.67	2.95E-06	1.07E-05
Rv1816	1508.74	-0.79	0.15	-5.35	8.92E-08	4.12E-07
Rv3157	3378.41	-0.79	0.14	-5.54	2.98E-08	1.47E-07
Rv3234c	733.93	-0.79	0.10	-7.94	2.01E-15	2.84E-14
Rv0073	723.14	-0.80	0.17	-4.69	2.68E-06	9.72E-06
Rv0428c	223.05	-0.80	0.19	-4.29	1.75E-05	5.56E-05
Rv1437	1817.04	-0.80	0.16	-4.98	6.45E-07	2.59E-06
Rv2238c	734.05	-0.80	0.17	-4.78	1.76E-06	6.56E-06
Rv0139	731.20	-0.80	0.12	-6.52	7.07E-11	5.15E-10
Rv3647c	1776.06	-0.80	0.23	-3.51	4.51E-04	1.08E-03
Rv1117	452.95	-0.80	0.17	-4.63	3.65E-06	1.30E-05
Rv3106	480.39	-0.80	0.11	-7.29	3.06E-13	3.18E-12
Rv3632	139.12	-0.80	0.21	-3.91	9.34E-05	2.58E-04
Rv2101	2996.75	-0.80	0.12	-6.66	2.65E-11	2.03E-10
Rv3208	843.84	-0.81	0.14	-5.79	7.04E-09	3.82E-08
Rv1200	1639.07	-0.81	0.11	-7.36	1.79E-13	1.95E-12

Rv0156	199.80	-0.81	0.17	-4.87	1.11E-06	4.26E-06
Rv3362c	347.06	-0.81	0.17	-4.82	1.40E-06	5.31E-06
Rv3107c	647.10	-0.81	0.22	-3.70	2.17E-04	5.61E-04
Rv1171	816.19	-0.81	0.17	-4.83	1.35E-06	5.11E-06
Rv0979A	174.17	-0.81	0.18	-4.63	3.71E-06	1.32E-05
Rv0934	1558.31	-0.81	0.20	-3.97	7.15E-05	2.03E-04
Rvnt03	78.63	-0.82	0.19	-4.19	2.75E-05	8.34E-05
Rv3158	2738.70	-0.82	0.13	-6.53	6.72E-11	4.92E-10
Rv2056c	72.65	-0.82	0.19	-4.35	1.38E-05	4.43E-05
Rv0798c	391.73	-0.82	0.16	-5.02	5.22E-07	2.14E-06
Rv0868c	352.88	-0.82	0.15	-5.46	4.69E-08	2.24E-07
Rv3152	2501.92	-0.82	0.22	-3.73	1.92E-04	5.00E-04
Rv0721	3572.15	-0.83	0.15	-5.68	1.33E-08	6.91E-08
Rv0773c	582.68	-0.83	0.18	-4.57	4.79E-06	1.68E-05
Rv1490	296.38	-0.83	0.12	-6.86	6.91E-12	5.82E-11
Rv3849	887.69	-0.83	0.18	-4.53	5.93E-06	2.04E-05
Rv3311	674.24	-0.83	0.13	-6.25	4.11E-10	2.68E-09
Rv3788	258.13	-0.83	0.17	-4.84	1.33E-06	5.04E-06
Rv1144	2011.74	-0.83	0.27	-3.03	2.46E-03	5.13E-03
Rv2937	1172.34	-0.83	0.11	-7.41	1.29E-13	1.43E-12
Rv1871c	7406.69	-0.83	0.16	-5.07	3.96E-07	1.65E-06
Rv3772	398.16	-0.83	0.11	-7.35	1.99E-13	2.14E-12
Rv2971	1206.44	-0.84	0.17	-4.89	1.01E-06	3.90E-06
Rv2231A	71.57	-0.84	0.17	-4.92	8.84E-07	3.45E-06
Rv1752	237.49	-0.84	0.14	-5.99	2.07E-09	1.22E-08
Rv1885c	486.20	-0.84	0.19	-4.37	1.25E-05	4.06E-05
Rvnt40	83.28	-0.84	0.24	-3.50	4.58E-04	1.10E-03
Rv3741c	291.60	-0.84	0.22	-3.80	1.42E-04	3.78E-04
Rv0801	125.19	-0.84	0.15	-5.55	2.87E-08	1.41E-07
Rv3363c	153.29	-0.84	0.17	-5.09	3.50E-07	1.48E-06

Rv0070c	587.24	-0.84	0.13	-6.55	5.69E-11	4.20E-10
Rv1079	1722.08	-0.84	0.17	-4.84	1.27E-06	4.87E-06
Rv1689	491.97	-0.85	0.15	-5.68	1.34E-08	6.93E-08
Rv2985	1802.71	-0.85	0.11	-8.04	8.92E-16	1.39E-14
Rv3584	1375.92	-0.85	0.15	-5.54	3.10E-08	1.52E-07
Rv0312	1183.85	-0.85	0.23	-3.75	1.75E-04	4.60E-04
Rv3310	562.18	-0.85	0.14	-6.01	1.91E-09	1.13E-08
Rv1262c	312.57	-0.85	0.16	-5.34	9.20E-08	4.24E-07
Rv2426c	727.71	-0.85	0.08	-10.03	1.16E-23	4.15E-22
Rv2435c	351.49	-0.85	0.17	-5.15	2.55E-07	1.10E-06
Rv3313c	416.05	-0.85	0.19	-4.38	1.21E-05	3.94E-05
Rv1646	2102.20	-0.85	0.16	-5.48	4.35E-08	2.09E-07
Rv2720	3382.15	-0.85	0.14	-5.94	2.80E-09	1.61E-08
Rv1824	145.12	-0.85	0.12	-6.93	4.10E-12	3.53E-11
Rv3295	2654.39	-0.85	0.16	-5.27	1.34E-07	6.09E-07
Rv0176	2208.04	-0.85	0.13	-6.46	1.04E-10	7.40E-10
Rv3493c	541.08	-0.86	0.14	-6.25	3.98E-10	2.61E-09
Rv3918c	1786.19	-0.86	0.12	-6.93	4.35E-12	3.74E-11
Rv1511	843.63	-0.86	0.14	-6.17	6.63E-10	4.14E-09
Rv1476	346.54	-0.86	0.17	-5.00	5.61E-07	2.28E-06
Rv3000	237.12	-0.86	0.20	-4.36	1.31E-05	4.25E-05
Rv3125c	664.73	-0.86	0.16	-5.29	1.25E-07	5.69E-07
Rv0460	437.44	-0.86	0.13	-6.64	3.14E-11	2.38E-10
Rv1825	433.58	-0.86	0.12	-6.93	4.07E-12	3.52E-11
Rv3740c	1237.39	-0.86	0.14	-6.07	1.31E-09	7.83E-09
Rv3478	7709.86	-0.86	0.24	-3.59	3.25E-04	8.05E-04
Rv0912	220.99	-0.86	0.11	-7.82	5.34E-15	7.14E-14
Rv0693	5145.57	-0.86	0.25	-3.43	6.11E-04	1.43E-03
Rv3096	1088.48	-0.86	0.12	-7.22	5.39E-13	5.35E-12
Rv2723	493.93	-0.86	0.14	-5.95	2.72E-09	1.57E-08

Rv3282	791.97	-0.86	0.10	-8.34	7.21E-17	1.30E-15
Rv3006	2015.97	-0.86	0.14	-6.37	1.85E-10	1.28E-09
Rv1469	695.33	-0.86	0.12	-7.03	2.03E-12	1.84E-11
Rv1524	340.36	-0.86	0.18	-4.86	1.19E-06	4.55E-06
Rv1502	1317.34	-0.87	0.27	-3.19	1.42E-03	3.13E-03
Rv1418	695.34	-0.87	0.10	-8.40	4.51E-17	8.44E-16
Rv1979c	1417.30	-0.87	0.13	-6.41	1.41E-10	9.82E-10
Rv1794	3100.38	-0.87	0.15	-5.64	1.69E-08	8.66E-08
Rv1902c	833.16	-0.87	0.25	-3.52	4.28E-04	1.04E-03
Rv1107c	272.93	-0.87	0.22	-3.91	9.34E-05	2.58E-04
Rv2856	492.98	-0.87	0.14	-6.35	2.19E-10	1.49E-09
Rv3414c	1389.48	-0.87	0.17	-5.14	2.77E-07	1.19E-06
Rv1438	1144.92	-0.87	0.14	-6.10	1.05E-09	6.37E-09
Rv1047	1440.06	-0.87	0.18	-4.94	7.91E-07	3.12E-06
Rv0642c	1962.33	-0.87	0.18	-4.91	9.15E-07	3.55E-06
Rv3366	124.24	-0.87	0.19	-4.53	5.91E-06	2.04E-05
Rv0722	430.15	-0.88	0.15	-5.81	6.13E-09	3.35E-08
Rv3154	1421.62	-0.88	0.17	-5.24	1.57E-07	7.03E-07
Rv3742c	424.47	-0.88	0.26	-3.38	7.24E-04	1.67E-03
Rv2432c	506.14	-0.88	0.17	-5.10	3.42E-07	1.46E-06
Rv0054	1216.88	-0.88	0.11	-7.84	4.57E-15	6.18E-14
Rv3190A	462.62	-0.88	0.16	-5.56	2.65E-08	1.31E-07
Rv1751	1806.19	-0.88	0.15	-5.83	5.69E-09	3.14E-08
Rv0501	2782.77	-0.88	0.11	-8.00	1.26E-15	1.88E-14
Rv3486	414.75	-0.89	0.23	-3.91	9.35E-05	2.58E-04
Rv2906c	486.55	-0.89	0.17	-5.21	1.85E-07	8.21E-07
Rv1010	1089.99	-0.89	0.18	-4.92	8.83E-07	3.45E-06
Rv3231c	275.48	-0.89	0.15	-5.89	3.93E-09	2.22E-08
Rv0157A	113.35	-0.89	0.19	-4.76	1.94E-06	7.20E-06
Rv3317	199.33	-0.89	0.16	-5.58	2.36E-08	1.18E-07

Rv2905	1623.12	-0.89	0.16	-5.66	1.50E-08	7.69E-08
Rv1444c	347.56	-0.90	0.12	-7.33	2.27E-13	2.41E-12
Rv0720	1052.86	-0.90	0.16	-5.75	8.74E-09	4.69E-08
Rv1512	685.76	-0.90	0.14	-6.55	5.57E-11	4.13E-10
Rv2722	128.68	-0.90	0.13	-6.94	4.01E-12	3.48E-11
Rv2337c	510.33	-0.90	0.19	-4.65	3.31E-06	1.19E-05
Rv3330	1649.89	-0.90	0.19	-4.78	1.74E-06	6.49E-06
Rv2032	2793.31	-0.90	0.27	-3.35	7.94E-04	1.82E-03
Rv0544c	348.20	-0.90	0.13	-6.78	1.19E-11	9.58E-11
Rv2102	1043.02	-0.90	0.10	-8.80	1.43E-18	3.17E-17
Rv0685	9775.35	-0.90	0.20	-4.63	3.67E-06	1.31E-05
Rv0158	351.72	-0.90	0.15	-6.24	4.38E-10	2.85E-09
Rv1826	912.04	-0.91	0.15	-5.88	4.10E-09	2.30E-08
Rv1645c	1553.32	-0.91	0.14	-6.33	2.53E-10	1.70E-09
Rv2510c	603.95	-0.91	0.12	-7.28	3.36E-13	3.44E-12
Rv0346c	1971.26	-0.91	0.18	-4.94	7.97E-07	3.14E-06
Rv3397c	126.79	-0.91	0.22	-4.13	3.58E-05	1.07E-04
Rv3416	723.51	-0.91	0.18	-4.91	9.11E-07	3.54E-06
Rv3319	405.17	-0.91	0.14	-6.68	2.46E-11	1.90E-10
Rv1509	486.04	-0.91	0.21	-4.28	1.87E-05	5.88E-05
Rv1318c	1565.91	-0.91	0.26	-3.49	4.80E-04	1.15E-03
Rv3280	2474.81	-0.91	0.16	-5.79	7.04E-09	3.82E-08
Rv0455c	1335.90	-0.91	0.16	-5.72	1.06E-08	5.62E-08
Rv1550	312.96	-0.91	0.16	-5.60	2.19E-08	1.10E-07
Rv1321	1096.47	-0.91	0.08	-11.69	1.45E-31	1.11E-29
Rv0780	220.73	-0.91	0.20	-4.66	3.18E-06	1.15E-05
Rv3044	1693.71	-0.91	0.17	-5.32	1.06E-07	4.85E-07
Rv1638A	260.10	-0.92	0.16	-5.81	6.14E-09	3.35E-08
Rv0115	236.95	-0.92	0.14	-6.60	4.07E-11	3.05E-10
Rv2928	390.67	-0.92	0.21	-4.35	1.37E-05	4.40E-05

Rv2944	140.34	-0.92	0.18	-5.08	3.69E-07	1.56E-06
Rv3705c	754.42	-0.92	0.20	-4.65	3.39E-06	1.22E-05
Rv2563	367.79	-0.92	0.17	-5.50	3.90E-08	1.88E-07
Rv1006	3906.09	-0.92	0.16	-5.67	1.45E-08	7.48E-08
Rv3023c	1323.42	-0.92	0.19	-4.97	6.85E-07	2.73E-06
Rv3115	1348.66	-0.92	0.18	-5.09	3.57E-07	1.51E-06
Rv3476c	267.16	-0.92	0.18	-5.20	1.99E-07	8.80E-07
Rv2442c	601.89	-0.92	0.24	-3.89	9.84E-05	2.70E-04
Rv0896	3411.35	-0.93	0.20	-4.53	5.93E-06	2.04E-05
Rv0519c	476.78	-0.93	0.21	-4.36	1.29E-05	4.18E-05
Rv3634c	868.80	-0.93	0.20	-4.66	3.14E-06	1.13E-05
Rv2679	546.98	-0.93	0.30	-3.09	2.01E-03	4.29E-03
Rv1953	111.78	-0.93	0.21	-4.45	8.59E-06	2.88E-05
Rv0069c	743.39	-0.93	0.13	-7.17	7.50E-13	7.36E-12
Rv2773c	685.61	-0.93	0.13	-7.27	3.70E-13	3.77E-12
Rv2293c	341.75	-0.94	0.17	-5.37	7.93E-08	3.69E-07
Rv2416c	342.15	-0.94	0.20	-4.64	3.54E-06	1.26E-05
Rv2512c	1856.69	-0.94	0.21	-4.47	7.90E-06	2.66E-05
Rv1099c	824.73	-0.94	0.14	-6.86	7.08E-12	5.91E-11
Rv0920c	452.73	-0.94	0.13	-7.43	1.10E-13	1.23E-12
Rv3390	577.73	-0.94	0.14	-6.82	9.37E-12	7.66E-11
Rv0698	310.94	-0.94	0.16	-5.78	7.52E-09	4.06E-08
Rv2190c	4993.30	-0.94	0.22	-4.34	1.40E-05	4.49E-05
Rv0395	79.23	-0.95	0.16	-6.08	1.21E-09	7.26E-09
Rv3146	800.16	-0.95	0.19	-4.99	5.94E-07	2.41E-06
Rv2386a	459.16	-0.95	0.21	-4.60	4.31E-06	1.52E-05
Rv1948c	174.45	-0.95	0.22	-4.41	1.01E-05	3.34E-05
Rv1314c	275.45	-0.95	0.12	-8.06	7.80E-16	1.23E-14
Rv2882c	1016.06	-0.95	0.18	-5.26	1.41E-07	6.36E-07
Rv2273	71.06	-0.96	0.21	-4.47	7.81E-06	2.63E-05

Rv0682	2538.30	-0.96	0.21	-4.52	6.31E-06	2.16E-05
Rv0506	289.31	-0.96	0.17	-5.59	2.28E-08	1.14E-07
Rv0692	1192.45	-0.96	0.30	-3.19	1.41E-03	3.10E-03
Rv2808	91.82	-0.97	0.33	-2.91	3.61E-03	7.24E-03
Rv3339c	1168.57	-0.97	0.38	-2.55	1.09E-02	1.96E-02
Rv2992c	980.32	-0.97	0.11	-8.76	1.90E-18	4.12E-17
Rv3331	1092.78	-0.97	0.16	-5.91	3.37E-09	1.92E-08
Rv1881c	515.88	-0.97	0.12	-8.11	5.10E-16	8.32E-15
Rv0434	746.32	-0.97	0.10	-9.26	2.09E-20	5.70E-19
Rv2633c	422.90	-0.97	0.22	-4.33	1.50E-05	4.81E-05
Rv3277	729.37	-0.97	0.19	-5.05	4.32E-07	1.79E-06
Rv2068c	615.35	-0.97	0.14	-7.18	7.07E-13	6.98E-12
Rv0002	973.94	-0.97	0.24	-3.99	6.64E-05	1.89E-04
Rv1478	1120.62	-0.98	0.21	-4.74	2.13E-06	7.83E-06
Rv1903	161.77	-0.98	0.15	-6.55	5.69E-11	4.20E-10
Rv0402c	661.02	-0.98	0.15	-6.41	1.49E-10	1.03E-09
Rv1630	8209.82	-0.98	0.17	-5.93	3.11E-09	1.78E-08
Rv0009	2002.36	-0.98	0.21	-4.64	3.43E-06	1.23E-05
Rv3116	570.29	-0.98	0.18	-5.43	5.50E-08	2.61E-07
Rv3676	2479.39	-0.99	0.17	-5.94	2.78E-09	1.60E-08
Rv0919	547.76	-0.99	0.14	-6.82	8.93E-12	7.33E-11
Rv1016c	610.73	-0.99	0.14	-6.95	3.76E-12	3.28E-11
Rv0701	7720.82	-0.99	0.18	-5.42	5.90E-08	2.79E-07
Rv2958c	479.11	-0.99	0.14	-7.04	1.86E-12	1.70E-11
Rv3398c	174.80	-1.00	0.21	-4.70	2.65E-06	9.63E-06
Rv0502	5897.02	-1.00	0.14	-7.14	9.57E-13	9.12E-12
Rv3477	1004.84	-1.00	0.29	-3.45	5.70E-04	1.35E-03
Rv0911	436.90	-1.00	0.11	-9.16	5.16E-20	1.33E-18
Rv3057c	600.72	-1.00	0.10	-10.46	1.28E-25	5.81E-24
Rv2929	172.07	-1.00	0.24	-4.25	2.14E-05	6.67E-05

Rv2075c	1610.89	-1.00	0.13	-8.02	1.09E-15	1.68E-14
Rv1160	153.92	-1.01	0.16	-6.11	9.85E-10	6.00E-09
Rv3919c	1465.55	-1.01	0.16	-6.17	6.63E-10	4.14E-09
Rv1017c	1309.01	-1.01	0.16	-6.24	4.28E-10	2.78E-09
Rv1198	4187.91	-1.01	0.24	-4.16	3.17E-05	9.57E-05
Rv2237A	277.28	-1.01	0.16	-6.52	6.99E-11	5.12E-10
Rv0892	541.69	-1.01	0.22	-4.63	3.62E-06	1.29E-05
Rv2757c	109.97	-1.02	0.20	-4.98	6.39E-07	2.57E-06
Rv2445c	432.33	-1.02	0.19	-5.45	4.92E-08	2.34E-07
Rv0888	772.93	-1.02	0.15	-6.72	1.85E-11	1.45E-10
Rv3329	635.66	-1.02	0.20	-5.18	2.20E-07	9.60E-07
Rv3687c	261.86	-1.03	0.13	-7.76	8.76E-15	1.14E-13
Rv0714	1260.67	-1.03	0.17	-6.13	8.89E-10	5.48E-09
Rv1316c	1471.78	-1.03	0.27	-3.74	1.82E-04	4.75E-04
Rv3528c	238.61	-1.03	0.20	-5.19	2.08E-07	9.12E-07
Rv1872c	26305.56	-1.03	0.14	-7.11	1.20E-12	1.13E-11
Rv2857c	465.59	-1.03	0.09	-11.64	2.67E-31	1.93E-29
Rv1199c	2269.15	-1.03	0.21	-4.81	1.50E-06	5.66E-06
Rv0925c	351.19	-1.03	0.19	-5.41	6.45E-08	3.04E-07
Rv2861c	1291.23	-1.03	0.14	-7.29	3.17E-13	3.28E-12
Rv2938	1082.78	-1.04	0.12	-8.34	7.62E-17	1.37E-15
Rv3631	250.87	-1.04	0.20	-5.09	3.51E-07	1.49E-06
Rv2288	81.61	-1.04	0.23	-4.50	6.92E-06	2.36E-05
Rv3278c	482.94	-1.04	0.21	-5.03	4.95E-07	2.03E-06
Rv2375	554.39	-1.04	0.13	-8.00	1.24E-15	1.85E-14
Rv3487c	1572.32	-1.05	0.25	-4.18	2.90E-05	8.79E-05
Rv0221	1435.61	-1.05	0.13	-8.01	1.17E-15	1.77E-14
Rv1252c	253.35	-1.05	0.18	-5.77	7.98E-09	4.30E-08
Rv0227c	911.20	-1.05	0.17	-6.28	3.48E-10	2.29E-09
Rvnt06	113.40	-1.05	0.26	-3.96	7.50E-05	2.12E-04

Rv2429	1223.14	-1.05	0.16	-6.69	2.27E-11	1.76E-10
Rv2430c	2199.73	-1.05	0.14	-7.71	1.30E-14	1.60E-13
Rv1566c	1089.96	-1.05	0.23	-4.58	4.71E-06	1.65E-05
Rv0174	7250.24	-1.06	0.16	-6.77	1.25E-11	1.01E-10
Rv1980c	2013.35	-1.06	0.18	-5.81	6.09E-09	3.34E-08
Rv1565c	2233.82	-1.06	0.12	-8.74	2.43E-18	5.15E-17
Rv1534	949.78	-1.06	0.20	-5.22	1.75E-07	7.79E-07
Rv0893c	173.15	-1.06	0.22	-4.79	1.63E-06	6.12E-06
Rv1682	632.19	-1.06	0.17	-6.43	1.31E-10	9.23E-10
Rv2418c	306.91	-1.06	0.21	-5.06	4.30E-07	1.79E-06
Rv0364	384.45	-1.07	0.12	-8.87	7.14E-19	1.66E-17
Rv0719	2965.68	-1.07	0.18	-5.99	2.04E-09	1.20E-08
Rv0059	531.59	-1.07	0.13	-8.36	6.45E-17	1.18E-15
Rv0208c	851.08	-1.07	0.15	-7.04	1.86E-12	1.70E-11
Rv3675	1398.31	-1.07	0.23	-4.69	2.79E-06	1.01E-05
Rv1739c	4482.83	-1.07	0.22	-4.92	8.63E-07	3.38E-06
Rv0636	837.27	-1.07	0.17	-6.47	9.50E-11	6.79E-10
Rv2431c	1939.82	-1.07	0.19	-5.60	2.16E-08	1.09E-07
Rv1998c	1285.35	-1.07	0.25	-4.38	1.18E-05	3.84E-05
Rv3783	856.22	-1.08	0.17	-6.35	2.20E-10	1.50E-09
Rv2965c	304.63	-1.08	0.12	-9.21	3.25E-20	8.47E-19
Rv0053	668.20	-1.08	0.17	-6.44	1.18E-10	8.34E-10
Rv0461	495.42	-1.08	0.16	-6.55	5.76E-11	4.24E-10
Rv3598c	666.95	-1.08	0.11	-10.06	7.93E-24	2.89E-22
Rv2594c	861.96	-1.08	0.14	-7.78	7.41E-15	9.73E-14
Rv2564	326.84	-1.08	0.19	-5.68	1.35E-08	7.00E-08
Rv1632c	626.12	-1.08	0.11	-10.10	5.65E-24	2.10E-22
Rv2258c	1110.28	-1.08	0.14	-7.96	1.67E-15	2.41E-14
Rv2006	9411.31	-1.08	0.22	-4.96	7.02E-07	2.80E-06
Rv2721c	4543.11	-1.09	0.14	-8.01	1.14E-15	1.73E-14

Rv2680	993.47	-1.09	0.14	-7.85	4.32E-15	5.89E-14
Rv0702	7911.21	-1.09	0.18	-6.00	1.93E-09	1.14E-08
Rv2927c	2000.93	-1.09	0.20	-5.57	2.57E-08	1.28E-07
Rv2939	2665.31	-1.09	0.12	-8.96	3.18E-19	7.83E-18
Rv1115	117.82	-1.09	0.13	-8.19	2.59E-16	4.41E-15
Rv0715	1244.74	-1.10	0.19	-5.72	1.07E-08	5.69E-08
Rv3376	1177.75	-1.10	0.16	-6.86	7.03E-12	5.89E-11
Rv3425	500.96	-1.10	0.26	-4.25	2.11E-05	6.57E-05
Rv0138	209.75	-1.10	0.16	-6.79	1.12E-11	9.05E-11
Rv1386	1607.08	-1.10	0.18	-6.23	4.53E-10	2.93E-09
Rv3260c	1496.79	-1.10	0.29	-3.79	1.50E-04	3.99E-04
Rv0883c	1154.51	-1.10	0.20	-5.50	3.79E-08	1.83E-07
Rv1271c	395.68	-1.10	0.11	-10.26	1.11E-24	4.48E-23
Rv0128	268.15	-1.10	0.21	-5.16	2.42E-07	1.05E-06
Rv0108c	1994.47	-1.11	0.21	-5.16	2.42E-07	1.05E-06
Rv1508A	95.15	-1.11	0.28	-3.91	9.25E-05	2.56E-04
Rv0894	228.55	-1.11	0.15	-7.16	8.20E-13	7.91E-12
Rv2200c	3258.83	-1.11	0.19	-5.71	1.16E-08	6.07E-08
Rv1320c	1481.73	-1.11	0.09	-12.12	8.63E-34	7.61E-32
Rv1508c	1263.11	-1.11	0.20	-5.61	2.03E-08	1.03E-07
Rv0431	451.81	-1.12	0.19	-5.89	3.98E-09	2.23E-08
Rv3153	1230.16	-1.12	0.20	-5.74	9.64E-09	5.16E-08
Rv3316	126.79	-1.12	0.18	-6.20	5.55E-10	3.56E-09
Rv3210c	611.89	-1.12	0.16	-7.21	5.43E-13	5.38E-12
Rv3714c	363.87	-1.12	0.13	-8.87	7.39E-19	1.70E-17
Rv0684	12021.11	-1.13	0.19	-5.87	4.24E-09	2.37E-08
Rv0831c	1939.99	-1.13	0.18	-6.30	2.99E-10	2.00E-09
Rv3795	3686.81	-1.13	0.39	-2.89	3.89E-03	7.77E-03
Rv3232c	549.90	-1.13	0.15	-7.70	1.40E-14	1.71E-13
Rv0716	2115.73	-1.14	0.19	-5.96	2.53E-09	1.47E-08

Rv2127	2316.71	-1.14	0.19	-6.01	1.81E-09	1.07E-08
Rv3789	157.30	-1.14	0.22	-5.20	2.00E-07	8.80E-07
Rv2331	82.64	-1.15	0.33	-3.46	5.42E-04	1.28E-03
Rv0732	1256.53	-1.15	0.17	-6.63	3.31E-11	2.49E-10
Rv3137	1248.86	-1.15	0.20	-5.78	7.50E-09	4.06E-08
Rv0700	3751.17	-1.16	0.20	-5.69	1.30E-08	6.78E-08
Rv2329c	719.55	-1.16	0.32	-3.66	2.56E-04	6.51E-04
Rv1815	1845.26	-1.16	0.22	-5.36	8.21E-08	3.81E-07
Rv0010c	1169.58	-1.16	0.16	-7.14	9.18E-13	8.78E-12
Rv2948c	1413.14	-1.16	0.20	-5.89	3.89E-09	2.19E-08
Rv0683	3086.91	-1.16	0.21	-5.57	2.49E-08	1.24E-07
Rv3491	1574.95	-1.16	0.16	-7.41	1.30E-13	1.45E-12
Rv3058c	772.63	-1.17	0.18	-6.59	4.36E-11	3.25E-10
Rv0644c	490.76	-1.17	0.17	-6.88	5.99E-12	5.09E-11
Rv0072	751.84	-1.17	0.13	-8.88	6.81E-19	1.60E-17
Rv1365c	167.70	-1.17	0.27	-4.42	9.81E-06	3.25E-05
Rv3790	468.73	-1.18	0.23	-5.06	4.27E-07	1.78E-06
Rv3126c	85.33	-1.18	0.20	-5.77	7.92E-09	4.27E-08
Rv0909	229.75	-1.18	0.17	-6.76	1.38E-11	1.10E-10
Rv0403c	87.00	-1.18	0.16	-7.41	1.29E-13	1.43E-12
Rv3620c	550.26	-1.18	0.24	-4.97	6.80E-07	2.71E-06
Rv3145	1061.05	-1.18	0.19	-6.20	5.63E-10	3.61E-09
Rv2993c	650.99	-1.18	0.14	-8.50	1.85E-17	3.61E-16
Rv0404	581.05	-1.18	0.19	-6.28	3.33E-10	2.21E-09
Rv2277c	186.34	-1.18	0.19	-6.26	3.88E-10	2.55E-09
Rv0055	273.27	-1.19	0.15	-7.85	4.03E-15	5.51E-14
Rv2331A	64.30	-1.19	0.30	-3.91	9.04E-05	2.51E-04
Rv1197	3795.71	-1.19	0.25	-4.81	1.51E-06	5.69E-06
Rv3004	492.94	-1.19	0.17	-6.84	7.77E-12	6.46E-11
Rv3312b	53.97	-1.19	0.28	-4.24	2.24E-05	6.95E-05

Rv0011c	745.89	-1.19	0.16	-7.49	7.13E-14	8.15E-13
Rv0056	698.92	-1.19	0.15	-7.82	5.23E-15	7.01E-14
Rv2007c	6787.99	-1.20	0.28	-4.22	2.50E-05	7.66E-05
Rv1919c	347.73	-1.20	0.20	-6.10	1.05E-09	6.35E-09
Rv2525c	799.22	-1.21	0.22	-5.45	4.92E-08	2.34E-07
Rv1116	72.09	-1.21	0.21	-5.85	4.85E-09	2.69E-08
Rv1513	362.10	-1.21	0.17	-7.22	5.33E-13	5.31E-12
Rv1196	11483.29	-1.21	0.22	-5.57	2.61E-08	1.29E-07
Rv2338c	303.70	-1.21	0.26	-4.58	4.70E-06	1.65E-05
Rv2950c	2032.02	-1.21	0.18	-6.66	2.68E-11	2.04E-10
Rv1182	1606.07	-1.21	0.22	-5.40	6.76E-08	3.18E-07
Rv3924c	270.63	-1.21	0.22	-5.64	1.71E-08	8.75E-08
Rv3312A	205.22	-1.22	0.28	-4.37	1.24E-05	4.04E-05
Rv0429c	238.92	-1.22	0.14	-8.75	2.21E-18	4.72E-17
Rv0177	1234.14	-1.22	0.15	-8.27	1.32E-16	2.30E-15
Rv0433	1055.44	-1.22	0.12	-10.15	3.42E-24	1.28E-22
Rv3258c	931.38	-1.22	0.20	-6.11	9.82E-10	5.99E-09
Rv2128	302.82	-1.22	0.17	-7.02	2.26E-12	2.02E-11
Rv2074	1093.28	-1.23	0.19	-6.43	1.26E-10	8.85E-10
Rv1613	1856.36	-1.24	0.17	-7.49	7.01E-14	8.03E-13
Rv0500	439.10	-1.24	0.15	-8.03	9.74E-16	1.51E-14
Rv0948c	452.90	-1.24	0.19	-6.37	1.85E-10	1.28E-09
Rv0178	1586.27	-1.25	0.12	-10.55	4.90E-26	2.34E-24
Rv2955c	514.86	-1.25	0.15	-8.20	2.33E-16	3.99E-15
Rv1433	587.47	-1.25	0.18	-7.05	1.84E-12	1.69E-11
Rv0694	4957.58	-1.26	0.20	-6.23	4.69E-10	3.03E-09
Rv0691A	166.84	-1.26	0.30	-4.21	2.54E-05	7.75E-05
Rv3378c	184.00	-1.27	0.27	-4.67	3.00E-06	1.08E-05
Rv0713	697.35	-1.27	0.15	-8.38	5.26E-17	9.80E-16
Rv1184c	1292.73	-1.27	0.25	-5.08	3.87E-07	1.62E-06

Rv2069	383.89	-1.27	0.16	-7.81	5.69E-15	7.55E-14
Rv3322c	518.78	-1.27	0.38	-3.36	7.69E-04	1.77E-03
Rv3492c	395.70	-1.27	0.16	-7.95	1.84E-15	2.64E-14
Rv0708	1992.67	-1.28	0.21	-6.16	7.06E-10	4.39E-09
Rv0680c	1541.08	-1.28	0.49	-2.60	9.23E-03	1.69E-02
Rv3811	2712.32	-1.28	0.15	-8.73	2.47E-18	5.21E-17
Rv0114	132.27	-1.28	0.17	-7.44	1.01E-13	1.14E-12
Rv2271	205.49	-1.29	0.18	-7.10	1.20E-12	1.13E-11
Rv2289	292.62	-1.29	0.19	-6.65	2.94E-11	2.23E-10
Rv3323c	516.41	-1.29	0.39	-3.29	1.00E-03	2.26E-03
Rv2959c	567.26	-1.29	0.21	-6.01	1.89E-09	1.12E-08
Rv1904	1300.20	-1.30	0.16	-7.87	3.59E-15	4.98E-14
Rv0503c	1391.56	-1.30	0.17	-7.67	1.66E-14	2.01E-13
Rv0236A	746.64	-1.30	0.15	-8.58	9.48E-18	1.87E-16
Rv3796	566.48	-1.30	0.28	-4.71	2.44E-06	8.91E-06
Rv0634A	407.19	-1.30	0.24	-5.51	3.64E-08	1.76E-07
Rv1547	3539.14	-1.30	0.12	-10.43	1.74E-25	7.74E-24
Rv1477	3024.04	-1.31	0.19	-6.83	8.45E-12	6.95E-11
Rv3791	386.24	-1.31	0.22	-6.07	1.27E-09	7.65E-09
Rv1185c	3085.18	-1.31	0.21	-6.20	5.64E-10	3.61E-09
Rv3727	1097.36	-1.32	0.21	-6.21	5.23E-10	3.37E-09
Rv2387	1032.12	-1.32	0.16	-8.35	6.86E-17	1.25E-15
Rv3464	1927.15	-1.32	0.14	-9.51	1.85E-21	5.56E-20
Rv0891c	328.32	-1.33	0.30	-4.42	1.01E-05	3.33E-05
Rv0167	2178.37	-1.33	0.22	-6.11	9.97E-10	6.06E-09
Rv0113	205.44	-1.33	0.20	-6.72	1.77E-11	1.39E-10
Rv3612c	788.23	-1.34	0.29	-4.64	3.55E-06	1.26E-05
Rv1830	1626.89	-1.34	0.18	-7.52	5.43E-14	6.30E-13
Rv1882c	895.93	-1.35	0.14	-9.37	7.08E-21	1.99E-19
Rv3686c	616.98	-1.35	0.33	-4.06	4.83E-05	1.41E-04

Rv0707	3873.19	-1.35	0.19	-7.14	9.03E-13	8.68E-12
Rv2189c	800.16	-1.36	0.25	-5.40	6.48E-08	3.05E-07
Rv1611	2395.02	-1.36	0.16	-8.75	2.05E-18	4.40E-17
Rv0709	682.34	-1.36	0.25	-5.35	8.86E-08	4.10E-07
Rv2778c	431.68	-1.37	0.18	-7.67	1.74E-14	2.10E-13
Rv3465	693.07	-1.37	0.13	-10.34	4.53E-25	1.98E-23
Rv3823c	4430.08	-1.37	0.14	-9.56	1.17E-21	3.65E-20
Rv2257c	539.58	-1.37	0.12	-11.24	2.45E-29	1.50E-27
Rv0705	2306.47	-1.38	0.20	-7.04	1.86E-12	1.70E-11
Rv2816c	232.32	-1.38	0.34	-4.00	6.43E-05	1.84E-04
Rv3920c	2520.65	-1.38	0.16	-8.42	3.83E-17	7.30E-16
Rv3155	208.00	-1.38	0.19	-7.11	1.20E-12	1.13E-11
Rv0706	2768.77	-1.39	0.18	-7.80	6.36E-15	8.40E-14
Rv3826	797.25	-1.39	0.17	-7.98	1.43E-15	2.10E-14
Rv1195	4166.38	-1.40	0.24	-5.92	3.31E-09	1.88E-08
Rv2067c	631.53	-1.40	0.18	-7.83	5.00E-15	6.72E-14
Rv2456c	1180.03	-1.40	0.14	-10.03	1.09E-23	3.91E-22
Rv2147c	2248.01	-1.40	0.18	-7.70	1.41E-14	1.72E-13
Rv0718	1771.02	-1.40	0.16	-8.64	5.49E-18	1.10E-16
Rv1507A	294.74	-1.41	0.19	-7.55	4.35E-14	5.09E-13
Rv1857	678.29	-1.41	0.20	-6.87	6.23E-12	5.29E-11
Rv2457c	8364.53	-1.41	0.19	-7.57	3.87E-14	4.56E-13
Rv3424c	1330.12	-1.42	0.19	-7.37	1.74E-13	1.91E-12
Rv1506c	143.58	-1.42	0.19	-7.43	1.06E-13	1.19E-12
Rv3717	744.90	-1.43	0.20	-7.06	1.61E-12	1.49E-11
Rv0704	5489.20	-1.43	0.19	-7.36	1.79E-13	1.96E-12
Rv3651	736.80	-1.44	0.17	-8.67	4.44E-18	8.99E-17
Rv3613c	797.69	-1.44	0.31	-4.67	3.04E-06	1.10E-05
Rv2076c	480.23	-1.45	0.25	-5.71	1.12E-08	5.91E-08
Rv1440	302.65	-1.45	0.18	-8.27	1.32E-16	2.30E-15

Rv0144	2012.24	-1.46	0.21	-7.02	2.29E-12	2.05E-11
Rvnt07	99.88	-1.46	0.27	-5.42	6.03E-08	2.85E-07
Rv1614	2724.94	-1.46	0.14	-10.68	1.25E-26	6.37E-25
Rv1690	136.35	-1.48	0.19	-7.71	1.23E-14	1.52E-13
Rv3005c	2362.34	-1.48	0.19	-7.73	1.03E-14	1.31E-13
Rv1183	2963.70	-1.48	0.19	-7.86	3.81E-15	5.25E-14
Rv0634B	257.83	-1.49	0.18	-8.34	7.71E-17	1.38E-15
Rv3320c	422.72	-1.49	0.16	-9.55	1.27E-21	3.90E-20
Rv1505c	193.10	-1.50	0.18	-8.25	1.56E-16	2.72E-15
Rv0717	559.42	-1.51	0.29	-5.27	1.35E-07	6.09E-07
Rv0703	1633.65	-1.51	0.20	-7.74	1.03E-14	1.31E-13
Rv0057	525.75	-1.52	0.13	-11.80	3.90E-32	3.22E-30
Rv1371	257.44	-1.52	0.25	-6.03	1.67E-09	9.93E-09
Rv0637	1116.39	-1.53	0.18	-8.44	3.10E-17	5.93E-16
Rv0173	2215.73	-1.53	0.21	-7.36	1.87E-13	2.03E-12
Rv2951c	1073.57	-1.53	0.15	-10.00	1.57E-23	5.57E-22
Rv2428	1163.52	-1.54	0.25	-6.17	6.79E-10	4.24E-09
Rv1360	1212.48	-1.54	0.12	-12.38	3.16E-35	3.30E-33
Rv3377c	953.00	-1.54	0.21	-7.38	1.53E-13	1.68E-12
Rv0164	584.12	-1.55	0.17	-8.93	4.39E-19	1.06E-17
Rv1870c	3628.18	-1.56	0.20	-7.63	2.33E-14	2.80E-13
Rv3392c	231.23	-1.56	0.18	-8.41	3.93E-17	7.47E-16
Rv2954c	1324.66	-1.56	0.21	-7.25	4.07E-13	4.12E-12
Rv0635	572.11	-1.56	0.18	-8.72	2.89E-18	5.98E-17
Rv3824c	1436.71	-1.57	0.24	-6.51	7.68E-11	5.57E-10
Rv0172	3825.61	-1.57	0.22	-7.10	1.29E-12	1.20E-11
Rv1612	1748.90	-1.58	0.19	-8.18	2.92E-16	4.96E-15
Rv0165c	1018.26	-1.58	0.16	-10.15	3.28E-24	1.24E-22
Rv2353c	984.38	-1.59	0.30	-5.29	1.25E-07	5.67E-07
Rv0643c	779.86	-1.60	0.19	-8.37	5.83E-17	1.07E-15

Rv0111	1282.09	-1.60	0.21	-7.75	9.45E-15	1.21E-13
Rv0430	329.80	-1.60	0.26	-6.06	1.37E-09	8.22E-09
Rv2956	776.42	-1.62	0.23	-7.00	2.59E-12	2.31E-11
Rv2276	344.89	-1.63	0.22	-7.50	6.30E-14	7.25E-13
RVnc0040	90.73	-1.64	0.27	-6.18	6.59E-10	4.13E-09
Rv2949c	581.03	-1.64	0.21	-7.99	1.34E-15	1.97E-14
Rv2275	191.11	-1.65	0.23	-7.03	2.07E-12	1.86E-11
Rv2953	909.37	-1.65	0.21	-7.74	1.03E-14	1.31E-13
Rv3324c	429.05	-1.65	0.49	-3.40	6.64E-04	1.54E-03
Rv3923c	795.75	-1.66	0.20	-8.31	9.58E-17	1.69E-15
Rv2952	585.08	-1.67	0.16	-10.34	4.84E-25	2.06E-23
Rv0710	885.49	-1.67	0.22	-7.54	4.78E-14	5.56E-13
Rv0169	2388.23	-1.68	0.24	-7.12	1.12E-12	1.05E-11
Rv0166	1897.67	-1.71	0.28	-6.14	8.05E-10	4.98E-09
Rv1698	834.60	-1.71	0.18	-9.65	5.04E-22	1.64E-20
Rv2336	498.37	-1.71	0.23	-7.55	4.38E-14	5.11E-13
Rv0170	1929.57	-1.72	0.21	-8.21	2.23E-16	3.83E-15
Rv3921c	3484.08	-1.72	0.21	-8.05	8.50E-16	1.33E-14
Rv2350c	733.18	-1.73	0.23	-7.56	4.15E-14	4.87E-13
Rv3135	1669.82	-1.74	0.17	-10.23	1.47E-24	5.83E-23
Rv3321c	122.40	-1.76	0.30	-5.87	4.39E-09	2.45E-08
Rv2817c	971.63	-1.77	0.36	-4.97	6.67E-07	2.66E-06
Rv0933	712.43	-1.77	0.18	-9.65	4.83E-22	1.58E-20
Rv0112	289.31	-1.80	0.22	-8.08	6.72E-16	1.08E-14
Rv0168	1044.18	-1.81	0.23	-7.90	2.70E-15	3.76E-14
Rv3209	1028.87	-1.82	0.19	-9.69	3.34E-22	1.11E-20
Rv3922c	576.36	-1.85	0.23	-8.08	6.61E-16	1.06E-14
Rv1883c	2116.76	-1.86	0.25	-7.48	7.21E-14	8.22E-13
Rv1507c	270.47	-1.86	0.23	-8.17	3.16E-16	5.33E-15
Rv1987	771.44	-1.90	0.26	-7.31	2.64E-13	2.77E-12

Rv0001	3078.07	-1.94	0.25	-7.73	1.08E-14	1.36E-13
Rv1697	1917.95	-1.97	0.21	-9.22	2.98E-20	7.94E-19
Rv1884c	2692.72	-2.07	0.30	-6.79	1.10E-11	8.96E-11
Rv2957	737.98	-2.07	0.18	-11.78	4.91E-32	3.89E-30

Differential gene analysis was conducted in DESeq2 [73] with framework analysis including both clinical isolates and H37Rv and interaction effect between condition and genotype. Sensitivity analysis excluded BDQ-exposed isolate S5 and its corresponding control.
